# Assessing the global dengue burden: Incidence, mortality, and disability trends over three decades

**DOI:** 10.1371/journal.pntd.0012932

**Published:** 2025-03-12

**Authors:** Wei-Xian Zhang, Tian-Yu Zhao, Cun-Chen Wang, Yong He, Hong-Zheng Lu, Hai-Ting Zhang, Lin-Min Wang, Mao Zhang, Chun-Xiao Li, Sheng-Qun Deng

**Affiliations:** 1 Department of Pathology, The Second Affiliated Hospital of Anhui Medical University, Hefei, China,; 2 State Key Laboratory of Pathogen and Biosecurity, Beijing Institute of Microbiology and Epidemiology, Beijing, China,; 3 Department of Pathogen Biology, Anhui Province Key Laboratory of Zoonoses, The Provincial Key Laboratory of Zoonoses of High Institutions in Anhui, School of Basic Medical Sciences, Anhui Medical University, Hefei, China,; 4 China National Center for Biotechnology Development, Beijing, China; Egerton University, KENYA

## Abstract

**Background:**

Dengue, the fastest-spreading vector-borne disease (VBD), significantly burdens global health systems. This study analyzed the trends in the global burden of dengue from 1990 to 2021, utilizing data from the Global Burden of Diseases, Injuries, and Risk Factors Study 2021 (GBD 2021).

**Methodology/Principal findings:**

We retrieved data from GBD 2021 regarding dengue, including the number of incidences and age-standardized incidence rate (ASIR), the number of deaths and age-standardized death rate (ASDR), disability-adjusted life-years (DALYs) and age-standardized DALYs. The estimated annual percentage change (EAPC) of ASIR, ASDR, and standardized DALYs rate was calculated to quantify trends over time. In addition, the correlations between dengue burden and sea level rise, as well as the Socio-Demographic Index (SDI), were evaluated. In this study, it was observed that from 1990 to 2021, the global incidence of dengue escalated from 26.45 million to 58.96 million cases, accompanied by an increase in related deaths from 14,315 to 29,075, and DALYs rising from 1.25 million to 2.08 million years. These data collectively indicate that the disease burden approximately doubled, with South Asia, Southeast Asia, and tropical Latin America being the most severely affected regions. The disease burden remained substantial in middle and low-middle-SDI regions, whereas high-middle and high SDI regions experienced pronounced growth rates in ASIR, ASDR, and age-standardized DALYs rate. Adolescents and the elderly showed higher incidence, yet children under 5 had the highest DALYs. Correlation analyses revealed an inverted U-shaped relationship between the SDI and both the ASDR and age-standardized DALYs rate, and changes in sea level height strongly correlated with the overall dengue burden.

**Conclusions/Significance:**

The global dengue burden has surged due to climate change, vector transmission, and population mobility. Increased focus and tailored control strategies are essential, particularly in South Asia, Southeast Asia, and Latin America.

## Author summary

In the past three decades, dengue has rapidly proliferated, significantly impacting global health. In order to comprehensively analyze the global epidemiological trends of dengue from 1990 to 2021, we utilized the latest data from the Global Burden of Disease (GBD) 2021 database, incorporating a multifaceted analysis considering variables such as age, sex, geographical region, and socioeconomic development. Our results indicated that the global burden of dengue approximately doubled during the study period, with the global incidence surging from 26.45 million to 58.96 million cases and the number of related deaths climbing from 14,315 to 29,075 between 1990 and 2021. Moreover, disability-adjusted life-years increased from 1.25 million to 2.08 million years. We also analyzed the factors contributing to these varying trends, including sea level rise and the impact of global dengue epidemiology at different stages of the COVID-19 pandemic. Our findings revealed a gradual escalation of the global dengue epidemic against the backdrop of climate warming and increased population mobility. This research provided insights that could assist policymakers in implementing effective control and management strategies for dengue.

## 1. Introduction

In 2019, the World Health Organization (WHO) classified dengue as one of the top ten global public health threats [1]. In recent decades, the global incidence of dengue has surged, establishing it as the fastest-spreading insect vector-borne disease (VBD) [2]. Approximately 90 countries have reported active dengue transmission in 2024 [3]. Brazil is the country most severely affected by dengue, followed by Argentina and Mexico [4]. According to the WHO Global Dengue Surveillance, from January to November 2024, the total number of dengue cases was 13,860,025, with a total of 9990 deaths. The majority of these cases were from the Americas, which reported 12,669,716 cases and 7713 deaths. The second most affected region was Southeast Asia, with a total of 693,596 cases and 2002 deaths [4]. Notably, many dengue cases are asymptomatic or mild, and some cases may have been misdiagnosed as other febrile illnesses, which indicates that the true incidence may exceed reported values.

Dengue primarily affects adolescents and elderly individuals. It is transmitted mainly by *Aedes aegypti* mosquitoes, with *Aedes albopictus* as the secondary vector [5]. The peak biting periods for these mosquitoes typically occur during dawn and dusk [6]. Changes in vector distribution may influence disease transmission, particularly in areas previously unaffected by dengue. Additionally, the increase in temperature, changes in rainfall and humidity associated with the El Niño phenomenon [7,8], healthcare system vulnerabilities, and population movement during the COVID-19 pandemic have influenced transmission. The WHO has documented rare cases of transmission through blood products, organ donations, and blood transfusions [6].

To improve the triage and management of dengue patients, the WHO revised its classification system in 2009, categorizing cases into dengue without warning signs, dengue with warning signs, and severe dengue [9]. Dengue without warning signs manifests as fever, nausea, vomiting, rash, and generalized pain, with a low risk of mortality. Dengue with warning signs includes severe abdominal pain, fluid accumulation, and positional hypotension. Severe dengue is characterized by severe fluid accumulation with respiratory distress and/or shock, severe mucocutaneous bleeding, and severe organ involvement [10]. Experienced medical care for severe dengue can reduce mortality rates from over 20% to less than 1% [11]. Following dengue virus infection, the humoral immune system produces specific neutralizing antibodies and differentiates B cells into memory B cells, establishing long-term immune memory. Simultaneously, T cells in the cellular immune system directly eliminate virus-infected cells. Notably, dengue virus belongs to the family Flaviviridae and includes four distinct serotypes: DENV-1, DENV-2, DENV-3, and DENV-4 [12]. Due to amino acid sequence differences exceeding 30% between serotypes [13], leading to significant antigenic divergence, cross-serotypic protective immunity is not feasible [14]. Infection with one dengue virus serotype confers long-lasting immunity to that serotype, while cross-protection against other serotypes is transient [15]. Additionally, genotypes are typically associated with specific geographic regions, and understanding the prevalent dengue serotypes in different areas is crucial for disease control and vaccination strategies.

Currently, the primary treatment for dengue remains supportive therapy to maintain hydration, with no specific antiviral agents available [3,16]. Vaccination and vector control are effective strategies for reducing transmission. In 2023, the WHO recommended the widespread use of the *Qdenga* vaccine, in high-incidence regions [17]. The vaccine has proven safe and effective against four serotypes. Vector control relies primarily on community and household efforts, such as insecticides, the regular cleaning of water containers, and the use of bed nets [18]. However, the current effectiveness of these methods remains limited [19].

Therefore, this study utilized data from the 2021 Global Burden of Diseases, Injuries, and Risk Factors Study (GBD 2021) to elucidate the dynamic trends of global incidence, mortality, and disability-adjusted life years (DALYs) associated with dengue from 1990 to 2021 [20]. Additionally, Given that GBD 2021 differs from iterative versions by including the COVID-19 pandemic in its update period, we also specifically investigated the impact of COVID-19 on dengue transmission. This study aims to provide policymakers with a more comprehensive perspective on the global distribution patterns, long-term trends, and regional disparities of dengue, thereby assisting them in more effectively planning and allocating healthcare resources.

## 2. Methods

### 2.1. Study design

This study is an observational research that comprehensively analyzes the trends in incidence, mortality, and DALYs of dengue fever from 1990 to 2021 globally. The primary objective of this study is to assess the changes in the global burden of dengue fever and guide the formulation of public health intervention measures. The study data are sourced from the GBD 2021 database, incorporating all data related to dengue fever, including incidence, age-standardized incidence rate (ASIR), mortality, age-standardized death rate (ASDR), and DALYs and age-standardized DALYs rate, as well as data categorized by age, sex, and Socio-Demographic Index (SDI). The analysis methods employ estimated annual percentage change (EAPC) to quantify the temporal trends in ASIR, ASDR, and age-standardized DALYs, while linear regression analysis is used to explore the correlation between sea level height changes and the burden of dengue fever. Additionally, locally weighted scatterplot smoothing is applied to fit trend lines separately for SDI with ASIR, ASDR, and age-standardized DALYs rate. All statistical analyses are conducted using R software version 4.4.1.

### 2.2. Data sources

The GBD 2021, spearheaded by the Institute for Health Metrics and Evaluation at the University of Washington, covers 204 countries and territories, 288 causes of death, 371 diseases and injuries, and 88 risk factors, providing detailed estimates for different age groups and sexes from 1990 to 2021. Dengue burden data from 1990 to 2021 were obtained from the Global Health Data Exchange GBD Results Tool (http://ghdx.healthdata.org/gbd-results-tool). This dataset includes incidence and ASIR, deaths and ASDR, as well as DALYs and age-standardized DALYs rate, stratified by country, region, age, and sex. The 95% uncertainty intervals (UIs) for the estimates are determined as the 2.5th and 97.5th percentile values from 500 simulations [21,22]. The GBD 2021 database utilizes the International Classification of Diseases (ICD, 10th Edition), wherein the ICD codes for non-fatal dengue are A90-A91.0, and the ICD codes for fatal dengue are A90-A91.9. The estimation process for disease burden in the GBD 2021 study and the significant changes compared with those in 2019 have been detailed in previous publications. Among these changes, the most significant is that the GBD 2021 study provides the first estimates of health loss related to the COVID-19 pandemic. The SDI, formulated by GBD researchers, closely correlates with health outcomes and serves as an indicator of development status. (S1–S3 Tables). It is the geometric mean of three 0–1 indicators: the total fertility rate for those under 25, the average education for people aged 15 and older, and the lag distributed income per capita. A value of 0 indicates the highest fertility rate, lowest level of education, and lowest per capita income, whereas a value of 1 indicates the opposite [23]. While, GBD regions, and in turn GBD super-regions, are composed of countries and territories that are geographically close, epidemiologically similar, and share similar distributions of causes of death. To investigate the correlation between dengue burden and sea level height, sea level height data from 1992 to 2021 were downloaded from NOAA (https://www.star.nesdis.noaa.gov/socd/lsa/SeaLevelRise/LSA_SLR_timeseries_global.php). The data were measured via various radar satellite altimeters, including TOPEX/Poseidon (T/P), JaJason-2, and Jason-3.

### 2.3. Data quality

The data in the GBD database exhibit reliability and representativeness: it integrates as much available data as possible from around the world, with data sources including vital registrations, verbal autopsies, police records, scientific literature, household survey data, epidemiological surveillance data, disease registry data, and clinical informatics data [22]. Furthermore, the GBD database is updated every two years to ensure the timeliness of the data. Lastly, the database demonstrates its comprehensiveness by covering multidimensional data on dengue fever, such as incidence, mortality, and DALYs, across various age groups, genders, and geographical regions, facilitating a multifaceted analysis of the disease burden of dengue fever. However, one unavoidable limitation of the GBD database is the potential for errors in the quality and collection of raw data, which necessitates continuous improvements in the data collection system to address these issues [22].

### 2.4. Statistical analysis

The EAPC was used to quantify the trends in the ASIR, ASDR, and DALYs rate from 1990 to 2021. A regression line was fitted to the natural logarithm of the age-standardized indicators, specifically ln(y) = α + βx + ε, where y represents the respective age-standardized indicator and x denotes the calendar year. The formula for calculating the EAPC is 100 × (exp(β) − 1), from which the 95% confidence interval (CI) can also be derived. An upward trend in the age-standardized rate is indicated when both the EAPC value and the lower bound of its 95% CI are greater than zero. Conversely, a downward trend is indicated when both the EAPC value and the upper bound of its 95% CI are less than zero. All other cases indicate a stable trend. Furthermore, linear regression analysis was performed to investigate the correlations between sea level elevation and the incidence, number of deaths and DALYs of dengue. A locally weighted scatterplot smoothing approach was utilized to individually fit trend lines for the SDI against the ASIR, ASDR, and age-standardized DALYs rate. All the statistical analyses were conducted using R 4.4.1.

## 3. Results

### 3.1. Incidence burden of dengue

**Fig 1:**
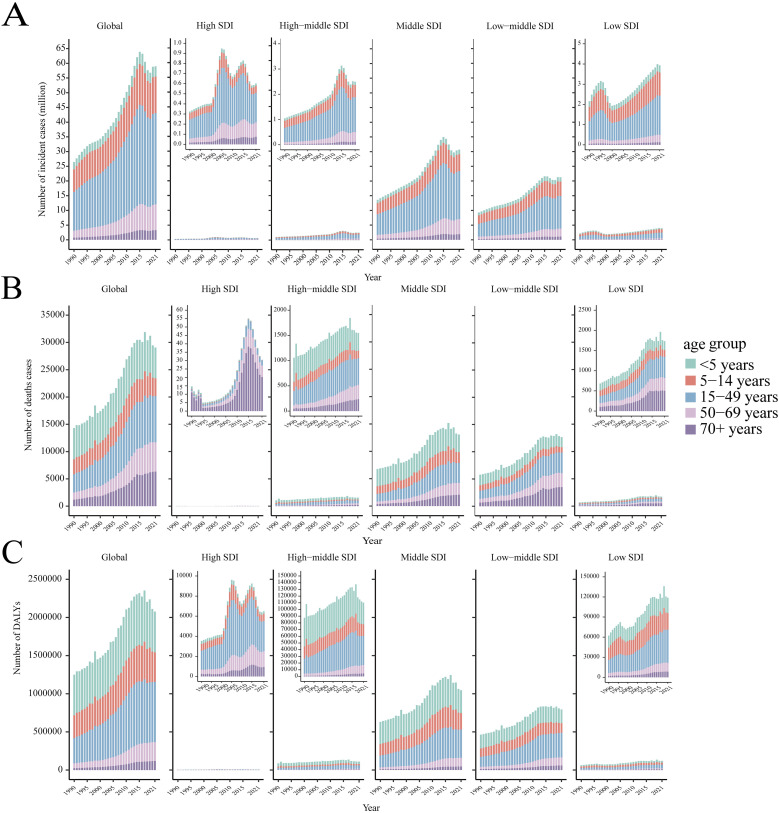
The age distribution of the number of incident cases (A), the number of deaths (B), and the number of DALYs (C) from 1990 to 2021, by global regions and the five SDI regions. SDI, the socio-demographic index. DALYs, the disability-adjusted life-years.

**Fig 2:**
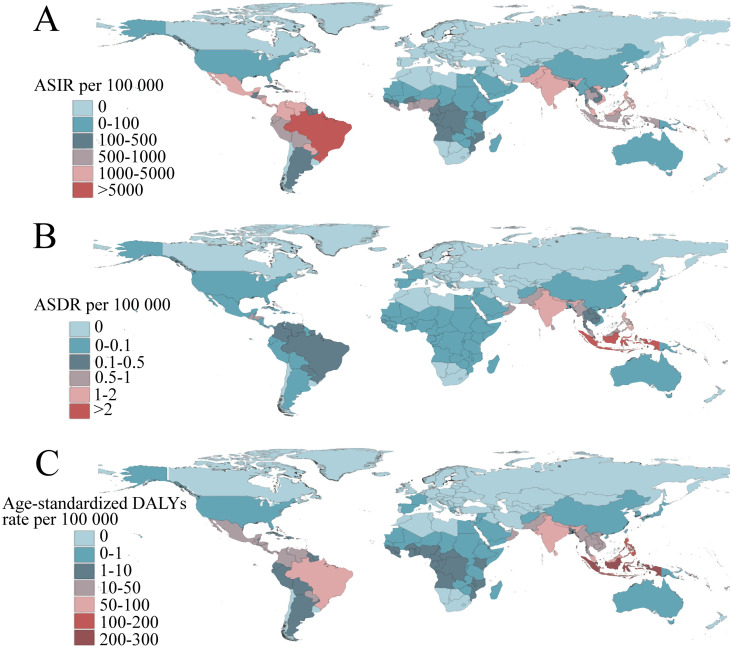
Global age-standardized incidence (A), death (B), and DALYs (C) rate of dengue in 204 countries or territories in 2021. The basemap shapefile is sourced from the Resources and Environmental Science Data Platform (https://www.resdc.cn). DALYs, the disability-adjusted life-years.

**Fig 3:**
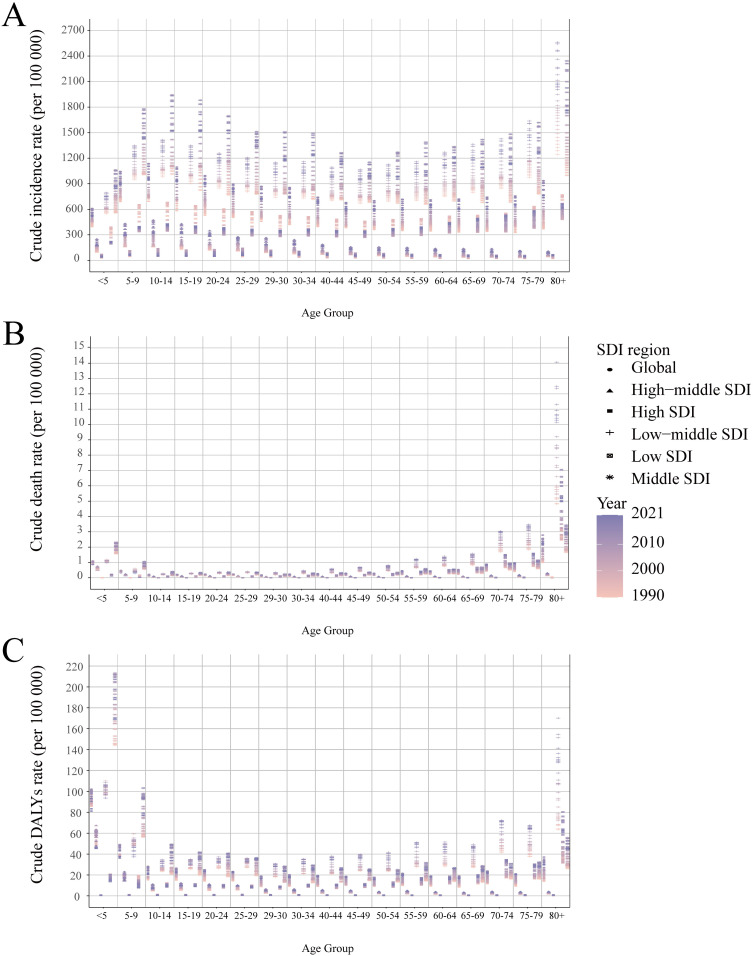
The crude incidence (A), death (B), and DALYs (C) rate by global and the five SDI regions in different age groups from 1990 to 2021. SDI, the socio-demographic index. DALYs, the disability-adjusted life-years.

**Fig 4:**
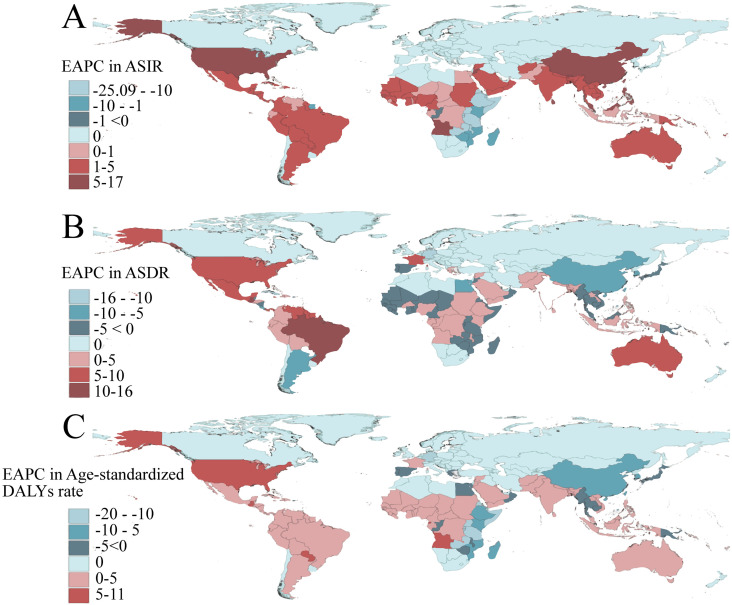
Estimated annual percentage change in age-standardized incidence (A), death (B), and DALYs rate (C)of dengue in 204 countries or territories in 2021. The basemap shapefile is sourced from the Resources and Environmental Science Data Platform (https://www.resdc.cn). DALYs, the disability-adjusted life-years.

**Fig 5:**
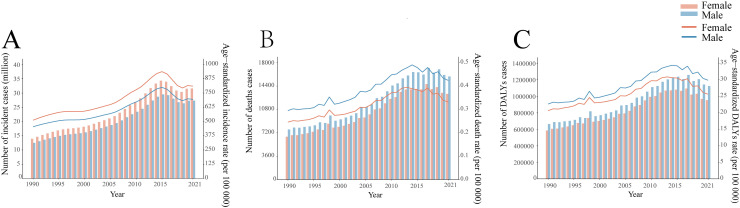
Dengue incident numbers and incidence rate by sex from 1990 to 2021 (A); Dengue death numbers and death rate by sex from 1990 to 2021 (B); Dengue DALYs numbers and DALYs rate by sex from 1990 to 2021 (C). DALYs, the disability-adjusted life-years.

Based on the data from the GBD 2021, dengue was reported in 126 countries and regions worldwide. From 1990 to 2021, the number of dengue cases increased from 26.45 million to 58.96 million (S4 Table). Notably, the peak number of cases, reaching 63.88 million, was observed in 2015 (Fig 1). The ASIR per 100,000 population increased from 481.90 in 1990 to 752.00 in 2021, with an EAPC of 1.83 (95% CI: 1.58, 2.08) (S4 Table). Among the 21 GBD regions, South Asia and tropical Latin America recorded the highest number of cases (Fig 2). Remarkably, high-income North America displayed the fastest growth in ASIR (EAPC: 6.75, 95% CI: 5.30, 8.22), followed by Australia (EAPC: 3.76, 95% CI: 2.93, 4.59) (S4 Table). Within the SDI regions, the highest incidence was seen in the middle SDI and low-middle SDI areas. (Fig 3) However, the ASIR in high-middle SDI regions exhibited the fastest growth (EAPC: 3.38, 95% CI: 2.98, 3.79) (S4 Table). At the national and regional levels, Tonga, Nauru, Equatorial Guinea, Maldives, and Tuvalu were the five countries with the fastest growth in ASIR, with EAPCs of 16.98 (95% CI: 12.71, 21.41), 15.79 (95% CI: 10.31, 21.55), 10.93 (95% CI: 9.18, 12.71), 10.54 (95% CI: 9.00, 12.11), and 8.72 (95% CI: 6.34, 11.16), respectively (Fig 4 and S7 Table). By sex, from 1990 to 2021, the number of cases in females consistently exceeded that in males, with females reporting 27.35 million cases in 2021 compared with 31.62 million cases in males (Fig 5). Additionally, the EAPC for females (1.88, 95% CI: 1.62, 2.13) surpassed that for males (EAPC: 1.76, 95% CI: 1.53, 2.00). In terms of age distribution, the incidence of cases worldwide and across the five SDI regions was primarily concentrated in the 15–49 age group, while the number of cases in the under 5 age group was the lowest. A similar trend was observed in the middle SDI, lower-middle SDI, and low SDI regions (Fig 1), where the incidence rate in the 0–14 years age group gradually increased, whereas the incidence rate in the 15–49 years age group gradually decreased, followed by a resurgence in the incidence rate for individuals aged 50 years and older (Fig 3).

### 3.2. Death burden of dengue

Globally, the number of deaths attributed to dengue increased from 14,315 in 1990 to 29,075 in 2021, peaking in 2017, with 31,897 fatalities (Fig 1 and S5 Table). The ASDR per 100,000 people rose from 268.00 in 1990 to 376.50 in 2021, with an EAPC of 1.70 (95% CI: 1.45, 1.94) (S5 Table). Among the 21 GBD regions, South Asia and Southeast Asia reported the greatest number of deaths (Fig 2), whereas tropical Latin America showed the fastest increase in ASDR (EAPC: 10.79, 95% CI: 9.23, 12.37), followed by high-income North America (EAPC: 8.60, 95% CI: 7.59, 9.63) (S5 Table). In the SDI regions, low SDI and middle SDI areas reported the highest number of deaths (Fig 1), however, the ASDR in high SDI regions exhibited the fastest growth (EAPC: 4.37, 95% CI: 2.66, 6.1) (S5 Table). At the national and regional levels, Paraguay, Taiwan (Province of China), Guatemala, Singapore, and the United States Virgin Islands were the five countries with the fastest-growing ASDR, with corresponding EAPCs of 25.63 (95% CI: 21.78, 29.61), 25.37 (95% CI: 22.12, 28.71), 12.97 (95% CI: 11.07, 14.9), 12.71 (95% CI: 9.68, 15.81), and 11.05 (95% CI: 7.14, 15.1) in that order (Fig 4 and S7 Table). In terms of gender, from 1990 to 2021, the number of male deaths consistently exceeded that of females. In 2021, there were 15,877 male deaths and 13,198 female deaths (Fig 5). Mortality increased more rapidly in males (EAPC: 1.76, 95% CI: 1.53, 2.00) than in females (EAPC: 1.57, 95% CI: 1.31, 1.83), in contrast to the pattern observed in incidence (S5 Table). Concerning age distribution, the 15–49 years age group had the highest number of deaths, while the 5–14 years age group had the fewest deaths. In regions with high SDI, those over 70 years demonstrated significantly greater numbers of deaths than other age groups did (Fig 1). Importantly, mortality progressively increased with age, particularly among individuals aged 35 years and above (Fig 3).

### 3.3. DALYs burden of dengue

Globally, the burden of DALYs attributable to dengue rose from 1.25 million in 1990 to 2.08 million in 2021, peaking at 2.35 million in 2017, mirroring the same trends observed in mortality (Fig 1 and S6 Table). The age-standardized DALYs rate per 100,000 population increased from 21.63 in 1990 to 27.76 in 2021, with an EAPC of 1.33 (95% CI: 1.10, 1.57) (S6 Table). Among the 21 GBD regions, South Asia and Southeast Asia recorded the highest number of DALYs (Fig 2). However, high-income North America (EAPC: 8.00, 95% CI: 7.16, 8.85) and Australia (EAPC: 3.81, 95% CI: 2.97, 4.65) exhibited the fastest growth in DALYs (S6 Table). Within the SDI regions, the burden of DALYs was most pronounced in the middle SDI and low-middle SDI regions (Fig 3). However, the ASIR showed the most rapid increase in high SDI regions (EAPC: 1.96, 95% CI: 1.04, 2.90) (S6 Table). At the country and regional levels, Equatorial Guinea, Guatemala, Nauru, the United States of America, and Angola presented the most rapid increases in ASIR, with EAPCs of 10.52 (95% CI: 8.76, 12.31), 9.96 (95% CI: 8.59, 11.33), 8.77 (95% CI: 6.12, 11.49), 8.01 (95% CI: 7.18, 8.85), and 7.43 (95% CI: 6.89, 7.97), respectively (Fig 4 and S7 Table). In terms of sex, the burden of DALYs for males consistently surpassed that for females from 1990 to 2021, reaching 1,123,515.90 years for males in 2021 compared with 953,008.80 years for females (Fig 5). Additionally, the EAPC for males (1.42, 95% CI: 1.2, 1.65) exceeded that for females (EAPC: 1.22, 95% CI: 0.96, 1.47), aligning with the trends observed in the ASDR (S6 Table). In terms of age distribution, the DALYs were predominantly concentrated in the 15–49 years age group globally and across the five SDI regions, while the >70 years age group bore the least burden. Notably, in high SDI regions, the <5 age group presented the lowest number of DALYs (Fig 1). Importantly, despite the highest incidence and mortality occurring in the 80+ age group, the peak DALYs were found in the <5 age group (Fig 3).

### 3.4. Correlations with dengue burden

**Fig 6:**
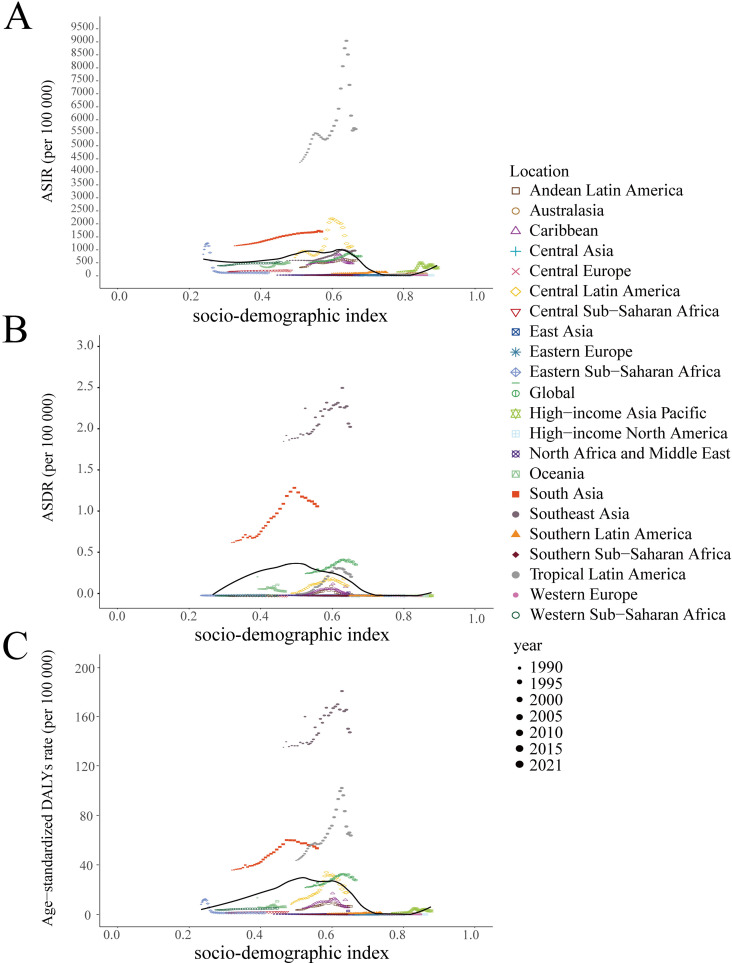
The correlation between ASIR (A), ASDR (B), and age-standardized DALYs rate(C) with the socio-demographic index in global and the 21 GBD regions. Different colors and shapes represent various GBD regions, with the size of the shapes increasing to denote the progression from 1990 to 2021. ASIR, the age-standardized incidence rate. ASDR, the age-standardized death rate. DALYs, the disability-adjusted life-years. GBD, the global burden of disease.

**Fig 7:**
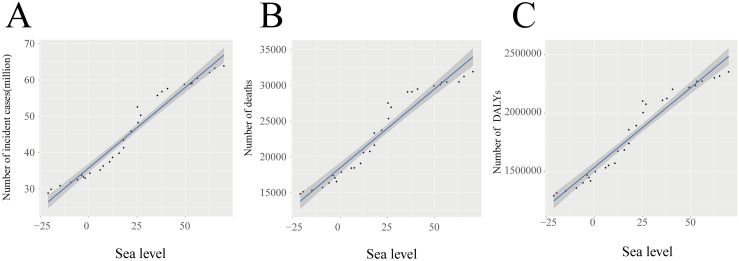
The correlation between the number of incident cases (A), the number of deaths (B), the number of DALYs (C), and sea level. DALYs, the disability-adjusted life-years.

There was a nonlinear relationship between the ASIR and the regional SDI from 1990 to 2021. In regions with an SDI below 0.6, such as western sub-Saharan Africa, South Asia, and Southeast Asia, the ASIR consistently remained high and stable over the past three decades. Conversely, when the SDI exceeds 0.6, the ASIR gradually decreases, but it tends to increase once the SDI reaches 0.8. Additionally, a relatively regular inverted U-shaped relationship existed between the regional SDI and both the ASDR and age-standardized DALYs rate for dengue from 1990 to 2019 (Fig 6). Furthermore, from 1992 to 2021, a strong positive correlations were identified between sea level height and dengue incidence, number of deaths, and DALYs, with R-square values of 0.96 (*P*<0.001), 0.94 (*P*<0.001), and 0.94 (*P*<0.001), respectively (Fig 7).

## 4. Discussion

In this study, we analyzed the global burden of dengue from 1990 to 2021 using the GBD 2021 database. The results indicated that the incidence, number of deaths, and DALYs associated with dengue globally approximately doubled during this period. The regions with the heaviest burden from 1990 to 2021 remained in the tropical and subtropical areas, including South Asia, Southeast Asia, and Tropical Latin America. Therefore, understanding the dynamics of dengue transmission is crucial for decision-makers in effectively allocating health resources and implementing successful intervention strategies.

The spatial distribution of dengue is strongly affected by climate [24], with summer and autumn being peak seasons. Dengue was most prevalent in tropical and subtropical regions such as South Asia, Tropical Latin America, Southeast Asia, Central Latin America, and Western Sub-Saharan Africa. This distribution pattern was due to climatic factors such as temperature, rainfall, humidity, and wind speed, which are crucial for dengue transmission [25,26]. Studies have shown that the optimal survival temperature for *Aedes* mosquitoes is 25–30°C [27], enhancing egg hatching, larval development, and oviposition. Concurrently, dengue virus transmission was most efficient at 20–26°C, increasing mosquito biting and virus replication [28]. Rainfall also impacted dengue transmission, as it increases water storage and vegetation, aiding mosquito oviposition and larval growth. However, there were exceptions to these influences. Extreme rainfall can potentially disrupt the survival conditions of mosquito eggs. Temperatures exceeding 40 degrees Celsius led to the mortality of mosquitoes. Additionally, excessively high vegetation coverage is often correlated with sparse human populations, all of which are detrimental to the transmission of dengue. A Lao study confirmed the nonlinear relationships among temperature, rainfall, vegetation coverage, and dengue transmission [29]. With increasing sea levels, the burden of dengue often increases. *Aedes aegypti* primarily oviposits and develops in freshwater. However, recent studies have identified heritable salt-tolerance genes in *Aedes aegypti*, enabling it to survive and develop in saltwater [30]. Rising sea levels have resulted in increased saltwater intrusion into freshwater layers, leading to the expansion of saltwater bodies. As a result, this expanded mosquito breeding sites and increased vector populations, particularly those affecting South Asia and Southeast Asia, which have extensive coastlines. Additionally, experiments have shown that *Aedes aegypti* developing in saline conditions can vertically transmit the dengue virus (DENV) to their offspring [31]. Therefore, salt-tolerant vectors may serve as reservoirs for DENV, facilitating faster transmission by freshwater vectors during monsoon seasons [32,33].

According to the GBD 2021 data, the regions with the highest incidence, number of deaths, and DALYs burden of dengue were chiefly concentrated in middle SDI countries, followed by lower-middle SDI countries. In middle SDI and lower-middle SDI countries, the heavy burden of dengue was associated with factors such as high population density, poor living conditions, inadequate water supply systems, and unregulated urbanization processes [34]. Particularly in 1995, the increase in dengue cases in low SDI countries was primarily attributed to the growing population, uncontrolled urbanization, and climatic conditions favorable to vector proliferation [35].Previous studies have indicated that the burden of mosquitoes is more severe in low-income cities than in high-income cities [36,37]. Thus, strengthening the management of landfills and abandoned houses in these areas, reducing unnecessary outdoor water storage, increasing the use of bed nets, and increasing the application of insecticides are essential. Conversely, regions with high SDI and high-middle SDI experienced the most rapid growth in ASIR, ASDR, and age-standardized DALYs rate. Despite possessing robust healthcare resources and public health infrastructure, high SDI countries experienced a significant increase in dengue cases from 2010 to 2015, primarily attributable to the flourishing of international exchanges. Specifically, global trade, particularly the transportation of used car tires, inadvertently facilitated the spread of vector-borne virus carriers to new regions [38]. Additionally, the movement of international travelers played a critical role. For instance, Queensland, Australia, which is not an endemic region for dengue, witnessed local transmission after infected returning travelers introduced the virus, which was subsequently transmitted by local *Aedes* mosquitoes. Between 2010 and 2015, Queensland recorded a total of 1,773 dengue cases, of which 632 were locally acquired and 1,141 were imported. This underscores the significant role of international travel in disease transmission [39].

In epidemiological investigations of dengue, a meaningful relationship existed between age and disease burden, the highest incidence rates of dengue occurred in both 5 to 14-year-olds and those aged 80 years and above, and the incidence of dengue progressively increases after the age of 60 [40]. The study revealed that children under the age of 14 years and elderly individuals over 50 years had higher mortality and hospitalization rates, which may be related to the development of herd immunity in the population following primary infection. This phenomenon led to a temporary reduction in case numbers, and as the immune population aged and the epidemic persisted, dengue cases periodically reinfected younger generations. After the age of 60, the risk of severe dengue from secondary infections also increased with age. The study findings indicate that the number of dengue fever cases is consistently higher among females compared to males. In contrast, the number of deaths and DALYs are higher in males. This discrepancy may be attributed to differences in health-seeking behaviors, where males are more likely to delay seeking medical attention due to under-recognition or neglect of symptoms. Consequently, there is an imperative for enhanced surveillance and prompt diagnosis in male patients to mitigate adverse outcomes associated with dengue infection [41].

Notably, DALYs were highest among those under 5 years of age, indicating that dengue infection during early childhood had the most significant impact on health. The high risk of severe dengue in infants is attributed to antibody-dependent mechanisms [42]. Although the antibodies initially acquired from the mother provided protection, this protective mechanism may have facilitated dengue infection as the antibody levels declined [43]. A study in Thailand showed that from 1981 to 2017, the average age of dengue incidence increased from 8.1 years to 24.3 years [44], with an increasing distribution toward older individuals. Older adults, due to comorbidities such as diabetes, hypertension, and heart disease, often faced more severe consequences [45]. This shift in age distribution provides crucial insights for optimizing epidemiological surveillance, prevention, and management strategies for dengue, particularly by focusing on the health needs of high-risk populations [46].

Compared with historical data, the variations in dengue statistics observed after 2019 are, to a certain degree, impacted by the COVID-19 pandemic’s influence. Overall, from 2019 to 2020, the incidence rate of dengue showed an increasing trend. However, the impact of the COVID-19 pandemic on dengue varied across different stages. At the beginning of 2020, as countries implemented lockdown measures due to the pandemic, international flights were significantly reduced, leading to a marked decrease in imported cases. Countries like Australia, China, Malaysia, and Italy [47–51], which were predominantly affected by imported cases, experienced a substantial decrease in dengue incidence in early 2020. In addition, other factors during the lockdown period may have contributed to the decrease in dengue cases. For example, some patients chose not to seek medical attention after falling ill, the early clinical manifestations of dengue were similar to those of COVID-19, and potential cross-reactivity in serological testing, all of which could have led to misdiagnoses. However, the study found that by late 2020, the long period of staying at home raised the likelihood of residents being bitten by mosquitoes, resulting in an increase in cases occurring in residential areas and surrounding parks. This led to a dramatic rebound in the number of cases in these countries. In summary, under the influence of the COVID-19 pandemic, the incidence of dengue has increased. In 2020, Singapore experienced the most severe dengue epidemic in seven years [52]. Simultaneously, Brazil also witnessed an increase in dengue cases during the COVID-19 pandemic. Furthermore, studies have indicated that despite the greater influence of climate on the dengue burden in Indonesia, Thailand and Vietnam, along with the relatively minor association with imported cases, there is a significant positive correlation between the dengue epidemic and the COVID-19 pandemic [53].

In this study, we comprehensively analyzed dengue using GBD 2021 data. However, there were several limitations to this research. First, the GBD database has inherent limitations related to the quality and collection processes of the raw data concerning dengue [53]. For example, deficiencies in data collection methods and potential underreporting resulted in a lower reported incidence, which can be continually improved by strengthening data-collection systems. The early symptoms of dengue are similar to those of COVID-19 [54], malaria, and typhoid fever, potentially leading to misdiagnosis. Concurrently, for countries lacking systematic national surveillance or population-based dengue studies, the GBD estimates rely on the out-of-sample predictive validity of the modeling process. This approach cannot fully replace high-quality primary data; it merely ensures that these populations are not excluded from important benchmarking exercises for burden estimation [22]. Second, this study lacked an in-depth analytical exploration of the four dengue serotypes. Infection with one serotype provides some immune protection, but subsequent infections with different serotypes often result in more severe symptoms [9]. Thus, a serotype-focused analysis would better support local epidemic prevention and treatment strategies [55–57]. Thirdly, this study found that the incidence of dengue fever is higher among females compared to males; however, in terms of mortality and DALYs, males exhibit a higher burden. The specific mechanisms underlying the observed gender differences in dengue disease burden remain unclear and urgently require further investigation to uncover the underlying causes. Finally, This study categorized age groups based on GBD predefined age categories, which may not sufficiently capture the variations in dengue burden across different age groups and may lack more granular age classifications that could provide critical data for vaccine strategies.

## 5. Conclusion

From 1990 to 2021, the global burden of dengue significantly increased due to the combined effects of multiple factors, including climate change, vector transmission, and population mobility, with particularly pronounced impacts observed in South Asia, Southeast Asia, and Latin America. Countries with middle and low-middle SDI bore a heavier disease burden, primarily because of their high population density, poor living conditions, and unreasonable urbanization processes in these regions. While the COVID-19 pandemic had a short-term impact on the spread of dengue, overall, there was no significant reduction in dengue cases during the pandemic, and a rebound was observed at the end of 2020.

In conclusion, this study highlights the significant increase in incidence, mortality, and DALYs rate over the past three decades. The findings emphasize the urgent need for enhanced surveillance, targeted prevention strategies, and improved resource allocation. By identifying regions and populations most affected by changes in disease burden, this study highlights the need for focused interventions. For example, in lower-middle SDI regions, efforts should focus on improving sanitation and controlling vector breeding sites, while in higher SDI regions, stricter quarantine measures and monitoring of international travelers should be implemented. Additionally, considering gender and age disparities, specific health education and monitoring programs should be designed for vulnerable groups, such as children, elderly, and males, to enhance treatment efficacy and overall public health protection. In summary, this research contributes to a deeper understanding of dengue epidemiology and supports the development of more effective and sustainable control strategies.

## Supporting information

S1 Table The different categories of the five SDI ranges from 0 to 1. (DOCX)

S2 Table 2021 SDI Index Value and SDI Quintile for 204 Countries or Regions. (DOCX)

S3 Table Socio-demographic Index values for all estimated GBD 2021 locations, 1990-2021. (DOCX)

S4 Table Incident cases and ASIR of dengue in 1990 and 2021, and the EAPC of ASIR from 1990 to 2021. (DOCX)

S5 Table Deaths Cases and ASDR of dengue in 1990 and 2021, and the EAPC of ASDR from 1990 to 2021. (DOCX)

S6 Table DALYs and age-standardized DALYs rate of dengue in 1990 and 2021, and the EAPC of age-standardized DALYs rate from 1990 to 2021. (DOCX)

S7 Table Top 10 Countries/Regions with the Highest EAPC in ASIR, ASDR, and Age-Standardized DALYs Rate from 1990 to 2021. (DOCX)
