# Supplementary material for: Assessing the global dengue burden: Incidence, mortality, and disability trends over three decades
Source: PLoS Negl Trop Dis. 2025 Mar 12;19(3):e0012932. doi: 10.1371/journal.pntd.0012932 (PMC11925280; doi:10.1371/journal.pntd.0012932)
Supplement: S1 Table — (DOCX) [file pntd.0012932.s001.docx]

| **location_name** | **lower_bound** | **upper_bound** |
| --- | --- | --- |
| Low SDI | 0 | 0.46581580319161997 |
| Low-middle SDI | 0.46581580319161997 | 0.6188294452454329 |
| Middle SDI | 0.6188294452454329 | 0.7119746219361235 |
| High-middle SDI | 0.7119746219361235 | 0.8102959891918925 |
| High SDI | 0.8102959891918925 | 1 |

**S1 Table. The different categories of the five SDI ranges from 0 to 1.**

**SDI: the socio-demographic index.**
