# Supplementary material for: Assessing the global dengue burden: Incidence, mortality, and disability trends over three decades
Source: PLoS Negl Trop Dis. 2025 Mar 12;19(3):e0012932. doi: 10.1371/journal.pntd.0012932 (PMC11925280; doi:10.1371/journal.pntd.0012932)
Supplement: S2 Table — (DOCX) [file pntd.0012932.s002.docx]

**S2 Table. 2021 SDI Index Value and SDI Quintile for 204 Countries or Regions.**

| **Location Name** | **2021 SDI Index Value** | **SDI Quintile** |
| --- | --- | --- |
| Taiwan (Province of China) | 0.875139514 | High SDI |
| Czechia | 0.828510085 | High SDI |
| Slovenia | 0.842633141 | High SDI |
| Estonia | 0.845787294 | High SDI |
| Latvia | 0.830715451 | High SDI |
| Lithuania | 0.857613278 | High SDI |
| Republic of Korea | 0.887195638 | High SDI |
| Singapore | 0.856235308 | High SDI |
| Australia | 0.844269408 | High SDI |
| Andorra | 0.869895393 | High SDI |
| Austria | 0.854558286 | High SDI |
| Belgium | 0.853674059 | High SDI |
| Cyprus | 0.835648571 | High SDI |
| Denmark | 0.897314038 | High SDI |
| Finland | 0.860244219 | High SDI |
| France | 0.837816091 | High SDI |
| Germany | 0.903515704 | High SDI |
| Iceland | 0.874628639 | High SDI |
| Ireland | 0.873989853 | High SDI |
| Luxembourg | 0.884636327 | High SDI |
| Netherlands | 0.888375951 | High SDI |
| Switzerland | 0.933531726 | High SDI |
| Canada | 0.873181934 | High SDI |
| Kuwait | 0.846802486 | High SDI |
| Qatar | 0.846704498 | High SDI |
| Saudi Arabia | 0.814515567 | High SDI |
| United Arab Emirates | 0.849740335 | High SDI |
| Bermuda | 0.821319794 | High SDI |
| Greenland | 0.835640003 | High SDI |
| Hong Kong Special Administrative Region of China | 0.850450848 | High SDI |
| Macao Special Administrative Region of China | 0.874759481 | High SDI |
| Monaco | 0.909519124 | High SDI |
| Puerto Rico | 0.824543903 | High SDI |
| San Marino | 0.887883596 | High SDI |
| United States Virgin Islands | 0.822988043 | High SDI |
| Northern Ireland | 0.841529955 | High SDI |
| Scotland | 0.853887319 | High SDI |
| Beijing | 0.845876468 | High SDI |
| Shanghai | 0.828661817 | High SDI |
| Tianjin | 0.833804693 | High SDI |
| Alabama | 0.826417909 | High SDI |
| Alaska | 0.857125567 | High SDI |
| Arizona | 0.847683473 | High SDI |
| Arkansas | 0.816754461 | High SDI |
| California | 0.871090459 | High SDI |
| Colorado | 0.875986347 | High SDI |
| Connecticut | 0.901972117 | High SDI |
| Delaware | 0.866079256 | High SDI |
| District of Columbia | 0.907426863 | High SDI |
| Florida | 0.861825164 | High SDI |
| Georgia | 0.847268118 | High SDI |
| Hawaii | 0.87084045 | High SDI |
| Idaho | 0.836495322 | High SDI |
| Illinois | 0.880611434 | High SDI |
| Indiana | 0.844050669 | High SDI |
| Iowa | 0.864342086 | High SDI |
| Kansas | 0.858890931 | High SDI |
| Kentucky | 0.821720983 | High SDI |
| Louisiana | 0.826669718 | High SDI |
| Maine | 0.866792716 | High SDI |
| Maryland | 0.891055635 | High SDI |
| Massachusetts | 0.90725037 | High SDI |
| Michigan | 0.864940748 | High SDI |
| Minnesota | 0.887884435 | High SDI |
| Mississippi | 0.811867151 | High SDI |
| Missouri | 0.849044295 | High SDI |
| Montana | 0.859517184 | High SDI |
| Nebraska | 0.865629234 | High SDI |
| Nevada | 0.847864111 | High SDI |
| New Hampshire | 0.898526447 | High SDI |
| New Jersey | 0.891850577 | High SDI |
| New Mexico | 0.832846305 | High SDI |
| New York | 0.88592619 | High SDI |
| North Carolina | 0.846173734 | High SDI |
| North Dakota | 0.876134627 | High SDI |
| Ohio | 0.851227042 | High SDI |
| Oklahoma | 0.82814491 | High SDI |
| Oregon | 0.870189511 | High SDI |
| Pennsylvania | 0.873950359 | High SDI |
| Rhode Island | 0.884283653 | High SDI |
| South Carolina | 0.838586487 | High SDI |
| South Dakota | 0.856263782 | High SDI |
| Tennessee | 0.831968835 | High SDI |
| Texas | 0.836777383 | High SDI |
| Utah | 0.854829295 | High SDI |
| Vermont | 0.89152237 | High SDI |
| Virginia | 0.881907315 | High SDI |
| Washington | 0.878013634 | High SDI |
| West Virginia | 0.82033351 | High SDI |
| Wisconsin | 0.873095963 | High SDI |
| Wyoming | 0.863142903 | High SDI |
| Wales | 0.833274667 | High SDI |
| Oslo | 0.947286104 | High SDI |
| Rogaland | 0.916764681 | High SDI |
| Møre og Romsdal | 0.908149197 | High SDI |
| Nordland | 0.898213885 | High SDI |
| Sweden except Stockholm | 0.875759008 | High SDI |
| Stockholm | 0.916765403 | High SDI |
| Hokkaidō | 0.844008657 | High SDI |
| Aomori | 0.828693902 | High SDI |
| Iwate | 0.835652812 | High SDI |
| Miyagi | 0.860058983 | High SDI |
| Akita | 0.832380775 | High SDI |
| Yamagata | 0.838535343 | High SDI |
| Fukushima | 0.841218485 | High SDI |
| Ibaraki | 0.860523394 | High SDI |
| Tochigi | 0.861256293 | High SDI |
| Gunma | 0.861770439 | High SDI |
| Saitama | 0.85655373 | High SDI |
| Chiba | 0.861645419 | High SDI |
| Tōkyō | 0.929043198 | High SDI |
| Kanagawa | 0.882743596 | High SDI |
| Niigata | 0.845233683 | High SDI |
| Toyama | 0.865453766 | High SDI |
| Ishikawa | 0.860214615 | High SDI |
| Fukui | 0.856579697 | High SDI |
| Yamanashi | 0.858173518 | High SDI |
| Nagano | 0.858966672 | High SDI |
| Gifu | 0.853447894 | High SDI |
| Shizuoka | 0.865720709 | High SDI |
| Aichi | 0.883329296 | High SDI |
| Mie | 0.860795402 | High SDI |
| Shiga | 0.874377981 | High SDI |
| Kyōto | 0.876288854 | High SDI |
| Ōsaka | 0.876433199 | High SDI |
| Hyōgo | 0.868490782 | High SDI |
| Nara | 0.851417382 | High SDI |
| Wakayama | 0.847330156 | High SDI |
| Tottori | 0.836091465 | High SDI |
| Shimane | 0.838873648 | High SDI |
| Okayama | 0.862350161 | High SDI |
| Hiroshima | 0.870170469 | High SDI |
| Yamaguchi | 0.856635725 | High SDI |
| Tokushima | 0.85729815 | High SDI |
| Kagawa | 0.857948449 | High SDI |
| Ehime | 0.843779842 | High SDI |
| Kōchi | 0.835890247 | High SDI |
| Fukuoka | 0.858415047 | High SDI |
| Saga | 0.835872528 | High SDI |
| Nagasaki | 0.829106338 | High SDI |
| Kumamoto | 0.834728198 | High SDI |
| Ōita | 0.848715974 | High SDI |
| Miyazaki | 0.826768586 | High SDI |
| Kagoshima | 0.832472168 | High SDI |
| Okinawa | 0.821640127 | High SDI |
| Darlington | 0.835427723 | High SDI |
| Northumberland | 0.822333819 | High SDI |
| Stockton-on-Tees | 0.829517602 | High SDI |
| Newcastle upon Tyne | 0.871588115 | High SDI |
| North Tyneside | 0.835247504 | High SDI |
| Redcar and Cleveland | 0.796360243 | High SDI |
| County Durham | 0.810754624 | High SDI |
| Gateshead | 0.828579746 | High SDI |
| Middlesbrough | 0.798976656 | High SDI |
| South Tyneside | 0.799489743 | High SDI |
| Sunderland | 0.817933435 | High SDI |
| Hartlepool | 0.797297174 | High SDI |
| Cheshire East | 0.884278805 | High SDI |
| Stockport | 0.861014705 | High SDI |
| Trafford | 0.896679044 | High SDI |
| Cheshire West and Chester | 0.870242062 | High SDI |
| Sefton | 0.825438177 | High SDI |
| Lancashire | 0.839626361 | High SDI |
| Cumbria | 0.842325739 | High SDI |
| Bolton | 0.812595954 | High SDI |
| Wirral | 0.818111684 | High SDI |
| Bury | 0.828816843 | High SDI |
| St Helens | 0.810914124 | High SDI |
| Warrington | 0.878129342 | High SDI |
| Oldham | 0.796704511 | High SDI |
| Rochdale | 0.800322186 | High SDI |
| Wigan | 0.80548334 | High SDI |
| Halton | 0.835687566 | High SDI |
| Liverpool | 0.847483043 | High SDI |
| Tameside | 0.798557943 | High SDI |
| Salford | 0.837393179 | High SDI |
| Blackburn with Darwen | 0.810134059 | High SDI |
| Knowsley | 0.811985259 | High SDI |
| Blackpool | 0.788904606 | High SDI |
| Manchester | 0.88057256 | High SDI |
| North Yorkshire | 0.855520996 | High SDI |
| East Riding of Yorkshire | 0.835193656 | High SDI |
| York | 0.888110353 | High SDI |
| North East Lincolnshire | 0.803563778 | High SDI |
| Calderdale | 0.836996359 | High SDI |
| North Lincolnshire | 0.824582845 | High SDI |
| Bradford | 0.814923693 | High SDI |
| Kirklees | 0.823525465 | High SDI |
| Leeds | 0.86787974 | High SDI |
| Sheffield | 0.854247178 | High SDI |
| Wakefield | 0.804506533 | High SDI |
| Rotherham | 0.803331209 | High SDI |
| Doncaster | 0.793628472 | High SDI |
| Kingston upon Hull, City of | 0.797611011 | High SDI |
| Barnsley | 0.788111758 | High SDI |
| Northamptonshire | 0.839033237 | High SDI |
| Leicestershire | 0.851100665 | High SDI |
| Lincolnshire | 0.820522815 | High SDI |
| Rutland | 0.852195785 | High SDI |
| Derby | 0.844076935 | High SDI |
| Derbyshire | 0.823174583 | High SDI |
| Nottinghamshire | 0.822432787 | High SDI |
| Nottingham | 0.858455251 | High SDI |
| Leicester | 0.828051202 | High SDI |
| Warwickshire | 0.865693767 | High SDI |
| Herefordshire, County of | 0.846194561 | High SDI |
| Solihull | 0.871566638 | High SDI |
| Shropshire | 0.842380445 | High SDI |
| Worcestershire | 0.842681308 | High SDI |
| Staffordshire | 0.828181727 | High SDI |
| Dudley | 0.802585915 | High SDI |
| Coventry | 0.847335743 | High SDI |
| Telford and Wrekin | 0.826093832 | High SDI |
| Stoke-on-Trent | 0.796441727 | High SDI |
| Walsall | 0.790654736 | High SDI |
| Wolverhampton | 0.810887602 | High SDI |
| Birmingham | 0.836949232 | High SDI |
| Sandwell | 0.793668776 | High SDI |
| Bedford | 0.856962284 | High SDI |
| Central Bedfordshire | 0.851318833 | High SDI |
| Suffolk | 0.840388992 | High SDI |
| Hertfordshire | 0.886963263 | High SDI |
| Essex | 0.844953071 | High SDI |
| Cambridgeshire | 0.887630336 | High SDI |
| Thurrock | 0.818629818 | High SDI |
| Norfolk | 0.836988602 | High SDI |
| Southend-on-Sea | 0.825018651 | High SDI |
| Peterborough | 0.837008458 | High SDI |
| Luton | 0.838003231 | High SDI |
| Richmond upon Thames | 0.932021729 | High SDI |
| Kensington and Chelsea | 0.946366051 | High SDI |
| Barnet | 0.885110546 | High SDI |
| Westminster | 0.93701032 | High SDI |
| Bromley | 0.869193749 | High SDI |
| Bexley | 0.844097911 | High SDI |
| Redbridge | 0.849278219 | High SDI |
| Merton | 0.887252238 | High SDI |
| Brent | 0.858683624 | High SDI |
| Hillingdon | 0.892536477 | High SDI |
| Havering | 0.834176853 | High SDI |
| Kingston upon Thames | 0.908394132 | High SDI |
| Sutton | 0.857530229 | High SDI |
| Harrow | 0.858244679 | High SDI |
| Enfield | 0.845516817 | High SDI |
| Croydon | 0.851439949 | High SDI |
| Hammersmith and Fulham | 0.934892187 | High SDI |
| Ealing | 0.882007048 | High SDI |
| Greenwich | 0.845443655 | High SDI |
| Wandsworth | 0.924121015 | High SDI |
| Waltham Forest | 0.840038834 | High SDI |
| Camden | 0.936076172 | High SDI |
| Lambeth | 0.916015975 | High SDI |
| Lewisham | 0.85672931 | High SDI |
| Hounslow | 0.896360928 | High SDI |
| Southwark | 0.919165412 | High SDI |
| Newham | 0.840477768 | High SDI |
| Barking and Dagenham | 0.80642649 | High SDI |
| Haringey | 0.8714663 | High SDI |
| Hackney | 0.891329222 | High SDI |
| Islington | 0.924302624 | High SDI |
| Tower Hamlets | 0.903700654 | High SDI |
| Wokingham | 0.910821902 | High SDI |
| Buckinghamshire | 0.888192339 | High SDI |
| Surrey | 0.904483995 | High SDI |
| Windsor and Maidenhead | 0.915543426 | High SDI |
| West Berkshire | 0.897124589 | High SDI |
| Hampshire | 0.87199888 | High SDI |
| Bracknell Forest | 0.890716234 | High SDI |
| West Sussex | 0.863885895 | High SDI |
| Oxfordshire | 0.899135231 | High SDI |
| Reading | 0.90527187 | High SDI |
| Kent | 0.844500542 | High SDI |
| Brighton and Hove | 0.897901251 | High SDI |
| Medway | 0.819461594 | High SDI |
| East Sussex | 0.83898657 | High SDI |
| Portsmouth | 0.864778156 | High SDI |
| Isle of Wight | 0.826023587 | High SDI |
| Milton Keynes | 0.886756129 | High SDI |
| Southampton | 0.860211008 | High SDI |
| Slough | 0.877374496 | High SDI |
| South Gloucestershire | 0.88411864 | High SDI |
| Dorset | 0.8514167 | High SDI |
| Wiltshire | 0.859020608 | High SDI |
| North Somerset | 0.85864883 | High SDI |
| Devon | 0.854622345 | High SDI |
| Poole | 0.862990852 | High SDI |
| Bath and North East Somerset | 0.895075988 | High SDI |
| Gloucestershire | 0.870918382 | High SDI |
| Somerset | 0.842820976 | High SDI |
| Swindon | 0.86670182 | High SDI |
| Torbay | 0.812459589 | High SDI |
| Bristol, City of | 0.896565514 | High SDI |
| Bournemouth | 0.870149525 | High SDI |
| Cornwall | 0.839219489 | High SDI |
| Plymouth | 0.84217698 | High SDI |
| New Zealand Maori population | 0.770791784 | High SDI |
| New Zealand non-Maori population | 0.870812225 | High SDI |
| Trøndelag | 0.916773946 | High SDI |
| Dolnośląskie | 0.817783922 | High SDI |
| Kujawsko-Pomorskie | 0.790185071 | High SDI |
| Łódzkie | 0.805693677 | High SDI |
| Lubelskie | 0.789673817 | High SDI |
| Lubuskie | 0.791264292 | High SDI |
| Małopolskie | 0.812434112 | High SDI |
| Mazowieckie | 0.849859302 | High SDI |
| Opolskie | 0.794191353 | High SDI |
| Podkarpackie | 0.792282338 | High SDI |
| Podlaskie | 0.786166754 | High SDI |
| Pomorskie | 0.804282452 | High SDI |
| Śląskie | 0.81907325 | High SDI |
| Świętokrzyskie | 0.796464119 | High SDI |
| Warmińsko-Mazurskie | 0.777449817 | High SDI |
| Wielkopolskie | 0.813257407 | High SDI |
| Zachodniopomorskie | 0.792789122 | High SDI |
| Vestland | 0.917642172 | High SDI |
| Agder | 0.907093137 | High SDI |
| Vestfold og Telemark | 0.907351769 | High SDI |
| Innlandet | 0.899977235 | High SDI |
| Viken | 0.914602128 | High SDI |
| Troms og Finnmark | 0.904453583 | High SDI |
| Malaysia | 0.742552841 | High-middle SDI |
| Georgia | 0.733123642 | High-middle SDI |
| Kazakhstan | 0.718331647 | High-middle SDI |
| Bosnia and Herzegovina | 0.72296408 | High-middle SDI |
| Bulgaria | 0.764641037 | High-middle SDI |
| Croatia | 0.799069214 | High-middle SDI |
| Hungary | 0.791024669 | High-middle SDI |
| North Macedonia | 0.750954677 | High-middle SDI |
| Montenegro | 0.796532951 | High-middle SDI |
| Romania | 0.766321392 | High-middle SDI |
| Serbia | 0.79221264 | High-middle SDI |
| Slovakia | 0.808329132 | High-middle SDI |
| Belarus | 0.784114127 | High-middle SDI |
| Republic of Moldova | 0.732393345 | High-middle SDI |
| Brunei Darussalam | 0.810288851 | High-middle SDI |
| Greece | 0.791882294 | High-middle SDI |
| Israel | 0.809091066 | High-middle SDI |
| Malta | 0.801853922 | High-middle SDI |
| Portugal | 0.745394909 | High-middle SDI |
| Spain | 0.76948336 | High-middle SDI |
| Argentina | 0.733528396 | High-middle SDI |
| Chile | 0.770149297 | High-middle SDI |
| Uruguay | 0.721713499 | High-middle SDI |
| Antigua and Barbuda | 0.749849952 | High-middle SDI |
| Bahamas | 0.805143711 | High-middle SDI |
| Barbados | 0.74706542 | High-middle SDI |
| Dominica | 0.747381853 | High-middle SDI |
| Trinidad and Tobago | 0.769401094 | High-middle SDI |
| Bahrain | 0.752218099 | High-middle SDI |
| Jordan | 0.725420238 | High-middle SDI |
| Lebanon | 0.741226017 | High-middle SDI |
| Libya | 0.73508433 | High-middle SDI |
| Oman | 0.773801229 | High-middle SDI |
| Turkey | 0.713246106 | High-middle SDI |
| Mauritius | 0.717977109 | High-middle SDI |
| Seychelles | 0.727579445 | High-middle SDI |
| American Samoa | 0.726267628 | High-middle SDI |
| Cook Islands | 0.778251758 | High-middle SDI |
| Guam | 0.80216771 | High-middle SDI |
| Niue | 0.72621855 | High-middle SDI |
| Northern Mariana Islands | 0.777504838 | High-middle SDI |
| Palau | 0.754590186 | High-middle SDI |
| Saint Kitts and Nevis | 0.756332641 | High-middle SDI |
| Chongqing | 0.713248108 | High-middle SDI |
| Fujian | 0.715426566 | High-middle SDI |
| Guangdong | 0.766590014 | High-middle SDI |
| Heilongjiang | 0.712062797 | High-middle SDI |
| Inner Mongolia | 0.731684517 | High-middle SDI |
| Jiangsu | 0.755114988 | High-middle SDI |
| Jilin | 0.735628033 | High-middle SDI |
| Liaoning | 0.744555054 | High-middle SDI |
| Shaanxi | 0.724147628 | High-middle SDI |
| Shandong | 0.725623497 | High-middle SDI |
| Zhejiang | 0.742041363 | High-middle SDI |
| Riau | 0.724215125 | High-middle SDI |
| Riau Islands | 0.749803315 | High-middle SDI |
| North Kalimantan | 0.754016402 | High-middle SDI |
| Jakarta | 0.801237549 | High-middle SDI |
| East Kalimantan | 0.761652368 | High-middle SDI |
| Distrito Federal | 0.776152007 | High-middle SDI |
| Piemonte | 0.806813454 | High-middle SDI |
| Valle d'Aosta | 0.812706016 | High-middle SDI |
| Liguria | 0.821521126 | High-middle SDI |
| Lombardia | 0.829091495 | High-middle SDI |
| Provincia autonoma di Bolzano | 0.838907386 | High-middle SDI |
| Provincia autonoma di Trento | 0.829531919 | High-middle SDI |
| Veneto | 0.808196606 | High-middle SDI |
| Friuli-Venezia Giulia | 0.819858092 | High-middle SDI |
| Emilia-Romagna | 0.829695157 | High-middle SDI |
| Toscana | 0.811485035 | High-middle SDI |
| Umbria | 0.799092255 | High-middle SDI |
| Marche | 0.803749028 | High-middle SDI |
| Lazio | 0.827195546 | High-middle SDI |
| Abruzzo | 0.816496827 | High-middle SDI |
| Molise | 0.787791453 | High-middle SDI |
| Campania | 0.766832076 | High-middle SDI |
| Puglia | 0.763946911 | High-middle SDI |
| Basilicata | 0.783798467 | High-middle SDI |
| Calabria | 0.775304844 | High-middle SDI |
| Sicilia | 0.76255912 | High-middle SDI |
| Sardegna | 0.772644153 | High-middle SDI |
| Delhi, Urban | 0.730151189 | High-middle SDI |
| Goa, Urban | 0.741223296 | High-middle SDI |
| Himachal Pradesh, Urban | 0.748161993 | High-middle SDI |
| Uttarakhand, Urban | 0.746776963 | High-middle SDI |
| Belgorod oblast | 0.803695074 | High-middle SDI |
| Bryansk oblast | 0.792329315 | High-middle SDI |
| Vladimir oblast | 0.788797618 | High-middle SDI |
| Voronezh oblast | 0.810406591 | High-middle SDI |
| Ivanovo oblast | 0.789631875 | High-middle SDI |
| Kaluga oblast | 0.800527701 | High-middle SDI |
| Kostroma oblast | 0.787756437 | High-middle SDI |
| Kursk oblast | 0.793649987 | High-middle SDI |
| Lipetzk oblast | 0.802916176 | High-middle SDI |
| Moscow oblast | 0.828120859 | High-middle SDI |
| Oryol oblast | 0.79451294 | High-middle SDI |
| Ryazan oblast | 0.793693116 | High-middle SDI |
| Smolensk oblast | 0.800950484 | High-middle SDI |
| Tambov oblast | 0.790982828 | High-middle SDI |
| Tver oblast | 0.799102213 | High-middle SDI |
| Tula oblast | 0.797660828 | High-middle SDI |
| Yaroslavl oblast | 0.805364359 | High-middle SDI |
| Moscow City | 0.887772609 | High-middle SDI |
| Republic of Karelia | 0.81238914 | High-middle SDI |
| Komi Republic | 0.790079817 | High-middle SDI |
| Nenets autonomous district | 0.800997369 | High-middle SDI |
| Arkhangelsk oblast without Nenets autonomous district | 0.812017693 | High-middle SDI |
| Vologda oblast | 0.786532281 | High-middle SDI |
| Kaliningrad oblast | 0.803693646 | High-middle SDI |
| Leningrad oblast | 0.814446452 | High-middle SDI |
| Murmansk oblast | 0.829529044 | High-middle SDI |
| Novgorod oblast | 0.779643289 | High-middle SDI |
| Pskov oblast | 0.787746847 | High-middle SDI |
| Saint Petersburg | 0.859435837 | High-middle SDI |
| Republic of Adygeya | 0.783355473 | High-middle SDI |
| Republic of Kalmykia | 0.754503495 | High-middle SDI |
| Republic of Crimea | 0.713851386 | High-middle SDI |
| Krasnodar kray | 0.799215014 | High-middle SDI |
| Astrakhan oblast | 0.777754786 | High-middle SDI |
| Volgograd oblast | 0.792090776 | High-middle SDI |
| Rostov oblast | 0.804560133 | High-middle SDI |
| Sevastopol | 0.778562755 | High-middle SDI |
| Republic of Dagestan | 0.75489971 | High-middle SDI |
| Republic of Ingushetia | 0.723181052 | High-middle SDI |
| Kabardino-Balkar Republic | 0.773368211 | High-middle SDI |
| Karachay-Cherkess Republic | 0.756823567 | High-middle SDI |
| Republic of North Ossetia-Alania | 0.792451998 | High-middle SDI |
| Chechen Republic | 0.697000298 | High-middle SDI |
| Stavropol kray | 0.785308224 | High-middle SDI |
| Republic of Bashkortostan | 0.807048582 | High-middle SDI |
| Republic of Mari El | 0.777484633 | High-middle SDI |
| Republic of Mordovia | 0.77934414 | High-middle SDI |
| Republic of Tatarstan | 0.815624973 | High-middle SDI |
| Udmurt Republic | 0.786843627 | High-middle SDI |
| Chuvash Republic | 0.779169152 | High-middle SDI |
| Perm kray | 0.796955473 | High-middle SDI |
| Kirov oblast | 0.785897475 | High-middle SDI |
| Nizhny Novgorod oblast | 0.81057854 | High-middle SDI |
| Orenburg oblast | 0.78313786 | High-middle SDI |
| Penza oblast | 0.790346819 | High-middle SDI |
| Samara oblast | 0.814000588 | High-middle SDI |
| Saratov oblast | 0.785980203 | High-middle SDI |
| Ulyanovsk oblast | 0.782641776 | High-middle SDI |
| Kurgan oblast | 0.76552614 | High-middle SDI |
| Sverdlovsk oblast | 0.816064746 | High-middle SDI |
| Khanty-Mansi autonomous area | 0.826709092 | High-middle SDI |
| Yamalo-Nenets autonomous area | 0.837700215 | High-middle SDI |
| Tyumen oblast without autonomous areas | 0.813017728 | High-middle SDI |
| Chelyabinsk oblast | 0.793022546 | High-middle SDI |
| Republic of Altai | 0.749802645 | High-middle SDI |
| Republic of Buryatia | 0.778616162 | High-middle SDI |
| Republic of Tuva | 0.692513465 | High-middle SDI |
| Republic of Khakassia | 0.777318539 | High-middle SDI |
| Altai kray | 0.777099855 | High-middle SDI |
| Zabaikalsk kray | 0.740925147 | High-middle SDI |
| Krasnoyarsk kray | 0.796852774 | High-middle SDI |
| Irkutsk oblast | 0.77351672 | High-middle SDI |
| Kemerovo oblast | 0.782797668 | High-middle SDI |
| Novosibirsk oblast | 0.796528925 | High-middle SDI |
| Omsk oblast | 0.791260764 | High-middle SDI |
| Tomsk oblast | 0.802325795 | High-middle SDI |
| Republic of Sakha (Yakutia) | 0.814649347 | High-middle SDI |
| Kamchatka kray | 0.798113769 | High-middle SDI |
| Primorsky kray | 0.802659761 | High-middle SDI |
| Khabarovsk kray | 0.818047393 | High-middle SDI |
| Amur oblast | 0.793881993 | High-middle SDI |
| Magadan oblast | 0.830546268 | High-middle SDI |
| Sakhalin oblast | 0.822655388 | High-middle SDI |
| Jewish autonomous oblast | 0.765463494 | High-middle SDI |
| Chukotka Autonomous Area | 0.800770455 | High-middle SDI |
| Ukraine (without Crimea & Sevastopol) | 0.761743094 | High-middle SDI |
| Timor-Leste | 0.450689053 | Low SDI |
| Papua New Guinea | 0.418098053 | Low SDI |
| Solomon Islands | 0.429541799 | Low SDI |
| Haiti | 0.448751017 | Low SDI |
| Yemen | 0.453539967 | Low SDI |
| Afghanistan | 0.335068107 | Low SDI |
| Nepal | 0.433952916 | Low SDI |
| Central African Republic | 0.311026626 | Low SDI |
| Democratic Republic of the Congo | 0.390178166 | Low SDI |
| Burundi | 0.291288817 | Low SDI |
| Eritrea | 0.404572056 | Low SDI |
| Madagascar | 0.401385119 | Low SDI |
| Malawi | 0.381985594 | Low SDI |
| Mozambique | 0.327475463 | Low SDI |
| Rwanda | 0.436140248 | Low SDI |
| Somalia | 0.077433678 | Low SDI |
| United Republic of Tanzania | 0.448565569 | Low SDI |
| Uganda | 0.426553554 | Low SDI |
| Benin | 0.37452237 | Low SDI |
| Burkina Faso | 0.284470947 | Low SDI |
| Chad | 0.243516859 | Low SDI |
| Côte d'Ivoire | 0.424540566 | Low SDI |
| Gambia | 0.410077462 | Low SDI |
| Guinea | 0.336555329 | Low SDI |
| Guinea-Bissau | 0.353448423 | Low SDI |
| Liberia | 0.353229409 | Low SDI |
| Mali | 0.271175692 | Low SDI |
| Niger | 0.170310328 | Low SDI |
| Senegal | 0.409005254 | Low SDI |
| Sierra Leone | 0.35900867 | Low SDI |
| Togo | 0.410016394 | Low SDI |
| South Sudan | 0.278377554 | Low SDI |
| Bauchi | 0.294070246 | Low SDI |
| Borno | 0.398589587 | Low SDI |
| Gombe | 0.379904259 | Low SDI |
| Jigawa | 0.287216991 | Low SDI |
| Kaduna | 0.442437011 | Low SDI |
| Kano | 0.408256207 | Low SDI |
| Katsina | 0.297115378 | Low SDI |
| Kebbi | 0.284501095 | Low SDI |
| Niger | 0.42518919 | Low SDI |
| Sokoto | 0.254081326 | Low SDI |
| Taraba | 0.438659637 | Low SDI |
| Yobe | 0.312002634 | Low SDI |
| Zamfara | 0.267800369 | Low SDI |
| Bihar, Rural | 0.434277772 | Low SDI |
| Jharkhand, Rural | 0.463971644 | Low SDI |
| Madhya Pradesh, Rural | 0.464482329 | Low SDI |
| Tigray | 0.38400255 | Low SDI |
| Afar | 0.286502778 | Low SDI |
| Amhara | 0.322156172 | Low SDI |
| Oromia | 0.337961203 | Low SDI |
| Somali | 0.27014767 | Low SDI |
| Benishangul-Gumuz | 0.323654895 | Low SDI |
| Southern Nations, Nationalities, and Peoples | 0.357248469 | Low SDI |
| Harari | 0.539739601 | Low SDI |
| Gambella | 0.460576186 | Low SDI |
| Addis Ababa | 0.695287927 | Low SDI |
| Dire Dawa | 0.542617703 | Low SDI |
| Balochistan | 0.417109886 | Low SDI |
| Gilgit-Baltistan | 0.399312068 | Low SDI |
| Khyber Pakhtunkhwa | 0.451366327 | Low SDI |
| Democratic People's Republic of Korea | 0.56945513 | Low-middle SDI |
| Cambodia | 0.473999694 | Low-middle SDI |
| Lao People's Democratic Republic | 0.489280726 | Low-middle SDI |
| Myanmar | 0.528492169 | Low-middle SDI |
| Kiribati | 0.525957502 | Low-middle SDI |
| Marshall Islands | 0.573524783 | Low-middle SDI |
| Micronesia (Federated States of) | 0.588012508 | Low-middle SDI |
| Samoa | 0.592340278 | Low-middle SDI |
| Vanuatu | 0.472796337 | Low-middle SDI |
| Kyrgyzstan | 0.609180728 | Low-middle SDI |
| Mongolia | 0.618744133 | Low-middle SDI |
| Tajikistan | 0.536613238 | Low-middle SDI |
| Belize | 0.61055234 | Low-middle SDI |
| Bolivia (Plurinational State of) | 0.604496662 | Low-middle SDI |
| El Salvador | 0.565569678 | Low-middle SDI |
| Guatemala | 0.540099007 | Low-middle SDI |
| Honduras | 0.513585699 | Low-middle SDI |
| Nicaragua | 0.52364671 | Low-middle SDI |
| Venezuela (Bolivarian Republic of) | 0.596599587 | Low-middle SDI |
| Egypt | 0.603962121 | Low-middle SDI |
| Morocco | 0.561680434 | Low-middle SDI |
| Bangladesh | 0.493106236 | Low-middle SDI |
| Bhutan | 0.476724988 | Low-middle SDI |
| Angola | 0.482946052 | Low-middle SDI |
| Congo | 0.586908906 | Low-middle SDI |
| Comoros | 0.476955685 | Low-middle SDI |
| Djibouti | 0.489200321 | Low-middle SDI |
| Zambia | 0.510230369 | Low-middle SDI |
| Lesotho | 0.51157061 | Low-middle SDI |
| Namibia | 0.618073651 | Low-middle SDI |
| Eswatini | 0.586216849 | Low-middle SDI |
| Zimbabwe | 0.475577138 | Low-middle SDI |
| Cameroon | 0.480364523 | Low-middle SDI |
| Cabo Verde | 0.533600978 | Low-middle SDI |
| Ghana | 0.563348184 | Low-middle SDI |
| Mauritania | 0.495266784 | Low-middle SDI |
| Sao Tome and Principe | 0.503305577 | Low-middle SDI |
| Tuvalu | 0.578627145 | Low-middle SDI |
| Tibet | 0.483893412 | Low-middle SDI |
| Sudan | 0.542748299 | Low-middle SDI |
| Bengkulu | 0.613885329 | Low-middle SDI |
| Lampung | 0.60891275 | Low-middle SDI |
| Central Java | 0.613780247 | Low-middle SDI |
| West Nusa Tenggara | 0.587663369 | Low-middle SDI |
| East Nusa Tenggara | 0.550545868 | Low-middle SDI |
| West Kalimantan | 0.587438981 | Low-middle SDI |
| Central Sulawesi | 0.617544908 | Low-middle SDI |
| Southeast Sulawesi | 0.618324728 | Low-middle SDI |
| Gorontalo | 0.571050074 | Low-middle SDI |
| West Sulawesi | 0.576458969 | Low-middle SDI |
| Maluku | 0.581624279 | Low-middle SDI |
| North Maluku | 0.563444978 | Low-middle SDI |
| Acre | 0.562074727 | Low-middle SDI |
| Alagoas | 0.529742892 | Low-middle SDI |
| Amazonas | 0.603585976 | Low-middle SDI |
| Bahia | 0.574142222 | Low-middle SDI |
| Ceará | 0.563912693 | Low-middle SDI |
| Maranhão | 0.49216193 | Low-middle SDI |
| Pará | 0.577314362 | Low-middle SDI |
| Paraíba | 0.557922296 | Low-middle SDI |
| Pernambuco | 0.583214758 | Low-middle SDI |
| Piaui | 0.520291625 | Low-middle SDI |
| Rio Grande do Norte | 0.585328241 | Low-middle SDI |
| Rondônia | 0.618510473 | Low-middle SDI |
| Roraima | 0.609883228 | Low-middle SDI |
| Sergipe | 0.590589987 | Low-middle SDI |
| Tocantins | 0.601403 | Low-middle SDI |
| Adamawa | 0.474621913 | Low-middle SDI |
| Akwa Ibom | 0.589407255 | Low-middle SDI |
| Bayelsa | 0.558802657 | Low-middle SDI |
| Benue | 0.49859261 | Low-middle SDI |
| Cross River | 0.562249026 | Low-middle SDI |
| Delta | 0.614789164 | Low-middle SDI |
| Ebonyi | 0.540874531 | Low-middle SDI |
| Ekiti | 0.609689218 | Low-middle SDI |
| Enugu | 0.60872942 | Low-middle SDI |
| Kogi | 0.560608509 | Low-middle SDI |
| Kwara | 0.536539828 | Low-middle SDI |
| Nasarawa | 0.518505244 | Low-middle SDI |
| Ogun | 0.569648186 | Low-middle SDI |
| Ondo | 0.592384037 | Low-middle SDI |
| Oyo | 0.584428907 | Low-middle SDI |
| Plateau | 0.530865998 | Low-middle SDI |
| Baringo | 0.514621991 | Low-middle SDI |
| Bomet | 0.530035509 | Low-middle SDI |
| Bungoma | 0.488274636 | Low-middle SDI |
| Busia | 0.478169739 | Low-middle SDI |
| Elgeyo-Marakwet | 0.525285148 | Low-middle SDI |
| Embu | 0.548377806 | Low-middle SDI |
| Garissa | 0.32165019 | Low-middle SDI |
| Homa Bay | 0.507694781 | Low-middle SDI |
| Isiolo | 0.435133975 | Low-middle SDI |
| Kajiado | 0.501634865 | Low-middle SDI |
| Kakamega | 0.508518514 | Low-middle SDI |
| Kericho | 0.520411743 | Low-middle SDI |
| Kiambu | 0.593046232 | Low-middle SDI |
| Kilifi | 0.486686815 | Low-middle SDI |
| Kirinyaga | 0.546740689 | Low-middle SDI |
| Kisii | 0.549398782 | Low-middle SDI |
| Kisumu | 0.548720604 | Low-middle SDI |
| Kitui | 0.474177297 | Low-middle SDI |
| Kwale | 0.47965716 | Low-middle SDI |
| Laikipia | 0.576686523 | Low-middle SDI |
| Lamu | 0.505575183 | Low-middle SDI |
| Machakos | 0.55015778 | Low-middle SDI |
| Makueni | 0.514230695 | Low-middle SDI |
| Mandera | 0.239926734 | Low-middle SDI |
| Marsabit | 0.399976958 | Low-middle SDI |
| Meru | 0.509152437 | Low-middle SDI |
| Migori | 0.482207014 | Low-middle SDI |
| Mombasa | 0.598166665 | Low-middle SDI |
| Murang'a | 0.55292203 | Low-middle SDI |
| Nairobi | 0.684188978 | Low-middle SDI |
| Nakuru | 0.5721094 | Low-middle SDI |
| Nandi | 0.516039708 | Low-middle SDI |
| Narok | 0.458227005 | Low-middle SDI |
| Nyamira | 0.593029203 | Low-middle SDI |
| Nyandarua | 0.577540201 | Low-middle SDI |
| Nyeri | 0.579362636 | Low-middle SDI |
| Samburu | 0.371475608 | Low-middle SDI |
| Siaya | 0.484421932 | Low-middle SDI |
| Taita Taveta | 0.542579215 | Low-middle SDI |
| Tana River | 0.389358757 | Low-middle SDI |
| Tharaka Nithi | 0.528624517 | Low-middle SDI |
| Trans Nzoia | 0.549784994 | Low-middle SDI |
| Turkana | 0.3683857 | Low-middle SDI |
| Uasin Gishu | 0.567922916 | Low-middle SDI |
| Vihiga | 0.527054687 | Low-middle SDI |
| Wajir | 0.258713923 | Low-middle SDI |
| West Pokot | 0.44769021 | Low-middle SDI |
| Bihar, Urban | 0.591183966 | Low-middle SDI |
| Andhra Pradesh, Rural | 0.495920251 | Low-middle SDI |
| Arunachal Pradesh, Rural | 0.549469207 | Low-middle SDI |
| Assam, Rural | 0.551639812 | Low-middle SDI |
| Chhattisgarh, Rural | 0.486779046 | Low-middle SDI |
| Gujarat, Rural | 0.549296846 | Low-middle SDI |
| Haryana, Rural | 0.579178153 | Low-middle SDI |
| Jammu & Kashmir and Ladakh, Rural | 0.572664356 | Low-middle SDI |
| Karnataka, Rural | 0.526848679 | Low-middle SDI |
| Maharashtra, Rural | 0.574895865 | Low-middle SDI |
| Manipur, Rural | 0.560840633 | Low-middle SDI |
| Meghalaya, Rural | 0.519625901 | Low-middle SDI |
| Mizoram, Rural | 0.57456484 | Low-middle SDI |
| Nagaland, Rural | 0.600219084 | Low-middle SDI |
| Odisha, Rural | 0.520670909 | Low-middle SDI |
| Punjab, Rural | 0.585512634 | Low-middle SDI |
| Rajasthan, Rural | 0.478194737 | Low-middle SDI |
| Sikkim, Rural | 0.593976905 | Low-middle SDI |
| Tamil Nadu, Rural | 0.580536414 | Low-middle SDI |
| Telangana, Rural | 0.483176983 | Low-middle SDI |
| Tripura, Rural | 0.515999282 | Low-middle SDI |
| Uttar Pradesh, Rural | 0.490024946 | Low-middle SDI |
| Uttarakhand, Rural | 0.61464175 | Low-middle SDI |
| West Bengal, Rural | 0.502428983 | Low-middle SDI |
| Other Union Territories, Rural | 0.601933292 | Low-middle SDI |
| Azad Jammu & Kashmir | 0.541342775 | Low-middle SDI |
| Punjab | 0.520053339 | Low-middle SDI |
| Sindh | 0.513737094 | Low-middle SDI |
| Maldives | 0.657665453 | Middle SDI |
| Sri Lanka | 0.701371778 | Middle SDI |
| Thailand | 0.682657272 | Middle SDI |
| Viet Nam | 0.621620778 | Middle SDI |
| Fiji | 0.669068631 | Middle SDI |
| Tonga | 0.629100964 | Middle SDI |
| Armenia | 0.702496602 | Middle SDI |
| Azerbaijan | 0.695410598 | Middle SDI |
| Turkmenistan | 0.683039569 | Middle SDI |
| Uzbekistan | 0.664964654 | Middle SDI |
| Albania | 0.706888685 | Middle SDI |
| Cuba | 0.669331767 | Middle SDI |
| Dominican Republic | 0.619170694 | Middle SDI |
| Grenada | 0.6693506 | Middle SDI |
| Guyana | 0.650902479 | Middle SDI |
| Jamaica | 0.68306364 | Middle SDI |
| Saint Lucia | 0.672601687 | Middle SDI |
| Saint Vincent and the Grenadines | 0.640886762 | Middle SDI |
| Suriname | 0.641162711 | Middle SDI |
| Ecuador | 0.665675436 | Middle SDI |
| Peru | 0.662036006 | Middle SDI |
| Colombia | 0.65664043 | Middle SDI |
| Costa Rica | 0.704369665 | Middle SDI |
| Panama | 0.706659844 | Middle SDI |
| Paraguay | 0.650487525 | Middle SDI |
| Algeria | 0.659720087 | Middle SDI |
| Iraq | 0.662777495 | Middle SDI |
| Palestine | 0.629201641 | Middle SDI |
| Syrian Arab Republic | 0.622855859 | Middle SDI |
| Tunisia | 0.681701488 | Middle SDI |
| Equatorial Guinea | 0.663978286 | Middle SDI |
| Gabon | 0.639080604 | Middle SDI |
| Botswana | 0.643077969 | Middle SDI |
| Nauru | 0.627549782 | Middle SDI |
| Tokelau | 0.68701842 | Middle SDI |
| Eastern Cape | 0.619101287 | Middle SDI |
| Free State | 0.678893967 | Middle SDI |
| Gauteng | 0.736905342 | Middle SDI |
| KwaZulu-Natal | 0.662386215 | Middle SDI |
| Limpopo | 0.613431617 | Middle SDI |
| Mpumalanga | 0.648324523 | Middle SDI |
| North-West | 0.654616033 | Middle SDI |
| Northern Cape | 0.665813245 | Middle SDI |
| Western Cape | 0.719732052 | Middle SDI |
| Anhui | 0.661241263 | Middle SDI |
| Gansu | 0.629648423 | Middle SDI |
| Guangxi | 0.677747142 | Middle SDI |
| Guizhou | 0.622926981 | Middle SDI |
| Hainan | 0.70526301 | Middle SDI |
| Hebei | 0.703535825 | Middle SDI |
| Henan | 0.699479878 | Middle SDI |
| Hubei | 0.711832348 | Middle SDI |
| Hunan | 0.692120518 | Middle SDI |
| Jiangxi | 0.678215672 | Middle SDI |
| Ningxia | 0.695319453 | Middle SDI |
| Qinghai | 0.648309483 | Middle SDI |
| Shanxi | 0.711816196 | Middle SDI |
| Sichuan | 0.672696014 | Middle SDI |
| Xinjiang | 0.70686651 | Middle SDI |
| Yunnan | 0.627205795 | Middle SDI |
| Aguascalientes | 0.682557435 | Middle SDI |
| Baja California | 0.704776585 | Middle SDI |
| Baja California Sur | 0.710175355 | Middle SDI |
| Campeche | 0.665087938 | Middle SDI |
| Coahuila | 0.678075116 | Middle SDI |
| Colima | 0.699338436 | Middle SDI |
| Chiapas | 0.569756592 | Middle SDI |
| Chihuahua | 0.674472052 | Middle SDI |
| Mexico City | 0.759378377 | Middle SDI |
| Durango | 0.640562517 | Middle SDI |
| Guanajuato | 0.647044734 | Middle SDI |
| Guerrero | 0.584126986 | Middle SDI |
| Hidalgo | 0.633128071 | Middle SDI |
| Jalisco | 0.677078025 | Middle SDI |
| México | 0.681505383 | Middle SDI |
| Michoacán de Ocampo | 0.613949206 | Middle SDI |
| Morelos | 0.670104932 | Middle SDI |
| Nayarit | 0.657928691 | Middle SDI |
| Nuevo León | 0.712152517 | Middle SDI |
| Oaxaca | 0.588389144 | Middle SDI |
| Puebla | 0.622884968 | Middle SDI |
| Querétaro | 0.684048164 | Middle SDI |
| Quintana Roo | 0.682591131 | Middle SDI |
| San Luis Potosí | 0.647579585 | Middle SDI |
| Sinaloa | 0.678348037 | Middle SDI |
| Sonora | 0.709903997 | Middle SDI |
| Tabasco | 0.649850519 | Middle SDI |
| Tamaulipas | 0.682912198 | Middle SDI |
| Tlaxcala | 0.64898896 | Middle SDI |
| Veracruz de Ignacio de la Llave | 0.627366789 | Middle SDI |
| Yucatán | 0.654459012 | Middle SDI |
| Zacatecas | 0.636670528 | Middle SDI |
| Aceh | 0.671759479 | Middle SDI |
| North Sumatra | 0.669499052 | Middle SDI |
| West Sumatra | 0.66784543 | Middle SDI |
| Jambi | 0.640779478 | Middle SDI |
| South Sumatra | 0.646421244 | Middle SDI |
| Bangka-Belitung Islands | 0.644388463 | Middle SDI |
| West Java | 0.644279321 | Middle SDI |
| Yogyakarta | 0.676829859 | Middle SDI |
| East Java | 0.646540022 | Middle SDI |
| Banten | 0.641544087 | Middle SDI |
| Bali | 0.652382779 | Middle SDI |
| Central Kalimantan | 0.639931265 | Middle SDI |
| South Kalimantan | 0.622221912 | Middle SDI |
| North Sulawesi | 0.652614588 | Middle SDI |
| South Sulawesi | 0.622994177 | Middle SDI |
| West Papua | 0.676555222 | Middle SDI |
| Papua | 0.646553603 | Middle SDI |
| Amapá | 0.629807813 | Middle SDI |
| Espírito Santo | 0.667428625 | Middle SDI |
| Goiás | 0.639347711 | Middle SDI |
| Minas Gerais | 0.648904701 | Middle SDI |
| Mato Grosso do Sul | 0.642693307 | Middle SDI |
| Mato Grosso | 0.647043759 | Middle SDI |
| Paraná | 0.669860641 | Middle SDI |
| Rio de Janeiro | 0.710470527 | Middle SDI |
| Rio Grande do Sul | 0.689722837 | Middle SDI |
| Santa Catarina | 0.694839624 | Middle SDI |
| São Paulo | 0.711182598 | Middle SDI |
| Abia | 0.629261465 | Middle SDI |
| Anambra | 0.62862687 | Middle SDI |
| Edo | 0.6270757 | Middle SDI |
| FCT (Abuja) | 0.621406693 | Middle SDI |
| Imo | 0.629384576 | Middle SDI |
| Lagos | 0.679573249 | Middle SDI |
| Osun | 0.627939478 | Middle SDI |
| Rivers | 0.638605802 | Middle SDI |
| Andhra Pradesh, Urban | 0.647551072 | Middle SDI |
| Arunachal Pradesh, Urban | 0.68419449 | Middle SDI |
| Assam, Urban | 0.675446354 | Middle SDI |
| Chhattisgarh, Urban | 0.679763349 | Middle SDI |
| Gujarat, Urban | 0.703282867 | Middle SDI |
| Haryana, Urban | 0.703932889 | Middle SDI |
| Jammu & Kashmir and Ladakh, Urban | 0.684343769 | Middle SDI |
| Jharkhand, Urban | 0.670458961 | Middle SDI |
| Karnataka, Urban | 0.658831954 | Middle SDI |
| Kerala, Urban | 0.672698917 | Middle SDI |
| Madhya Pradesh, Urban | 0.675329604 | Middle SDI |
| Maharashtra, Urban | 0.705231591 | Middle SDI |
| Manipur, Urban | 0.632756357 | Middle SDI |
| Meghalaya, Urban | 0.685725867 | Middle SDI |
| Mizoram, Urban | 0.670528706 | Middle SDI |
| Nagaland, Urban | 0.691617405 | Middle SDI |
| Odisha, Urban | 0.657514066 | Middle SDI |
| Punjab, Urban | 0.698430939 | Middle SDI |
| Rajasthan, Urban | 0.671757713 | Middle SDI |
| Sikkim, Urban | 0.695740102 | Middle SDI |
| Tamil Nadu, Urban | 0.676173806 | Middle SDI |
| Telangana, Urban | 0.667241582 | Middle SDI |
| Tripura, Urban | 0.633348097 | Middle SDI |
| Uttar Pradesh, Urban | 0.63211101 | Middle SDI |
| West Bengal, Urban | 0.6493937 | Middle SDI |
| Delhi, Rural | 0.655467765 | Middle SDI |
| Goa, Rural | 0.687517157 | Middle SDI |
| Himachal Pradesh, Rural | 0.629512224 | Middle SDI |
| Kerala, Rural | 0.659487084 | Middle SDI |
| Other Union Territories, Urban | 0.708204745 | Middle SDI |
| Alborz | 0.748208954 | Middle SDI |
| Ardebil | 0.658777858 | Middle SDI |
| East Azarbayejan | 0.667933993 | Middle SDI |
| West Azarbayejan | 0.626918833 | Middle SDI |
| Bushehr | 0.708677286 | Middle SDI |
| Chahar Mahaal and Bakhtiari | 0.678339055 | Middle SDI |
| Fars | 0.715109154 | Middle SDI |
| Gilan | 0.712361968 | Middle SDI |
| Golestan | 0.656422158 | Middle SDI |
| Hamadan | 0.666968762 | Middle SDI |
| Hormozgan | 0.670775004 | Middle SDI |
| Ilam | 0.705185457 | Middle SDI |
| Isfahan | 0.709893952 | Middle SDI |
| Kerman | 0.66878396 | Middle SDI |
| Kermanshah | 0.674511267 | Middle SDI |
| North Khorasan | 0.651483141 | Middle SDI |
| Khorasan-e-Razavi | 0.67053956 | Middle SDI |
| South Khorasan | 0.653364832 | Middle SDI |
| Khuzestan | 0.669816556 | Middle SDI |
| Kohgiluyeh and Boyer-Ahmad | 0.694488035 | Middle SDI |
| Kurdistan | 0.642334326 | Middle SDI |
| Lorestan | 0.669421197 | Middle SDI |
| Markazi | 0.682789151 | Middle SDI |
| Mazandaran | 0.729935836 | Middle SDI |
| Qazvin | 0.687516004 | Middle SDI |
| Qom | 0.694133773 | Middle SDI |
| Semnan | 0.724001841 | Middle SDI |
| Sistan and Baluchistan | 0.549869409 | Middle SDI |
| Tehran | 0.776102826 | Middle SDI |
| Yazd | 0.713637577 | Middle SDI |
| Zanjan | 0.661463751 | Middle SDI |
| Mountain Province | 0.51921863 | Middle SDI |
| Ifugao | 0.597325624 | Middle SDI |
| Benguet | 0.716293119 | Middle SDI |
| Abra | 0.654173651 | Middle SDI |
| Apayao | 0.60770129 | Middle SDI |
| Kalinga | 0.575239213 | Middle SDI |
| La Union | 0.661006064 | Middle SDI |
| Ilocos Norte | 0.687657015 | Middle SDI |
| Ilocos Sur | 0.671836522 | Middle SDI |
| Pangasinan | 0.666135554 | Middle SDI |
| Nueva Vizcaya | 0.616609509 | Middle SDI |
| Cagayan | 0.63433253 | Middle SDI |
| Isabela | 0.636783935 | Middle SDI |
| Quirino | 0.575885131 | Middle SDI |
| Batanes | 0.682565947 | Middle SDI |
| Bataan | 0.660374268 | Middle SDI |
| Zambales | 0.65425423 | Middle SDI |
| Tarlac | 0.650760386 | Middle SDI |
| Pampanga | 0.697271285 | Middle SDI |
| Bulacan | 0.708002625 | Middle SDI |
| Nueva Ecija | 0.650591609 | Middle SDI |
| Aurora | 0.614063114 | Middle SDI |
| Rizal | 0.710524418 | Middle SDI |
| Cavite | 0.72917856 | Middle SDI |
| Laguna | 0.701620417 | Middle SDI |
| Batangas | 0.686055208 | Middle SDI |
| Quezon | 0.630168626 | Middle SDI |
| Occidental Mindoro | 0.46028876 | Middle SDI |
| Oriental Mindoro | 0.60555167 | Middle SDI |
| Romblon | 0.527572654 | Middle SDI |
| Palawan | 0.527211201 | Middle SDI |
| Marinduque | 0.549650294 | Middle SDI |
| Catanduanes | 0.60942324 | Middle SDI |
| Camarines Norte | 0.594459098 | Middle SDI |
| Sorsogon | 0.600776304 | Middle SDI |
| Albay | 0.640151269 | Middle SDI |
| Masbate | 0.458060424 | Middle SDI |
| Camarines Sur | 0.633743794 | Middle SDI |
| Capiz | 0.5714003 | Middle SDI |
| Aklan | 0.64042058 | Middle SDI |
| Antique | 0.569823295 | Middle SDI |
| Negros Occidental | 0.604235111 | Middle SDI |
| Iloilo | 0.673508341 | Middle SDI |
| Guimaras | 0.609860186 | Middle SDI |
| Negros Oriental | 0.578181475 | Middle SDI |
| Cebu | 0.658154663 | Middle SDI |
| Bohol | 0.604933545 | Middle SDI |
| Siquijor | 0.600945243 | Middle SDI |
| Southern Leyte | 0.60149118 | Middle SDI |
| Eastern Samar | 0.499800385 | Middle SDI |
| Northern Samar | 0.52396754 | Middle SDI |
| Samar (Western Samar) | 0.527590959 | Middle SDI |
| Leyte | 0.611701754 | Middle SDI |
| Biliran | 0.643027685 | Middle SDI |
| Zamboanga Sibugay | 0.549420679 | Middle SDI |
| Zamboanga Del Norte | 0.53803222 | Middle SDI |
| Zamboanga Del Sur | 0.630995266 | Middle SDI |
| Misamis Occidental | 0.588184738 | Middle SDI |
| Bukidnon | 0.551058807 | Middle SDI |
| Lanao Del Norte | 0.587617211 | Middle SDI |
| Misamis Oriental | 0.662873429 | Middle SDI |
| Camiguin | 0.632089767 | Middle SDI |
| Davao Oriental | 0.547236917 | Middle SDI |
| Davao de Oro | 0.533098715 | Middle SDI |
| Davao Del Sur | 0.659917629 | Middle SDI |
| Davao Occidental | 0.578314908 | Middle SDI |
| Davao Del Norte | 0.636970822 | Middle SDI |
| South Cotabato | 0.632536869 | Middle SDI |
| Sultan Kudarat | 0.519757294 | Middle SDI |
| Cotabato (North Cotabato) | 0.55211245 | Middle SDI |
| Sarangani | 0.582418699 | Middle SDI |
| Agusan Del Norte | 0.615707618 | Middle SDI |
| Agusan Del Sur | 0.541666497 | Middle SDI |
| Surigao Del Sur | 0.587486668 | Middle SDI |
| Surigao Del Norte | 0.627545678 | Middle SDI |
| Dinagat Islands | 0.622041414 | Middle SDI |
| Tawi-Tawi | 0.535621341 | Middle SDI |
| Basilan | 0.546108643 | Middle SDI |
| Sulu | 0.48399862 | Middle SDI |
| Maguindanao | 0.51070243 | Middle SDI |
| Lanao Del Sur | 0.532909532 | Middle SDI |
| National Capital Region | 0.751536473 | Middle SDI |
| Islamabad Capital Territory | 0.695559154 | Middle SDI |
| Global | 0.666367819 |  |
| Southeast Asia, East Asia, and Oceania | 0.696630914 |  |
| East Asia | 0.722912119 |  |
| China | 0.71867919 |  |
| Southeast Asia | 0.64907177 |  |
| Indonesia | 0.657934796 |  |
| Philippines | 0.651920253 |  |
| Oceania | 0.467359461 |  |
| Central Europe, Eastern Europe, and Central Asia | 0.768649142 |  |
| Central Asia | 0.674963478 |  |
| Central Europe | 0.795780357 |  |
| Poland | 0.812073312 |  |
| Eastern Europe | 0.803414319 |  |
| Russian Federation | 0.809111108 |  |
| Ukraine | 0.761045561 |  |
| High-income | 0.85286503 |  |
| High-income Asia Pacific | 0.877157409 |  |
| Japan | 0.871459701 |  |
| Australasia | 0.845644432 |  |
| New Zealand | 0.850145187 |  |
| Western Europe | 0.848728514 |  |
| Italy | 0.805537426 |  |
| Norway | 0.916631633 |  |
| Sweden | 0.887384361 |  |
| United Kingdom | 0.858444983 |  |
| Southern Latin America | 0.743029817 |  |
| High-income North America | 0.86421664 |  |
| United States of America | 0.863243823 |  |
| Latin America and Caribbean | 0.646195591 |  |
| Caribbean | 0.6423146 |  |
| Andean Latin America | 0.654007956 |  |
| Central Latin America | 0.641931122 |  |
| Mexico | 0.66496867 |  |
| Tropical Latin America | 0.648941531 |  |
| Brazil | 0.648846512 |  |
| North Africa and Middle East | 0.658716072 |  |
| North Africa and Middle East | 0.658716072 |  |
| Iran (Islamic Republic of) | 0.69729326 |  |
| South Asia | 0.559642669 |  |
| South Asia | 0.559642669 |  |
| India | 0.5777383 |  |
| Pakistan | 0.504275856 |  |
| Sub-Saharan Africa | 0.461224007 |  |
| Central Sub-Saharan Africa | 0.484517732 |  |
| Eastern Sub-Saharan Africa | 0.412187942 |  |
| Ethiopia | 0.360727644 |  |
| Kenya | 0.524783146 |  |
| Southern Sub-Saharan Africa | 0.643347819 |  |
| South Africa | 0.681292244 |  |
| Western Sub-Saharan Africa | 0.446420999 |  |
| Nigeria | 0.503698612 |  |
| North East England | 0.825842759 |  |
| North West England | 0.843295382 |  |
| Yorkshire and the Humber | 0.836407087 |  |
| East Midlands | 0.835023214 |  |
| West Midlands | 0.834648192 |  |
| East of England | 0.85577408 |  |
| Greater London | 0.9041535 |  |
| South East England | 0.875365568 |  |
| South West England | 0.860849638 |  |
| England | 0.860552694 |  |
| Andhra Pradesh | 0.552472881 |  |
| Arunachal Pradesh | 0.585180701 |  |
| Assam | 0.573442541 |  |
| Bihar | 0.45805086 |  |
| Chhattisgarh | 0.540359862 |  |
| Delhi | 0.728650031 |  |
| Goa | 0.724320503 |  |
| Gujarat | 0.622975241 |  |
| Haryana | 0.629602575 |  |
| Himachal Pradesh | 0.643033305 |  |
| Jammu & Kashmir and Ladakh | 0.609583681 |  |
| Jharkhand | 0.5242297 |  |
| Karnataka | 0.585910724 |  |
| Kerala | 0.666417241 |  |
| Madhya Pradesh | 0.531115567 |  |
| Maharashtra | 0.639494993 |  |
| Manipur | 0.584558179 |  |
| Meghalaya | 0.560405068 |  |
| Mizoram | 0.62843683 |  |
| Nagaland | 0.637648412 |  |
| Odisha | 0.551364782 |  |
| Punjab | 0.632901572 |  |
| Rajasthan | 0.536745684 |  |
| Sikkim | 0.635190296 |  |
| Tamil Nadu | 0.629914474 |  |
| Telangana | 0.567480596 |  |
| Tripura | 0.555782348 |  |
| Uttar Pradesh | 0.527579329 |  |
| Uttarakhand | 0.661804198 |  |
| West Bengal | 0.556791581 |  |
| China (without Hong Kong and Macao) | 0.717406624 |  |
| Other Union Territories | 0.676062684 |  |
| Central | 0.577789494 |  |
| Coast | 0.518014043 |  |
| Eastern | 0.508217906 |  |
| Nairobi | 0.684188971 |  |
| North Eastern | 0.264360533 |  |
| Nyanza | 0.525077558 |  |
| Rift Valley | 0.51425552 |  |
| Western | 0.504497721 |  |

SDI: the socio-demographic index.
