# Supplementary material for: Assessing the global dengue burden: Incidence, mortality, and disability trends over three decades
Source: PLoS Negl Trop Dis. 2025 Mar 12;19(3):e0012932. doi: 10.1371/journal.pntd.0012932 (PMC11925280; doi:10.1371/journal.pntd.0012932)
Supplement: S3 Table — (DOCX) [file pntd.0012932.s003.docx]

**S3 Table. Socio-demographic Index values for all estimated GBD 2021 locations, 1990-2021.**

| **Loaction** | **1990** | **1991** | **1992** | **1993** | **1994** | **1995** | **1996** | **1997** | **1998** | **1999** | **2000** | **2001** | **2002** | **2003** | **2004** | **2005** | **2006** | **2007** | **2008** | **2009** | **2010** | **2011** | **2012** | **2013** | **2014** | **2015** | **2016** | **2017** | **2018** | **2019** | **2020** | **2021** |
| --- | --- | --- | --- | --- | --- | --- | --- | --- | --- | --- | --- | --- | --- | --- | --- | --- | --- | --- | --- | --- | --- | --- | --- | --- | --- | --- | --- | --- | --- | --- | --- | --- |
| Global | 0.525528856 | 0.530272485 | 0.534636544 | 0.538598319 | 0.542389433 | 0.546327999 | 0.550703413 | 0.555139426 | 0.559380567 | 0.563738017 | 0.568003164 | 0.571777531 | 0.57568796 | 0.579907077 | 0.584378588 | 0.58897852 | 0.593756623 | 0.598641368 | 0.603370099 | 0.60775944 | 0.612952497 | 0.617759223 | 0.621844977 | 0.626343279 | 0.630982367 | 0.635441488 | 0.640153586 | 0.645564304 | 0.651048474 | 0.656575898 | 0.661343543 | 0.665820975 |
| Central Europe, eastern Europe, and central Asia | 0.638316821 | 0.644260725 | 0.650300228 | 0.654984112 | 0.658896401 | 0.663230612 | 0.6673985 | 0.671061614 | 0.674441709 | 0.678044379 | 0.682043284 | 0.686165462 | 0.690410622 | 0.695480481 | 0.701414414 | 0.707071788 | 0.71222406 | 0.717221963 | 0.722555135 | 0.72727958 | 0.732077661 | 0.736078287 | 0.739674513 | 0.74328837 | 0.74665736 | 0.749877167 | 0.753359993 | 0.757098469 | 0.760760366 | 0.764227123 | 0.766837051 | 0.769466346 |
| Central Asia | 0.553361946 | 0.555147774 | 0.557269541 | 0.560082892 | 0.562699531 | 0.565585602 | 0.568760584 | 0.571766483 | 0.574659386 | 0.578120701 | 0.582155536 | 0.586645798 | 0.591457113 | 0.596529769 | 0.601736719 | 0.60729382 | 0.613078276 | 0.619104578 | 0.624930378 | 0.630102885 | 0.635295597 | 0.640313561 | 0.644673516 | 0.648888141 | 0.65299988 | 0.656817827 | 0.660312421 | 0.663576782 | 0.666666449 | 0.669837304 | 0.67248671 | 0.675163978 |
| Armenia | 0.544414535 | 0.547561863 | 0.548974724 | 0.551214674 | 0.553783844 | 0.556815513 | 0.560537778 | 0.564627462 | 0.569556663 | 0.574323064 | 0.579437569 | 0.585419295 | 0.592710614 | 0.60080917 | 0.609031766 | 0.617759634 | 0.626688335 | 0.635870982 | 0.644431591 | 0.649714845 | 0.654811534 | 0.659818092 | 0.664833143 | 0.669534496 | 0.67393219 | 0.678054635 | 0.681746395 | 0.685980265 | 0.690276872 | 0.694765962 | 0.698268899 | 0.701833194 |
| Azerbaijan | 0.595986033 | 0.595807712 | 0.594651335 | 0.593542676 | 0.592088785 | 0.589441457 | 0.586315946 | 0.583221813 | 0.580392893 | 0.579192506 | 0.580020573 | 0.582193708 | 0.585716143 | 0.590038705 | 0.594646085 | 0.602485193 | 0.613771097 | 0.626110926 | 0.636901109 | 0.645163341 | 0.652056507 | 0.657971629 | 0.662897002 | 0.667658779 | 0.672162741 | 0.676244911 | 0.679807643 | 0.683263204 | 0.686561093 | 0.689834715 | 0.692605192 | 0.694851274 |
| Georgia | 0.656136044 | 0.662113186 | 0.665021428 | 0.663466862 | 0.658134139 | 0.651363604 | 0.64455809 | 0.638525631 | 0.633347931 | 0.630277946 | 0.630265991 | 0.6333883 | 0.638370108 | 0.644065579 | 0.648920978 | 0.653616005 | 0.658403372 | 0.663697966 | 0.668442496 | 0.672449381 | 0.677034892 | 0.681567044 | 0.685519223 | 0.688603268 | 0.692155207 | 0.697316133 | 0.70373149 | 0.710874718 | 0.717993654 | 0.724536462 | 0.729125889 | 0.732473604 |
| Kazakhstan | 0.589435804 | 0.590967832 | 0.596127664 | 0.603138328 | 0.6105911 | 0.618573582 | 0.626013138 | 0.631166626 | 0.635388624 | 0.640180485 | 0.644689623 | 0.648397258 | 0.651529335 | 0.654835926 | 0.658733071 | 0.662943942 | 0.667048858 | 0.671367208 | 0.676352869 | 0.681606839 | 0.686996643 | 0.691758952 | 0.695312767 | 0.699053593 | 0.703511921 | 0.708229736 | 0.71256904 | 0.716026688 | 0.718531999 | 0.720797602 | 0.722788768 | 0.725144495 |
| Kyrgyzstan | 0.519407652 | 0.523746714 | 0.527901552 | 0.532431008 | 0.53623298 | 0.53905282 | 0.541628927 | 0.54334115 | 0.543258546 | 0.542867282 | 0.543036487 | 0.543805551 | 0.54437467 | 0.545649262 | 0.547262343 | 0.548260449 | 0.549383594 | 0.551146192 | 0.553206382 | 0.555123866 | 0.556416545 | 0.558923335 | 0.561541574 | 0.565776769 | 0.570569357 | 0.575463231 | 0.580388551 | 0.585766096 | 0.591237134 | 0.596501638 | 0.600499554 | 0.603979328 |
| Mongolia | 0.466550118 | 0.472199014 | 0.477586554 | 0.482726727 | 0.488276683 | 0.495006781 | 0.501703718 | 0.508558268 | 0.514793383 | 0.520781189 | 0.526650345 | 0.532620201 | 0.538424835 | 0.543971651 | 0.5495515 | 0.554680284 | 0.559561138 | 0.564236795 | 0.568500599 | 0.571341686 | 0.574406669 | 0.578600663 | 0.583255886 | 0.588080811 | 0.592867353 | 0.596859648 | 0.600260767 | 0.603721965 | 0.607501833 | 0.611250681 | 0.614629637 | 0.617621565 |
| Tajikistan | 0.466155413 | 0.472342173 | 0.475745195 | 0.478238813 | 0.478726011 | 0.477586868 | 0.474346027 | 0.470274796 | 0.465341742 | 0.460505616 | 0.457061333 | 0.456256513 | 0.45880778 | 0.463329706 | 0.469154214 | 0.474637786 | 0.479651831 | 0.484211156 | 0.488441136 | 0.492064041 | 0.495893964 | 0.499869804 | 0.503953885 | 0.507970676 | 0.511989518 | 0.515783245 | 0.519897002 | 0.524492607 | 0.529153916 | 0.533769951 | 0.537690531 | 0.541511187 |
| Turkmenistan | 0.563126887 | 0.564939386 | 0.565587505 | 0.567174449 | 0.567288976 | 0.567198635 | 0.567061261 | 0.565623084 | 0.564405264 | 0.564716006 | 0.567421033 | 0.571043819 | 0.575596166 | 0.580820126 | 0.586712349 | 0.593388895 | 0.599896069 | 0.606042198 | 0.611923309 | 0.617652905 | 0.623234589 | 0.629233722 | 0.635263183 | 0.641361893 | 0.647542417 | 0.65335279 | 0.658945613 | 0.664284692 | 0.669433599 | 0.674303536 | 0.67829818 | 0.682160776 |
| Uzbekistan | 0.500241735 | 0.501544609 | 0.502905194 | 0.506556615 | 0.511018525 | 0.516986264 | 0.52439291 | 0.532686328 | 0.541279069 | 0.549739391 | 0.557522311 | 0.564803383 | 0.571690143 | 0.578006538 | 0.583942986 | 0.589530777 | 0.594498057 | 0.599628055 | 0.604889158 | 0.610351745 | 0.616097907 | 0.621344619 | 0.626202125 | 0.630796403 | 0.63525705 | 0.639805639 | 0.644230597 | 0.648119347 | 0.651918144 | 0.655836959 | 0.659124204 | 0.662621694 |
| Central Europe | 0.637272174 | 0.643082622 | 0.648772145 | 0.654469912 | 0.661219388 | 0.667954947 | 0.674121167 | 0.680037255 | 0.685968374 | 0.692056184 | 0.698779713 | 0.705656783 | 0.712000597 | 0.717823644 | 0.72349425 | 0.728674093 | 0.733476441 | 0.738076675 | 0.743147843 | 0.748765718 | 0.754919495 | 0.760349288 | 0.764803271 | 0.768567712 | 0.771755473 | 0.774844012 | 0.777817247 | 0.78135932 | 0.785476334 | 0.789748976 | 0.793111123 | 0.796244448 |
| Albania | 0.5577733 | 0.555629412 | 0.553107897 | 0.552453328 | 0.553763669 | 0.557040889 | 0.562248886 | 0.566802804 | 0.572534102 | 0.578295462 | 0.584664188 | 0.592412947 | 0.599865978 | 0.606971166 | 0.613864247 | 0.620679615 | 0.627485617 | 0.634167969 | 0.640766134 | 0.64681613 | 0.652654203 | 0.658196773 | 0.66355678 | 0.668739559 | 0.674112985 | 0.679668601 | 0.685036648 | 0.690379199 | 0.695566878 | 0.700287863 | 0.703790245 | 0.706849791 |
| Bosnia and Herzegovina | 0.54113254 | 0.541346251 | 0.539303227 | 0.536031882 | 0.53385339 | 0.534025536 | 0.545549215 | 0.563565415 | 0.581829515 | 0.597997469 | 0.610950685 | 0.621989464 | 0.632075518 | 0.640635102 | 0.647793732 | 0.653859137 | 0.659813662 | 0.665578735 | 0.671100814 | 0.6760329 | 0.680824196 | 0.685399264 | 0.689506566 | 0.693508711 | 0.697031893 | 0.700446047 | 0.704079344 | 0.708053431 | 0.712347302 | 0.716857125 | 0.720202056 | 0.723077893 |
| Bulgaria | 0.633446498 | 0.643069488 | 0.651186876 | 0.659161569 | 0.66743046 | 0.673999977 | 0.678862584 | 0.680221466 | 0.678496877 | 0.677446543 | 0.681004524 | 0.686487366 | 0.691511674 | 0.695565965 | 0.699881951 | 0.704471207 | 0.708868294 | 0.713192402 | 0.71767245 | 0.723676627 | 0.730701737 | 0.735749281 | 0.739177517 | 0.742135948 | 0.745372643 | 0.748979058 | 0.752259199 | 0.755665865 | 0.75914846 | 0.762359791 | 0.765089711 | 0.768150939 |
| Croatia | 0.668906358 | 0.6747246 | 0.678093133 | 0.678221047 | 0.677548133 | 0.677029465 | 0.679507318 | 0.68536501 | 0.691365519 | 0.696636411 | 0.703039899 | 0.709536841 | 0.715383422 | 0.721130377 | 0.726327652 | 0.73215414 | 0.738066879 | 0.743148777 | 0.748469602 | 0.754065605 | 0.759472252 | 0.763686381 | 0.767405336 | 0.77115188 | 0.774479241 | 0.777865888 | 0.781443605 | 0.785074537 | 0.78867141 | 0.792475415 | 0.795462272 | 0.798341027 |
| Czechia | 0.681848021 | 0.688002859 | 0.697577809 | 0.710853416 | 0.725969686 | 0.737981722 | 0.74604138 | 0.751680344 | 0.757033755 | 0.76242888 | 0.767766159 | 0.77216724 | 0.776927934 | 0.782448081 | 0.787126405 | 0.791333948 | 0.794922784 | 0.798254118 | 0.801764846 | 0.804962225 | 0.808747328 | 0.812054784 | 0.814230401 | 0.815615361 | 0.816227257 | 0.816564684 | 0.817153026 | 0.81906768 | 0.822034486 | 0.824784473 | 0.826626631 | 0.828450433 |
| Hungary | 0.649419991 | 0.653707155 | 0.660382905 | 0.667543556 | 0.675051406 | 0.683186412 | 0.690883334 | 0.697935514 | 0.70420272 | 0.709980627 | 0.715805281 | 0.722099055 | 0.728384596 | 0.734450658 | 0.740099975 | 0.745356806 | 0.750515381 | 0.754942942 | 0.759317468 | 0.763563687 | 0.767328649 | 0.769701942 | 0.770784245 | 0.771561698 | 0.772575046 | 0.774047383 | 0.776150305 | 0.779116417 | 0.782083661 | 0.784910309 | 0.78762039 | 0.790754768 |
| Montenegro | 0.67422572 | 0.674305723 | 0.672664772 | 0.668665719 | 0.665011931 | 0.662765437 | 0.663414769 | 0.665542032 | 0.669024771 | 0.672096103 | 0.67730685 | 0.683433454 | 0.689939483 | 0.696517506 | 0.702998276 | 0.709065465 | 0.715459074 | 0.72250394 | 0.729981756 | 0.736538926 | 0.743347873 | 0.750080328 | 0.75557838 | 0.760886907 | 0.765776527 | 0.770561278 | 0.77520077 | 0.779764632 | 0.784408971 | 0.789026903 | 0.792554393 | 0.795800584 |
| North Macedonia | 0.609026094 | 0.611337453 | 0.612708856 | 0.613195099 | 0.614362905 | 0.616994878 | 0.620944017 | 0.626223811 | 0.63205941 | 0.636687259 | 0.641651243 | 0.648049676 | 0.65539879 | 0.663408891 | 0.67123752 | 0.678047273 | 0.684044336 | 0.689729481 | 0.695376955 | 0.700453052 | 0.705589324 | 0.710592521 | 0.715173148 | 0.719671149 | 0.724089589 | 0.728603773 | 0.733173624 | 0.737339955 | 0.74133437 | 0.745193569 | 0.7478499 | 0.750629703 |
| Poland | 0.627227888 | 0.632671471 | 0.640661793 | 0.6489505 | 0.658010514 | 0.666678867 | 0.674378829 | 0.682587612 | 0.690805671 | 0.698536056 | 0.706662052 | 0.714831304 | 0.722009458 | 0.728155254 | 0.733673192 | 0.738428767 | 0.742363709 | 0.746008817 | 0.750521426 | 0.756427378 | 0.763184974 | 0.769407164 | 0.774680767 | 0.779434257 | 0.783682369 | 0.787445343 | 0.790651015 | 0.794530345 | 0.799478982 | 0.804791332 | 0.808795318 | 0.812042809 |
| Romania | 0.619298862 | 0.626795627 | 0.630475082 | 0.633801422 | 0.638371932 | 0.643718879 | 0.648104195 | 0.651225576 | 0.654927769 | 0.659544846 | 0.665294346 | 0.672020167 | 0.678297417 | 0.684074929 | 0.690417055 | 0.69499895 | 0.699490766 | 0.704523727 | 0.710757128 | 0.717445122 | 0.723891349 | 0.729018272 | 0.733892482 | 0.737880473 | 0.740395467 | 0.74347353 | 0.74705871 | 0.751117367 | 0.755533333 | 0.760283971 | 0.764276444 | 0.768453864 |
| Serbia | 0.63051102 | 0.638016934 | 0.641971994 | 0.642096034 | 0.642223221 | 0.642739671 | 0.644846417 | 0.647162094 | 0.650245989 | 0.655534174 | 0.6607528 | 0.665407335 | 0.671511072 | 0.678334929 | 0.686819842 | 0.696097568 | 0.704945018 | 0.713242305 | 0.721505306 | 0.729965596 | 0.740091311 | 0.750072762 | 0.757105082 | 0.762170908 | 0.766043091 | 0.769842223 | 0.773654077 | 0.777460384 | 0.781494974 | 0.7857683 | 0.789183333 | 0.792416294 |
| Slovakia | 0.653853505 | 0.658731402 | 0.665830111 | 0.675967937 | 0.688136477 | 0.697476536 | 0.704405003 | 0.71151321 | 0.719058766 | 0.726144173 | 0.73404624 | 0.741392554 | 0.746791142 | 0.75116727 | 0.755836694 | 0.761724185 | 0.767673036 | 0.772914153 | 0.7771974 | 0.78105226 | 0.786254422 | 0.791465382 | 0.795456216 | 0.797903723 | 0.799618796 | 0.80039418 | 0.80087821 | 0.802277317 | 0.804357302 | 0.806448457 | 0.808305966 | 0.81061053 |
| Slovenia | 0.727463928 | 0.732666986 | 0.737391552 | 0.741774172 | 0.746234861 | 0.750994975 | 0.756348987 | 0.762275292 | 0.767728526 | 0.773236192 | 0.77957801 | 0.786240776 | 0.791805569 | 0.796371531 | 0.80096224 | 0.805601248 | 0.809708595 | 0.813006319 | 0.81634725 | 0.81887856 | 0.821178486 | 0.823023561 | 0.824336563 | 0.825826275 | 0.827799246 | 0.829837071 | 0.831367915 | 0.833038139 | 0.835472481 | 0.838266782 | 0.840373846 | 0.842430731 |
| Eastern Europe | 0.664250165 | 0.671452375 | 0.678752887 | 0.683359195 | 0.686011586 | 0.689459344 | 0.692791353 | 0.695202953 | 0.696939833 | 0.69881365 | 0.70101118 | 0.703506217 | 0.706727141 | 0.71202517 | 0.719215436 | 0.726265709 | 0.732617452 | 0.738786858 | 0.745450424 | 0.751070158 | 0.756606481 | 0.761025305 | 0.76542275 | 0.770338293 | 0.775091704 | 0.779663484 | 0.784804691 | 0.790014867 | 0.794485575 | 0.798138377 | 0.800505061 | 0.802851009 |
| Belarus | 0.622446576 | 0.627038267 | 0.632891737 | 0.639394169 | 0.644652781 | 0.648959504 | 0.652855221 | 0.655627296 | 0.657246467 | 0.66017101 | 0.664705822 | 0.671333269 | 0.678121782 | 0.684868556 | 0.692502135 | 0.698985483 | 0.705119103 | 0.711496562 | 0.719120871 | 0.727292784 | 0.734954626 | 0.739504326 | 0.743468837 | 0.748529323 | 0.75351148 | 0.758205743 | 0.764328222 | 0.771131466 | 0.776568957 | 0.780508019 | 0.782381565 | 0.784484711 |
| Estonia | 0.674967632 | 0.685223089 | 0.695586674 | 0.70369315 | 0.708456181 | 0.713172725 | 0.718813024 | 0.72465244 | 0.729027035 | 0.733250616 | 0.739713154 | 0.746602207 | 0.752629898 | 0.757815678 | 0.763223727 | 0.769112182 | 0.774451842 | 0.781074443 | 0.788598874 | 0.794728213 | 0.800829718 | 0.806633078 | 0.811109358 | 0.814887177 | 0.818770732 | 0.823221278 | 0.82736436 | 0.830975311 | 0.834566296 | 0.838573509 | 0.841817139 | 0.844917787 |
| Latvia | 0.680193638 | 0.688117297 | 0.696157036 | 0.705249855 | 0.712531353 | 0.717639459 | 0.721139538 | 0.72374658 | 0.725427336 | 0.727219685 | 0.730951449 | 0.736294196 | 0.74187044 | 0.748168162 | 0.755263211 | 0.761929433 | 0.768459929 | 0.776481959 | 0.786324573 | 0.794504427 | 0.798171594 | 0.799080776 | 0.801026474 | 0.80266049 | 0.803721486 | 0.806985995 | 0.811463591 | 0.816923118 | 0.821419809 | 0.824781777 | 0.827900448 | 0.830663516 |
| Lithuania | 0.668503938 | 0.673234121 | 0.68099288 | 0.687480621 | 0.691151653 | 0.69473438 | 0.698591301 | 0.70284095 | 0.707468182 | 0.712834171 | 0.71989696 | 0.727133585 | 0.733793398 | 0.741229601 | 0.748949762 | 0.75635236 | 0.763059124 | 0.770229639 | 0.778273712 | 0.785689739 | 0.793364682 | 0.800924994 | 0.807827026 | 0.813219722 | 0.817886376 | 0.823112629 | 0.829303695 | 0.83587174 | 0.84191891 | 0.847906339 | 0.852752388 | 0.856484049 |
| Moldova | 0.604251762 | 0.607270774 | 0.609664558 | 0.61415292 | 0.617603767 | 0.620951321 | 0.623113984 | 0.624370105 | 0.624116761 | 0.622834529 | 0.622004934 | 0.62293641 | 0.625874845 | 0.629985045 | 0.635394822 | 0.641530965 | 0.647996264 | 0.654215851 | 0.661046597 | 0.666284394 | 0.671989616 | 0.678004137 | 0.682979796 | 0.688519175 | 0.694104557 | 0.699355166 | 0.705144542 | 0.711206637 | 0.717244427 | 0.723011648 | 0.72771054 | 0.732214875 |
| Russia | 0.671600578 | 0.680099211 | 0.688068022 | 0.692036194 | 0.694144966 | 0.697854214 | 0.701468231 | 0.703752867 | 0.705198518 | 0.706863538 | 0.708643648 | 0.710570359 | 0.713460957 | 0.718875116 | 0.726305319 | 0.733592777 | 0.740064664 | 0.746191512 | 0.752623243 | 0.757960405 | 0.763320116 | 0.767633588 | 0.771920384 | 0.776526979 | 0.780617524 | 0.784913774 | 0.790330149 | 0.795669484 | 0.800108144 | 0.803658201 | 0.806011474 | 0.808536005 |
| Ukraine | 0.647461466 | 0.651950021 | 0.657600333 | 0.662867405 | 0.665506375 | 0.666950167 | 0.668104744 | 0.669207846 | 0.670322301 | 0.670642905 | 0.671095001 | 0.67246782 | 0.674543221 | 0.678614636 | 0.685380936 | 0.692023301 | 0.698093383 | 0.704202308 | 0.710980184 | 0.716185465 | 0.72101987 | 0.724891957 | 0.7288172 | 0.734033573 | 0.739883557 | 0.743790863 | 0.746857989 | 0.750593324 | 0.754451727 | 0.757980081 | 0.759929872 | 0.760773913 |
| High income | 0.751589646 | 0.755897938 | 0.760450188 | 0.764678286 | 0.768692619 | 0.772362673 | 0.775646213 | 0.778714489 | 0.781567859 | 0.78449535 | 0.78791261 | 0.791477945 | 0.794800548 | 0.797577398 | 0.800086763 | 0.802177445 | 0.804037939 | 0.806419386 | 0.809492671 | 0.812835897 | 0.816526032 | 0.820039001 | 0.823312168 | 0.826445395 | 0.829542032 | 0.832972459 | 0.836547027 | 0.840197943 | 0.84388377 | 0.847532653 | 0.849867902 | 0.85188481 |
| Australasia | 0.731234397 | 0.734915293 | 0.739005622 | 0.743426014 | 0.747726041 | 0.752015072 | 0.756387302 | 0.760990093 | 0.765299502 | 0.769521272 | 0.773791157 | 0.778073921 | 0.782480024 | 0.786285288 | 0.789427625 | 0.791721729 | 0.792856547 | 0.793949581 | 0.796401957 | 0.79993153 | 0.804112332 | 0.808160633 | 0.812463563 | 0.817349223 | 0.821647278 | 0.825611025 | 0.829456874 | 0.83292442 | 0.836618893 | 0.840581577 | 0.843333427 | 0.845514063 |
| Australia | 0.725982519 | 0.729759859 | 0.733901404 | 0.738374743 | 0.742846572 | 0.747426824 | 0.751991736 | 0.756633901 | 0.761133265 | 0.765697506 | 0.770223843 | 0.774662787 | 0.77936094 | 0.783600181 | 0.787016543 | 0.789615521 | 0.791155688 | 0.792532683 | 0.7952929 | 0.799174373 | 0.803561544 | 0.80752022 | 0.811644461 | 0.816453661 | 0.82084085 | 0.824860715 | 0.828559943 | 0.831798266 | 0.835354624 | 0.839317426 | 0.842051314 | 0.844252814 |
| New Zealand | 0.752321655 | 0.755616091 | 0.759461372 | 0.763634604 | 0.767118658 | 0.769998036 | 0.773575283 | 0.778205776 | 0.781730757 | 0.784428638 | 0.787659557 | 0.791426079 | 0.794519591 | 0.796286883 | 0.798177477 | 0.799107294 | 0.798456866 | 0.798447616 | 0.799669514 | 0.801727535 | 0.805133185 | 0.809760352 | 0.814957564 | 0.82014599 | 0.823908944 | 0.827462493 | 0.831843967 | 0.836301795 | 0.840597863 | 0.844533209 | 0.847398737 | 0.849442499 |
| High-income Asia Pacific | 0.767803786 | 0.773332908 | 0.778834454 | 0.783967344 | 0.789028457 | 0.794099242 | 0.798862943 | 0.803127763 | 0.806831345 | 0.810468966 | 0.814089018 | 0.817544764 | 0.821110491 | 0.824635809 | 0.828073761 | 0.831062145 | 0.833766326 | 0.836546108 | 0.839279641 | 0.841770635 | 0.844682036 | 0.847721965 | 0.850693448 | 0.853671415 | 0.856611561 | 0.859602611 | 0.862733183 | 0.86587024 | 0.869072595 | 0.872218835 | 0.874437255 | 0.876767 |
| Brunei | 0.666081917 | 0.671233831 | 0.676702526 | 0.68217544 | 0.687394093 | 0.692471156 | 0.697602233 | 0.702944386 | 0.708630119 | 0.714851237 | 0.721552493 | 0.728535069 | 0.735343916 | 0.741578091 | 0.747311715 | 0.752481793 | 0.757318655 | 0.761643129 | 0.76571426 | 0.769601682 | 0.773219221 | 0.776631893 | 0.780216552 | 0.784017041 | 0.787733289 | 0.791367567 | 0.794980772 | 0.798309936 | 0.801436553 | 0.804499388 | 0.807424091 | 0.810234367 |
| Japan | 0.790253516 | 0.794616189 | 0.799156225 | 0.802972994 | 0.806689483 | 0.810510771 | 0.813870746 | 0.816675782 | 0.819016202 | 0.821066637 | 0.822938999 | 0.824723241 | 0.826936774 | 0.829392223 | 0.832037381 | 0.834288685 | 0.836222264 | 0.83829416 | 0.840301217 | 0.842133664 | 0.844361811 | 0.846750267 | 0.849061489 | 0.851356994 | 0.853692749 | 0.856097499 | 0.858698173 | 0.861393309 | 0.864237137 | 0.867148183 | 0.869106879 | 0.871241813 |
| Aichi | 0.800854834 | 0.805713722 | 0.810525696 | 0.814428732 | 0.818156687 | 0.821991858 | 0.825359323 | 0.827980287 | 0.830020885 | 0.831752637 | 0.833245504 | 0.834907207 | 0.837089612 | 0.8394057 | 0.84194956 | 0.84427199 | 0.846660454 | 0.849204413 | 0.851117043 | 0.852776742 | 0.8546368 | 0.856832713 | 0.859325788 | 0.861948216 | 0.86460482 | 0.867236785 | 0.870037285 | 0.872907383 | 0.875900188 | 0.878893097 | 0.880918649 | 0.883048835 |
| Akita | 0.743856278 | 0.747574745 | 0.751582445 | 0.755273102 | 0.759436294 | 0.764014469 | 0.768510645 | 0.772345859 | 0.775568933 | 0.778279035 | 0.78054579 | 0.782797361 | 0.785418659 | 0.788163622 | 0.790943978 | 0.793140396 | 0.794412518 | 0.795732063 | 0.797174326 | 0.798841506 | 0.800866168 | 0.80326262 | 0.805853631 | 0.808613052 | 0.811547349 | 0.814414598 | 0.817649094 | 0.820981223 | 0.824355311 | 0.827672686 | 0.829844435 | 0.832249015 |
| Aomori | 0.734632276 | 0.738220585 | 0.742503691 | 0.746331387 | 0.750745944 | 0.755390883 | 0.759330321 | 0.762402008 | 0.765051749 | 0.767594613 | 0.769874946 | 0.772279425 | 0.775271085 | 0.778511074 | 0.781878868 | 0.784477083 | 0.786807752 | 0.789067216 | 0.791063776 | 0.793246651 | 0.795891544 | 0.798854467 | 0.801774286 | 0.804342397 | 0.807055783 | 0.809935577 | 0.813181287 | 0.816564418 | 0.820078557 | 0.823525299 | 0.825864102 | 0.828344206 |
| Chiba | 0.788716613 | 0.793422788 | 0.798192042 | 0.80219287 | 0.806285916 | 0.810242865 | 0.813860435 | 0.816764764 | 0.818997279 | 0.820582065 | 0.821901513 | 0.822966863 | 0.824476622 | 0.82641886 | 0.828577649 | 0.830349755 | 0.831727315 | 0.833387723 | 0.835137793 | 0.836735633 | 0.838615275 | 0.840537076 | 0.842359915 | 0.844473984 | 0.846576069 | 0.848633504 | 0.850842454 | 0.853092595 | 0.855449616 | 0.857945831 | 0.859472148 | 0.861292639 |
| Ehime | 0.756008354 | 0.759776393 | 0.764179312 | 0.768344579 | 0.772700377 | 0.777227284 | 0.781698624 | 0.785531592 | 0.789125021 | 0.792193405 | 0.794882045 | 0.797150033 | 0.799482838 | 0.802036205 | 0.804784491 | 0.806635571 | 0.807645383 | 0.808722037 | 0.809900717 | 0.81141894 | 0.813555484 | 0.816154609 | 0.818223753 | 0.820405925 | 0.822770357 | 0.825507133 | 0.828682146 | 0.831957961 | 0.83543283 | 0.83887631 | 0.841139972 | 0.843501706 |
| Fukui | 0.766481771 | 0.770995682 | 0.775919886 | 0.780107783 | 0.784392079 | 0.788687729 | 0.792784195 | 0.796523142 | 0.799770058 | 0.802613732 | 0.80520343 | 0.807426161 | 0.809883399 | 0.812426508 | 0.814961413 | 0.817230546 | 0.819442105 | 0.821978707 | 0.824621794 | 0.82713517 | 0.829913507 | 0.832655508 | 0.834741096 | 0.836677569 | 0.83847613 | 0.840575929 | 0.843117259 | 0.845855461 | 0.848784731 | 0.851844711 | 0.853836068 | 0.856142577 |
| Fukuoka | 0.781940943 | 0.785543342 | 0.789628637 | 0.793282003 | 0.797043517 | 0.800880098 | 0.804436744 | 0.807588525 | 0.810293078 | 0.81268464 | 0.814821048 | 0.816674028 | 0.818966763 | 0.821512481 | 0.824085264 | 0.826073658 | 0.827117032 | 0.828349177 | 0.829698726 | 0.831354981 | 0.833630638 | 0.83594027 | 0.838033717 | 0.839924849 | 0.84181947 | 0.843862128 | 0.846146413 | 0.848605186 | 0.851386883 | 0.854300938 | 0.856232862 | 0.858347709 |
| Fukushima | 0.749067309 | 0.752895552 | 0.757341189 | 0.760912537 | 0.764659856 | 0.768845833 | 0.772560203 | 0.775574524 | 0.777881776 | 0.780010634 | 0.781826466 | 0.783609941 | 0.786462471 | 0.789647785 | 0.793278594 | 0.796251769 | 0.798607642 | 0.800836033 | 0.802851608 | 0.804878402 | 0.807085223 | 0.808942846 | 0.811021414 | 0.813403285 | 0.816369047 | 0.819453568 | 0.823033808 | 0.826997514 | 0.831257741 | 0.835392875 | 0.838162681 | 0.840941474 |
| Gifu | 0.77061016 | 0.775167156 | 0.779921097 | 0.784063798 | 0.787935465 | 0.791899237 | 0.795713773 | 0.798845458 | 0.801413024 | 0.803599335 | 0.8055828 | 0.807572147 | 0.809830537 | 0.812386706 | 0.815010996 | 0.817237048 | 0.819118856 | 0.821056673 | 0.822963341 | 0.824666431 | 0.826780101 | 0.829107299 | 0.831449781 | 0.833618063 | 0.835875885 | 0.838198134 | 0.840758833 | 0.843401284 | 0.8462117 | 0.849135879 | 0.851123481 | 0.85334719 |
| Gunma | 0.772669278 | 0.776841517 | 0.781302495 | 0.78512725 | 0.789110353 | 0.793289401 | 0.796752555 | 0.799746577 | 0.801983844 | 0.803736657 | 0.80504171 | 0.806442849 | 0.80891736 | 0.811968481 | 0.814939707 | 0.817273501 | 0.819116823 | 0.821226067 | 0.823304259 | 0.82547856 | 0.828263558 | 0.831236787 | 0.833996064 | 0.836862714 | 0.839948486 | 0.843152025 | 0.846455341 | 0.849730332 | 0.85313864 | 0.856564041 | 0.858805681 | 0.861111359 |
| Hiroshima | 0.788739945 | 0.792996904 | 0.797721054 | 0.801685783 | 0.805563645 | 0.809687101 | 0.81343513 | 0.816491478 | 0.818883202 | 0.820972318 | 0.822971615 | 0.82467757 | 0.826643347 | 0.828941146 | 0.831364153 | 0.833358806 | 0.834573257 | 0.836047797 | 0.837309959 | 0.838661175 | 0.840539399 | 0.842942703 | 0.845106306 | 0.84740061 | 0.850061099 | 0.852995106 | 0.856092481 | 0.859150066 | 0.86231082 | 0.865491143 | 0.867658745 | 0.869908602 |
| Hokkaidō | 0.766618759 | 0.770545049 | 0.775002204 | 0.778997897 | 0.783027038 | 0.787104355 | 0.790624396 | 0.793472978 | 0.795841261 | 0.797971991 | 0.799858003 | 0.801784083 | 0.804273819 | 0.806973437 | 0.809745877 | 0.811638411 | 0.812678459 | 0.813779245 | 0.814942902 | 0.816263184 | 0.817995299 | 0.819928296 | 0.821889277 | 0.823890768 | 0.82607466 | 0.828414741 | 0.831003381 | 0.83370273 | 0.836540626 | 0.839531731 | 0.841554111 | 0.843743975 |
| Hyōgo | 0.793422254 | 0.797854129 | 0.8025584 | 0.806739758 | 0.810591501 | 0.815021468 | 0.818935508 | 0.821963838 | 0.824275794 | 0.826204856 | 0.827898233 | 0.829190881 | 0.830632651 | 0.832100273 | 0.833806314 | 0.835134117 | 0.836270386 | 0.837522759 | 0.838956997 | 0.840230151 | 0.842299741 | 0.844484633 | 0.846580123 | 0.848782551 | 0.851173819 | 0.853663438 | 0.856257068 | 0.858897482 | 0.86159612 | 0.864347138 | 0.866173398 | 0.868170156 |
| Ibaraki | 0.771923199 | 0.776240273 | 0.780828611 | 0.784864357 | 0.789197702 | 0.7934051 | 0.796815911 | 0.799620314 | 0.80165805 | 0.803334713 | 0.804986677 | 0.806526554 | 0.808860068 | 0.811556249 | 0.814275167 | 0.816436475 | 0.818773359 | 0.821631862 | 0.824385606 | 0.826867064 | 0.829813 | 0.832852317 | 0.83559506 | 0.837937155 | 0.840322018 | 0.842916063 | 0.845842052 | 0.848944244 | 0.852221676 | 0.855478381 | 0.857591997 | 0.85980457 |
| Ishikawa | 0.772257224 | 0.777743697 | 0.783048924 | 0.787478366 | 0.791675216 | 0.796129464 | 0.800359655 | 0.803859554 | 0.807072346 | 0.810199083 | 0.813151689 | 0.815800313 | 0.818380354 | 0.820917503 | 0.823555491 | 0.825732518 | 0.826895375 | 0.828021818 | 0.829270081 | 0.830311719 | 0.831676569 | 0.833157802 | 0.834853923 | 0.836991266 | 0.8393891 | 0.842072063 | 0.845090372 | 0.848178261 | 0.85146605 | 0.854748631 | 0.85697088 | 0.859378938 |
| Iwate | 0.736184911 | 0.740022447 | 0.744750245 | 0.748974983 | 0.753613781 | 0.758465624 | 0.762700621 | 0.766128626 | 0.768943899 | 0.77169128 | 0.774133004 | 0.776069176 | 0.778716479 | 0.781715994 | 0.785098485 | 0.787757256 | 0.789322719 | 0.790993556 | 0.792805016 | 0.794775929 | 0.7970759 | 0.799886347 | 0.80294938 | 0.806206565 | 0.80977659 | 0.813367906 | 0.817362833 | 0.82153995 | 0.825693047 | 0.829618765 | 0.832304292 | 0.834979691 |
| Kagawa | 0.771183472 | 0.776049865 | 0.781312969 | 0.785691208 | 0.789987341 | 0.794422443 | 0.797920663 | 0.800638876 | 0.803051941 | 0.805151605 | 0.807138881 | 0.809248316 | 0.811880914 | 0.81461316 | 0.817596224 | 0.819691544 | 0.8208718 | 0.822169805 | 0.823752161 | 0.825773855 | 0.828077381 | 0.830935912 | 0.833670284 | 0.836035636 | 0.838524183 | 0.841122522 | 0.844132356 | 0.847154981 | 0.85026935 | 0.853403508 | 0.855556651 | 0.85781836 |
| Kagoshima | 0.743332441 | 0.747341162 | 0.751621404 | 0.755297035 | 0.759208018 | 0.763288767 | 0.766955466 | 0.770187831 | 0.773109002 | 0.775891103 | 0.778448597 | 0.780876891 | 0.78373284 | 0.786612027 | 0.78943215 | 0.791649394 | 0.793270964 | 0.79535829 | 0.797372711 | 0.799248694 | 0.801523264 | 0.804015123 | 0.806525734 | 0.808993746 | 0.811572052 | 0.81436806 | 0.817370566 | 0.820384331 | 0.823664608 | 0.827131884 | 0.829539178 | 0.832047307 |
| Kanagawa | 0.817021862 | 0.821082745 | 0.825155986 | 0.828639824 | 0.832064556 | 0.835578487 | 0.839006037 | 0.841826124 | 0.843998149 | 0.845619122 | 0.846948412 | 0.848061667 | 0.849481085 | 0.851245229 | 0.853158522 | 0.854831089 | 0.856596079 | 0.858641495 | 0.860618051 | 0.861910898 | 0.8636736 | 0.865506836 | 0.867136143 | 0.86864031 | 0.870016062 | 0.871613074 | 0.873389861 | 0.875300788 | 0.877422553 | 0.879676716 | 0.881030376 | 0.882654391 |
| Kōchi | 0.74200445 | 0.745306112 | 0.749418836 | 0.753381034 | 0.757898646 | 0.762671737 | 0.767032245 | 0.770866113 | 0.774272806 | 0.77745194 | 0.780453108 | 0.783419671 | 0.786654001 | 0.789619151 | 0.792218783 | 0.794048566 | 0.795413452 | 0.79680627 | 0.798412578 | 0.800232141 | 0.802575474 | 0.805249451 | 0.807744715 | 0.810306364 | 0.81302166 | 0.815950174 | 0.819251889 | 0.822724764 | 0.826327764 | 0.829899245 | 0.832363996 | 0.834906164 |
| Kumamoto | 0.753731159 | 0.757620049 | 0.761919289 | 0.765363874 | 0.768826777 | 0.77248233 | 0.775352054 | 0.777632614 | 0.779395773 | 0.781053455 | 0.782930293 | 0.785062117 | 0.787830395 | 0.790916046 | 0.793987945 | 0.796321537 | 0.797541439 | 0.79891207 | 0.800415573 | 0.801898142 | 0.803963031 | 0.806508758 | 0.80927457 | 0.812085578 | 0.814939409 | 0.8176735 | 0.820663179 | 0.823647274 | 0.826778944 | 0.8300365 | 0.83222153 | 0.834536592 |
| Kyōto | 0.799903927 | 0.804110731 | 0.808471829 | 0.812385279 | 0.816066282 | 0.8196826 | 0.8229264 | 0.825615659 | 0.828035273 | 0.83024396 | 0.83257268 | 0.834463406 | 0.836608769 | 0.839026293 | 0.8416575 | 0.843957444 | 0.845700386 | 0.847526443 | 0.849082564 | 0.850453959 | 0.852274417 | 0.854135125 | 0.855908206 | 0.857629263 | 0.859653196 | 0.862061252 | 0.864524259 | 0.866987862 | 0.869571155 | 0.8722428 | 0.874040973 | 0.87601835 |
| Mie | 0.763830036 | 0.768821888 | 0.77394322 | 0.778119696 | 0.782166127 | 0.786285717 | 0.790482619 | 0.793990014 | 0.79686977 | 0.799521316 | 0.80211902 | 0.804648701 | 0.807645892 | 0.810795466 | 0.814218604 | 0.817118578 | 0.819461883 | 0.821950685 | 0.823904353 | 0.825998921 | 0.828617075 | 0.831144169 | 0.833782702 | 0.83666254 | 0.83962612 | 0.842732497 | 0.846016971 | 0.849243622 | 0.852536083 | 0.855820092 | 0.858124725 | 0.860561189 |
| Miyagi | 0.77250193 | 0.777005693 | 0.781843374 | 0.785881585 | 0.789943637 | 0.793938841 | 0.797152556 | 0.799749629 | 0.801736292 | 0.80353098 | 0.805234417 | 0.806956712 | 0.809279907 | 0.811994682 | 0.814898734 | 0.817389532 | 0.81927682 | 0.820947724 | 0.822575613 | 0.824209992 | 0.826185496 | 0.828424497 | 0.831392881 | 0.834525773 | 0.83789656 | 0.841345741 | 0.844973452 | 0.848541346 | 0.851996092 | 0.855356427 | 0.857591139 | 0.859886569 |
| Miyazaki | 0.73910863 | 0.742497639 | 0.74629663 | 0.749475469 | 0.752936057 | 0.75675515 | 0.760702676 | 0.764435684 | 0.767829809 | 0.77098375 | 0.773614482 | 0.776046647 | 0.779084253 | 0.782228869 | 0.785355993 | 0.787456398 | 0.788237145 | 0.789397373 | 0.790877995 | 0.792549616 | 0.794610967 | 0.797205404 | 0.799906343 | 0.80274632 | 0.805668001 | 0.808544779 | 0.811623266 | 0.814663519 | 0.817943559 | 0.821461354 | 0.823973916 | 0.826589859 |
| Nagano | 0.776599842 | 0.780650743 | 0.784828945 | 0.78830185 | 0.791792619 | 0.795782568 | 0.799540002 | 0.802747372 | 0.805406739 | 0.80787798 | 0.810264685 | 0.812343076 | 0.814486841 | 0.816792496 | 0.81933682 | 0.821600202 | 0.823513017 | 0.825581672 | 0.827507061 | 0.829241484 | 0.831310533 | 0.833518264 | 0.835395137 | 0.837240938 | 0.839328961 | 0.841860629 | 0.844714695 | 0.847680549 | 0.850781591 | 0.853968109 | 0.856191465 | 0.858577839 |
| Nagasaki | 0.741965825 | 0.745796116 | 0.750451731 | 0.754563164 | 0.758831053 | 0.763247037 | 0.766907912 | 0.769883826 | 0.772443732 | 0.774924835 | 0.77718146 | 0.779252501 | 0.781663368 | 0.784149042 | 0.786819696 | 0.789096258 | 0.790253758 | 0.791470351 | 0.792807501 | 0.794467475 | 0.796867803 | 0.799781173 | 0.802657287 | 0.805336368 | 0.807846997 | 0.810748834 | 0.813962687 | 0.81740776 | 0.82095367 | 0.824391507 | 0.826743051 | 0.829213881 |
| Nara | 0.77849492 | 0.783115286 | 0.78771718 | 0.79188812 | 0.796021231 | 0.800103269 | 0.804190177 | 0.807484441 | 0.810068329 | 0.812418526 | 0.814657216 | 0.81672094 | 0.818924779 | 0.821147271 | 0.8233192 | 0.825028283 | 0.826074492 | 0.827192689 | 0.828461896 | 0.829589408 | 0.831279129 | 0.83279357 | 0.834362462 | 0.83615687 | 0.837949321 | 0.839736968 | 0.841733308 | 0.843786321 | 0.846060158 | 0.848447659 | 0.849935197 | 0.851652195 |
| Niigata | 0.755942624 | 0.760144111 | 0.764757048 | 0.768690995 | 0.772862924 | 0.77731692 | 0.781102782 | 0.784394068 | 0.787313486 | 0.790064429 | 0.792701067 | 0.795364716 | 0.798377676 | 0.801361451 | 0.804422384 | 0.806976121 | 0.808560162 | 0.810278614 | 0.811947308 | 0.813666444 | 0.815958032 | 0.81846309 | 0.821091673 | 0.823871725 | 0.826600242 | 0.829189367 | 0.831936266 | 0.834766849 | 0.837691588 | 0.840740483 | 0.842852952 | 0.845152095 |
| Ōita | 0.763945301 | 0.768203854 | 0.772857222 | 0.776865072 | 0.780927437 | 0.785177869 | 0.788782354 | 0.792177023 | 0.794961335 | 0.797469451 | 0.80007512 | 0.802454692 | 0.805363631 | 0.808583745 | 0.811737142 | 0.813985626 | 0.815285128 | 0.816504976 | 0.817803259 | 0.818935756 | 0.820888873 | 0.823309697 | 0.825615402 | 0.827698241 | 0.829941553 | 0.832394373 | 0.835229299 | 0.838265302 | 0.841453896 | 0.844711413 | 0.846938835 | 0.849282438 |
| Okayama | 0.777378183 | 0.781781398 | 0.786620382 | 0.790512314 | 0.794237375 | 0.798187391 | 0.801324812 | 0.803913327 | 0.806408327 | 0.808773094 | 0.810684948 | 0.812815147 | 0.815473844 | 0.818186298 | 0.820986706 | 0.823435967 | 0.825738698 | 0.828099225 | 0.830246724 | 0.832233101 | 0.834469523 | 0.83699498 | 0.839234841 | 0.841506668 | 0.843771616 | 0.846279741 | 0.848967874 | 0.851820621 | 0.854897006 | 0.858073993 | 0.860271557 | 0.862560219 |
| Okinawa | 0.735453216 | 0.739066472 | 0.743244 | 0.746807885 | 0.75059463 | 0.754516933 | 0.757191367 | 0.759079599 | 0.760650243 | 0.762281886 | 0.764017248 | 0.766447751 | 0.770068471 | 0.773787589 | 0.777663138 | 0.780784583 | 0.782797085 | 0.784471535 | 0.786144724 | 0.788188942 | 0.790322895 | 0.792593812 | 0.794622106 | 0.796847437 | 0.799504764 | 0.802309239 | 0.805536252 | 0.809062993 | 0.812862855 | 0.816697317 | 0.819223205 | 0.821803051 |
| Ōsaka | 0.804615792 | 0.809253996 | 0.814292929 | 0.818462434 | 0.822327463 | 0.826243329 | 0.829397081 | 0.831927222 | 0.834077574 | 0.835937911 | 0.837433401 | 0.838747315 | 0.840377203 | 0.842145411 | 0.844113305 | 0.845678547 | 0.846828264 | 0.848105777 | 0.849459599 | 0.850775739 | 0.852583573 | 0.854675296 | 0.856660147 | 0.85852076 | 0.860513588 | 0.862604586 | 0.864902704 | 0.867341395 | 0.869936008 | 0.8726364 | 0.874434185 | 0.87640925 |
| Saga | 0.75102028 | 0.754552146 | 0.758988687 | 0.763078633 | 0.767195485 | 0.771356713 | 0.774823474 | 0.777808464 | 0.780496425 | 0.783006033 | 0.785231708 | 0.787492154 | 0.790478224 | 0.793627053 | 0.796788131 | 0.799255142 | 0.800529497 | 0.802161972 | 0.803911309 | 0.805411665 | 0.807489315 | 0.809549957 | 0.811367665 | 0.813173673 | 0.815230927 | 0.817602748 | 0.820355282 | 0.823361971 | 0.826683869 | 0.83011189 | 0.832455576 | 0.834925899 |
| Saitama | 0.782473382 | 0.787026452 | 0.7919327 | 0.796062996 | 0.799950232 | 0.803740545 | 0.807022733 | 0.809773681 | 0.811852464 | 0.813468055 | 0.814842005 | 0.816047693 | 0.817675029 | 0.819751783 | 0.822182819 | 0.824367649 | 0.82602183 | 0.82767181 | 0.82943717 | 0.831084217 | 0.833090516 | 0.835249104 | 0.837197155 | 0.839248663 | 0.841282809 | 0.843350546 | 0.845603624 | 0.847958105 | 0.850461024 | 0.853007085 | 0.854594527 | 0.85637105 |
| Shiga | 0.792940745 | 0.798110131 | 0.80365653 | 0.808228355 | 0.812630828 | 0.816938757 | 0.820598571 | 0.823777408 | 0.826135886 | 0.828136198 | 0.829863692 | 0.831564272 | 0.834279033 | 0.837205083 | 0.840085544 | 0.842213465 | 0.843515438 | 0.84523639 | 0.846893081 | 0.848390553 | 0.850334822 | 0.852203572 | 0.854179806 | 0.856217905 | 0.858271854 | 0.860424971 | 0.862717276 | 0.865366727 | 0.868087684 | 0.870741428 | 0.872422839 | 0.874323368 |
| Shimane | 0.739553491 | 0.743602718 | 0.748040781 | 0.751888094 | 0.756163362 | 0.760864658 | 0.765509795 | 0.769777421 | 0.773586608 | 0.776878236 | 0.7796426 | 0.782327312 | 0.785563933 | 0.789111128 | 0.79279072 | 0.795524837 | 0.797480845 | 0.799616396 | 0.801438777 | 0.803065712 | 0.805321815 | 0.808324275 | 0.811489704 | 0.814509811 | 0.817199718 | 0.819955758 | 0.823256683 | 0.826534406 | 0.830029689 | 0.833621104 | 0.836085125 | 0.838672262 |
| Shizuoka | 0.780199834 | 0.784630605 | 0.789107265 | 0.792731175 | 0.796358359 | 0.800079048 | 0.803573974 | 0.806491752 | 0.808828856 | 0.810842339 | 0.812922473 | 0.814770533 | 0.817412214 | 0.820352735 | 0.823382083 | 0.825989141 | 0.82816662 | 0.830388863 | 0.832534333 | 0.83441062 | 0.836666216 | 0.839127543 | 0.841569601 | 0.844128451 | 0.846620653 | 0.849254156 | 0.852159253 | 0.855121969 | 0.85814927 | 0.861201347 | 0.863250756 | 0.865450452 |
| Tochigi | 0.772234384 | 0.776753976 | 0.781631541 | 0.785867332 | 0.789941714 | 0.794094128 | 0.797501856 | 0.800171508 | 0.802235255 | 0.80389371 | 0.805408653 | 0.807151779 | 0.809633605 | 0.812456105 | 0.815567088 | 0.818243669 | 0.820391222 | 0.822630004 | 0.824766727 | 0.827014679 | 0.829701956 | 0.832312553 | 0.834704501 | 0.837131494 | 0.839901112 | 0.843084967 | 0.846434564 | 0.849703273 | 0.853133891 | 0.856527338 | 0.858795379 | 0.861186498 |
| Tokushima | 0.7538988 | 0.758620934 | 0.763702302 | 0.767994545 | 0.772177487 | 0.776639766 | 0.780695318 | 0.784247331 | 0.787488054 | 0.790802494 | 0.793971708 | 0.797258714 | 0.801143463 | 0.805463391 | 0.809635324 | 0.812775192 | 0.815075531 | 0.817124333 | 0.819272353 | 0.821440752 | 0.824230535 | 0.827206815 | 0.829994667 | 0.832757357 | 0.83565784 | 0.838642974 | 0.841951621 | 0.845474429 | 0.849173842 | 0.852723549 | 0.855060563 | 0.85748626 |
| Tōkyō | 0.866280939 | 0.870778848 | 0.875074043 | 0.878657556 | 0.881894869 | 0.885104598 | 0.887853602 | 0.890256549 | 0.892409102 | 0.894271154 | 0.896011699 | 0.897695304 | 0.899484786 | 0.901254645 | 0.903007849 | 0.904519595 | 0.905859577 | 0.907249257 | 0.908795113 | 0.910613377 | 0.912389001 | 0.914245227 | 0.915901243 | 0.917402279 | 0.918882985 | 0.920275888 | 0.921719523 | 0.923100582 | 0.924571878 | 0.926065613 | 0.9274607 | 0.928810109 |
| Tottori | 0.757802249 | 0.761596984 | 0.766011272 | 0.769773137 | 0.773845827 | 0.778242264 | 0.782119283 | 0.785438087 | 0.788207078 | 0.790690858 | 0.7927611 | 0.794763592 | 0.797045297 | 0.799434447 | 0.802000403 | 0.803886239 | 0.805303679 | 0.806679324 | 0.807970557 | 0.809196932 | 0.810384996 | 0.811884483 | 0.81345937 | 0.815162517 | 0.817126781 | 0.819337393 | 0.821839839 | 0.824656271 | 0.827927541 | 0.831309178 | 0.833576472 | 0.835956957 |
| Toyama | 0.776809557 | 0.781820272 | 0.786683387 | 0.790791202 | 0.794681022 | 0.798741596 | 0.802895812 | 0.806275928 | 0.809217162 | 0.811903678 | 0.814595298 | 0.817152056 | 0.819992044 | 0.823170006 | 0.826396892 | 0.829120465 | 0.830974129 | 0.832827695 | 0.834714939 | 0.83622232 | 0.83836985 | 0.840622847 | 0.842672029 | 0.844835451 | 0.847124857 | 0.849541744 | 0.852210744 | 0.854938254 | 0.857850987 | 0.860840668 | 0.862880011 | 0.865181131 |
| Wakayama | 0.753739111 | 0.757745147 | 0.76215474 | 0.766116135 | 0.769938364 | 0.773914914 | 0.777687657 | 0.780840732 | 0.78355184 | 0.786085234 | 0.788608259 | 0.791184093 | 0.794061798 | 0.797106859 | 0.800368643 | 0.803396044 | 0.805908732 | 0.808220465 | 0.810480139 | 0.812550369 | 0.815357274 | 0.818685888 | 0.822005337 | 0.825122793 | 0.828111746 | 0.830917363 | 0.833898196 | 0.836723197 | 0.839654018 | 0.842731874 | 0.844761276 | 0.847037481 |
| Yamagata | 0.746246131 | 0.750074468 | 0.754225012 | 0.757638499 | 0.761162835 | 0.764952999 | 0.768540479 | 0.771656499 | 0.774287768 | 0.776609157 | 0.778635343 | 0.780593138 | 0.783366568 | 0.786464293 | 0.789853806 | 0.792823143 | 0.795371298 | 0.798156114 | 0.800468521 | 0.802631004 | 0.80530324 | 0.808059422 | 0.810785405 | 0.813663335 | 0.816524034 | 0.81960797 | 0.822985563 | 0.826340218 | 0.829759981 | 0.833160261 | 0.835456309 | 0.837933155 |
| Yamaguchi | 0.770512746 | 0.774213385 | 0.778637263 | 0.782122399 | 0.785735172 | 0.789678824 | 0.793129836 | 0.796171204 | 0.798722798 | 0.800975841 | 0.803161598 | 0.805353955 | 0.808280787 | 0.811210207 | 0.81415528 | 0.816559685 | 0.818182261 | 0.820068132 | 0.822086832 | 0.824120996 | 0.826831876 | 0.829798262 | 0.832547214 | 0.83524197 | 0.837772249 | 0.839893531 | 0.842479904 | 0.845387567 | 0.84859169 | 0.851797934 | 0.853962511 | 0.856255885 |
| Yamanashi | 0.777354522 | 0.781500795 | 0.785662771 | 0.78921375 | 0.792929522 | 0.796882169 | 0.800258994 | 0.803108456 | 0.805065438 | 0.806818886 | 0.808638418 | 0.810422395 | 0.812572074 | 0.815050876 | 0.817920438 | 0.820471732 | 0.823198084 | 0.826019186 | 0.828514097 | 0.830194394 | 0.832723126 | 0.835465036 | 0.83777821 | 0.839849449 | 0.841965894 | 0.844199011 | 0.846499725 | 0.848750205 | 0.851177127 | 0.853882078 | 0.85570176 | 0.857782852 |
| South Korea | 0.692329307 | 0.702631128 | 0.712440842 | 0.72228674 | 0.732197906 | 0.74194003 | 0.75155324 | 0.760591683 | 0.768281885 | 0.776773436 | 0.785428821 | 0.793496281 | 0.801024295 | 0.807687786 | 0.813720927 | 0.8190934 | 0.82417982 | 0.829127953 | 0.833894627 | 0.838108331 | 0.842511429 | 0.846913229 | 0.851140263 | 0.855441 | 0.859665568 | 0.863907408 | 0.868317824 | 0.872617764 | 0.876823317 | 0.88073151 | 0.883717569 | 0.886675267 |
| Singapore | 0.686404444 | 0.695055558 | 0.703427834 | 0.712315204 | 0.721256797 | 0.729982231 | 0.739028235 | 0.747571898 | 0.754873204 | 0.760831136 | 0.76771737 | 0.774294657 | 0.77973577 | 0.784165555 | 0.789852774 | 0.796455751 | 0.803342694 | 0.810625304 | 0.818124162 | 0.824251516 | 0.83022216 | 0.833829608 | 0.837391807 | 0.840420178 | 0.842894538 | 0.845401857 | 0.847596999 | 0.849051518 | 0.850762729 | 0.852497338 | 0.85429601 | 0.856097766 |
| High-income North America | 0.765659862 | 0.768049984 | 0.771549819 | 0.774977626 | 0.778206755 | 0.781348237 | 0.784104747 | 0.78634761 | 0.788569028 | 0.791196294 | 0.794997034 | 0.799274822 | 0.802961331 | 0.805821251 | 0.807991191 | 0.808886691 | 0.809721162 | 0.812486106 | 0.817083931 | 0.822470006 | 0.827814138 | 0.832060879 | 0.835668937 | 0.839058685 | 0.842340953 | 0.845876702 | 0.849423368 | 0.852960115 | 0.856316178 | 0.859749397 | 0.861874247 | 0.863465474 |
| Canada | 0.781977864 | 0.78415936 | 0.787030537 | 0.789716593 | 0.792598518 | 0.796521166 | 0.80106497 | 0.804833356 | 0.808135441 | 0.81226594 | 0.816685059 | 0.82069625 | 0.824402272 | 0.827801096 | 0.83092811 | 0.833288294 | 0.83491566 | 0.836625096 | 0.838913685 | 0.841583671 | 0.844853489 | 0.848017833 | 0.850978048 | 0.853844945 | 0.856759934 | 0.859532904 | 0.862174226 | 0.86505383 | 0.867905541 | 0.870554479 | 0.871992631 | 0.87317068 |
| Greenland | 0.732258252 | 0.730626532 | 0.729781103 | 0.729894842 | 0.730875896 | 0.730831638 | 0.730216225 | 0.730551087 | 0.733242588 | 0.73655713 | 0.74085701 | 0.746289605 | 0.752878371 | 0.759444152 | 0.765401567 | 0.77209958 | 0.779141406 | 0.785321922 | 0.791441257 | 0.79702575 | 0.803003131 | 0.808105185 | 0.811684562 | 0.814368308 | 0.81652706 | 0.817719189 | 0.818961902 | 0.819781101 | 0.820815514 | 0.822339996 | 0.824359028 | 0.826210336 |
| USA | 0.76364769 | 0.766044295 | 0.769595492 | 0.773103517 | 0.776368588 | 0.779410254 | 0.78193477 | 0.783979278 | 0.786076332 | 0.788544535 | 0.79231665 | 0.796676793 | 0.800403318 | 0.803228025 | 0.805304692 | 0.80603308 | 0.806783583 | 0.80968805 | 0.814577102 | 0.820294574 | 0.825884658 | 0.830266388 | 0.833958192 | 0.837412988 | 0.840746528 | 0.844370336 | 0.848015521 | 0.851630299 | 0.855049193 | 0.858578065 | 0.860792773 | 0.862448354 |
| Alabama | 0.723971524 | 0.726751726 | 0.731172405 | 0.735422776 | 0.739157219 | 0.742448805 | 0.744779226 | 0.745964171 | 0.747298417 | 0.749782983 | 0.755622209 | 0.763456895 | 0.769676317 | 0.773721933 | 0.775449019 | 0.773795704 | 0.771407284 | 0.772854009 | 0.778972353 | 0.787618454 | 0.795550179 | 0.800866201 | 0.804534897 | 0.807097586 | 0.808851739 | 0.810003813 | 0.811181846 | 0.813588056 | 0.816421842 | 0.820526551 | 0.823776522 | 0.825605142 |
| Alaska | 0.745456697 | 0.745077011 | 0.748832169 | 0.755756001 | 0.762254073 | 0.767586469 | 0.771153384 | 0.773369618 | 0.774814237 | 0.776839421 | 0.780830769 | 0.785219722 | 0.788981903 | 0.792223828 | 0.794916317 | 0.795403346 | 0.794437095 | 0.795554894 | 0.799643985 | 0.804586634 | 0.80994445 | 0.815967748 | 0.820908359 | 0.824571781 | 0.828891472 | 0.834500637 | 0.840154869 | 0.844889335 | 0.848599564 | 0.853232249 | 0.856135719 | 0.857524599 |
| Arizona | 0.736537113 | 0.738789693 | 0.741489192 | 0.743897802 | 0.745861893 | 0.74756578 | 0.749188043 | 0.750627388 | 0.752124723 | 0.753689992 | 0.756360016 | 0.759537356 | 0.762461119 | 0.765014788 | 0.767425903 | 0.769378053 | 0.772647636 | 0.778812867 | 0.787176044 | 0.79577678 | 0.803380787 | 0.80908547 | 0.813563778 | 0.817747791 | 0.821651225 | 0.825647941 | 0.82971346 | 0.834063601 | 0.838216155 | 0.842261777 | 0.844752831 | 0.846732871 |
| Arkansas | 0.702645719 | 0.707897114 | 0.714654406 | 0.718950616 | 0.721219502 | 0.722524935 | 0.724044374 | 0.726629462 | 0.729878243 | 0.733090647 | 0.737482289 | 0.743201655 | 0.747755292 | 0.751535518 | 0.753728785 | 0.75302192 | 0.751467203 | 0.754121526 | 0.760439725 | 0.768575186 | 0.775874779 | 0.781333963 | 0.786037573 | 0.78957499 | 0.792652136 | 0.795565863 | 0.79882242 | 0.802706201 | 0.806352351 | 0.811713421 | 0.815679301 | 0.81770902 |
| California | 0.75549857 | 0.756354327 | 0.759310592 | 0.763141736 | 0.767219317 | 0.771536824 | 0.77579064 | 0.77980965 | 0.78378332 | 0.787586289 | 0.791934864 | 0.796169169 | 0.79984861 | 0.803048533 | 0.805901427 | 0.807962701 | 0.810489716 | 0.814481317 | 0.81964069 | 0.825074259 | 0.830488509 | 0.83516311 | 0.839368226 | 0.843425794 | 0.847381746 | 0.851531457 | 0.855674822 | 0.859917798 | 0.863889589 | 0.867176016 | 0.868979466 | 0.87021505 |
| Colorado | 0.783966537 | 0.784800899 | 0.788062706 | 0.792444753 | 0.7964655 | 0.799673811 | 0.801839991 | 0.802452207 | 0.80204853 | 0.801964458 | 0.80418719 | 0.80819566 | 0.812791253 | 0.816945265 | 0.81988706 | 0.821091489 | 0.821714932 | 0.823989861 | 0.828337824 | 0.834138261 | 0.840361488 | 0.845140761 | 0.8485599 | 0.851510371 | 0.854964695 | 0.860413867 | 0.865889342 | 0.870244272 | 0.873306822 | 0.874750588 | 0.875278 | 0.876654771 |
| Connecticut | 0.824312178 | 0.827833018 | 0.831569583 | 0.83447056 | 0.836957596 | 0.839146647 | 0.840550575 | 0.841612081 | 0.843664579 | 0.847149752 | 0.851754248 | 0.856697188 | 0.860699188 | 0.862567469 | 0.863343742 | 0.86325105 | 0.863409623 | 0.864951825 | 0.868111319 | 0.872325503 | 0.876428127 | 0.879692601 | 0.882652249 | 0.885677278 | 0.888741467 | 0.89176531 | 0.89437426 | 0.896433869 | 0.898250057 | 0.900422117 | 0.902144773 | 0.903107563 |
| Delaware | 0.783519768 | 0.786707633 | 0.790250993 | 0.793239859 | 0.795656336 | 0.797818794 | 0.799505987 | 0.800487658 | 0.801527954 | 0.803179514 | 0.806280354 | 0.810038877 | 0.813174598 | 0.815527567 | 0.817446441 | 0.818455176 | 0.819924189 | 0.822989054 | 0.827413281 | 0.832627376 | 0.837710456 | 0.842082817 | 0.845544447 | 0.848508739 | 0.85080132 | 0.852944753 | 0.855133015 | 0.857616616 | 0.860229422 | 0.863076532 | 0.864801832 | 0.866366234 |
| Washington, DC | 0.785119619 | 0.788995157 | 0.794617144 | 0.801002677 | 0.807851272 | 0.814989188 | 0.821998365 | 0.828325838 | 0.834034202 | 0.839474011 | 0.84538819 | 0.851353626 | 0.856483378 | 0.859521738 | 0.862004052 | 0.863849201 | 0.865843899 | 0.868749885 | 0.872355189 | 0.876299235 | 0.880192679 | 0.883688737 | 0.886672124 | 0.889317883 | 0.891765521 | 0.894242602 | 0.896844752 | 0.899434958 | 0.901606736 | 0.903467775 | 0.904873973 | 0.905964755 |
| Florida | 0.755299508 | 0.759577357 | 0.764214506 | 0.768635499 | 0.772756742 | 0.776116633 | 0.77817577 | 0.779535058 | 0.781221493 | 0.783279839 | 0.787066181 | 0.792341558 | 0.796594298 | 0.798747015 | 0.799664028 | 0.799508523 | 0.800160462 | 0.805009003 | 0.81281767 | 0.820855087 | 0.827521372 | 0.831942512 | 0.835180664 | 0.837648764 | 0.83990453 | 0.842418769 | 0.84525239 | 0.848950379 | 0.85290197 | 0.857354268 | 0.860284346 | 0.861979282 |
| Georgia | 0.739511622 | 0.743490724 | 0.748286271 | 0.752772815 | 0.756508865 | 0.75943308 | 0.761496988 | 0.76281708 | 0.764068933 | 0.765608241 | 0.768348485 | 0.771841185 | 0.774881297 | 0.777196817 | 0.779001306 | 0.779955806 | 0.781722097 | 0.785899502 | 0.792041569 | 0.799080124 | 0.805881596 | 0.81150306 | 0.815982727 | 0.82006035 | 0.823730315 | 0.827363984 | 0.83101053 | 0.834853877 | 0.838524146 | 0.842234949 | 0.844594925 | 0.84656427 |
| Hawaii | 0.765363789 | 0.767370787 | 0.770565923 | 0.774531808 | 0.780201178 | 0.785663235 | 0.790617446 | 0.794722364 | 0.795831504 | 0.797273841 | 0.800522109 | 0.804191426 | 0.807175079 | 0.810108752 | 0.813562653 | 0.814896319 | 0.814177197 | 0.815945752 | 0.820453916 | 0.825436041 | 0.829680693 | 0.834030662 | 0.83817902 | 0.842520301 | 0.846419717 | 0.849503358 | 0.85219162 | 0.855447075 | 0.859154213 | 0.864222524 | 0.867878093 | 0.869546917 |
| Idaho | 0.736462409 | 0.73732233 | 0.740411992 | 0.744371806 | 0.74781858 | 0.751005264 | 0.754557185 | 0.757603191 | 0.760381495 | 0.763630894 | 0.767696599 | 0.771877982 | 0.77524921 | 0.777505018 | 0.778962013 | 0.778860968 | 0.778740135 | 0.781354308 | 0.786880605 | 0.793827606 | 0.800526207 | 0.805246716 | 0.808619844 | 0.811684128 | 0.814248203 | 0.817110093 | 0.820531288 | 0.824658803 | 0.828597355 | 0.832173054 | 0.834427424 | 0.83651792 |
| Illinois | 0.769244886 | 0.771253878 | 0.774669165 | 0.778294843 | 0.782376993 | 0.787158475 | 0.791538331 | 0.79467225 | 0.797249579 | 0.799893083 | 0.804211242 | 0.809997491 | 0.815507949 | 0.819692548 | 0.822701196 | 0.823631161 | 0.823805065 | 0.826435047 | 0.831320474 | 0.836978935 | 0.842506713 | 0.846647278 | 0.850139945 | 0.853400744 | 0.856245133 | 0.859278259 | 0.862379074 | 0.865936648 | 0.870034366 | 0.874668099 | 0.877587649 | 0.879187797 |
| Indiana | 0.750328787 | 0.752783713 | 0.756627315 | 0.760335613 | 0.763370222 | 0.765609249 | 0.767485194 | 0.768930907 | 0.77055891 | 0.77291208 | 0.777615379 | 0.783099173 | 0.786753084 | 0.788334508 | 0.788951325 | 0.787800418 | 0.786614138 | 0.78833222 | 0.793265546 | 0.799953918 | 0.806321908 | 0.810265795 | 0.813070241 | 0.815936459 | 0.819175917 | 0.823468702 | 0.827498753 | 0.830688573 | 0.83390058 | 0.83808164 | 0.841042847 | 0.842859834 |
| Iowa | 0.77326663 | 0.775424525 | 0.778950883 | 0.782341469 | 0.78534145 | 0.787707685 | 0.790466653 | 0.793014723 | 0.795066897 | 0.797782149 | 0.801968911 | 0.806587816 | 0.809775906 | 0.811213846 | 0.811295202 | 0.809947208 | 0.809085447 | 0.811196635 | 0.815936752 | 0.82209863 | 0.828055972 | 0.832204587 | 0.835128851 | 0.837958475 | 0.841207977 | 0.846025673 | 0.851183221 | 0.85520558 | 0.858202676 | 0.861065039 | 0.862740604 | 0.86405652 |
| Kansas | 0.765782681 | 0.767582978 | 0.770095478 | 0.773377613 | 0.776860843 | 0.780048096 | 0.781992392 | 0.782827972 | 0.783895322 | 0.78541963 | 0.789389781 | 0.794104243 | 0.797568743 | 0.799959348 | 0.800655499 | 0.799614788 | 0.798502182 | 0.799285816 | 0.802711507 | 0.808299516 | 0.81486303 | 0.820157473 | 0.82515781 | 0.829922405 | 0.833681897 | 0.839090813 | 0.844895672 | 0.848823836 | 0.85189158 | 0.855685461 | 0.858113245 | 0.859383805 |
| Kentucky | 0.721698825 | 0.72457641 | 0.729310325 | 0.733694156 | 0.737528023 | 0.740997266 | 0.743590395 | 0.745736402 | 0.747721967 | 0.749786034 | 0.754208329 | 0.760002314 | 0.765089398 | 0.769024348 | 0.770757301 | 0.769299981 | 0.766895021 | 0.76742391 | 0.771709954 | 0.778281829 | 0.784585994 | 0.788588415 | 0.791720608 | 0.795024818 | 0.798210068 | 0.801617682 | 0.804878659 | 0.808372169 | 0.81203222 | 0.816524046 | 0.819471019 | 0.821325377 |
| Louisiana | 0.717356733 | 0.718644553 | 0.722292552 | 0.727389573 | 0.733052482 | 0.73756337 | 0.739839768 | 0.740648793 | 0.742007099 | 0.744576037 | 0.749806826 | 0.75602604 | 0.761097619 | 0.765532549 | 0.770636699 | 0.772229792 | 0.77020122 | 0.771286536 | 0.776068063 | 0.782616789 | 0.789357791 | 0.793482969 | 0.796137552 | 0.798684495 | 0.801748004 | 0.806551913 | 0.811533751 | 0.815665977 | 0.8189813 | 0.822020131 | 0.823862164 | 0.825346457 |
| Maine | 0.76873693 | 0.774261462 | 0.780753171 | 0.786406603 | 0.790840643 | 0.794018772 | 0.795956749 | 0.796972597 | 0.798548665 | 0.801118613 | 0.804783142 | 0.809198617 | 0.813195477 | 0.816153774 | 0.818171863 | 0.818579628 | 0.818955716 | 0.821310267 | 0.825516188 | 0.830548525 | 0.835627343 | 0.839537257 | 0.842417833 | 0.844874643 | 0.847238428 | 0.849459797 | 0.852060838 | 0.855397357 | 0.859088149 | 0.862540157 | 0.864291992 | 0.865754199 |
| Maryland | 0.796023679 | 0.800123865 | 0.805640343 | 0.810165128 | 0.813717476 | 0.816936095 | 0.819304762 | 0.820462715 | 0.821113793 | 0.822317266 | 0.825712918 | 0.830775476 | 0.83635915 | 0.841266491 | 0.844351981 | 0.844774332 | 0.844526008 | 0.846193513 | 0.850228952 | 0.855757815 | 0.86155986 | 0.866240885 | 0.870180572 | 0.873221707 | 0.875514199 | 0.877500816 | 0.879287478 | 0.881350145 | 0.883315999 | 0.885907515 | 0.888170102 | 0.889225569 |
| Massachusetts | 0.825701409 | 0.829794176 | 0.833579189 | 0.83688148 | 0.840107349 | 0.843307106 | 0.845791623 | 0.847710767 | 0.850036784 | 0.853151393 | 0.857383433 | 0.861876395 | 0.865742159 | 0.868623505 | 0.870689586 | 0.871460873 | 0.872082668 | 0.874074763 | 0.877351918 | 0.880897287 | 0.884341694 | 0.887092654 | 0.889819686 | 0.892349986 | 0.894632983 | 0.897268319 | 0.899790758 | 0.901900621 | 0.903490562 | 0.904741494 | 0.905805675 | 0.906621364 |
| Michigan | 0.766908044 | 0.770918039 | 0.776702532 | 0.781831965 | 0.78626406 | 0.790168022 | 0.792991452 | 0.795174389 | 0.79715201 | 0.799259746 | 0.80337512 | 0.808495105 | 0.812406457 | 0.814777885 | 0.816419216 | 0.816807855 | 0.817025793 | 0.818909755 | 0.822181679 | 0.825728129 | 0.829320047 | 0.832147399 | 0.834594703 | 0.837177662 | 0.840368054 | 0.844647668 | 0.849215154 | 0.853507142 | 0.857198131 | 0.860396019 | 0.862294815 | 0.863971128 |
| Minnesota | 0.799549491 | 0.802800135 | 0.806657992 | 0.809858578 | 0.812711516 | 0.815039875 | 0.816853863 | 0.818606518 | 0.820740585 | 0.823131666 | 0.826307038 | 0.82986937 | 0.833015353 | 0.835605681 | 0.837631771 | 0.838033657 | 0.837957362 | 0.840129733 | 0.844601029 | 0.849986943 | 0.855018051 | 0.858376351 | 0.861164562 | 0.864109277 | 0.86735216 | 0.871179197 | 0.874900003 | 0.878491495 | 0.881532381 | 0.884230985 | 0.885747223 | 0.887013945 |
| Mississippi | 0.697709614 | 0.700217175 | 0.704872701 | 0.709860449 | 0.714793277 | 0.719703472 | 0.722782499 | 0.723481129 | 0.724910651 | 0.727549666 | 0.733009252 | 0.73981595 | 0.744197184 | 0.74613448 | 0.746344361 | 0.742949789 | 0.737859663 | 0.738856293 | 0.747125181 | 0.758398998 | 0.767961978 | 0.774526602 | 0.77966675 | 0.783267014 | 0.786702744 | 0.79102178 | 0.79498021 | 0.798404112 | 0.801547191 | 0.805629824 | 0.808653045 | 0.810471681 |
| Missouri | 0.751221706 | 0.754144786 | 0.759033459 | 0.763528036 | 0.767727876 | 0.770676976 | 0.772616132 | 0.774189081 | 0.775833232 | 0.778570909 | 0.783010744 | 0.787691547 | 0.790817519 | 0.792596495 | 0.793373138 | 0.792329879 | 0.791353315 | 0.793394832 | 0.798977133 | 0.806089233 | 0.812427703 | 0.816665158 | 0.820164887 | 0.823270265 | 0.8261325 | 0.829681845 | 0.833248768 | 0.836116401 | 0.839204061 | 0.843603124 | 0.846540373 | 0.848169136 |
| Montana | 0.756650394 | 0.757937467 | 0.761274633 | 0.765982268 | 0.770667046 | 0.775171489 | 0.779342863 | 0.782092826 | 0.785168355 | 0.788348735 | 0.791659477 | 0.794710948 | 0.797329112 | 0.799482872 | 0.801671683 | 0.802317625 | 0.80216067 | 0.803600984 | 0.807472959 | 0.812863013 | 0.818025958 | 0.821628 | 0.824108694 | 0.826828049 | 0.830792246 | 0.836985022 | 0.843801602 | 0.849350356 | 0.853624655 | 0.856401323 | 0.85767995 | 0.859107705 |
| Nebraska | 0.775521295 | 0.778575121 | 0.782198328 | 0.785008349 | 0.78729054 | 0.790375571 | 0.793533526 | 0.795744941 | 0.797403922 | 0.798826658 | 0.800743844 | 0.802842667 | 0.805472581 | 0.808433836 | 0.810448578 | 0.810490134 | 0.809756434 | 0.811219766 | 0.814655322 | 0.819950893 | 0.826273607 | 0.83120772 | 0.834317036 | 0.837012727 | 0.84065182 | 0.846764168 | 0.852463699 | 0.856302756 | 0.859388561 | 0.862237117 | 0.863916298 | 0.865128654 |
| Nevada | 0.744408991 | 0.747795607 | 0.75322181 | 0.756591811 | 0.758586729 | 0.760947269 | 0.763339269 | 0.764626561 | 0.766305068 | 0.76947594 | 0.774611508 | 0.78005427 | 0.78373812 | 0.785861482 | 0.786562061 | 0.785499897 | 0.785677896 | 0.7907889 | 0.799908513 | 0.809575063 | 0.817422734 | 0.82207684 | 0.825007474 | 0.826968972 | 0.827673453 | 0.827522917 | 0.827838199 | 0.830482907 | 0.835156755 | 0.84159686 | 0.845717344 | 0.847682783 |
| New Hampshire | 0.811328701 | 0.81700015 | 0.821128436 | 0.823177296 | 0.824703999 | 0.826108506 | 0.827137369 | 0.829009623 | 0.832449006 | 0.835975626 | 0.840296149 | 0.845440377 | 0.849705793 | 0.852482529 | 0.854433412 | 0.855162494 | 0.855702971 | 0.857402742 | 0.860650248 | 0.864739468 | 0.86873586 | 0.872428876 | 0.875756144 | 0.878502205 | 0.881402415 | 0.885202504 | 0.888805466 | 0.891793984 | 0.894405373 | 0.896504056 | 0.897425753 | 0.898263681 |
| New Jersey | 0.811232625 | 0.814871765 | 0.81932112 | 0.822530721 | 0.825335771 | 0.828572215 | 0.830945354 | 0.83222171 | 0.833528425 | 0.835453315 | 0.838489011 | 0.842403817 | 0.846604774 | 0.850030773 | 0.852469148 | 0.853304878 | 0.853758732 | 0.855585512 | 0.858967919 | 0.863011633 | 0.866668139 | 0.869786334 | 0.873150081 | 0.876283327 | 0.879024387 | 0.881593394 | 0.88383729 | 0.885532902 | 0.887040829 | 0.889165735 | 0.890959743 | 0.891922443 |
| New Mexico | 0.715668063 | 0.7165368 | 0.719172257 | 0.722824713 | 0.726743025 | 0.730670777 | 0.734164709 | 0.737302352 | 0.740407672 | 0.743575257 | 0.74758474 | 0.751907871 | 0.755535517 | 0.758375149 | 0.760502425 | 0.76140853 | 0.762453079 | 0.76599787 | 0.771945019 | 0.779325853 | 0.786779385 | 0.792889743 | 0.798003906 | 0.802788811 | 0.807200414 | 0.811393411 | 0.815507406 | 0.819642638 | 0.82352103 | 0.827492685 | 0.829941766 | 0.831716579 |
| New York | 0.79741955 | 0.800179201 | 0.803449532 | 0.806504513 | 0.809459648 | 0.812489092 | 0.815392499 | 0.818000166 | 0.820494984 | 0.823397251 | 0.827162079 | 0.831222326 | 0.834862379 | 0.837950891 | 0.840485797 | 0.842078423 | 0.84362318 | 0.846239571 | 0.849441056 | 0.853092358 | 0.856993764 | 0.860488239 | 0.863829479 | 0.867210264 | 0.870378869 | 0.873179675 | 0.875676469 | 0.877966161 | 0.880036383 | 0.882226992 | 0.883987685 | 0.885176868 |
| North Carolina | 0.743977619 | 0.747181909 | 0.75142845 | 0.755446059 | 0.758702636 | 0.761241318 | 0.763040923 | 0.764300516 | 0.765614356 | 0.767476174 | 0.770775805 | 0.774807758 | 0.778305547 | 0.781032612 | 0.78319075 | 0.784228711 | 0.785569826 | 0.789137441 | 0.794720808 | 0.80127194 | 0.807648675 | 0.812742601 | 0.816978511 | 0.820581135 | 0.823708854 | 0.826845778 | 0.830094436 | 0.833460429 | 0.836790068 | 0.84032264 | 0.842589912 | 0.844445313 |
| North Dakota | 0.771430284 | 0.774178767 | 0.777204987 | 0.780101317 | 0.783372445 | 0.786797633 | 0.790927725 | 0.794867929 | 0.799168564 | 0.802966297 | 0.806593183 | 0.809255878 | 0.810890445 | 0.812040486 | 0.812488543 | 0.812432704 | 0.812480851 | 0.814358846 | 0.817757018 | 0.821289367 | 0.825292989 | 0.829075667 | 0.833702585 | 0.839189609 | 0.845720318 | 0.852727229 | 0.858731519 | 0.863328779 | 0.866777718 | 0.869464107 | 0.871463395 | 0.873031775 |
| Ohio | 0.761347777 | 0.763467823 | 0.766720356 | 0.769983371 | 0.773023773 | 0.775826089 | 0.778193383 | 0.780258958 | 0.782408296 | 0.784924235 | 0.788604203 | 0.792630712 | 0.796035917 | 0.798706795 | 0.80065882 | 0.801308907 | 0.801902949 | 0.804081545 | 0.807770775 | 0.812388082 | 0.817084761 | 0.820869548 | 0.824007326 | 0.827149387 | 0.830155177 | 0.833271169 | 0.836558719 | 0.839983014 | 0.843190007 | 0.84650352 | 0.848644339 | 0.850513434 |
| Oklahoma | 0.733039141 | 0.734196414 | 0.736747722 | 0.739537192 | 0.741859464 | 0.743721209 | 0.744911312 | 0.745878648 | 0.747298975 | 0.749183419 | 0.752474519 | 0.756428757 | 0.760126386 | 0.763301691 | 0.76566904 | 0.766453061 | 0.767208796 | 0.769951411 | 0.774975922 | 0.780747615 | 0.786766285 | 0.791750172 | 0.796232194 | 0.801028247 | 0.805692041 | 0.809921023 | 0.813959891 | 0.817797623 | 0.821134229 | 0.824609706 | 0.826857314 | 0.828663242 |
| Oregon | 0.769007728 | 0.771098606 | 0.774222179 | 0.777396013 | 0.780262258 | 0.783117654 | 0.786069235 | 0.789017842 | 0.79214028 | 0.795603641 | 0.800041494 | 0.804545587 | 0.808362446 | 0.811477655 | 0.81383084 | 0.81497578 | 0.81617291 | 0.818904233 | 0.823137953 | 0.82804832 | 0.8328378 | 0.836719812 | 0.840018664 | 0.843339006 | 0.846882733 | 0.850941122 | 0.855219503 | 0.859297789 | 0.862948282 | 0.866310501 | 0.868364592 | 0.870177689 |
| Pennsylvania | 0.781295559 | 0.784392033 | 0.788268353 | 0.792143491 | 0.795839015 | 0.799306425 | 0.802375058 | 0.804980941 | 0.807457013 | 0.810210115 | 0.813759276 | 0.817346307 | 0.820408613 | 0.82292176 | 0.824973395 | 0.826046573 | 0.827064273 | 0.82941159 | 0.832861985 | 0.836945566 | 0.841251322 | 0.844934104 | 0.848201399 | 0.85128821 | 0.854085978 | 0.856931135 | 0.859828665 | 0.862811861 | 0.865774081 | 0.868886274 | 0.87079815 | 0.872416752 |
| Rhode Island | 0.795258398 | 0.798390144 | 0.801855402 | 0.805080215 | 0.807904471 | 0.810442142 | 0.812506454 | 0.814513325 | 0.816822887 | 0.819318879 | 0.822331855 | 0.825599261 | 0.828790297 | 0.831784568 | 0.834606193 | 0.836633586 | 0.838685944 | 0.841862129 | 0.845850461 | 0.85014421 | 0.854680847 | 0.858480813 | 0.861809635 | 0.864827599 | 0.867521022 | 0.870254439 | 0.872964884 | 0.875666776 | 0.878360458 | 0.880992167 | 0.882471147 | 0.883893485 |
| South Carolina | 0.731363176 | 0.735334588 | 0.740915117 | 0.746431495 | 0.751163651 | 0.754990851 | 0.757648952 | 0.759363511 | 0.760937156 | 0.763031619 | 0.766526142 | 0.770765552 | 0.774503246 | 0.777292728 | 0.779207295 | 0.779607388 | 0.780104065 | 0.782910558 | 0.788009136 | 0.794432539 | 0.800910415 | 0.806182063 | 0.810480957 | 0.814246382 | 0.817508098 | 0.820668565 | 0.823885917 | 0.827142014 | 0.830279075 | 0.833612925 | 0.835806254 | 0.83771865 |
| South Dakota | 0.751220476 | 0.753878059 | 0.757773382 | 0.762220786 | 0.766643094 | 0.770649708 | 0.77477888 | 0.77838801 | 0.781724628 | 0.784752668 | 0.78803856 | 0.790771229 | 0.792338924 | 0.793450229 | 0.794231747 | 0.794218482 | 0.794357734 | 0.796857977 | 0.801127175 | 0.806159252 | 0.811311939 | 0.815537404 | 0.819136289 | 0.823215131 | 0.827704286 | 0.833016133 | 0.838669077 | 0.843754632 | 0.84799196 | 0.851839736 | 0.854053827 | 0.855720868 |
| Tennessee | 0.73014798 | 0.732744426 | 0.736784404 | 0.740865341 | 0.74467146 | 0.748223265 | 0.751039118 | 0.753174397 | 0.755385064 | 0.75781867 | 0.761073113 | 0.764320771 | 0.766492124 | 0.767616579 | 0.768073237 | 0.767538474 | 0.767671024 | 0.770573447 | 0.775976647 | 0.782740853 | 0.789257597 | 0.794240586 | 0.798369958 | 0.802482236 | 0.806651253 | 0.811496038 | 0.816472333 | 0.820852394 | 0.82464378 | 0.828414504 | 0.830911729 | 0.833033239 |
| Texas | 0.728266043 | 0.729424929 | 0.731642933 | 0.733962991 | 0.736074692 | 0.738228462 | 0.740187744 | 0.741934487 | 0.743758711 | 0.745750934 | 0.749034838 | 0.752995721 | 0.756436178 | 0.759118112 | 0.761172208 | 0.762034184 | 0.763265498 | 0.766925947 | 0.772872036 | 0.779705582 | 0.786698439 | 0.792425847 | 0.79736383 | 0.802334011 | 0.807471581 | 0.81284725 | 0.818012074 | 0.822857899 | 0.826873539 | 0.830586686 | 0.832882686 | 0.834783278 |
| Utah | 0.744547789 | 0.747423518 | 0.75088115 | 0.754103143 | 0.756690593 | 0.759085491 | 0.761724198 | 0.764769513 | 0.768070193 | 0.771590884 | 0.775903528 | 0.780096634 | 0.78341518 | 0.785835951 | 0.787422594 | 0.787949727 | 0.7888842 | 0.792358526 | 0.798076728 | 0.804807247 | 0.811328069 | 0.816564662 | 0.820788054 | 0.824767847 | 0.828662233 | 0.833072591 | 0.837659888 | 0.842095256 | 0.846219618 | 0.850130104 | 0.852569054 | 0.854616788 |
| Vermont | 0.795613598 | 0.799050645 | 0.802863714 | 0.80640126 | 0.809688005 | 0.812681885 | 0.815343961 | 0.818030649 | 0.821075338 | 0.824411585 | 0.828172219 | 0.83198085 | 0.835513041 | 0.83878558 | 0.841896018 | 0.844014853 | 0.84594287 | 0.848651899 | 0.851971969 | 0.855319187 | 0.858728951 | 0.861890181 | 0.864858756 | 0.867983835 | 0.871252071 | 0.874819639 | 0.878336831 | 0.881587698 | 0.884522352 | 0.887223145 | 0.888677239 | 0.8899999 |
| Virginia | 0.785556276 | 0.788297264 | 0.791798398 | 0.795226744 | 0.798412739 | 0.801287236 | 0.803612093 | 0.805400288 | 0.806999575 | 0.809009858 | 0.812194756 | 0.815900619 | 0.819331879 | 0.822507738 | 0.825396401 | 0.827585424 | 0.829953818 | 0.83381356 | 0.838714689 | 0.844106207 | 0.849388711 | 0.85368094 | 0.857208438 | 0.860242162 | 0.862822636 | 0.865347099 | 0.868029261 | 0.87085374 | 0.873697324 | 0.87680399 | 0.878660604 | 0.880119901 |
| Washington | 0.781101794 | 0.783192157 | 0.786443145 | 0.789887398 | 0.793226943 | 0.796465958 | 0.799622268 | 0.80278059 | 0.806271493 | 0.810109375 | 0.814635753 | 0.818988206 | 0.822602361 | 0.825375051 | 0.827580684 | 0.828453702 | 0.829319568 | 0.831703984 | 0.835316854 | 0.839438851 | 0.84369776 | 0.847318357 | 0.850781789 | 0.854267806 | 0.857750077 | 0.861346597 | 0.86494843 | 0.868573193 | 0.871884538 | 0.874936929 | 0.876491156 | 0.877584845 |
| West Virginia | 0.725935457 | 0.72805707 | 0.7316111 | 0.735513184 | 0.739303585 | 0.742828588 | 0.745902371 | 0.748529084 | 0.750992443 | 0.753469156 | 0.756723408 | 0.759940466 | 0.762297992 | 0.763739376 | 0.764472457 | 0.764040711 | 0.763665988 | 0.764966911 | 0.768011671 | 0.772395493 | 0.777025268 | 0.780692544 | 0.783816863 | 0.787432975 | 0.791469578 | 0.795917586 | 0.800419978 | 0.80464921 | 0.808610815 | 0.812844076 | 0.815637625 | 0.817713511 |
| Wisconsin | 0.784207391 | 0.786860748 | 0.790207111 | 0.793541709 | 0.796764779 | 0.799800129 | 0.802553387 | 0.80497178 | 0.80727955 | 0.809696788 | 0.812823347 | 0.816199508 | 0.81920468 | 0.82161328 | 0.823417104 | 0.824283809 | 0.825150038 | 0.82724699 | 0.830491655 | 0.834526077 | 0.838754514 | 0.842405297 | 0.845668185 | 0.848959525 | 0.852244397 | 0.855673483 | 0.859091933 | 0.86235081 | 0.865378715 | 0.868434573 | 0.870237461 | 0.871726096 |
| Wyoming | 0.750880744 | 0.753215731 | 0.756800183 | 0.76077982 | 0.764501193 | 0.768054327 | 0.771234146 | 0.77449507 | 0.778014938 | 0.781583393 | 0.785653346 | 0.789883005 | 0.793696585 | 0.79716501 | 0.800017544 | 0.801824217 | 0.804022475 | 0.807852352 | 0.813310959 | 0.818741347 | 0.824282071 | 0.828788638 | 0.832510567 | 0.836013441 | 0.839641542 | 0.843439015 | 0.847264337 | 0.851059167 | 0.854693318 | 0.858338343 | 0.860520845 | 0.862148722 |
| Southern Latin America | 0.587308121 | 0.592118694 | 0.598079761 | 0.603588571 | 0.609392016 | 0.614372849 | 0.619260048 | 0.624756487 | 0.630349086 | 0.635748461 | 0.641237434 | 0.646040371 | 0.649825844 | 0.652552403 | 0.655718876 | 0.660519129 | 0.664386241 | 0.6673925 | 0.670688619 | 0.67415239 | 0.678761222 | 0.684134275 | 0.688427908 | 0.691715992 | 0.696039552 | 0.703287229 | 0.710787735 | 0.717548418 | 0.724952157 | 0.731133817 | 0.733963357 | 0.735984717 |
| Argentina | 0.587397284 | 0.59170754 | 0.597996324 | 0.603739312 | 0.60978733 | 0.614473706 | 0.618942624 | 0.623934409 | 0.628723436 | 0.633175129 | 0.638194472 | 0.642175705 | 0.644472507 | 0.645906577 | 0.648296828 | 0.65336883 | 0.6574459 | 0.660187576 | 0.663079235 | 0.665911944 | 0.66992588 | 0.675242697 | 0.67921824 | 0.681633215 | 0.684974509 | 0.691710126 | 0.698618031 | 0.705135529 | 0.712888693 | 0.719002843 | 0.721294303 | 0.723122973 |
| Chile | 0.5864951 | 0.59300658 | 0.599106083 | 0.604828509 | 0.610673933 | 0.617234677 | 0.62369446 | 0.630540085 | 0.638082758 | 0.645928084 | 0.652989028 | 0.66026551 | 0.667888408 | 0.673445927 | 0.678258212 | 0.682589191 | 0.685892748 | 0.688930323 | 0.692359787 | 0.696602141 | 0.702304704 | 0.708597936 | 0.714743477 | 0.720448781 | 0.727275927 | 0.736098082 | 0.744972224 | 0.752058077 | 0.758625714 | 0.765120323 | 0.769213676 | 0.771514716 |
| Uruguay | 0.581921855 | 0.586156118 | 0.589822355 | 0.592295025 | 0.595188424 | 0.596825409 | 0.599775104 | 0.605665051 | 0.612700815 | 0.619009271 | 0.623208291 | 0.625796068 | 0.6282858 | 0.631437774 | 0.634216906 | 0.63697577 | 0.640590696 | 0.646331714 | 0.653525522 | 0.660321135 | 0.666099135 | 0.667079733 | 0.665802192 | 0.668228361 | 0.67333426 | 0.680595506 | 0.689816733 | 0.699189456 | 0.70690493 | 0.712536588 | 0.716169766 | 0.719283445 |
| Western Europe | 0.746400479 | 0.751670034 | 0.757021934 | 0.762056783 | 0.766693114 | 0.770621721 | 0.774079855 | 0.777577691 | 0.780866892 | 0.784019197 | 0.787400011 | 0.790904403 | 0.794165178 | 0.796961505 | 0.79971572 | 0.802414038 | 0.80506685 | 0.807723334 | 0.810484377 | 0.813110222 | 0.816142165 | 0.819375239 | 0.822633726 | 0.825786901 | 0.828609513 | 0.831321799 | 0.834216019 | 0.837522174 | 0.84083355 | 0.844212109 | 0.846550359 | 0.848726316 |
| Andorra | 0.76146388 | 0.764709071 | 0.767188282 | 0.768953828 | 0.770023765 | 0.770988683 | 0.772403471 | 0.774580971 | 0.776972578 | 0.779629634 | 0.781976506 | 0.785417035 | 0.790158759 | 0.797278901 | 0.80473198 | 0.81221398 | 0.81978726 | 0.826617254 | 0.831821715 | 0.835929887 | 0.839489966 | 0.842794802 | 0.845810831 | 0.84832991 | 0.851010492 | 0.853591218 | 0.856257053 | 0.85947703 | 0.862588951 | 0.865621024 | 0.867449169 | 0.869444113 |
| Austria | 0.749853693 | 0.751652734 | 0.754375286 | 0.758804554 | 0.764704364 | 0.769827471 | 0.774446565 | 0.779232856 | 0.783521016 | 0.787451584 | 0.79184768 | 0.795770118 | 0.79915412 | 0.802043253 | 0.804889144 | 0.808419194 | 0.812064609 | 0.815480522 | 0.818793658 | 0.821541167 | 0.824500109 | 0.828001999 | 0.831113309 | 0.83367978 | 0.835838577 | 0.837579895 | 0.83988379 | 0.843173331 | 0.84661447 | 0.849862515 | 0.852020385 | 0.853837004 |
| Belgium | 0.737390656 | 0.742173074 | 0.748012299 | 0.753685087 | 0.758903638 | 0.763170401 | 0.766826075 | 0.770813953 | 0.774228268 | 0.77704104 | 0.780396331 | 0.784713043 | 0.788836054 | 0.792014384 | 0.795224936 | 0.798497236 | 0.801572763 | 0.804612664 | 0.807757945 | 0.811055825 | 0.815030207 | 0.819470362 | 0.824226016 | 0.828619832 | 0.832425052 | 0.8358784 | 0.83946294 | 0.843212044 | 0.846617625 | 0.849498395 | 0.851346649 | 0.853654016 |
| Cyprus | 0.648230872 | 0.655726169 | 0.666011849 | 0.677547477 | 0.688829361 | 0.699923898 | 0.709839946 | 0.718864244 | 0.727964382 | 0.736846135 | 0.745199301 | 0.753594717 | 0.761502465 | 0.767896216 | 0.773764775 | 0.779031361 | 0.784560965 | 0.792068383 | 0.799454089 | 0.805112085 | 0.809840188 | 0.813726132 | 0.816978525 | 0.819380602 | 0.821184302 | 0.822732851 | 0.824283624 | 0.826139878 | 0.828343243 | 0.830980728 | 0.833059523 | 0.835630545 |
| Denmark | 0.801154655 | 0.80454035 | 0.808164434 | 0.811585713 | 0.815138614 | 0.819104612 | 0.823375243 | 0.827679979 | 0.831683237 | 0.835381543 | 0.839184298 | 0.84317775 | 0.847086357 | 0.850441326 | 0.853304103 | 0.855690732 | 0.85803321 | 0.859781734 | 0.861782061 | 0.86394805 | 0.866767079 | 0.869778682 | 0.87251751 | 0.874932974 | 0.876814932 | 0.878543945 | 0.880589783 | 0.883688487 | 0.887529846 | 0.891624571 | 0.894368297 | 0.896424204 |
| Finland | 0.756221509 | 0.758410687 | 0.7608041 | 0.763526604 | 0.767101901 | 0.771163742 | 0.774809059 | 0.778814215 | 0.782305059 | 0.785773988 | 0.78998523 | 0.794453835 | 0.798770274 | 0.802325431 | 0.805694227 | 0.808936787 | 0.812081482 | 0.815597613 | 0.818875544 | 0.821457588 | 0.824844982 | 0.828270577 | 0.831434484 | 0.834425401 | 0.837431872 | 0.840878477 | 0.844622338 | 0.848586128 | 0.852202943 | 0.855365179 | 0.857655553 | 0.859831368 |
| France | 0.730747466 | 0.736452382 | 0.742871973 | 0.748582327 | 0.753176132 | 0.757125789 | 0.760899579 | 0.764650672 | 0.767852561 | 0.770304363 | 0.772792033 | 0.776225157 | 0.780088872 | 0.783178757 | 0.786084655 | 0.789042086 | 0.79232192 | 0.795557651 | 0.79817743 | 0.80050867 | 0.803240247 | 0.806378738 | 0.809615389 | 0.812984015 | 0.816551448 | 0.820238385 | 0.823730929 | 0.827098441 | 0.83044859 | 0.833772805 | 0.836049443 | 0.838364875 |
| Germany | 0.817077666 | 0.823339898 | 0.828323503 | 0.832286809 | 0.835561963 | 0.837643152 | 0.839188129 | 0.841385903 | 0.843854978 | 0.846232398 | 0.848925756 | 0.852070413 | 0.855066793 | 0.85767579 | 0.860240333 | 0.862894178 | 0.865873707 | 0.86914044 | 0.872552849 | 0.875087911 | 0.87794671 | 0.881027508 | 0.883656408 | 0.885926953 | 0.887651614 | 0.888867854 | 0.890896369 | 0.894050472 | 0.896975545 | 0.899703158 | 0.901438614 | 0.902957091 |
| Greece | 0.674186465 | 0.68031648 | 0.687055389 | 0.693328926 | 0.699153022 | 0.704862873 | 0.710139336 | 0.715687268 | 0.721593982 | 0.727529455 | 0.73271054 | 0.737154008 | 0.741945221 | 0.747185956 | 0.752418607 | 0.756384396 | 0.760209696 | 0.763927173 | 0.767527658 | 0.771247356 | 0.775155815 | 0.778525867 | 0.780789483 | 0.781949052 | 0.782442013 | 0.782489523 | 0.782798443 | 0.784108594 | 0.785999753 | 0.788138138 | 0.78963222 | 0.791854408 |
| Iceland | 0.764212517 | 0.769027393 | 0.772706415 | 0.776389423 | 0.779967961 | 0.782740057 | 0.78535642 | 0.788528335 | 0.792626368 | 0.797368808 | 0.80303033 | 0.808965786 | 0.813400392 | 0.816597067 | 0.819528087 | 0.822766536 | 0.826244743 | 0.83028989 | 0.834622678 | 0.838121013 | 0.841384642 | 0.84461993 | 0.847299444 | 0.850173948 | 0.853558697 | 0.857724976 | 0.861844467 | 0.865702891 | 0.869219822 | 0.87242558 | 0.874323153 | 0.87636168 |
| Ireland | 0.719891819 | 0.725195019 | 0.731918691 | 0.738384074 | 0.744011728 | 0.748921575 | 0.754010124 | 0.759970384 | 0.766488721 | 0.773538834 | 0.780190655 | 0.786353741 | 0.793010606 | 0.800005865 | 0.80685314 | 0.811713818 | 0.814505479 | 0.816931026 | 0.819932128 | 0.823929666 | 0.828103848 | 0.831804792 | 0.835325359 | 0.838644433 | 0.842082115 | 0.847437887 | 0.852642502 | 0.857816164 | 0.863925284 | 0.869412503 | 0.871958822 | 0.87375385 |
| Israel | 0.709178347 | 0.713371633 | 0.717897923 | 0.722250176 | 0.726654736 | 0.731127859 | 0.734714026 | 0.738015277 | 0.74192719 | 0.745443338 | 0.749184084 | 0.752834297 | 0.755988076 | 0.758875409 | 0.762213291 | 0.765543461 | 0.768733898 | 0.770756143 | 0.772011615 | 0.773303913 | 0.77525953 | 0.777649669 | 0.780403415 | 0.783267167 | 0.786151084 | 0.788874457 | 0.791772445 | 0.795173437 | 0.799124994 | 0.803232991 | 0.806351589 | 0.809011652 |
| Italy | 0.706255224 | 0.711457349 | 0.716649746 | 0.721720992 | 0.726870913 | 0.731644082 | 0.73605087 | 0.739972336 | 0.743101596 | 0.746074084 | 0.749675986 | 0.753554839 | 0.757191479 | 0.760093744 | 0.762787749 | 0.765689914 | 0.768402408 | 0.77069838 | 0.77306847 | 0.775499262 | 0.778109206 | 0.780703468 | 0.783090981 | 0.785356877 | 0.787533956 | 0.789902119 | 0.792477244 | 0.795311186 | 0.79836269 | 0.801530021 | 0.80363568 | 0.805773534 |
| Abruzzo | 0.714650756 | 0.720314474 | 0.726036648 | 0.731441857 | 0.736853274 | 0.742135794 | 0.747013718 | 0.75138555 | 0.754985849 | 0.758414771 | 0.762309511 | 0.766271428 | 0.769810782 | 0.772591285 | 0.774920803 | 0.77748367 | 0.780034109 | 0.782335096 | 0.78475363 | 0.787067403 | 0.789594271 | 0.792388751 | 0.795033523 | 0.797428884 | 0.799640686 | 0.801911688 | 0.804328502 | 0.807027985 | 0.8099339 | 0.812941682 | 0.814853743 | 0.816844362 |
| Basilicata | 0.666507396 | 0.672611491 | 0.678846405 | 0.684857076 | 0.690931633 | 0.696773911 | 0.702437784 | 0.707815571 | 0.712597373 | 0.717439425 | 0.722474045 | 0.72738238 | 0.731856204 | 0.73532332 | 0.738453185 | 0.741572183 | 0.744738215 | 0.747716263 | 0.750560689 | 0.752971143 | 0.755301032 | 0.757796971 | 0.760122256 | 0.762534447 | 0.764566446 | 0.767231505 | 0.769938709 | 0.772992333 | 0.776198806 | 0.779479535 | 0.781707729 | 0.784016427 |
| Calabria | 0.656864369 | 0.663315571 | 0.669924506 | 0.676566868 | 0.683266057 | 0.689455482 | 0.695133568 | 0.700346283 | 0.704675627 | 0.70898274 | 0.71376972 | 0.718760514 | 0.72334746 | 0.727115967 | 0.730640811 | 0.73425293 | 0.737680692 | 0.740692771 | 0.74375746 | 0.746812852 | 0.749792421 | 0.752585913 | 0.755026295 | 0.757109552 | 0.758942326 | 0.760881929 | 0.763140529 | 0.765782067 | 0.7686683 | 0.771722078 | 0.773667682 | 0.775713053 |
| Campania | 0.661702253 | 0.667520742 | 0.673477318 | 0.679435188 | 0.685497259 | 0.690950704 | 0.695804806 | 0.700110353 | 0.703349843 | 0.706425625 | 0.710384001 | 0.714832506 | 0.719124693 | 0.72245743 | 0.725511919 | 0.728812162 | 0.731796379 | 0.734297928 | 0.736802869 | 0.739399386 | 0.741904705 | 0.744177482 | 0.746309839 | 0.74826609 | 0.750108382 | 0.752185579 | 0.754640228 | 0.757244646 | 0.760097593 | 0.763089194 | 0.76501158 | 0.766943369 |
| Emilia-Romagna | 0.721948871 | 0.727184143 | 0.732437186 | 0.737706301 | 0.743180402 | 0.748223375 | 0.753051528 | 0.757340662 | 0.760781386 | 0.764140844 | 0.768271045 | 0.772538731 | 0.776282088 | 0.779019607 | 0.781429184 | 0.784124518 | 0.786732201 | 0.788933636 | 0.791338539 | 0.793965803 | 0.796928825 | 0.800084705 | 0.803078264 | 0.8060609 | 0.808956556 | 0.811966214 | 0.815135918 | 0.81840568 | 0.821823859 | 0.825242785 | 0.827499175 | 0.829724714 |
| Friuli-Venezia Giulia | 0.724866124 | 0.72994147 | 0.734873099 | 0.739543768 | 0.74431686 | 0.74912879 | 0.753536933 | 0.757480159 | 0.760771763 | 0.763955725 | 0.767611599 | 0.771418969 | 0.774825039 | 0.777382095 | 0.779659716 | 0.782196659 | 0.78473396 | 0.78691235 | 0.788986271 | 0.790979908 | 0.793273797 | 0.795636551 | 0.79771559 | 0.799865299 | 0.80196795 | 0.804376757 | 0.807022862 | 0.809845083 | 0.812872642 | 0.816036204 | 0.818126931 | 0.820302061 |
| Lazio | 0.737620712 | 0.742562604 | 0.747476738 | 0.75217172 | 0.756881007 | 0.761258174 | 0.765200121 | 0.768876599 | 0.772108974 | 0.77514763 | 0.778568808 | 0.782171677 | 0.785616923 | 0.788425471 | 0.791132889 | 0.79392525 | 0.796433169 | 0.798544925 | 0.800550062 | 0.802613205 | 0.804809539 | 0.806966794 | 0.808829668 | 0.810556633 | 0.812162864 | 0.813951936 | 0.815952403 | 0.818295398 | 0.820892974 | 0.823653274 | 0.825448518 | 0.827338854 |
| Liguria | 0.730184642 | 0.734884027 | 0.739396416 | 0.743578301 | 0.747799606 | 0.751953443 | 0.75585102 | 0.759456466 | 0.762584744 | 0.765514591 | 0.768910221 | 0.772530273 | 0.775708849 | 0.77832143 | 0.780718147 | 0.783248589 | 0.785637499 | 0.787794085 | 0.790104023 | 0.79236739 | 0.794703085 | 0.797129188 | 0.799372589 | 0.801546507 | 0.803757664 | 0.806187607 | 0.808805761 | 0.811581509 | 0.814582992 | 0.817754688 | 0.819904952 | 0.822133391 |
| Lombardia | 0.742347949 | 0.747008975 | 0.751487308 | 0.755700771 | 0.759947659 | 0.763880108 | 0.767494336 | 0.770699542 | 0.773316188 | 0.77567219 | 0.778464086 | 0.781511232 | 0.784403178 | 0.786658469 | 0.788686704 | 0.790911296 | 0.792946525 | 0.794603498 | 0.79657114 | 0.798688188 | 0.801241996 | 0.803843779 | 0.80622528 | 0.808492554 | 0.810749863 | 0.813192888 | 0.815835408 | 0.818754967 | 0.8218674 | 0.825097687 | 0.827249763 | 0.829429918 |
| Marche | 0.697071608 | 0.702424493 | 0.70784338 | 0.713228463 | 0.718742438 | 0.723813645 | 0.728647889 | 0.733058156 | 0.736742308 | 0.740419601 | 0.744717927 | 0.749120978 | 0.753266338 | 0.756685771 | 0.759825008 | 0.76309227 | 0.766221112 | 0.768994909 | 0.771713392 | 0.774452637 | 0.777209008 | 0.779849784 | 0.782223007 | 0.784415899 | 0.786547154 | 0.78879484 | 0.791181254 | 0.793720129 | 0.796550133 | 0.799507565 | 0.801466426 | 0.803473184 |
| Molise | 0.676593732 | 0.682668668 | 0.688908551 | 0.694994766 | 0.701219137 | 0.707208041 | 0.712892946 | 0.718413588 | 0.723261661 | 0.727778774 | 0.732626449 | 0.737493769 | 0.741963545 | 0.745613573 | 0.74901584 | 0.75235123 | 0.755822928 | 0.759108179 | 0.76205899 | 0.764767398 | 0.767240047 | 0.769577055 | 0.771550126 | 0.772922815 | 0.774073933 | 0.77544044 | 0.777279822 | 0.779405578 | 0.78188174 | 0.784608168 | 0.786360968 | 0.788350602 |
| Piemonte | 0.720360415 | 0.724925882 | 0.729438003 | 0.733675332 | 0.738002739 | 0.742133738 | 0.745882478 | 0.749299394 | 0.752084872 | 0.754728908 | 0.757802459 | 0.761047975 | 0.764064777 | 0.766471214 | 0.768680124 | 0.771065429 | 0.773345616 | 0.775246752 | 0.777149579 | 0.778979509 | 0.781148533 | 0.78340101 | 0.785422049 | 0.787438688 | 0.789410681 | 0.791618357 | 0.794040433 | 0.796749223 | 0.799706693 | 0.802813397 | 0.804900631 | 0.80703906 |
| Provincia autonoma di Bolzano | 0.732400638 | 0.737857914 | 0.743168764 | 0.748346382 | 0.753629005 | 0.758853531 | 0.76380399 | 0.767954752 | 0.771384014 | 0.774456235 | 0.778193877 | 0.782131782 | 0.78557187 | 0.788197747 | 0.790596357 | 0.793191215 | 0.79577814 | 0.797981456 | 0.800492982 | 0.803296738 | 0.806355644 | 0.809507495 | 0.812686784 | 0.81577868 | 0.818682328 | 0.821801112 | 0.825089844 | 0.828359195 | 0.831803971 | 0.835223636 | 0.837533583 | 0.839711339 |
| Provincia autonoma di Trento | 0.73819977 | 0.74282506 | 0.747253469 | 0.751445442 | 0.755646179 | 0.759487795 | 0.763090364 | 0.76618372 | 0.768732353 | 0.77128382 | 0.774456189 | 0.777965404 | 0.781240906 | 0.783865578 | 0.78621061 | 0.788746589 | 0.791058925 | 0.793014867 | 0.795195785 | 0.797608908 | 0.800258139 | 0.802918274 | 0.805445765 | 0.808166307 | 0.8107183 | 0.813299855 | 0.815953749 | 0.818815088 | 0.82189684 | 0.825090209 | 0.827225375 | 0.829384918 |
| Puglia | 0.657350626 | 0.663352452 | 0.669409029 | 0.675292038 | 0.681240093 | 0.686760664 | 0.691821949 | 0.69621578 | 0.69972705 | 0.703207369 | 0.707432129 | 0.711871851 | 0.715974391 | 0.719246338 | 0.722198909 | 0.725255528 | 0.72809395 | 0.730484684 | 0.732742105 | 0.735021669 | 0.73741543 | 0.739756848 | 0.74204733 | 0.744126245 | 0.746119992 | 0.748360443 | 0.750729081 | 0.753599373 | 0.756669008 | 0.759819029 | 0.761882503 | 0.76398208 |
| Sardegna | 0.679922875 | 0.685123182 | 0.690290443 | 0.695245687 | 0.70019795 | 0.704781418 | 0.708967469 | 0.712966475 | 0.716299229 | 0.719548668 | 0.72321647 | 0.727093173 | 0.730646957 | 0.733701802 | 0.736569001 | 0.739396126 | 0.742114948 | 0.744516524 | 0.74693411 | 0.749179711 | 0.751429496 | 0.753586394 | 0.755585265 | 0.757197458 | 0.758674878 | 0.760461605 | 0.762361543 | 0.764539716 | 0.76692095 | 0.769462663 | 0.771065933 | 0.772793427 |
| Sicilia | 0.657025838 | 0.662897641 | 0.668946236 | 0.675082629 | 0.681367885 | 0.687160758 | 0.692495615 | 0.696977061 | 0.700008669 | 0.702797087 | 0.706568293 | 0.710836713 | 0.714885611 | 0.718010356 | 0.720915948 | 0.724326379 | 0.727453965 | 0.72993636 | 0.732453075 | 0.735208709 | 0.737949835 | 0.740402564 | 0.742713171 | 0.744809406 | 0.746634981 | 0.748691268 | 0.75092322 | 0.753452911 | 0.756271655 | 0.759283639 | 0.761187608 | 0.76308681 |
| Toscana | 0.713229274 | 0.718119176 | 0.722962647 | 0.727582851 | 0.732172134 | 0.736513094 | 0.740530304 | 0.744217322 | 0.747383239 | 0.750402163 | 0.753878275 | 0.757509437 | 0.760922763 | 0.763742562 | 0.766387263 | 0.769195841 | 0.77191678 | 0.77429999 | 0.776801982 | 0.779428719 | 0.782198274 | 0.784979732 | 0.787583097 | 0.790068653 | 0.792506107 | 0.795119235 | 0.797917666 | 0.800829838 | 0.803952938 | 0.80719229 | 0.809403865 | 0.811654111 |
| Umbria | 0.706686715 | 0.711703294 | 0.716750971 | 0.721590593 | 0.726422295 | 0.731104304 | 0.735293537 | 0.739163012 | 0.742302398 | 0.745232881 | 0.748661758 | 0.752215672 | 0.755444793 | 0.758053929 | 0.760499872 | 0.763159734 | 0.765795039 | 0.768114508 | 0.770583361 | 0.772908278 | 0.775414581 | 0.777832889 | 0.779986946 | 0.781953104 | 0.783600689 | 0.785536288 | 0.787557536 | 0.789865843 | 0.792498638 | 0.795274397 | 0.797133075 | 0.799101322 |
| Valle d'Aosta | 0.726784665 | 0.731705275 | 0.736548149 | 0.741102591 | 0.745640193 | 0.750137728 | 0.754142816 | 0.757432036 | 0.759929736 | 0.762095115 | 0.76461864 | 0.767545717 | 0.770281178 | 0.772547957 | 0.774568158 | 0.776816354 | 0.778969871 | 0.780694251 | 0.782655938 | 0.784700769 | 0.787182711 | 0.789811855 | 0.792424492 | 0.794694011 | 0.796752354 | 0.79884438 | 0.801080525 | 0.803438241 | 0.805989884 | 0.80868573 | 0.810449762 | 0.812304291 |
| Veneto | 0.714979957 | 0.719998279 | 0.724834198 | 0.729421168 | 0.733951591 | 0.738130376 | 0.742059383 | 0.745613004 | 0.748588806 | 0.751361817 | 0.754649954 | 0.758093566 | 0.761164725 | 0.763774747 | 0.766279521 | 0.768947771 | 0.771510221 | 0.773649157 | 0.775817913 | 0.778035418 | 0.780421806 | 0.782934183 | 0.785223094 | 0.787451765 | 0.789630294 | 0.792001513 | 0.794640114 | 0.797608536 | 0.800784149 | 0.80404827 | 0.80626835 | 0.808534438 |
| Luxembourg | 0.781051609 | 0.785961442 | 0.789053364 | 0.792843836 | 0.797824008 | 0.80268247 | 0.806516138 | 0.810235288 | 0.813889026 | 0.817570995 | 0.822006568 | 0.825312816 | 0.828223491 | 0.830983946 | 0.833672567 | 0.836675264 | 0.840574453 | 0.844707006 | 0.847978883 | 0.850688725 | 0.853258277 | 0.856216834 | 0.859789031 | 0.863368792 | 0.866693556 | 0.870112937 | 0.87342088 | 0.876347684 | 0.878637895 | 0.880593895 | 0.882495316 | 0.884428955 |
| Malta | 0.656504584 | 0.661112691 | 0.666324648 | 0.672120257 | 0.677847314 | 0.682617704 | 0.686623261 | 0.691756406 | 0.698095287 | 0.704734545 | 0.711805254 | 0.717803038 | 0.722790275 | 0.727511133 | 0.731933853 | 0.736188747 | 0.740140181 | 0.744237812 | 0.748427095 | 0.752171059 | 0.7562505 | 0.760310547 | 0.76453788 | 0.76861614 | 0.772728741 | 0.777276905 | 0.78167341 | 0.786410184 | 0.790965618 | 0.795197846 | 0.798375166 | 0.801585034 |
| Monaco | 0.845495153 | 0.84830369 | 0.85086394 | 0.853264992 | 0.855626927 | 0.857986147 | 0.860245432 | 0.86244672 | 0.864716513 | 0.866842394 | 0.868973583 | 0.871094755 | 0.873182769 | 0.875197652 | 0.877231549 | 0.879288089 | 0.881274148 | 0.883262291 | 0.885331394 | 0.887328279 | 0.889262841 | 0.891237592 | 0.893073822 | 0.894864472 | 0.896547874 | 0.898356337 | 0.900051303 | 0.901752712 | 0.903388687 | 0.905030991 | 0.906685437 | 0.908262831 |
| Netherlands | 0.794612123 | 0.799285437 | 0.803965978 | 0.808278967 | 0.812530216 | 0.816662769 | 0.81998422 | 0.82288177 | 0.825882234 | 0.828693827 | 0.831913984 | 0.835518148 | 0.838676077 | 0.841639477 | 0.844807348 | 0.84793419 | 0.850798808 | 0.853595391 | 0.856368467 | 0.858600459 | 0.861255298 | 0.864246678 | 0.866950626 | 0.869402979 | 0.871848914 | 0.874402841 | 0.876784052 | 0.879300152 | 0.881900218 | 0.88464384 | 0.886558566 | 0.888464256 |
| Norway | 0.795887277 | 0.800468886 | 0.805437473 | 0.810565689 | 0.815414668 | 0.820223522 | 0.825701668 | 0.83146877 | 0.836562809 | 0.841130237 | 0.846475451 | 0.852111936 | 0.856757888 | 0.860302576 | 0.863468908 | 0.866217488 | 0.868588435 | 0.87092967 | 0.873745121 | 0.876582817 | 0.880378381 | 0.884351667 | 0.888160475 | 0.891989618 | 0.895499546 | 0.898912644 | 0.902584187 | 0.906044049 | 0.909261782 | 0.912278761 | 0.91452992 | 0.91613281 |
| Agder | 0.783912098 | 0.788332788 | 0.79314313 | 0.798179607 | 0.802898816 | 0.807571929 | 0.81314131 | 0.819160991 | 0.824508265 | 0.829429423 | 0.835206002 | 0.841137184 | 0.845788867 | 0.848861265 | 0.851230755 | 0.853247166 | 0.855131403 | 0.857111766 | 0.859646563 | 0.863012998 | 0.867775168 | 0.872804104 | 0.877660514 | 0.88240259 | 0.886464538 | 0.890237549 | 0.893916649 | 0.897491112 | 0.90098392 | 0.904361013 | 0.906183377 | 0.907489539 |
| Innlandet | 0.78002909 | 0.784849736 | 0.789925941 | 0.795040066 | 0.800016866 | 0.804874963 | 0.81013378 | 0.815499936 | 0.820122385 | 0.824149118 | 0.828876562 | 0.834071748 | 0.838551994 | 0.842130107 | 0.845459347 | 0.848176723 | 0.850255277 | 0.851981464 | 0.853899928 | 0.856474848 | 0.86025388 | 0.864296676 | 0.868500928 | 0.872746811 | 0.876683206 | 0.880696912 | 0.884723091 | 0.888690957 | 0.892398558 | 0.895981516 | 0.898080574 | 0.899754056 |
| Møre og Romsdal | 0.779159162 | 0.784374841 | 0.789929274 | 0.7956521 | 0.800960694 | 0.805986693 | 0.811592672 | 0.817417706 | 0.822556658 | 0.827212892 | 0.832715855 | 0.838381936 | 0.843125543 | 0.846893807 | 0.850360738 | 0.853295324 | 0.855735212 | 0.857889479 | 0.860346277 | 0.863764973 | 0.868646242 | 0.873733552 | 0.877862552 | 0.881991738 | 0.885839731 | 0.889490109 | 0.89335062 | 0.896936857 | 0.90026227 | 0.90339563 | 0.905740097 | 0.907426289 |
| Nordland | 0.764634665 | 0.769725265 | 0.775292839 | 0.780791239 | 0.785865667 | 0.790924709 | 0.796892447 | 0.802989116 | 0.808160059 | 0.81237422 | 0.817444225 | 0.823004333 | 0.827595559 | 0.830860462 | 0.833560254 | 0.835700116 | 0.837292129 | 0.838519795 | 0.840154234 | 0.84295699 | 0.847557075 | 0.852680384 | 0.857835369 | 0.862926165 | 0.867608704 | 0.872602911 | 0.87791292 | 0.883038499 | 0.887842449 | 0.892254103 | 0.895051975 | 0.896942278 |
| Oslo | 0.839440851 | 0.842718294 | 0.846455149 | 0.850400684 | 0.854382432 | 0.858623102 | 0.863699754 | 0.869486225 | 0.874881254 | 0.879981632 | 0.886028439 | 0.892304447 | 0.896187571 | 0.899090556 | 0.901749138 | 0.904222491 | 0.906444546 | 0.90866384 | 0.911216234 | 0.914411069 | 0.91810535 | 0.921609921 | 0.924858063 | 0.927953073 | 0.930766814 | 0.9334734 | 0.936252518 | 0.938785621 | 0.941017206 | 0.943006745 | 0.94451283 | 0.945691791 |
| Rogaland | 0.790232798 | 0.795102916 | 0.800853825 | 0.806845242 | 0.812333764 | 0.817488085 | 0.823108012 | 0.829018909 | 0.834332203 | 0.839124112 | 0.844759276 | 0.850559161 | 0.854984294 | 0.858246021 | 0.861262987 | 0.863900761 | 0.866344909 | 0.86740461 | 0.86848793 | 0.871094607 | 0.875193657 | 0.879758685 | 0.884231912 | 0.888715554 | 0.892707683 | 0.896469727 | 0.900462034 | 0.904222076 | 0.907848148 | 0.911139221 | 0.913509653 | 0.915083733 |
| Troms og Finnmark | 0.77581152 | 0.780265229 | 0.7853149 | 0.790702564 | 0.796009998 | 0.80143653 | 0.807533278 | 0.813683372 | 0.818758455 | 0.822973826 | 0.82812721 | 0.833782263 | 0.838579848 | 0.842143187 | 0.845136134 | 0.847215525 | 0.848801574 | 0.850225116 | 0.852223375 | 0.8556188 | 0.860948699 | 0.866780601 | 0.872349762 | 0.877347845 | 0.881473764 | 0.8853693 | 0.889548167 | 0.893369864 | 0.896972481 | 0.900550473 | 0.903265075 | 0.905163204 |
| Trøndelag | 0.784941155 | 0.790034372 | 0.795463405 | 0.800889108 | 0.805914648 | 0.810700901 | 0.816224863 | 0.822159304 | 0.827407436 | 0.8320705 | 0.837515718 | 0.843442199 | 0.848495926 | 0.852433072 | 0.85574463 | 0.858546848 | 0.860966125 | 0.863389024 | 0.86648419 | 0.870473186 | 0.875594833 | 0.880727641 | 0.8855232 | 0.890174387 | 0.893985844 | 0.89755853 | 0.901327842 | 0.904938475 | 0.908326408 | 0.911706699 | 0.914235528 | 0.915977587 |
| Vestfold og Telemark | 0.790051833 | 0.794780111 | 0.79954847 | 0.804272723 | 0.808580434 | 0.812995348 | 0.818342689 | 0.824152521 | 0.829269348 | 0.833812082 | 0.839065149 | 0.844660797 | 0.849336148 | 0.852737252 | 0.85557901 | 0.857912257 | 0.85977761 | 0.861489776 | 0.863615603 | 0.866416019 | 0.870241561 | 0.874170437 | 0.878117669 | 0.882056846 | 0.885721403 | 0.889436049 | 0.893194565 | 0.896913898 | 0.900492293 | 0.90394682 | 0.905871187 | 0.907307419 |
| Vestland | 0.789791575 | 0.79490379 | 0.800378133 | 0.806037267 | 0.811382054 | 0.816491018 | 0.822167287 | 0.827889616 | 0.832915531 | 0.837348232 | 0.842763132 | 0.848633203 | 0.853474418 | 0.857169642 | 0.860512058 | 0.863399231 | 0.865922343 | 0.868416896 | 0.871689771 | 0.875811054 | 0.880068098 | 0.88446835 | 0.888599361 | 0.892643882 | 0.896251247 | 0.899842261 | 0.903706572 | 0.907358501 | 0.910648846 | 0.913675529 | 0.91595896 | 0.917596286 |
| Viken | 0.811900165 | 0.816358577 | 0.821078772 | 0.825920984 | 0.830499396 | 0.835018647 | 0.840030512 | 0.845170776 | 0.849684467 | 0.853773523 | 0.858471481 | 0.863279251 | 0.867148115 | 0.870061564 | 0.872678402 | 0.874849383 | 0.876424804 | 0.876736851 | 0.87764793 | 0.8798049 | 0.883116601 | 0.886541008 | 0.889863533 | 0.893226632 | 0.89630876 | 0.899291858 | 0.902539065 | 0.905645805 | 0.908547881 | 0.911226761 | 0.913234341 | 0.914692607 |
| Portugal | 0.599777757 | 0.607269449 | 0.61480808 | 0.622101177 | 0.629306387 | 0.635498761 | 0.640864526 | 0.646192904 | 0.651335994 | 0.656120667 | 0.661610852 | 0.667247151 | 0.67258394 | 0.677663412 | 0.682352926 | 0.686887105 | 0.691158795 | 0.694974853 | 0.698604599 | 0.702223399 | 0.706563555 | 0.711262893 | 0.715804084 | 0.719836991 | 0.723021282 | 0.72575622 | 0.728300849 | 0.731225535 | 0.734402947 | 0.738090481 | 0.741037384 | 0.744151851 |
| San Marino | 0.813244888 | 0.818282513 | 0.822782758 | 0.827382324 | 0.831970983 | 0.836762726 | 0.841389495 | 0.845864865 | 0.850474771 | 0.855338613 | 0.859695363 | 0.863747757 | 0.865680592 | 0.867522009 | 0.869287134 | 0.870979596 | 0.87265215 | 0.874307253 | 0.875991881 | 0.877705228 | 0.879440814 | 0.881197518 | 0.882924531 | 0.884592627 | 0.885353694 | 0.884693365 | 0.884477665 | 0.884557607 | 0.885133516 | 0.88620066 | 0.886867937 | 0.888005474 |
| Spain | 0.636673166 | 0.644130745 | 0.651597597 | 0.658637569 | 0.665321549 | 0.671576034 | 0.677088943 | 0.682429497 | 0.687490717 | 0.692245197 | 0.697056031 | 0.70172607 | 0.706309655 | 0.710678584 | 0.71488623 | 0.718717289 | 0.721970128 | 0.725211443 | 0.729411041 | 0.733956055 | 0.738352055 | 0.74209645 | 0.745181867 | 0.747651982 | 0.749800929 | 0.752269823 | 0.754757939 | 0.757663276 | 0.760659575 | 0.763942918 | 0.766506025 | 0.769283698 |
| Sweden | 0.785535792 | 0.790096441 | 0.795431458 | 0.801009522 | 0.806700264 | 0.812244654 | 0.817227025 | 0.821694059 | 0.825731995 | 0.829487099 | 0.833028112 | 0.835956398 | 0.838741527 | 0.841663138 | 0.844506882 | 0.846876344 | 0.849115871 | 0.851420847 | 0.853705851 | 0.855702748 | 0.858727941 | 0.861892379 | 0.864706672 | 0.867383768 | 0.869923325 | 0.872388515 | 0.874661655 | 0.877120611 | 0.879888912 | 0.882984116 | 0.88506201 | 0.886880299 |
| Stockholm | 0.828649292 | 0.832525028 | 0.837056407 | 0.841511909 | 0.846125387 | 0.850654749 | 0.854868259 | 0.858962185 | 0.862507239 | 0.865644835 | 0.86874746 | 0.871699893 | 0.874281664 | 0.876706566 | 0.879256399 | 0.881626909 | 0.883851426 | 0.886249494 | 0.888863855 | 0.891152891 | 0.8943043 | 0.897493993 | 0.900238956 | 0.902345396 | 0.90437172 | 0.906359995 | 0.908308393 | 0.910042557 | 0.911931497 | 0.91388968 | 0.915448181 | 0.916622714 |
| Sweden except Stockholm | 0.774127452 | 0.778841201 | 0.784342898 | 0.790150522 | 0.796045895 | 0.801770911 | 0.806862729 | 0.811330613 | 0.815395871 | 0.819215685 | 0.822787128 | 0.825651241 | 0.828445536 | 0.831456241 | 0.834336315 | 0.83666605 | 0.838858151 | 0.841067317 | 0.843179317 | 0.844988591 | 0.847857593 | 0.850890332 | 0.853589272 | 0.856164537 | 0.858583637 | 0.860863648 | 0.862929154 | 0.865303073 | 0.868031892 | 0.871181143 | 0.873303472 | 0.87515115 |
| Switzerland | 0.862766844 | 0.864796867 | 0.867736079 | 0.870863558 | 0.873140871 | 0.874835331 | 0.876640557 | 0.878485545 | 0.880035152 | 0.882045586 | 0.885243531 | 0.888445951 | 0.890902811 | 0.892780807 | 0.894909812 | 0.89727263 | 0.899962305 | 0.902913413 | 0.905778454 | 0.908049998 | 0.910530522 | 0.913276573 | 0.915714893 | 0.917955006 | 0.919994182 | 0.921832991 | 0.923930598 | 0.926202291 | 0.92855059 | 0.930682102 | 0.932027645 | 0.933059111 |
| UK | 0.744334126 | 0.749148231 | 0.754556689 | 0.76015435 | 0.7651646 | 0.768656864 | 0.771576484 | 0.775050122 | 0.779057165 | 0.784036475 | 0.78921896 | 0.793513165 | 0.79665556 | 0.799186111 | 0.80195087 | 0.804489194 | 0.806817046 | 0.809352772 | 0.812039287 | 0.814793642 | 0.818033994 | 0.821718995 | 0.826453754 | 0.831508668 | 0.835516642 | 0.838951919 | 0.842460233 | 0.846263618 | 0.849969788 | 0.854130573 | 0.85692029 | 0.859000182 |
| England | 0.745220814 | 0.750010695 | 0.755362083 | 0.760861507 | 0.765852166 | 0.769445553 | 0.772571526 | 0.776369611 | 0.780575801 | 0.785737637 | 0.791218167 | 0.795728741 | 0.798931223 | 0.801422116 | 0.804229161 | 0.80686705 | 0.80929741 | 0.811878279 | 0.814510302 | 0.817149149 | 0.820248074 | 0.823888064 | 0.828814735 | 0.834175981 | 0.838287848 | 0.841762298 | 0.845301132 | 0.849192107 | 0.853015796 | 0.85723951 | 0.859846755 | 0.861437309 |
| East Midlands | 0.720595588 | 0.725382189 | 0.730646772 | 0.736028016 | 0.740970402 | 0.744731977 | 0.748029077 | 0.751865275 | 0.756017311 | 0.761092911 | 0.766504947 | 0.77091617 | 0.773976472 | 0.776302898 | 0.778881547 | 0.781276256 | 0.783467778 | 0.785811632 | 0.78826944 | 0.790748773 | 0.793722444 | 0.797311942 | 0.802290193 | 0.807619102 | 0.811635093 | 0.815140939 | 0.818866498 | 0.823027658 | 0.827107376 | 0.831569842 | 0.834248143 | 0.835856049 |
| Derby | 0.729286457 | 0.734133474 | 0.740362913 | 0.747124171 | 0.753028474 | 0.757013791 | 0.759883575 | 0.763064357 | 0.766716021 | 0.771726951 | 0.777273547 | 0.781685175 | 0.784817185 | 0.787243876 | 0.789857006 | 0.791679853 | 0.793004535 | 0.794675388 | 0.797022608 | 0.800032055 | 0.803214798 | 0.806665952 | 0.812015129 | 0.818033612 | 0.822819186 | 0.827102987 | 0.831026138 | 0.834720006 | 0.837971939 | 0.841759874 | 0.844305892 | 0.846010683 |
| Derbyshire | 0.713327582 | 0.717621039 | 0.722732128 | 0.727957391 | 0.732604274 | 0.735976543 | 0.738909926 | 0.742314924 | 0.74595707 | 0.750737546 | 0.755768881 | 0.759582766 | 0.76190732 | 0.763261937 | 0.764939488 | 0.766737093 | 0.768627808 | 0.770493303 | 0.772073502 | 0.773739343 | 0.776470659 | 0.780495474 | 0.786587281 | 0.793174593 | 0.798115371 | 0.802333418 | 0.806559661 | 0.811072552 | 0.815358055 | 0.81982696 | 0.822551445 | 0.824238096 |
| Leicester | 0.709100479 | 0.714837923 | 0.720499844 | 0.725856916 | 0.731189577 | 0.735508537 | 0.739395071 | 0.743846264 | 0.748201117 | 0.753065154 | 0.758143825 | 0.762269684 | 0.765316094 | 0.768058038 | 0.771496909 | 0.774860919 | 0.777834262 | 0.780959228 | 0.784570211 | 0.788353525 | 0.792657572 | 0.79717113 | 0.802161284 | 0.806902634 | 0.810110275 | 0.812604157 | 0.815217839 | 0.818155529 | 0.821236098 | 0.825030585 | 0.827484168 | 0.8290671 |
| Leicestershire | 0.749605138 | 0.754437304 | 0.759361182 | 0.764242331 | 0.76853066 | 0.771947325 | 0.775105495 | 0.778598236 | 0.782224664 | 0.786641392 | 0.791420048 | 0.795249651 | 0.797889621 | 0.800134684 | 0.802990051 | 0.805925748 | 0.808765576 | 0.811543763 | 0.814008206 | 0.815846111 | 0.817802988 | 0.820199578 | 0.824087611 | 0.828535894 | 0.831645713 | 0.834224824 | 0.83734304 | 0.840957482 | 0.844440937 | 0.848214886 | 0.850461117 | 0.851888947 |
| Lincolnshire | 0.713279193 | 0.717375606 | 0.721714956 | 0.726455465 | 0.731051075 | 0.734487907 | 0.73750007 | 0.741067708 | 0.744968286 | 0.749813215 | 0.754891416 | 0.758996546 | 0.761645216 | 0.763389666 | 0.765112198 | 0.766609139 | 0.767702458 | 0.768606331 | 0.769699566 | 0.771495971 | 0.774167508 | 0.777764999 | 0.783140614 | 0.788999523 | 0.793644111 | 0.797855618 | 0.802146295 | 0.806834452 | 0.811313961 | 0.816238426 | 0.819445322 | 0.821363757 |
| Northamptonshire | 0.731115469 | 0.736430778 | 0.742413863 | 0.748219029 | 0.753135116 | 0.756541719 | 0.759175237 | 0.76205477 | 0.764865474 | 0.768415453 | 0.772584343 | 0.776233319 | 0.778602739 | 0.780201942 | 0.781860614 | 0.783251567 | 0.784522145 | 0.786126423 | 0.787862475 | 0.789850855 | 0.792732034 | 0.796667117 | 0.802416396 | 0.808609718 | 0.813110179 | 0.816860849 | 0.820827752 | 0.825388927 | 0.829918874 | 0.834815127 | 0.837888978 | 0.839635363 |
| Nottingham | 0.739549358 | 0.744347228 | 0.749752031 | 0.755675822 | 0.761270252 | 0.765718479 | 0.769870011 | 0.774833163 | 0.780210006 | 0.785997331 | 0.791617368 | 0.79628495 | 0.799811234 | 0.802920142 | 0.806037454 | 0.808877432 | 0.81175174 | 0.815260399 | 0.819380042 | 0.823058575 | 0.826123972 | 0.829069575 | 0.832506053 | 0.835958439 | 0.838719214 | 0.841212169 | 0.844157624 | 0.847553502 | 0.851042154 | 0.855012486 | 0.857407491 | 0.85887136 |
| Nottinghamshire | 0.715311951 | 0.71968933 | 0.724606164 | 0.729613454 | 0.734297939 | 0.737770308 | 0.740783285 | 0.74447891 | 0.748548904 | 0.753457256 | 0.758501711 | 0.762368908 | 0.76487914 | 0.766612871 | 0.768795277 | 0.770845189 | 0.772561475 | 0.774354059 | 0.77595881 | 0.777442959 | 0.779620921 | 0.782742828 | 0.787521693 | 0.792831247 | 0.796886453 | 0.800497839 | 0.804456534 | 0.809056345 | 0.813638203 | 0.818434112 | 0.821317017 | 0.823007864 |
| Rutland | 0.766192523 | 0.769723391 | 0.773297051 | 0.777004638 | 0.780391624 | 0.783018585 | 0.785485478 | 0.788347669 | 0.790914093 | 0.793736539 | 0.796912236 | 0.799624972 | 0.801611266 | 0.803284303 | 0.805247748 | 0.807235453 | 0.809351235 | 0.811666315 | 0.813742727 | 0.815829699 | 0.818645412 | 0.821760251 | 0.825659232 | 0.829914781 | 0.833405086 | 0.836430478 | 0.839437886 | 0.842656108 | 0.845782867 | 0.849379258 | 0.8514898 | 0.852733752 |
| East of England | 0.741478228 | 0.746266375 | 0.751345668 | 0.756660707 | 0.761739487 | 0.765640975 | 0.768889275 | 0.772612162 | 0.776766244 | 0.781857965 | 0.787303227 | 0.791887212 | 0.795240074 | 0.79792144 | 0.800875809 | 0.80356092 | 0.805885573 | 0.808246579 | 0.810698617 | 0.813175296 | 0.816027366 | 0.819220469 | 0.823543603 | 0.828460153 | 0.832433448 | 0.835920152 | 0.839563487 | 0.843623215 | 0.847672703 | 0.852132705 | 0.85481968 | 0.856407925 |
| Bedford | 0.751540717 | 0.756436515 | 0.761124416 | 0.766028513 | 0.770676544 | 0.774226978 | 0.77774583 | 0.78174237 | 0.785784479 | 0.79042273 | 0.79524673 | 0.799243534 | 0.802281541 | 0.804859857 | 0.807563466 | 0.810135036 | 0.812354243 | 0.814043648 | 0.815525816 | 0.81756501 | 0.820271515 | 0.823555758 | 0.827946504 | 0.832637385 | 0.836371317 | 0.839760894 | 0.843205461 | 0.84698617 | 0.850609173 | 0.854421023 | 0.856666658 | 0.858093661 |
| Cambridgeshire | 0.779009799 | 0.783985181 | 0.78906669 | 0.794080165 | 0.798821016 | 0.802652426 | 0.805838099 | 0.809458806 | 0.813666046 | 0.818714151 | 0.824005565 | 0.828590929 | 0.832097208 | 0.835138431 | 0.838174585 | 0.840904432 | 0.843373134 | 0.845774722 | 0.848307442 | 0.85060155 | 0.852942629 | 0.855628048 | 0.859359581 | 0.863546686 | 0.86697755 | 0.870164031 | 0.873583479 | 0.877288267 | 0.880884343 | 0.88467525 | 0.886772256 | 0.888027582 |
| Central Bedfordshire | 0.749851975 | 0.754455551 | 0.759106474 | 0.764070857 | 0.769062023 | 0.77297579 | 0.775964356 | 0.779029842 | 0.782147226 | 0.786078181 | 0.790706567 | 0.794790391 | 0.797868904 | 0.800106261 | 0.802332254 | 0.804306726 | 0.805960537 | 0.807573824 | 0.809286679 | 0.811126972 | 0.813412842 | 0.816407473 | 0.82064807 | 0.825812324 | 0.830245467 | 0.833837248 | 0.837331498 | 0.841045415 | 0.844643434 | 0.848596722 | 0.85088571 | 0.852254516 |
| Essex | 0.732582426 | 0.737593163 | 0.742876315 | 0.748384483 | 0.753574414 | 0.757518885 | 0.760760768 | 0.764430182 | 0.768444949 | 0.773439012 | 0.778835458 | 0.783515069 | 0.787080952 | 0.790062159 | 0.793205592 | 0.796084361 | 0.798648296 | 0.801242192 | 0.803699544 | 0.805858029 | 0.808217375 | 0.810884062 | 0.81473743 | 0.819163658 | 0.822688043 | 0.825748062 | 0.828893563 | 0.832599496 | 0.836565532 | 0.841099104 | 0.843867275 | 0.845491449 |
| Hertfordshire | 0.781615003 | 0.786069739 | 0.790941758 | 0.796102505 | 0.801069367 | 0.804952951 | 0.808196939 | 0.811891827 | 0.816073697 | 0.821142625 | 0.82676247 | 0.831867792 | 0.835831085 | 0.838924304 | 0.842055076 | 0.844603732 | 0.84659708 | 0.848364499 | 0.850030623 | 0.85160974 | 0.853790032 | 0.856412602 | 0.859987089 | 0.86411927 | 0.867548885 | 0.87067495 | 0.873957396 | 0.877450394 | 0.88076523 | 0.884292377 | 0.886293328 | 0.887510721 |
| Luton | 0.720456369 | 0.725553255 | 0.730739857 | 0.736365577 | 0.741805998 | 0.746002485 | 0.74981492 | 0.753976308 | 0.757898739 | 0.76232555 | 0.766866172 | 0.770498401 | 0.773653743 | 0.777124619 | 0.781049112 | 0.784700502 | 0.787898065 | 0.791414641 | 0.79524194 | 0.799161573 | 0.803258519 | 0.807371344 | 0.812255336 | 0.817039724 | 0.820106778 | 0.822610184 | 0.825647904 | 0.828878285 | 0.831745431 | 0.835519445 | 0.838085607 | 0.839717125 |
| Norfolk | 0.722519771 | 0.726932383 | 0.731763446 | 0.736990424 | 0.742026703 | 0.745919726 | 0.749228008 | 0.752979265 | 0.757142663 | 0.762415377 | 0.768263063 | 0.773034087 | 0.776163657 | 0.778388903 | 0.781071261 | 0.783559716 | 0.785822258 | 0.788223153 | 0.790811534 | 0.793312689 | 0.795797346 | 0.798340741 | 0.802117982 | 0.806733386 | 0.810577243 | 0.814099207 | 0.818085824 | 0.822737576 | 0.827369068 | 0.832396789 | 0.835502709 | 0.837309678 |
| Peterborough | 0.720906113 | 0.724486723 | 0.728629234 | 0.733591239 | 0.738835596 | 0.742688022 | 0.74577596 | 0.749385711 | 0.753029136 | 0.75782605 | 0.76296228 | 0.766882039 | 0.769346519 | 0.770841297 | 0.772552588 | 0.773912856 | 0.774654056 | 0.775492512 | 0.776510805 | 0.778466296 | 0.781671147 | 0.785331501 | 0.790819913 | 0.797776023 | 0.803503364 | 0.808450703 | 0.813724395 | 0.819607368 | 0.825283167 | 0.83148331 | 0.835615468 | 0.837874469 |
| Southend-on-Sea | 0.710768644 | 0.714455755 | 0.718963861 | 0.724101622 | 0.729301332 | 0.733065945 | 0.735870203 | 0.739467426 | 0.74388285 | 0.749656346 | 0.755767574 | 0.760872727 | 0.765072078 | 0.768484028 | 0.771741697 | 0.774015818 | 0.775534058 | 0.777425741 | 0.779667458 | 0.782494943 | 0.785759326 | 0.788706662 | 0.792478932 | 0.79723967 | 0.801283697 | 0.804525877 | 0.807862453 | 0.811934395 | 0.816279642 | 0.821165159 | 0.824137028 | 0.825801394 |
| Suffolk | 0.728635207 | 0.734038687 | 0.73963033 | 0.74494139 | 0.749561717 | 0.752729101 | 0.755145422 | 0.758265898 | 0.76219203 | 0.767158916 | 0.7723517 | 0.776420292 | 0.778871405 | 0.780547712 | 0.782982911 | 0.785366662 | 0.787307902 | 0.789178716 | 0.791208359 | 0.793531455 | 0.796429607 | 0.799990491 | 0.804967479 | 0.810596065 | 0.814992072 | 0.8187038 | 0.822460607 | 0.826718272 | 0.831140021 | 0.836167666 | 0.839325164 | 0.841151028 |
| Thurrock | 0.714583085 | 0.71931345 | 0.724233006 | 0.72995803 | 0.7359403 | 0.740257075 | 0.743684038 | 0.747559204 | 0.75183529 | 0.756916493 | 0.761798399 | 0.765080554 | 0.766925778 | 0.768085682 | 0.769558358 | 0.770781434 | 0.771561929 | 0.772446085 | 0.774190365 | 0.776873075 | 0.780155048 | 0.783491759 | 0.787488529 | 0.792094183 | 0.79558483 | 0.798345889 | 0.801489257 | 0.805430572 | 0.809719713 | 0.814642388 | 0.817763511 | 0.819597724 |
| Greater London | 0.79481868 | 0.799834703 | 0.805204307 | 0.810571153 | 0.815466559 | 0.819126417 | 0.822454571 | 0.826380411 | 0.830469675 | 0.835217016 | 0.840308832 | 0.844702107 | 0.848088378 | 0.850943486 | 0.854087714 | 0.857021818 | 0.859815601 | 0.862806931 | 0.865551894 | 0.868350839 | 0.871601022 | 0.875432469 | 0.880131568 | 0.8848343 | 0.888143302 | 0.890573067 | 0.892963026 | 0.89553814 | 0.898028841 | 0.900846103 | 0.90328554 | 0.904864758 |
| Barking and Dagenham | 0.684001119 | 0.688516583 | 0.694051852 | 0.700523075 | 0.707012651 | 0.711712654 | 0.715453534 | 0.720077271 | 0.725050347 | 0.730521788 | 0.735724234 | 0.739714043 | 0.742142354 | 0.743352218 | 0.744352367 | 0.744871221 | 0.745397899 | 0.746776337 | 0.748816592 | 0.751434823 | 0.754436368 | 0.758148172 | 0.764242078 | 0.771432178 | 0.776823469 | 0.78108087 | 0.785619711 | 0.791001107 | 0.796224781 | 0.80168939 | 0.805253667 | 0.807324954 |
| Barnet | 0.781612853 | 0.786012382 | 0.790506684 | 0.794961341 | 0.799470411 | 0.803264526 | 0.806863184 | 0.811078659 | 0.815559927 | 0.820742881 | 0.82620045 | 0.830788061 | 0.834138651 | 0.836765813 | 0.839301635 | 0.841622863 | 0.843827655 | 0.846045434 | 0.848109449 | 0.85015497 | 0.852997439 | 0.856626406 | 0.861265156 | 0.865948317 | 0.869282024 | 0.871835597 | 0.874261156 | 0.876975595 | 0.87976099 | 0.882986005 | 0.884817198 | 0.88597606 |
| Bexley | 0.725753639 | 0.731228465 | 0.736819341 | 0.742315968 | 0.74726382 | 0.750917475 | 0.754069305 | 0.757639668 | 0.761348499 | 0.766013793 | 0.77138492 | 0.776205501 | 0.779957943 | 0.782933435 | 0.78604034 | 0.788783665 | 0.79118262 | 0.793712339 | 0.796259699 | 0.798544635 | 0.800935453 | 0.804123632 | 0.809034117 | 0.81463145 | 0.819442437 | 0.823880888 | 0.828190516 | 0.832438529 | 0.836391727 | 0.84046774 | 0.842931859 | 0.844547626 |
| Brent | 0.754591719 | 0.758736052 | 0.763433359 | 0.768771548 | 0.773904287 | 0.777701948 | 0.781248313 | 0.785590309 | 0.789871932 | 0.794789662 | 0.800062065 | 0.804319183 | 0.807316064 | 0.809800323 | 0.812529348 | 0.814742813 | 0.816562737 | 0.818195653 | 0.819393826 | 0.820722691 | 0.82331879 | 0.826936448 | 0.83211444 | 0.837758591 | 0.84167643 | 0.844333173 | 0.846674559 | 0.849281463 | 0.852123101 | 0.855960764 | 0.858568469 | 0.860164338 |
| Bromley | 0.768353433 | 0.77275209 | 0.777301364 | 0.782008337 | 0.786466597 | 0.789948728 | 0.793321613 | 0.797411783 | 0.801741687 | 0.806731006 | 0.812085744 | 0.816720764 | 0.820129808 | 0.823040214 | 0.826092682 | 0.828746285 | 0.831031643 | 0.833241903 | 0.83510496 | 0.836406157 | 0.837556501 | 0.839455077 | 0.842869392 | 0.846827301 | 0.850139305 | 0.853031163 | 0.855860518 | 0.85911733 | 0.86254855 | 0.8661867 | 0.86822346 | 0.869545327 |
| Camden | 0.866757584 | 0.870073842 | 0.873487908 | 0.876929449 | 0.880212413 | 0.882802077 | 0.885330458 | 0.888101161 | 0.890852059 | 0.893707053 | 0.896506091 | 0.898966846 | 0.900985183 | 0.90281368 | 0.904957969 | 0.907042592 | 0.909098423 | 0.91143591 | 0.914046337 | 0.916612548 | 0.91892516 | 0.921484642 | 0.924195412 | 0.926615728 | 0.92829456 | 0.929347575 | 0.930402756 | 0.931612604 | 0.932908341 | 0.93428657 | 0.935492642 | 0.936474834 |
| Croydon | 0.749157558 | 0.754470413 | 0.760135211 | 0.765609579 | 0.770455557 | 0.773919422 | 0.776817337 | 0.780426701 | 0.784393556 | 0.78928507 | 0.794810602 | 0.799443932 | 0.802651985 | 0.804938594 | 0.807573997 | 0.809791123 | 0.811435516 | 0.813212207 | 0.814844078 | 0.816257397 | 0.818208512 | 0.820724489 | 0.824412923 | 0.82849933 | 0.831280418 | 0.833557122 | 0.836455352 | 0.840121037 | 0.843843228 | 0.848055613 | 0.850621011 | 0.852066336 |
| Ealing | 0.776440874 | 0.780781212 | 0.785687945 | 0.790925528 | 0.795884412 | 0.79991058 | 0.803736232 | 0.808046513 | 0.812508974 | 0.81758411 | 0.822898908 | 0.827395299 | 0.830786452 | 0.833247078 | 0.835595927 | 0.837380747 | 0.838690188 | 0.839891274 | 0.840934617 | 0.841938299 | 0.843771099 | 0.846937413 | 0.851884051 | 0.857483803 | 0.861983934 | 0.865745232 | 0.869263303 | 0.872819689 | 0.876113721 | 0.879541502 | 0.881496434 | 0.88272888 |
| Enfield | 0.734015063 | 0.7388007 | 0.744266862 | 0.749820519 | 0.754511903 | 0.757858488 | 0.761079071 | 0.765104673 | 0.769544589 | 0.774731301 | 0.780137168 | 0.784391493 | 0.787405618 | 0.789760749 | 0.792185723 | 0.794356682 | 0.796352427 | 0.798514562 | 0.800925074 | 0.80349802 | 0.806899456 | 0.810996499 | 0.816221135 | 0.821709962 | 0.8255965 | 0.828647236 | 0.831858692 | 0.835400396 | 0.838825766 | 0.842652738 | 0.845003032 | 0.846461002 |
| Greenwich | 0.724794432 | 0.730568978 | 0.737056996 | 0.743585009 | 0.749387751 | 0.753646856 | 0.757402944 | 0.761515247 | 0.765732658 | 0.770821244 | 0.776319665 | 0.780942856 | 0.784275935 | 0.786599546 | 0.788917323 | 0.790983564 | 0.79330042 | 0.796377234 | 0.799502468 | 0.802184767 | 0.805060295 | 0.808882631 | 0.814368078 | 0.820243137 | 0.824489796 | 0.827849159 | 0.831340992 | 0.835098823 | 0.838711207 | 0.842540743 | 0.844750971 | 0.846080185 |
| Hackney | 0.772855945 | 0.780149291 | 0.788048608 | 0.794849376 | 0.799756997 | 0.801617428 | 0.802540605 | 0.804391122 | 0.806670726 | 0.810459046 | 0.814712579 | 0.818153404 | 0.820828658 | 0.823265809 | 0.826833933 | 0.830824549 | 0.835303266 | 0.840429707 | 0.844971841 | 0.849740986 | 0.855414377 | 0.861393137 | 0.867708973 | 0.873396387 | 0.876716306 | 0.878777684 | 0.880631495 | 0.882688413 | 0.884880596 | 0.888062194 | 0.891057352 | 0.892946278 |
| Hammersmith and Fulham | 0.84480445 | 0.849996132 | 0.855195257 | 0.860183244 | 0.864779843 | 0.868625952 | 0.8722719 | 0.876150721 | 0.880063755 | 0.884634222 | 0.889023544 | 0.891501813 | 0.893488656 | 0.895404122 | 0.897540324 | 0.899710637 | 0.90205138 | 0.904527845 | 0.906952059 | 0.90956889 | 0.912392154 | 0.91529824 | 0.918367904 | 0.921452527 | 0.923784134 | 0.925597589 | 0.927323964 | 0.929063315 | 0.930742036 | 0.932555918 | 0.934089147 | 0.935153171 |
| Haringey | 0.762031142 | 0.766734225 | 0.771662106 | 0.776384587 | 0.78046201 | 0.783315684 | 0.786091474 | 0.789701982 | 0.793422832 | 0.797741588 | 0.802629398 | 0.807326894 | 0.811040185 | 0.814043712 | 0.817316211 | 0.820322514 | 0.822829932 | 0.825255057 | 0.827362145 | 0.829266291 | 0.832249982 | 0.83617217 | 0.840791267 | 0.845567385 | 0.849215615 | 0.852382214 | 0.855969725 | 0.860147561 | 0.864232341 | 0.868452764 | 0.870780607 | 0.872084552 |
| Harrow | 0.76590342 | 0.770692636 | 0.775646349 | 0.780600528 | 0.785093902 | 0.788395101 | 0.79148835 | 0.795565116 | 0.800355253 | 0.805905431 | 0.811450755 | 0.815848364 | 0.818862918 | 0.821388384 | 0.824674616 | 0.827689066 | 0.830205345 | 0.832428723 | 0.834273205 | 0.835620566 | 0.837416895 | 0.839869092 | 0.843248141 | 0.846769317 | 0.848853968 | 0.849999225 | 0.850833224 | 0.85201227 | 0.853711866 | 0.856608609 | 0.858637704 | 0.860015269 |
| Havering | 0.718170603 | 0.723397767 | 0.728622374 | 0.733407867 | 0.737906412 | 0.741479928 | 0.744618622 | 0.748109635 | 0.752005979 | 0.757329069 | 0.763115405 | 0.767967754 | 0.771557279 | 0.774223035 | 0.777642388 | 0.781309185 | 0.784500105 | 0.787305189 | 0.790058014 | 0.79238045 | 0.794807248 | 0.79793974 | 0.802207089 | 0.806728504 | 0.810232085 | 0.813537348 | 0.817157436 | 0.821197014 | 0.825368845 | 0.829939392 | 0.832796681 | 0.834643869 |
| Hillingdon | 0.783602665 | 0.78858177 | 0.794126231 | 0.799479633 | 0.804346255 | 0.807977966 | 0.811165388 | 0.815091125 | 0.819769381 | 0.825370586 | 0.831137578 | 0.835938954 | 0.839583791 | 0.842037991 | 0.844176116 | 0.8459004 | 0.8473667 | 0.849010908 | 0.851046786 | 0.8538497 | 0.856916806 | 0.86043264 | 0.864965694 | 0.870203067 | 0.874231363 | 0.876922084 | 0.87948897 | 0.882356486 | 0.885268198 | 0.88848906 | 0.891268975 | 0.893138131 |
| Hounslow | 0.777744991 | 0.782777713 | 0.78797048 | 0.793088964 | 0.797766026 | 0.801045775 | 0.803720178 | 0.807413818 | 0.811981973 | 0.817880504 | 0.824203579 | 0.829126097 | 0.832684607 | 0.83526961 | 0.837971143 | 0.840481064 | 0.842635638 | 0.844844527 | 0.846988295 | 0.848873541 | 0.851382651 | 0.855090437 | 0.860640988 | 0.866954272 | 0.872069889 | 0.876418971 | 0.880660089 | 0.884934879 | 0.888851892 | 0.893006464 | 0.895535228 | 0.896994582 |
| Islington | 0.829730557 | 0.834538971 | 0.840074285 | 0.845991632 | 0.851344046 | 0.855202575 | 0.858405045 | 0.861427577 | 0.864283728 | 0.867619926 | 0.871507061 | 0.875083239 | 0.87763267 | 0.879841388 | 0.882371517 | 0.884922497 | 0.887621895 | 0.890573454 | 0.893724543 | 0.897186816 | 0.900716085 | 0.904193451 | 0.907506447 | 0.910313878 | 0.91232004 | 0.914041333 | 0.915757543 | 0.917630147 | 0.919612034 | 0.921755199 | 0.923529309 | 0.924740074 |
| Kensington and Chelsea | 0.87554948 | 0.879909108 | 0.884016873 | 0.887919316 | 0.891609781 | 0.894876949 | 0.89819944 | 0.901878354 | 0.90481233 | 0.906671066 | 0.908431894 | 0.910065595 | 0.911509546 | 0.912965312 | 0.914709201 | 0.916417012 | 0.918346772 | 0.920480103 | 0.922728206 | 0.925097508 | 0.927502151 | 0.930094056 | 0.932646449 | 0.934899887 | 0.936585872 | 0.937966481 | 0.939521727 | 0.941162574 | 0.942668513 | 0.944205752 | 0.945506136 | 0.946476699 |
| Kingston upon Thames | 0.811664993 | 0.816506309 | 0.821409292 | 0.825913128 | 0.830163346 | 0.833946915 | 0.837507878 | 0.841469241 | 0.845810942 | 0.850801759 | 0.856033529 | 0.86055739 | 0.864118277 | 0.867042389 | 0.870061455 | 0.872963876 | 0.875770088 | 0.878704304 | 0.881669795 | 0.883962988 | 0.886115962 | 0.888299312 | 0.890812567 | 0.893262004 | 0.894904635 | 0.896362442 | 0.898285289 | 0.900802182 | 0.903476265 | 0.906374438 | 0.907803889 | 0.908698276 |
| Lambeth | 0.787597726 | 0.793585528 | 0.799554012 | 0.805240529 | 0.810384225 | 0.814263416 | 0.817846569 | 0.822277641 | 0.826880899 | 0.832010865 | 0.837526186 | 0.84236157 | 0.846147671 | 0.849581414 | 0.853629409 | 0.857704941 | 0.861835364 | 0.866160365 | 0.870256382 | 0.874099448 | 0.878356779 | 0.882821935 | 0.887754283 | 0.892752941 | 0.896725702 | 0.900141632 | 0.903432601 | 0.906859706 | 0.910166393 | 0.913563581 | 0.915357834 | 0.916380817 |
| Lewisham | 0.741501455 | 0.746637661 | 0.75223091 | 0.757916286 | 0.763039852 | 0.766982601 | 0.770342927 | 0.774216109 | 0.778468843 | 0.783543336 | 0.789280503 | 0.79422735 | 0.79790394 | 0.800666579 | 0.803321085 | 0.805534778 | 0.807697155 | 0.810063111 | 0.812499824 | 0.814925803 | 0.817939091 | 0.821589346 | 0.826608209 | 0.832134298 | 0.836393139 | 0.839839363 | 0.843239201 | 0.846805101 | 0.850232111 | 0.853955562 | 0.856070393 | 0.857308935 |
| Merton | 0.774133503 | 0.779264091 | 0.784517026 | 0.789931319 | 0.795075297 | 0.799208115 | 0.802983981 | 0.807597715 | 0.812524816 | 0.81785755 | 0.823308839 | 0.827860229 | 0.831187029 | 0.833488952 | 0.835766046 | 0.837672924 | 0.839476873 | 0.841821372 | 0.844486468 | 0.846735014 | 0.849230797 | 0.85250906 | 0.857340914 | 0.862598018 | 0.866644751 | 0.870057239 | 0.873361498 | 0.876737437 | 0.880135011 | 0.884133481 | 0.886501588 | 0.887733957 |
| Newham | 0.701795321 | 0.706435607 | 0.711724521 | 0.717248995 | 0.722188557 | 0.725423043 | 0.728685786 | 0.733076299 | 0.737392944 | 0.742819967 | 0.749382822 | 0.755500384 | 0.760511692 | 0.764926699 | 0.769471639 | 0.773306251 | 0.776430433 | 0.7797725 | 0.783017826 | 0.786554114 | 0.791452624 | 0.797446191 | 0.80471522 | 0.811923568 | 0.817120439 | 0.821113561 | 0.825009485 | 0.829166845 | 0.83304735 | 0.837329112 | 0.840068005 | 0.841688128 |
| Redbridge | 0.741118203 | 0.745625644 | 0.750744848 | 0.756022404 | 0.760918122 | 0.764646431 | 0.768051696 | 0.772273 | 0.776802421 | 0.781831216 | 0.787231364 | 0.79189485 | 0.795472872 | 0.798415222 | 0.801404001 | 0.803831523 | 0.806182488 | 0.808872572 | 0.811032991 | 0.812479614 | 0.814546327 | 0.817725215 | 0.822531963 | 0.827775206 | 0.831557908 | 0.834366109 | 0.8371229 | 0.840194082 | 0.843207872 | 0.846793217 | 0.848962076 | 0.850265079 |
| Richmond upon Thames | 0.837032814 | 0.841391747 | 0.84569853 | 0.84989642 | 0.853761379 | 0.857003294 | 0.860178395 | 0.863807321 | 0.867759476 | 0.872359082 | 0.877172936 | 0.88124833 | 0.884347533 | 0.886895017 | 0.889383741 | 0.891544358 | 0.893595171 | 0.895814266 | 0.897952784 | 0.899598982 | 0.901637922 | 0.9043754 | 0.907731989 | 0.911396634 | 0.914650045 | 0.917727441 | 0.920772011 | 0.923914496 | 0.926952638 | 0.929956533 | 0.931324466 | 0.932132548 |
| Southwark | 0.797907534 | 0.803926439 | 0.810137544 | 0.816098289 | 0.821544009 | 0.825849944 | 0.829764819 | 0.834456408 | 0.839649071 | 0.845546146 | 0.85185331 | 0.857230683 | 0.861269303 | 0.864736199 | 0.867536763 | 0.869900661 | 0.872527573 | 0.875686653 | 0.879263889 | 0.883064359 | 0.887097401 | 0.891620598 | 0.896426382 | 0.900778404 | 0.903809107 | 0.906064807 | 0.908359882 | 0.910951805 | 0.913457165 | 0.916052895 | 0.918164679 | 0.919509828 |
| Sutton | 0.745539692 | 0.750322856 | 0.755413986 | 0.760752139 | 0.76603767 | 0.770410677 | 0.774311397 | 0.778686753 | 0.783260471 | 0.788775107 | 0.794821411 | 0.799725587 | 0.803264865 | 0.806048382 | 0.80871934 | 0.81083275 | 0.812660097 | 0.814678049 | 0.816762697 | 0.818754559 | 0.821181816 | 0.824332169 | 0.828959736 | 0.834131452 | 0.838360085 | 0.841903925 | 0.845245326 | 0.848536334 | 0.851572536 | 0.855036272 | 0.85711221 | 0.858421577 |
| Tower Hamlets | 0.765109358 | 0.771711626 | 0.778966354 | 0.786159164 | 0.792573377 | 0.797321441 | 0.802062544 | 0.807837588 | 0.813677375 | 0.81971193 | 0.825601011 | 0.830492016 | 0.834775637 | 0.839143327 | 0.844292365 | 0.849343432 | 0.853982287 | 0.858413384 | 0.86236273 | 0.866672637 | 0.87145478 | 0.876244382 | 0.88124895 | 0.886032761 | 0.889448237 | 0.891959749 | 0.894271385 | 0.896664014 | 0.89879702 | 0.901051033 | 0.903082005 | 0.904488141 |
| Waltham Forest | 0.716668274 | 0.72145392 | 0.727050373 | 0.733124582 | 0.738929149 | 0.743403588 | 0.747381584 | 0.751791267 | 0.756269456 | 0.76142493 | 0.766923015 | 0.771588189 | 0.77497571 | 0.777633641 | 0.780520142 | 0.782972952 | 0.784907508 | 0.786786017 | 0.78857743 | 0.790421126 | 0.793057618 | 0.796828937 | 0.802519656 | 0.809104344 | 0.814502386 | 0.81900025 | 0.823304672 | 0.827785924 | 0.832043458 | 0.836442839 | 0.83902216 | 0.840509602 |
| Wandsworth | 0.820916201 | 0.825894317 | 0.831010381 | 0.836144422 | 0.84099764 | 0.845261428 | 0.849505433 | 0.85423898 | 0.858944733 | 0.86399287 | 0.869303621 | 0.874076766 | 0.878067327 | 0.881463943 | 0.884724892 | 0.887652111 | 0.890511556 | 0.893444078 | 0.896206385 | 0.898373803 | 0.900524981 | 0.902869921 | 0.905725139 | 0.908819701 | 0.911294986 | 0.913467326 | 0.915719805 | 0.918062236 | 0.920335562 | 0.922738996 | 0.923703881 | 0.924241493 |
| Westminster | 0.860957245 | 0.864667843 | 0.86896405 | 0.873169724 | 0.876962898 | 0.879974952 | 0.882941481 | 0.885943119 | 0.888570465 | 0.891199613 | 0.893950243 | 0.896558965 | 0.898616696 | 0.900460189 | 0.902514709 | 0.904645511 | 0.907035241 | 0.909662413 | 0.912355807 | 0.914728286 | 0.916429199 | 0.918391465 | 0.921137933 | 0.923914956 | 0.925955012 | 0.927561927 | 0.929279663 | 0.931141661 | 0.932865986 | 0.934622267 | 0.936117972 | 0.937198627 |
| North East England | 0.705613776 | 0.70969006 | 0.715108428 | 0.721157918 | 0.726624022 | 0.730569673 | 0.73413026 | 0.738518133 | 0.743378297 | 0.749166653 | 0.755176562 | 0.760000916 | 0.763442093 | 0.766109249 | 0.769008428 | 0.771810523 | 0.774601757 | 0.777616493 | 0.780491791 | 0.783069614 | 0.786152055 | 0.790036667 | 0.795401926 | 0.801074387 | 0.80511943 | 0.808296694 | 0.81151855 | 0.815149277 | 0.818802097 | 0.82310774 | 0.825712585 | 0.827273029 |
| County Durham | 0.698702667 | 0.702094932 | 0.707437931 | 0.713686849 | 0.718932791 | 0.722237487 | 0.72504864 | 0.728821377 | 0.733442582 | 0.739289883 | 0.745185939 | 0.74970548 | 0.752717306 | 0.754906886 | 0.757469437 | 0.760126806 | 0.762500708 | 0.765183319 | 0.768098456 | 0.77067482 | 0.77361282 | 0.777441237 | 0.782602437 | 0.787976163 | 0.791603989 | 0.794454886 | 0.797555429 | 0.801012935 | 0.804297085 | 0.808205035 | 0.810719064 | 0.812276525 |
| Darlington | 0.713740668 | 0.717208445 | 0.722525017 | 0.728664363 | 0.734149787 | 0.737707345 | 0.740342727 | 0.743345895 | 0.74677718 | 0.752013583 | 0.757750312 | 0.761646681 | 0.764189885 | 0.766353943 | 0.769520119 | 0.772730957 | 0.775609422 | 0.778011416 | 0.779631777 | 0.781595515 | 0.785202302 | 0.790023441 | 0.796884576 | 0.804578287 | 0.810375696 | 0.815141909 | 0.819636777 | 0.824069115 | 0.828074765 | 0.832545835 | 0.835467512 | 0.837204088 |
| Gateshead | 0.707597434 | 0.711674996 | 0.716966528 | 0.722399178 | 0.72717075 | 0.730390263 | 0.733155036 | 0.737095049 | 0.741590115 | 0.746754291 | 0.752343205 | 0.757313756 | 0.761221342 | 0.764454653 | 0.767918951 | 0.771240265 | 0.774296987 | 0.777132916 | 0.779451333 | 0.782481028 | 0.787148756 | 0.792382446 | 0.798337243 | 0.804199045 | 0.808221695 | 0.811473097 | 0.815139152 | 0.819229905 | 0.822581149 | 0.826232364 | 0.828663428 | 0.830244183 |
| Hartlepool | 0.676173399 | 0.679522189 | 0.683978451 | 0.688914886 | 0.693795603 | 0.697925929 | 0.701718428 | 0.705386794 | 0.708784926 | 0.71319269 | 0.718454608 | 0.722823493 | 0.725628981 | 0.727301313 | 0.729160089 | 0.730822385 | 0.733132712 | 0.736274968 | 0.739047383 | 0.741306198 | 0.744135612 | 0.748735428 | 0.756549146 | 0.764932733 | 0.770583365 | 0.774760866 | 0.778986561 | 0.783634694 | 0.788412632 | 0.793676592 | 0.796959004 | 0.798877773 |
| Middlesbrough | 0.686457603 | 0.690513802 | 0.695091863 | 0.700220906 | 0.705304231 | 0.709086957 | 0.712340786 | 0.716402145 | 0.720571197 | 0.725772294 | 0.731968226 | 0.736889814 | 0.739632523 | 0.741490813 | 0.744150859 | 0.747498005 | 0.75101106 | 0.754776002 | 0.757897126 | 0.759848693 | 0.762010424 | 0.765351482 | 0.770783087 | 0.776412826 | 0.780041152 | 0.782493491 | 0.784984984 | 0.788126859 | 0.791578304 | 0.79618331 | 0.799434822 | 0.801470999 |
| Newcastle upon Tyne | 0.751971871 | 0.756852693 | 0.762151849 | 0.767520106 | 0.77259528 | 0.776904285 | 0.781153688 | 0.786083077 | 0.791241968 | 0.796583502 | 0.802023959 | 0.80722356 | 0.812290271 | 0.817159297 | 0.821959788 | 0.826255258 | 0.830527303 | 0.835103999 | 0.839617346 | 0.84325483 | 0.84616954 | 0.849049079 | 0.852482019 | 0.855853278 | 0.858365926 | 0.860224349 | 0.861883508 | 0.863985284 | 0.866414854 | 0.869473942 | 0.871307093 | 0.872520663 |
| North Tyneside | 0.711450488 | 0.715545308 | 0.721175663 | 0.727445092 | 0.732544195 | 0.735868226 | 0.738895127 | 0.742604096 | 0.746971489 | 0.752173036 | 0.757655003 | 0.762267849 | 0.765716992 | 0.768637184 | 0.77204743 | 0.775655462 | 0.779320079 | 0.782446475 | 0.784972202 | 0.787437856 | 0.790837035 | 0.795349117 | 0.801506147 | 0.807833849 | 0.81251038 | 0.816416503 | 0.820096837 | 0.82406908 | 0.828044092 | 0.832298325 | 0.834834388 | 0.836400602 |
| Northumberland | 0.711789114 | 0.714907396 | 0.71927485 | 0.724587736 | 0.729679695 | 0.733250929 | 0.73626221 | 0.739826495 | 0.743823392 | 0.749123568 | 0.754330997 | 0.758337401 | 0.76108912 | 0.763186112 | 0.765806193 | 0.768651508 | 0.771410197 | 0.773875717 | 0.775862776 | 0.777862075 | 0.780777441 | 0.784316658 | 0.789678587 | 0.795588108 | 0.799634044 | 0.803131067 | 0.806770097 | 0.810609074 | 0.814486699 | 0.819024255 | 0.821776692 | 0.823317013 |
| Redcar and Cleveland | 0.683036341 | 0.685800422 | 0.690393576 | 0.696219709 | 0.701839301 | 0.705764239 | 0.709095171 | 0.713212033 | 0.71757085 | 0.722753167 | 0.727723661 | 0.731255086 | 0.733395434 | 0.73446566 | 0.736413995 | 0.73919644 | 0.742318653 | 0.74511908 | 0.747207735 | 0.748600396 | 0.750594255 | 0.754449909 | 0.761395089 | 0.769234248 | 0.774515592 | 0.777728754 | 0.780759877 | 0.78427063 | 0.78810445 | 0.793106964 | 0.796445438 | 0.798369621 |
| South Tyneside | 0.674543573 | 0.678543978 | 0.683716248 | 0.689358644 | 0.694396754 | 0.698025126 | 0.701471242 | 0.705756197 | 0.710404589 | 0.716041346 | 0.721991767 | 0.726915635 | 0.730522724 | 0.733589751 | 0.737080441 | 0.740235184 | 0.742760907 | 0.745517892 | 0.748420801 | 0.750830253 | 0.753711715 | 0.757811442 | 0.763910283 | 0.770758985 | 0.77581579 | 0.779844319 | 0.783805944 | 0.787983013 | 0.792015872 | 0.796529018 | 0.79932541 | 0.801073026 |
| Stockton-on-Tees | 0.716320949 | 0.721109506 | 0.727228349 | 0.734102101 | 0.740372526 | 0.744452093 | 0.747815414 | 0.751862566 | 0.75599085 | 0.760936797 | 0.765919021 | 0.769334213 | 0.771376739 | 0.772840828 | 0.774613645 | 0.776208773 | 0.778166817 | 0.780183261 | 0.781800591 | 0.783969216 | 0.787353426 | 0.791767172 | 0.797836441 | 0.804118216 | 0.80850829 | 0.811700648 | 0.8149351 | 0.818426863 | 0.821877617 | 0.826308399 | 0.829310547 | 0.83098489 |
| Sunderland | 0.693436907 | 0.697931364 | 0.704371173 | 0.711319643 | 0.717271466 | 0.721360113 | 0.724995257 | 0.729357509 | 0.734016404 | 0.739538447 | 0.745358267 | 0.749970409 | 0.753294074 | 0.75609381 | 0.759041443 | 0.761348916 | 0.763720521 | 0.766614451 | 0.769615273 | 0.772412295 | 0.775500642 | 0.779478282 | 0.784977472 | 0.790780202 | 0.795064335 | 0.798394812 | 0.801673547 | 0.805482239 | 0.809538529 | 0.814268145 | 0.817309606 | 0.819164125 |
| North West England | 0.723853024 | 0.728909506 | 0.734960051 | 0.741129046 | 0.746459648 | 0.74992915 | 0.752791702 | 0.756477484 | 0.760650507 | 0.76598297 | 0.771824098 | 0.776643033 | 0.779849293 | 0.782021541 | 0.784604298 | 0.787248288 | 0.789809138 | 0.79258226 | 0.795403416 | 0.798260117 | 0.80162241 | 0.805513604 | 0.810760519 | 0.816470961 | 0.820805929 | 0.824405824 | 0.828005972 | 0.831952508 | 0.835829626 | 0.84018255 | 0.842847775 | 0.844443823 |
| Blackburn with Darwen | 0.68238976 | 0.685731636 | 0.690008092 | 0.695005459 | 0.699698108 | 0.702632539 | 0.7053888 | 0.70887123 | 0.712786846 | 0.718095097 | 0.723811203 | 0.728312718 | 0.731476372 | 0.733717688 | 0.736408195 | 0.739046307 | 0.741857507 | 0.744687186 | 0.746840928 | 0.749452074 | 0.753781345 | 0.759122509 | 0.766711821 | 0.775469564 | 0.782017271 | 0.786917314 | 0.791236947 | 0.795486741 | 0.800059739 | 0.805725324 | 0.809425808 | 0.811503741 |
| Blackpool | 0.675252664 | 0.679927587 | 0.68518121 | 0.690174524 | 0.694593438 | 0.697103334 | 0.69907228 | 0.70250716 | 0.706827137 | 0.712501767 | 0.718462526 | 0.7224849 | 0.723744704 | 0.723430315 | 0.724603021 | 0.726702513 | 0.728852988 | 0.73089932 | 0.732814533 | 0.735078985 | 0.738029927 | 0.741426861 | 0.746846954 | 0.753262728 | 0.758165273 | 0.762367109 | 0.766958763 | 0.7723135 | 0.777595117 | 0.783557348 | 0.787585853 | 0.789925893 |
| Bolton | 0.701717327 | 0.707123313 | 0.713437318 | 0.719670447 | 0.724604349 | 0.727095634 | 0.728802704 | 0.731464409 | 0.734662473 | 0.739275432 | 0.744331856 | 0.74837426 | 0.751013687 | 0.752478869 | 0.754290275 | 0.756010401 | 0.757730827 | 0.759344024 | 0.760567007 | 0.762437589 | 0.76605381 | 0.770797743 | 0.777362551 | 0.784333907 | 0.789117194 | 0.79270227 | 0.796294174 | 0.80056448 | 0.804712977 | 0.809306337 | 0.812340765 | 0.814170764 |
| Bury | 0.717064481 | 0.722315648 | 0.728541718 | 0.734837387 | 0.740145419 | 0.743550793 | 0.74612714 | 0.749191337 | 0.752662934 | 0.757184989 | 0.76184501 | 0.765389349 | 0.767550598 | 0.768977564 | 0.771177019 | 0.773710054 | 0.775845384 | 0.777829378 | 0.779430434 | 0.780918713 | 0.78349505 | 0.787599835 | 0.793815211 | 0.800715332 | 0.806043627 | 0.810299015 | 0.814202249 | 0.818155045 | 0.82186126 | 0.826019123 | 0.8285613 | 0.830089162 |
| Cheshire East | 0.771674646 | 0.776171884 | 0.781368716 | 0.786590269 | 0.791325813 | 0.794808609 | 0.797632724 | 0.800895963 | 0.804325731 | 0.808972433 | 0.814183073 | 0.818550682 | 0.821588382 | 0.82352578 | 0.825763879 | 0.827783328 | 0.829838065 | 0.832133621 | 0.834333428 | 0.836359447 | 0.838921113 | 0.842318573 | 0.847573799 | 0.853657247 | 0.858395056 | 0.862538482 | 0.866637335 | 0.871197954 | 0.875820368 | 0.88048343 | 0.883191175 | 0.884754634 |
| Cheshire West and Chester | 0.759102313 | 0.764448654 | 0.770695945 | 0.776715784 | 0.781653165 | 0.784738117 | 0.787267683 | 0.790772656 | 0.794815905 | 0.799793841 | 0.804976915 | 0.809351665 | 0.812567479 | 0.814711768 | 0.817159505 | 0.819898683 | 0.822702703 | 0.825791288 | 0.82895026 | 0.831416172 | 0.833802505 | 0.836774227 | 0.841383531 | 0.846585442 | 0.850695077 | 0.854135841 | 0.857434235 | 0.860794971 | 0.86387828 | 0.867508876 | 0.869780195 | 0.871177933 |
| Cumbria | 0.723302241 | 0.726945661 | 0.732305222 | 0.738372222 | 0.743815552 | 0.747052396 | 0.749318145 | 0.752561756 | 0.756270732 | 0.760885712 | 0.765989836 | 0.770097987 | 0.772497057 | 0.773996336 | 0.776451856 | 0.778915922 | 0.781258368 | 0.783839498 | 0.786572789 | 0.789527384 | 0.793116736 | 0.797366439 | 0.803686986 | 0.81079256 | 0.816323168 | 0.821124453 | 0.825768065 | 0.830456314 | 0.834725103 | 0.839082726 | 0.841776363 | 0.843466901 |
| Halton | 0.704162024 | 0.709000642 | 0.715316123 | 0.722218377 | 0.7283098 | 0.732239076 | 0.734714285 | 0.736727017 | 0.739207013 | 0.743894948 | 0.749475696 | 0.754275431 | 0.757265995 | 0.758487164 | 0.76002317 | 0.762286706 | 0.765261702 | 0.768623765 | 0.771956576 | 0.775317187 | 0.77925367 | 0.783471535 | 0.789229918 | 0.796248387 | 0.802257922 | 0.807465606 | 0.812771359 | 0.818551638 | 0.824143615 | 0.830222403 | 0.834099527 | 0.836181349 |
| Knowsley | 0.68322195 | 0.687997471 | 0.693783342 | 0.698891684 | 0.703117436 | 0.706171182 | 0.709475536 | 0.713689691 | 0.717494486 | 0.722184627 | 0.728717107 | 0.7351277 | 0.739114058 | 0.741111167 | 0.743251296 | 0.746332754 | 0.749654893 | 0.753227834 | 0.757286083 | 0.76104959 | 0.764315712 | 0.768288027 | 0.774237182 | 0.780609465 | 0.785457454 | 0.789482569 | 0.793408857 | 0.797687514 | 0.802114147 | 0.807477929 | 0.811323511 | 0.813671711 |
| Lancashire | 0.727956978 | 0.733591254 | 0.740115399 | 0.746543407 | 0.751957131 | 0.755326739 | 0.757928218 | 0.761319536 | 0.765075881 | 0.769850615 | 0.774914844 | 0.779092606 | 0.781590828 | 0.783009642 | 0.785146588 | 0.787683128 | 0.790070493 | 0.792556962 | 0.795303407 | 0.798417268 | 0.802044573 | 0.806072389 | 0.811097338 | 0.816185653 | 0.819833333 | 0.822993117 | 0.82627369 | 0.829847655 | 0.833322126 | 0.837211371 | 0.839613677 | 0.841125535 |
| Liverpool | 0.725467323 | 0.731475935 | 0.737600847 | 0.743305292 | 0.748057395 | 0.751380354 | 0.754931797 | 0.75970248 | 0.765176947 | 0.77130966 | 0.777699859 | 0.783445457 | 0.788125246 | 0.792469092 | 0.79693049 | 0.801091401 | 0.805467924 | 0.810133238 | 0.814687452 | 0.818301894 | 0.821097702 | 0.82391341 | 0.82742119 | 0.830944112 | 0.833379699 | 0.835090888 | 0.836921635 | 0.839128076 | 0.841444206 | 0.844783292 | 0.847036463 | 0.848437635 |
| Manchester | 0.746647615 | 0.751705623 | 0.757925193 | 0.764651684 | 0.77085982 | 0.775441989 | 0.77948319 | 0.784412505 | 0.790043864 | 0.796729323 | 0.803637854 | 0.809553972 | 0.814037951 | 0.817623439 | 0.821526324 | 0.825619567 | 0.829883918 | 0.83485281 | 0.840257627 | 0.845101398 | 0.848986389 | 0.85230152 | 0.856079222 | 0.860063972 | 0.863211515 | 0.865984421 | 0.86882049 | 0.871906818 | 0.874834613 | 0.878140602 | 0.880157636 | 0.881429144 |
| Oldham | 0.680659819 | 0.684881077 | 0.690479928 | 0.696535421 | 0.70102578 | 0.703103672 | 0.704684602 | 0.707004718 | 0.709396487 | 0.713174671 | 0.717805545 | 0.721454702 | 0.724039294 | 0.725876998 | 0.728265089 | 0.73060863 | 0.732777929 | 0.735001899 | 0.736237572 | 0.738193611 | 0.742825897 | 0.748985857 | 0.756691331 | 0.76466026 | 0.770258209 | 0.774129288 | 0.777678707 | 0.782140863 | 0.786946053 | 0.792524204 | 0.79625616 | 0.798408293 |
| Rochdale | 0.682495148 | 0.686914997 | 0.692740297 | 0.69886136 | 0.70449136 | 0.70786881 | 0.710105757 | 0.713178503 | 0.716891 | 0.722227937 | 0.727995529 | 0.732233488 | 0.734856617 | 0.736711125 | 0.738933716 | 0.741031709 | 0.742513408 | 0.744115452 | 0.745705587 | 0.747775543 | 0.751571519 | 0.756799464 | 0.764057223 | 0.771788263 | 0.777208866 | 0.78100561 | 0.784406065 | 0.788137396 | 0.791992543 | 0.796814439 | 0.800110079 | 0.802069322 |
| Salford | 0.707740126 | 0.712145618 | 0.718390732 | 0.725411036 | 0.731641191 | 0.735455032 | 0.737696 | 0.740745454 | 0.745601195 | 0.75253971 | 0.759734519 | 0.765483161 | 0.769876783 | 0.773204342 | 0.776433961 | 0.779505268 | 0.782575351 | 0.785692024 | 0.78908628 | 0.792573243 | 0.795918834 | 0.799408117 | 0.804479518 | 0.810154795 | 0.813959968 | 0.816918301 | 0.820542217 | 0.824786789 | 0.828912525 | 0.833671744 | 0.836849261 | 0.838699172 |
| Sefton | 0.718750428 | 0.724685208 | 0.73131607 | 0.737480071 | 0.742595906 | 0.745922692 | 0.748511595 | 0.751985561 | 0.756073253 | 0.760803623 | 0.765775157 | 0.769983999 | 0.773000896 | 0.775567172 | 0.77886283 | 0.781784567 | 0.784112471 | 0.786298296 | 0.78800487 | 0.789423362 | 0.791196082 | 0.79306048 | 0.796215479 | 0.800465642 | 0.803998119 | 0.807095845 | 0.810138665 | 0.813683706 | 0.817535947 | 0.821863859 | 0.824386421 | 0.825841895 |
| St Helens | 0.694233681 | 0.69856037 | 0.704405114 | 0.710740518 | 0.71592096 | 0.718794057 | 0.721045314 | 0.723957516 | 0.727473651 | 0.732620088 | 0.738580068 | 0.74341145 | 0.746254479 | 0.74828049 | 0.751073309 | 0.753413074 | 0.755518714 | 0.758137633 | 0.760877168 | 0.763978505 | 0.767673701 | 0.771753897 | 0.777292503 | 0.783570976 | 0.788046363 | 0.791189803 | 0.794210811 | 0.79810586 | 0.802505867 | 0.807404778 | 0.810420074 | 0.8122146 |
| Stockport | 0.751620973 | 0.757002826 | 0.763161137 | 0.769049941 | 0.773894448 | 0.777004091 | 0.779429439 | 0.782650547 | 0.786493222 | 0.791417208 | 0.796903838 | 0.801480978 | 0.804775277 | 0.807042857 | 0.809342833 | 0.81152974 | 0.813567198 | 0.81570169 | 0.817616656 | 0.819441774 | 0.82200484 | 0.825237167 | 0.830016178 | 0.835601339 | 0.839979958 | 0.843625218 | 0.847332112 | 0.851176959 | 0.854776689 | 0.858492733 | 0.860522249 | 0.861719713 |
| Tameside | 0.690169822 | 0.694965954 | 0.701117587 | 0.707610216 | 0.713230143 | 0.716889481 | 0.720061883 | 0.723720255 | 0.727376926 | 0.732192994 | 0.737502819 | 0.741927754 | 0.744986666 | 0.7468947 | 0.749097837 | 0.750656373 | 0.751434367 | 0.752362378 | 0.753117446 | 0.754404371 | 0.756896912 | 0.760465288 | 0.766338054 | 0.773030268 | 0.777914016 | 0.781579501 | 0.78478989 | 0.78811133 | 0.791369027 | 0.795733717 | 0.798749192 | 0.800546694 |
| Trafford | 0.781976839 | 0.78671279 | 0.792185092 | 0.797857675 | 0.802838184 | 0.806303374 | 0.80907728 | 0.812217293 | 0.815846012 | 0.82081479 | 0.826354235 | 0.83102853 | 0.834705715 | 0.837765499 | 0.841002981 | 0.843814355 | 0.846218967 | 0.848329943 | 0.849820753 | 0.85138938 | 0.854090014 | 0.857502815 | 0.862227852 | 0.867785323 | 0.872544364 | 0.876876091 | 0.881017394 | 0.885201626 | 0.889292461 | 0.893322381 | 0.895506133 | 0.896753779 |
| Warrington | 0.757425219 | 0.762524405 | 0.768492764 | 0.774199848 | 0.778864593 | 0.781605683 | 0.783694627 | 0.786230134 | 0.78928744 | 0.794297245 | 0.800427615 | 0.805528013 | 0.80924165 | 0.812293769 | 0.815764744 | 0.819001128 | 0.821219822 | 0.822990028 | 0.824672705 | 0.826804037 | 0.830447786 | 0.835116805 | 0.84105702 | 0.847393535 | 0.852495778 | 0.857010575 | 0.861506735 | 0.865948965 | 0.870099593 | 0.874597793 | 0.877257419 | 0.878756212 |
| Wigan | 0.69385144 | 0.698265316 | 0.703972367 | 0.710030694 | 0.7151698 | 0.718306821 | 0.720969646 | 0.724216723 | 0.727230537 | 0.731261727 | 0.736562437 | 0.741057771 | 0.743569203 | 0.744721411 | 0.746555181 | 0.749067247 | 0.751463927 | 0.753064367 | 0.754269267 | 0.756390539 | 0.760195877 | 0.765184807 | 0.771412509 | 0.777457071 | 0.781781458 | 0.785499107 | 0.789440132 | 0.793911138 | 0.798191322 | 0.802569612 | 0.805200084 | 0.80685214 |
| Wirral | 0.706506429 | 0.711299157 | 0.71684603 | 0.722583903 | 0.727889248 | 0.731471658 | 0.734278981 | 0.737502235 | 0.740843839 | 0.745220385 | 0.750337652 | 0.754471063 | 0.756932172 | 0.75855582 | 0.760945164 | 0.763330473 | 0.765487295 | 0.767513629 | 0.769416773 | 0.771316129 | 0.773929551 | 0.777438613 | 0.783056317 | 0.789561711 | 0.794585613 | 0.79886987 | 0.80294669 | 0.807228539 | 0.81120829 | 0.815329448 | 0.817782465 | 0.819316291 |
| South East England | 0.772790602 | 0.777244962 | 0.781939055 | 0.786728675 | 0.791151604 | 0.794546578 | 0.797511116 | 0.801016407 | 0.804897274 | 0.809591967 | 0.814535922 | 0.818668755 | 0.821734573 | 0.824171831 | 0.826765475 | 0.828992172 | 0.830941465 | 0.83305352 | 0.835171007 | 0.837193557 | 0.839606798 | 0.84249881 | 0.846531945 | 0.851079067 | 0.854774684 | 0.858124977 | 0.861580415 | 0.865248203 | 0.868772082 | 0.872533598 | 0.874728205 | 0.876066596 |
| Bracknell Forest | 0.78835599 | 0.792376918 | 0.796362939 | 0.800201995 | 0.80372606 | 0.806494029 | 0.809234574 | 0.812806613 | 0.817085183 | 0.822449514 | 0.828073571 | 0.832727962 | 0.836101605 | 0.838621882 | 0.841260799 | 0.843476341 | 0.845187298 | 0.846725893 | 0.848142347 | 0.849721467 | 0.8520067 | 0.854975184 | 0.85932558 | 0.864398153 | 0.868642802 | 0.872623175 | 0.876611094 | 0.880532558 | 0.884212898 | 0.887840109 | 0.889815809 | 0.891002655 |
| Brighton and Hove | 0.792979183 | 0.797690676 | 0.802289163 | 0.806994316 | 0.811592102 | 0.815718978 | 0.819632815 | 0.823822985 | 0.82813446 | 0.832806187 | 0.837756547 | 0.842402297 | 0.846430952 | 0.850073941 | 0.853689814 | 0.856952703 | 0.860056516 | 0.863181188 | 0.866289904 | 0.869007589 | 0.871724738 | 0.87446877 | 0.877508853 | 0.880608184 | 0.883209139 | 0.885565747 | 0.888042628 | 0.89074714 | 0.893377156 | 0.896051578 | 0.897327734 | 0.898257203 |
| Buckinghamshire | 0.795667014 | 0.79972445 | 0.804153468 | 0.808566331 | 0.812712818 | 0.816047897 | 0.819147253 | 0.822745824 | 0.826370169 | 0.830647683 | 0.835272797 | 0.839091761 | 0.841879461 | 0.844087857 | 0.846431258 | 0.848299346 | 0.84960667 | 0.850792624 | 0.851787805 | 0.852674157 | 0.854178824 | 0.856674909 | 0.860626791 | 0.865089926 | 0.868669591 | 0.871833664 | 0.875012421 | 0.878519564 | 0.882078195 | 0.885714583 | 0.887625477 | 0.888785243 |
| East Sussex | 0.729563972 | 0.734239331 | 0.739055297 | 0.743947209 | 0.748758441 | 0.752523436 | 0.755398663 | 0.758712415 | 0.762538853 | 0.767349635 | 0.772518394 | 0.776749683 | 0.779820552 | 0.782247364 | 0.784897923 | 0.78706102 | 0.78873477 | 0.790402138 | 0.791900365 | 0.793308109 | 0.795595611 | 0.79867364 | 0.803275626 | 0.808744537 | 0.813340362 | 0.817592228 | 0.821999928 | 0.826523161 | 0.830772959 | 0.835311336 | 0.838009845 | 0.839594794 |
| Hampshire | 0.770575146 | 0.775101568 | 0.779529051 | 0.78406633 | 0.788214791 | 0.791303029 | 0.794093757 | 0.797339118 | 0.800995234 | 0.805765068 | 0.810897327 | 0.815092934 | 0.818011068 | 0.820111233 | 0.822383816 | 0.824397821 | 0.826128201 | 0.828047093 | 0.829879764 | 0.831636082 | 0.833981752 | 0.837095049 | 0.841579574 | 0.846560597 | 0.850560542 | 0.854375329 | 0.858340565 | 0.862279368 | 0.865799775 | 0.869467151 | 0.87155772 | 0.87285 |
| Isle of Wight | 0.708723981 | 0.71354308 | 0.718447987 | 0.723447718 | 0.728367559 | 0.732199272 | 0.735867966 | 0.740313162 | 0.744732602 | 0.749753512 | 0.755179098 | 0.75993144 | 0.763227414 | 0.765197873 | 0.767190305 | 0.769048155 | 0.771243742 | 0.773851582 | 0.776296319 | 0.778205286 | 0.780188294 | 0.782772792 | 0.78683895 | 0.791784961 | 0.79608654 | 0.800417378 | 0.805199237 | 0.810675778 | 0.816035572 | 0.821304775 | 0.824516043 | 0.826399628 |
| Kent | 0.737842246 | 0.742361735 | 0.747373795 | 0.752527314 | 0.757224036 | 0.760513289 | 0.763050684 | 0.766333125 | 0.77029105 | 0.775213379 | 0.780312892 | 0.784435596 | 0.787449661 | 0.789716958 | 0.792148635 | 0.794133227 | 0.795956889 | 0.797982096 | 0.799958109 | 0.802007094 | 0.804780832 | 0.808198531 | 0.812863954 | 0.817983196 | 0.821948139 | 0.825302036 | 0.828736799 | 0.832666755 | 0.836630287 | 0.841021526 | 0.843724114 | 0.845345393 |
| Medway | 0.712321819 | 0.717281118 | 0.722438665 | 0.72800876 | 0.733302788 | 0.73683364 | 0.739660696 | 0.743128307 | 0.746490245 | 0.750536591 | 0.755108235 | 0.758772068 | 0.761624452 | 0.76420479 | 0.767136918 | 0.769424529 | 0.771189669 | 0.772975246 | 0.774689394 | 0.776622804 | 0.779421132 | 0.782734833 | 0.787410437 | 0.792556673 | 0.796368193 | 0.799588319 | 0.803053901 | 0.807348197 | 0.811672931 | 0.816185171 | 0.818939547 | 0.820667842 |
| Milton Keynes | 0.77322075 | 0.778331804 | 0.783797352 | 0.789245102 | 0.794136978 | 0.79737607 | 0.799996722 | 0.803147662 | 0.806334838 | 0.810432989 | 0.815088196 | 0.818976393 | 0.821873203 | 0.824210268 | 0.826891549 | 0.828972199 | 0.830338091 | 0.831421312 | 0.832265447 | 0.833394583 | 0.835474015 | 0.838811441 | 0.844445549 | 0.851413166 | 0.857573353 | 0.863206548 | 0.868750467 | 0.874152562 | 0.878326764 | 0.882215768 | 0.885303944 | 0.88717934 |
| Oxfordshire | 0.797879483 | 0.801991449 | 0.806535965 | 0.811259773 | 0.815680507 | 0.819112355 | 0.821896669 | 0.825133939 | 0.828850108 | 0.833407475 | 0.838355753 | 0.842716639 | 0.845995856 | 0.848592941 | 0.851190903 | 0.853538101 | 0.855660328 | 0.858044853 | 0.860541876 | 0.862723103 | 0.864982803 | 0.867568947 | 0.871095351 | 0.875123778 | 0.878637595 | 0.882188277 | 0.885915328 | 0.88953937 | 0.892791488 | 0.896327169 | 0.898318875 | 0.899475931 |
| Portsmouth | 0.759838477 | 0.764590616 | 0.769683769 | 0.775121398 | 0.780223929 | 0.784492061 | 0.788624308 | 0.793325128 | 0.797954383 | 0.80271548 | 0.807617795 | 0.811990624 | 0.815429111 | 0.818227591 | 0.82090657 | 0.823227719 | 0.825452088 | 0.828055077 | 0.83115236 | 0.834162466 | 0.836719758 | 0.839095588 | 0.842330728 | 0.846062164 | 0.849116772 | 0.851793636 | 0.854472501 | 0.857352698 | 0.860011857 | 0.862929609 | 0.864674721 | 0.865875927 |
| Reading | 0.808190771 | 0.812818 | 0.817601328 | 0.822252547 | 0.826413735 | 0.829859222 | 0.833249724 | 0.837373085 | 0.841883449 | 0.847268994 | 0.852937444 | 0.857842987 | 0.861816603 | 0.865080304 | 0.868230149 | 0.870574529 | 0.871852174 | 0.873668648 | 0.87611377 | 0.878229234 | 0.879047613 | 0.879578251 | 0.881401748 | 0.884560136 | 0.887659422 | 0.890492706 | 0.893300578 | 0.896216776 | 0.898970632 | 0.901901565 | 0.904319078 | 0.905873593 |
| Slough | 0.772614527 | 0.777852952 | 0.783348505 | 0.789105341 | 0.79477329 | 0.799107234 | 0.803009866 | 0.807531049 | 0.811901035 | 0.815957412 | 0.819002653 | 0.8214746 | 0.82330758 | 0.82451084 | 0.825758598 | 0.826216748 | 0.825983746 | 0.825858964 | 0.826156592 | 0.828044614 | 0.831354863 | 0.835839705 | 0.842132062 | 0.848741236 | 0.853853348 | 0.858037019 | 0.862026526 | 0.865941874 | 0.869476047 | 0.873282531 | 0.876537297 | 0.878557248 |
| Southampton | 0.759131491 | 0.764184185 | 0.769582897 | 0.775357415 | 0.780772518 | 0.785228459 | 0.789500375 | 0.794431093 | 0.799486349 | 0.804825417 | 0.810150229 | 0.814751289 | 0.818510828 | 0.821644155 | 0.82455951 | 0.826937006 | 0.829068171 | 0.831368611 | 0.833849752 | 0.835651537 | 0.836889524 | 0.838285073 | 0.840582564 | 0.843309487 | 0.845349027 | 0.847251511 | 0.849610473 | 0.852434281 | 0.855177649 | 0.85828484 | 0.860100186 | 0.861349084 |
| Surrey | 0.809241765 | 0.813218803 | 0.81740927 | 0.821597713 | 0.825347581 | 0.828574383 | 0.831625334 | 0.835056831 | 0.839083767 | 0.844177461 | 0.849501381 | 0.853985687 | 0.857289362 | 0.859941511 | 0.862466195 | 0.864453156 | 0.866073865 | 0.867884821 | 0.869703675 | 0.871325979 | 0.873267404 | 0.875482917 | 0.878778277 | 0.882605379 | 0.885886345 | 0.889046625 | 0.892281431 | 0.895537743 | 0.898748168 | 0.902084102 | 0.903829646 | 0.904859403 |
| West Berkshire | 0.805550058 | 0.810452711 | 0.815744156 | 0.820928839 | 0.825327085 | 0.828370424 | 0.830796334 | 0.833740327 | 0.837243331 | 0.841790163 | 0.846819868 | 0.85087726 | 0.853476018 | 0.85414895 | 0.854778966 | 0.854821244 | 0.854399691 | 0.854041077 | 0.854250282 | 0.855786905 | 0.85834418 | 0.861880374 | 0.866856452 | 0.872264683 | 0.87666497 | 0.88025107 | 0.88359419 | 0.887025363 | 0.890218579 | 0.893450611 | 0.896086136 | 0.897757011 |
| West Sussex | 0.762071928 | 0.766742633 | 0.771673315 | 0.776482782 | 0.780726888 | 0.783815722 | 0.786408682 | 0.789594608 | 0.793359433 | 0.798066328 | 0.80303899 | 0.807279054 | 0.810319563 | 0.812483034 | 0.814960956 | 0.817071906 | 0.818834655 | 0.820587246 | 0.82211726 | 0.823584136 | 0.825900887 | 0.829013534 | 0.833474469 | 0.838651426 | 0.842910214 | 0.846594332 | 0.850143095 | 0.853809119 | 0.857333807 | 0.86114164 | 0.863390071 | 0.864792147 |
| Windsor and Maidenhead | 0.818140871 | 0.82161379 | 0.825335136 | 0.829300668 | 0.833100456 | 0.83615734 | 0.838814994 | 0.842056621 | 0.845764989 | 0.850320792 | 0.855261344 | 0.859475538 | 0.862981586 | 0.866000205 | 0.868865983 | 0.871171982 | 0.873010755 | 0.874888711 | 0.876646829 | 0.878021485 | 0.879898647 | 0.882329264 | 0.886039945 | 0.890857911 | 0.89511708 | 0.898810609 | 0.902350869 | 0.905902704 | 0.909291748 | 0.912848892 | 0.914738041 | 0.91581805 |
| Wokingham | 0.82484241 | 0.829020445 | 0.83302523 | 0.836971846 | 0.840701088 | 0.843846097 | 0.846792561 | 0.850285993 | 0.85408238 | 0.858443204 | 0.863062582 | 0.867108607 | 0.870407601 | 0.873114573 | 0.875894292 | 0.878347065 | 0.880284856 | 0.882139737 | 0.884006565 | 0.885252182 | 0.886109883 | 0.887270311 | 0.889972209 | 0.893653298 | 0.896659728 | 0.89922274 | 0.901731391 | 0.904247613 | 0.906513571 | 0.909188749 | 0.910727479 | 0.911658755 |
| South West England | 0.747943254 | 0.752830357 | 0.757839338 | 0.762930652 | 0.767653174 | 0.77118217 | 0.77424567 | 0.778030724 | 0.782430638 | 0.787870311 | 0.793436971 | 0.797868344 | 0.801046338 | 0.803681793 | 0.806525756 | 0.809004253 | 0.8111514 | 0.81334646 | 0.815632009 | 0.817788616 | 0.820449088 | 0.823678966 | 0.828209506 | 0.833313039 | 0.837517074 | 0.841351033 | 0.845232202 | 0.849369624 | 0.85337881 | 0.857594353 | 0.860009885 | 0.861467015 |
| Bath and North East Somerset | 0.787410378 | 0.791679434 | 0.796062083 | 0.800686236 | 0.80515302 | 0.808856243 | 0.812099586 | 0.815971076 | 0.820464266 | 0.825483228 | 0.830750853 | 0.835845017 | 0.840505747 | 0.844822463 | 0.848730498 | 0.85221763 | 0.855689014 | 0.859385246 | 0.862965554 | 0.865552028 | 0.867777094 | 0.870018231 | 0.872744296 | 0.875752044 | 0.878281448 | 0.880904492 | 0.88409246 | 0.887455633 | 0.890314568 | 0.893069398 | 0.894450981 | 0.895435359 |
| Bournemouth | 0.755273408 | 0.760904777 | 0.76639109 | 0.771589882 | 0.776447775 | 0.780418675 | 0.78395412 | 0.788175663 | 0.793129783 | 0.798834399 | 0.804807497 | 0.810110083 | 0.814539803 | 0.818524192 | 0.822418447 | 0.825604473 | 0.828413212 | 0.831354696 | 0.834344269 | 0.836829312 | 0.838989663 | 0.841236486 | 0.844591334 | 0.848619677 | 0.852113046 | 0.855196656 | 0.858227263 | 0.861432654 | 0.864478081 | 0.867611695 | 0.869319211 | 0.870464766 |
| Bristol, City of | 0.784610852 | 0.789563536 | 0.794631999 | 0.799891908 | 0.804923182 | 0.809005773 | 0.812692564 | 0.817008436 | 0.8216647 | 0.826973496 | 0.832226334 | 0.836580941 | 0.839828402 | 0.842421762 | 0.845139321 | 0.847860098 | 0.850672104 | 0.853930639 | 0.857673759 | 0.860850724 | 0.863395265 | 0.865810896 | 0.869120932 | 0.873065734 | 0.876725185 | 0.880247248 | 0.883751206 | 0.887387496 | 0.890720447 | 0.894066989 | 0.895872352 | 0.897041874 |
| Cornwall | 0.723353527 | 0.728807695 | 0.734489376 | 0.740317544 | 0.745287422 | 0.74856818 | 0.751176734 | 0.754545903 | 0.758684459 | 0.764192088 | 0.769946585 | 0.77429251 | 0.777613544 | 0.780452616 | 0.783394975 | 0.785738389 | 0.787802963 | 0.78975369 | 0.791484651 | 0.793226048 | 0.795477202 | 0.798435082 | 0.803325769 | 0.809026625 | 0.813704391 | 0.817756448 | 0.821920462 | 0.826381015 | 0.830734457 | 0.835393966 | 0.838174298 | 0.839768559 |
| Devon | 0.744138186 | 0.749037326 | 0.753944293 | 0.758875878 | 0.763286888 | 0.766307015 | 0.768956137 | 0.772504244 | 0.776764326 | 0.782179207 | 0.787746699 | 0.792365567 | 0.795891582 | 0.798924806 | 0.802244086 | 0.805419115 | 0.808202325 | 0.810775677 | 0.81318568 | 0.815304647 | 0.817806666 | 0.820897905 | 0.825396213 | 0.830435184 | 0.834424792 | 0.837781348 | 0.840893713 | 0.844266449 | 0.847718202 | 0.851620091 | 0.853964987 | 0.855376103 |
| Dorset | 0.740994535 | 0.745585307 | 0.750206833 | 0.754874337 | 0.759291606 | 0.762327758 | 0.765060227 | 0.768865932 | 0.773326451 | 0.778577111 | 0.783533597 | 0.787348356 | 0.790163476 | 0.792355649 | 0.79467044 | 0.79649652 | 0.797790122 | 0.798827814 | 0.799844051 | 0.801417665 | 0.804496802 | 0.808422996 | 0.813572422 | 0.819216792 | 0.823879571 | 0.828160771 | 0.832619806 | 0.837485234 | 0.842285505 | 0.847110441 | 0.849937041 | 0.851615144 |
| Gloucestershire | 0.76125096 | 0.766177348 | 0.771016768 | 0.775795147 | 0.780262872 | 0.783751041 | 0.786736732 | 0.790009743 | 0.793687427 | 0.798643084 | 0.80408717 | 0.808627669 | 0.811966666 | 0.815068798 | 0.818415689 | 0.820946515 | 0.82260538 | 0.824146797 | 0.825820938 | 0.827594568 | 0.830560377 | 0.834379062 | 0.839154557 | 0.844291173 | 0.848593621 | 0.852615597 | 0.856484454 | 0.860413976 | 0.864171481 | 0.868101692 | 0.870378306 | 0.871806302 |
| North Somerset | 0.74308392 | 0.748176981 | 0.753257523 | 0.758151374 | 0.762368074 | 0.76532543 | 0.767841052 | 0.770787331 | 0.774183006 | 0.778905521 | 0.783953429 | 0.788123371 | 0.791371298 | 0.794079225 | 0.796909821 | 0.799354421 | 0.801202402 | 0.803309757 | 0.805743877 | 0.808238652 | 0.811526506 | 0.815625055 | 0.821090589 | 0.826968565 | 0.831723961 | 0.836036401 | 0.840567339 | 0.845527164 | 0.850137809 | 0.854732853 | 0.857425593 | 0.859017669 |
| Plymouth | 0.73401315 | 0.73892965 | 0.744467059 | 0.750099797 | 0.755131717 | 0.758732071 | 0.76162238 | 0.765366246 | 0.769783922 | 0.775008813 | 0.780270166 | 0.784321431 | 0.787204459 | 0.789572622 | 0.792281704 | 0.794566384 | 0.796948073 | 0.800283567 | 0.804417176 | 0.80773762 | 0.810001553 | 0.8120936 | 0.815749678 | 0.820199608 | 0.823560517 | 0.826420497 | 0.829300363 | 0.832416108 | 0.835704506 | 0.839543923 | 0.84182482 | 0.843307541 |
| Poole | 0.753452228 | 0.75793914 | 0.762633126 | 0.767637869 | 0.772653461 | 0.776587663 | 0.779892358 | 0.783655076 | 0.787872761 | 0.793233602 | 0.79869131 | 0.802695173 | 0.805212564 | 0.807210522 | 0.80983995 | 0.812313077 | 0.814015564 | 0.815509833 | 0.816786645 | 0.818019962 | 0.820496305 | 0.824366827 | 0.829958136 | 0.835996465 | 0.840807903 | 0.844820001 | 0.848653683 | 0.852480216 | 0.855986622 | 0.859738998 | 0.86203606 | 0.863494302 |
| Somerset | 0.729813379 | 0.734663347 | 0.739871994 | 0.745266885 | 0.750237632 | 0.753664979 | 0.756291819 | 0.759357155 | 0.763015065 | 0.767910485 | 0.773030535 | 0.776980191 | 0.779409743 | 0.781458554 | 0.784004782 | 0.786259552 | 0.788072833 | 0.789491229 | 0.790525349 | 0.791907079 | 0.794720836 | 0.79856494 | 0.803822901 | 0.80964748 | 0.814347249 | 0.818787241 | 0.823408847 | 0.82842183 | 0.833355226 | 0.838465114 | 0.841563519 | 0.843404311 |
| South Gloucestershire | 0.77431501 | 0.778082843 | 0.782047764 | 0.786109976 | 0.789994211 | 0.793285599 | 0.796854367 | 0.801304921 | 0.805966637 | 0.811302141 | 0.816875631 | 0.821378582 | 0.824498108 | 0.827223744 | 0.830252572 | 0.833260022 | 0.836038404 | 0.838666265 | 0.841333469 | 0.843596734 | 0.845849249 | 0.84861734 | 0.853155506 | 0.85859109 | 0.863021034 | 0.866985675 | 0.870666364 | 0.87417854 | 0.877532672 | 0.881307358 | 0.883603797 | 0.884984764 |
| Swindon | 0.763337469 | 0.768908604 | 0.774454839 | 0.779791493 | 0.784518226 | 0.787462164 | 0.78949609 | 0.792243574 | 0.795621411 | 0.8002938 | 0.805265289 | 0.809135874 | 0.81160586 | 0.813267347 | 0.814877514 | 0.815930853 | 0.816499473 | 0.817313657 | 0.818551293 | 0.82002005 | 0.822941591 | 0.827164702 | 0.832998398 | 0.839087817 | 0.84340154 | 0.846891126 | 0.850662363 | 0.854967259 | 0.85914119 | 0.863470405 | 0.866058344 | 0.867545906 |
| Torbay | 0.708212902 | 0.712970993 | 0.718010472 | 0.723061826 | 0.727404081 | 0.730273039 | 0.732910662 | 0.736375272 | 0.74010756 | 0.745153112 | 0.750308514 | 0.753381461 | 0.754388112 | 0.754903002 | 0.756464687 | 0.75815326 | 0.759624249 | 0.76116012 | 0.763021878 | 0.764741518 | 0.766408812 | 0.768474084 | 0.772549303 | 0.777851635 | 0.782366044 | 0.786669013 | 0.791305292 | 0.796524157 | 0.801813216 | 0.807399566 | 0.810951699 | 0.813038477 |
| Wiltshire | 0.751135505 | 0.755451157 | 0.759884685 | 0.764427612 | 0.768893382 | 0.77224644 | 0.774881797 | 0.778147472 | 0.782047535 | 0.787235972 | 0.79261431 | 0.796721433 | 0.799589586 | 0.801972665 | 0.804750599 | 0.806772535 | 0.807979797 | 0.808488816 | 0.80853481 | 0.809256053 | 0.811888722 | 0.815869405 | 0.821393499 | 0.827251456 | 0.831994693 | 0.836522546 | 0.841232024 | 0.84609662 | 0.850598018 | 0.855158959 | 0.85786894 | 0.859466242 |
| West Midlands | 0.712229751 | 0.717294817 | 0.722991462 | 0.728771674 | 0.73384891 | 0.737082996 | 0.73977625 | 0.743457914 | 0.747776577 | 0.753376821 | 0.759401986 | 0.764194931 | 0.767392117 | 0.769902529 | 0.772965911 | 0.776022184 | 0.778765345 | 0.781588429 | 0.784605518 | 0.787625943 | 0.790933208 | 0.794735154 | 0.800225851 | 0.806279667 | 0.81090038 | 0.814669794 | 0.818336808 | 0.822459817 | 0.826556355 | 0.831192891 | 0.834122604 | 0.835865141 |
| Birmingham | 0.711918055 | 0.716941572 | 0.722790385 | 0.728461133 | 0.733522363 | 0.736635931 | 0.739095684 | 0.742751257 | 0.74712647 | 0.752647744 | 0.758474324 | 0.763184147 | 0.766441105 | 0.769276164 | 0.772988033 | 0.776629303 | 0.779852388 | 0.783399128 | 0.787615964 | 0.791917284 | 0.795687818 | 0.799495087 | 0.805044618 | 0.810990262 | 0.815333169 | 0.818569572 | 0.821693851 | 0.825470981 | 0.829283015 | 0.833601709 | 0.836372702 | 0.838066899 |
| Coventry | 0.730835783 | 0.73669449 | 0.742633422 | 0.748709332 | 0.754362863 | 0.758228958 | 0.761683748 | 0.765997002 | 0.770440984 | 0.775696268 | 0.781340721 | 0.785852876 | 0.789000304 | 0.791463548 | 0.794418626 | 0.796852475 | 0.798630544 | 0.800716967 | 0.803626023 | 0.806894084 | 0.810128588 | 0.813655926 | 0.818328388 | 0.8231046 | 0.826674259 | 0.829857964 | 0.833254704 | 0.836828618 | 0.840277578 | 0.844305733 | 0.846953998 | 0.848618069 |
| Dudley | 0.698687632 | 0.702693024 | 0.707436032 | 0.712484459 | 0.716784504 | 0.719634967 | 0.722330173 | 0.725895686 | 0.72971007 | 0.734414914 | 0.739793627 | 0.744278417 | 0.747291284 | 0.749367708 | 0.751521968 | 0.753961386 | 0.756202693 | 0.758001399 | 0.759196456 | 0.76011798 | 0.761604447 | 0.763846673 | 0.768809796 | 0.775120615 | 0.779790857 | 0.783167934 | 0.786375036 | 0.790388126 | 0.79458623 | 0.799240521 | 0.802291327 | 0.804164522 |
| Herefordshire, County of | 0.722860527 | 0.728012127 | 0.733900249 | 0.739954651 | 0.745296614 | 0.748918535 | 0.751922712 | 0.755598086 | 0.75991016 | 0.765623471 | 0.771593577 | 0.776260112 | 0.779357541 | 0.781822347 | 0.784566239 | 0.787030241 | 0.789024654 | 0.790937821 | 0.792409002 | 0.793606175 | 0.795854116 | 0.799053945 | 0.804075001 | 0.810241185 | 0.815700859 | 0.820697877 | 0.825703135 | 0.831061655 | 0.836179742 | 0.841373245 | 0.844517114 | 0.846310474 |
| Sandwell | 0.674777822 | 0.678886945 | 0.684257203 | 0.690250227 | 0.69529774 | 0.698067871 | 0.700465198 | 0.703999554 | 0.707805314 | 0.712879546 | 0.718579451 | 0.722832161 | 0.725456062 | 0.727563042 | 0.730418188 | 0.733333103 | 0.735381719 | 0.736428916 | 0.737293459 | 0.738987154 | 0.741843398 | 0.746092794 | 0.753308922 | 0.761506262 | 0.76752487 | 0.77225644 | 0.776349578 | 0.780608145 | 0.784856711 | 0.789951722 | 0.793547271 | 0.795722297 |
| Shropshire | 0.72909223 | 0.733978943 | 0.739324288 | 0.7444821 | 0.749057252 | 0.751921113 | 0.754203631 | 0.757586414 | 0.761759383 | 0.767237234 | 0.773018221 | 0.777462842 | 0.780665832 | 0.783912621 | 0.787578851 | 0.790784338 | 0.793288862 | 0.795228136 | 0.796635562 | 0.798336178 | 0.801410392 | 0.805207767 | 0.810272262 | 0.815760258 | 0.819691291 | 0.822977941 | 0.826572567 | 0.830653046 | 0.834681072 | 0.83906667 | 0.841686065 | 0.843218762 |
| Solihull | 0.75697799 | 0.762336153 | 0.768332551 | 0.773961556 | 0.778524879 | 0.781575749 | 0.784494296 | 0.788254026 | 0.792430764 | 0.797823903 | 0.803605572 | 0.808278922 | 0.811374965 | 0.813800016 | 0.816467005 | 0.818826206 | 0.821062822 | 0.823433076 | 0.825588089 | 0.827159038 | 0.828629079 | 0.830999217 | 0.835342227 | 0.840597768 | 0.845159862 | 0.849339045 | 0.853280591 | 0.857609222 | 0.862207818 | 0.867196335 | 0.870260723 | 0.872079235 |
| Staffordshire | 0.723512602 | 0.728904975 | 0.734918802 | 0.740985181 | 0.74579796 | 0.748529298 | 0.750866348 | 0.754092905 | 0.75817765 | 0.763618739 | 0.769074373 | 0.773295701 | 0.775829753 | 0.777541421 | 0.779900739 | 0.782473421 | 0.784658583 | 0.78686557 | 0.789367177 | 0.791667665 | 0.794200202 | 0.797156213 | 0.801454848 | 0.806136424 | 0.80949591 | 0.812158109 | 0.814747387 | 0.817858166 | 0.821129742 | 0.825175255 | 0.827769573 | 0.829320238 |
| Stoke-on-Trent | 0.682717867 | 0.688223883 | 0.6937868 | 0.699181327 | 0.704248652 | 0.707532771 | 0.710120289 | 0.713765953 | 0.717625826 | 0.722274839 | 0.727149756 | 0.730272244 | 0.731569975 | 0.731857774 | 0.732529049 | 0.733420289 | 0.734041565 | 0.734899914 | 0.737228396 | 0.74076156 | 0.744676523 | 0.748926302 | 0.75520015 | 0.762563778 | 0.768503545 | 0.773377806 | 0.777866492 | 0.782393123 | 0.786806823 | 0.79227991 | 0.796083385 | 0.798242518 |
| Telford and Wrekin | 0.716348041 | 0.721031078 | 0.726515931 | 0.732272613 | 0.737756079 | 0.741501812 | 0.744657839 | 0.748356888 | 0.752034519 | 0.757024051 | 0.76241506 | 0.766026485 | 0.767944599 | 0.768625174 | 0.769621259 | 0.770690612 | 0.771584473 | 0.772718107 | 0.773656016 | 0.774521611 | 0.776453077 | 0.779904058 | 0.786219311 | 0.793378906 | 0.798714526 | 0.802632214 | 0.806386526 | 0.810912259 | 0.81586187 | 0.82163784 | 0.825355976 | 0.827341561 |
| Walsall | 0.67868011 | 0.681778926 | 0.686454909 | 0.692208265 | 0.696833385 | 0.698847612 | 0.699530532 | 0.70106263 | 0.704017822 | 0.708980276 | 0.714607176 | 0.718867494 | 0.721266268 | 0.723058637 | 0.725481795 | 0.727927054 | 0.730592779 | 0.73319739 | 0.735083915 | 0.737555208 | 0.741381517 | 0.745818998 | 0.751947961 | 0.758620152 | 0.763666232 | 0.767774886 | 0.771618493 | 0.776018231 | 0.780619588 | 0.786272093 | 0.790212752 | 0.792529439 |
| Warwickshire | 0.753085339 | 0.758705831 | 0.764235811 | 0.769224656 | 0.773814492 | 0.777157593 | 0.78000855 | 0.783515693 | 0.787519487 | 0.792720704 | 0.798307509 | 0.802705911 | 0.805673688 | 0.807703357 | 0.810017159 | 0.812270517 | 0.814557195 | 0.817143078 | 0.819860344 | 0.822565395 | 0.825789364 | 0.829590471 | 0.834440759 | 0.839707435 | 0.843788548 | 0.847231009 | 0.850977317 | 0.854931018 | 0.858547072 | 0.862573675 | 0.865129865 | 0.866672661 |
| Wolverhampton | 0.692133664 | 0.696834259 | 0.702632522 | 0.709165938 | 0.715134949 | 0.718299619 | 0.720305573 | 0.7235514 | 0.727692618 | 0.73356159 | 0.739908622 | 0.744865582 | 0.747704087 | 0.74969265 | 0.752372351 | 0.754941499 | 0.757178976 | 0.759717518 | 0.762225351 | 0.764185098 | 0.76655327 | 0.769970314 | 0.775229815 | 0.780941242 | 0.785448141 | 0.78948146 | 0.793602406 | 0.798315867 | 0.802766114 | 0.807488073 | 0.81054331 | 0.812389843 |
| Worcestershire | 0.731756994 | 0.736468614 | 0.741498891 | 0.746667055 | 0.751165511 | 0.754157486 | 0.756841299 | 0.76053245 | 0.76511163 | 0.771225385 | 0.777658804 | 0.782601726 | 0.785656516 | 0.787881831 | 0.79043604 | 0.793067151 | 0.795464655 | 0.797614858 | 0.799252409 | 0.800335848 | 0.802171746 | 0.804940638 | 0.809378941 | 0.81463709 | 0.818905026 | 0.822682238 | 0.826431276 | 0.830706067 | 0.834897769 | 0.839321589 | 0.842016969 | 0.843611304 |
| Yorkshire and the Humber | 0.71744891 | 0.721942348 | 0.727224023 | 0.732715226 | 0.7376591 | 0.741090229 | 0.744213656 | 0.748039192 | 0.752248852 | 0.757564663 | 0.763224901 | 0.767823157 | 0.77092397 | 0.773168847 | 0.775854923 | 0.778524477 | 0.781082793 | 0.783776479 | 0.786470004 | 0.789423343 | 0.793000001 | 0.797154483 | 0.802732454 | 0.808730122 | 0.813009261 | 0.816418476 | 0.819998903 | 0.824127659 | 0.828232043 | 0.832925305 | 0.835780283 | 0.837459877 |
| Barnsley | 0.674409277 | 0.677297607 | 0.682143092 | 0.687701817 | 0.692117147 | 0.694451745 | 0.697069072 | 0.701108238 | 0.70552609 | 0.710642371 | 0.715802147 | 0.719392947 | 0.721277901 | 0.722545485 | 0.724471913 | 0.726149094 | 0.727841215 | 0.729964017 | 0.731431562 | 0.733223499 | 0.73632621 | 0.740854922 | 0.747685128 | 0.755033873 | 0.75979321 | 0.763311183 | 0.767384248 | 0.772287683 | 0.777243814 | 0.783153097 | 0.787047392 | 0.789261821 |
| Bradford | 0.695777168 | 0.699723241 | 0.704427991 | 0.709368942 | 0.714104834 | 0.717070903 | 0.719157139 | 0.721203086 | 0.723017633 | 0.726441976 | 0.730742015 | 0.734492367 | 0.737350893 | 0.739688766 | 0.742459724 | 0.745267457 | 0.748046153 | 0.750427091 | 0.752144987 | 0.754842206 | 0.759353682 | 0.765149899 | 0.773072225 | 0.781460741 | 0.787448016 | 0.791929518 | 0.79610027 | 0.800784366 | 0.805493076 | 0.81077249 | 0.814086601 | 0.815985897 |
| Calderdale | 0.717583821 | 0.72220176 | 0.727794375 | 0.733498025 | 0.738242957 | 0.740888394 | 0.743001819 | 0.746067255 | 0.749534048 | 0.754305043 | 0.75936439 | 0.763070625 | 0.765036263 | 0.766114687 | 0.768140651 | 0.770655122 | 0.772995677 | 0.774932262 | 0.776320458 | 0.778266253 | 0.781813114 | 0.787002593 | 0.794603956 | 0.802852503 | 0.80879226 | 0.813179835 | 0.817416247 | 0.82242643 | 0.82754697 | 0.832906122 | 0.836044305 | 0.837863928 |
| Doncaster | 0.67510562 | 0.678434292 | 0.682996246 | 0.688022272 | 0.692463822 | 0.694956323 | 0.696786009 | 0.699423852 | 0.703136631 | 0.708291833 | 0.713663492 | 0.717964645 | 0.720747418 | 0.722319813 | 0.724690893 | 0.727304607 | 0.729961966 | 0.732014801 | 0.732969178 | 0.735089988 | 0.740324101 | 0.747230348 | 0.755466364 | 0.763439304 | 0.768508899 | 0.772354454 | 0.77655094 | 0.781038028 | 0.785082063 | 0.790051639 | 0.793497396 | 0.795614015 |
| East Riding of Yorkshire | 0.729479586 | 0.734058197 | 0.739801277 | 0.745398719 | 0.750075423 | 0.753204147 | 0.755850404 | 0.759027005 | 0.76253921 | 0.767140291 | 0.771907918 | 0.775419839 | 0.777841424 | 0.779836645 | 0.782542813 | 0.784942959 | 0.786776588 | 0.788874755 | 0.7906523 | 0.792518557 | 0.795255191 | 0.798278865 | 0.802607998 | 0.807537004 | 0.811202612 | 0.814476872 | 0.818187083 | 0.822624118 | 0.826996166 | 0.831595396 | 0.834209325 | 0.835731941 |
| Kingston upon Hull, City of | 0.681942142 | 0.686303885 | 0.691360749 | 0.696855984 | 0.702058029 | 0.705435171 | 0.708841245 | 0.713685639 | 0.719310797 | 0.725860694 | 0.732359854 | 0.737468456 | 0.740566236 | 0.742361797 | 0.744630497 | 0.747081585 | 0.750043007 | 0.753881436 | 0.758091921 | 0.761921858 | 0.76485484 | 0.767912873 | 0.77243865 | 0.777488527 | 0.780283357 | 0.781802257 | 0.783604426 | 0.78627627 | 0.789437808 | 0.794307674 | 0.797839402 | 0.79993173 |
| Kirklees | 0.710984285 | 0.715952904 | 0.721728673 | 0.727298233 | 0.731498351 | 0.733768462 | 0.73592606 | 0.739005644 | 0.742545969 | 0.747270177 | 0.751884101 | 0.755279893 | 0.757517348 | 0.759456095 | 0.76230224 | 0.764949204 | 0.767067999 | 0.769230338 | 0.77148322 | 0.774020836 | 0.777078791 | 0.780978172 | 0.786783234 | 0.793343739 | 0.798045132 | 0.801751802 | 0.805703689 | 0.810140662 | 0.814546942 | 0.819503047 | 0.822518326 | 0.824207653 |
| Leeds | 0.753340826 | 0.758704729 | 0.764464312 | 0.770128491 | 0.775218036 | 0.779274871 | 0.783217875 | 0.787712447 | 0.792454193 | 0.79795548 | 0.803601369 | 0.808531446 | 0.812560295 | 0.816054502 | 0.819626742 | 0.823118881 | 0.826643646 | 0.830338426 | 0.834168555 | 0.837448075 | 0.839889242 | 0.842117622 | 0.845230848 | 0.848774316 | 0.851341048 | 0.853428794 | 0.855662117 | 0.858365329 | 0.861299051 | 0.865026612 | 0.86733998 | 0.868761251 |
| North East Lincolnshire | 0.679517747 | 0.685012163 | 0.691269153 | 0.697182622 | 0.702389449 | 0.705504761 | 0.707733487 | 0.710493044 | 0.713452048 | 0.717885014 | 0.723124017 | 0.726729804 | 0.727908289 | 0.727692862 | 0.728566093 | 0.73075318 | 0.733665413 | 0.736791642 | 0.739162885 | 0.741883838 | 0.745879374 | 0.750859695 | 0.75815562 | 0.76636165 | 0.772235512 | 0.776952378 | 0.781762906 | 0.786975085 | 0.792016024 | 0.798171397 | 0.802421054 | 0.804837847 |
| North Lincolnshire | 0.721431975 | 0.724986018 | 0.729588766 | 0.734674199 | 0.739194201 | 0.74239997 | 0.745328254 | 0.748478341 | 0.7514857 | 0.754696264 | 0.758263706 | 0.761554866 | 0.763693398 | 0.764742853 | 0.765819215 | 0.76708404 | 0.768745014 | 0.770399745 | 0.771325869 | 0.773989407 | 0.779320176 | 0.785397813 | 0.792600047 | 0.799678654 | 0.803778132 | 0.806444548 | 0.809532871 | 0.813240995 | 0.816941198 | 0.821413628 | 0.824243094 | 0.825904867 |
| North Yorkshire | 0.746089107 | 0.750306404 | 0.755072311 | 0.759882813 | 0.763926127 | 0.766510062 | 0.768917453 | 0.772331385 | 0.77626258 | 0.781084123 | 0.786357293 | 0.790607748 | 0.793548542 | 0.795859243 | 0.798762903 | 0.801416091 | 0.803575941 | 0.805072327 | 0.806213527 | 0.808078822 | 0.811183811 | 0.815392441 | 0.821264361 | 0.827174793 | 0.831049513 | 0.834197534 | 0.83777266 | 0.842403588 | 0.847169497 | 0.851904971 | 0.854617695 | 0.85619466 |
| Rotherham | 0.684048953 | 0.688005253 | 0.693073788 | 0.698683561 | 0.703887797 | 0.706812985 | 0.709061667 | 0.712089621 | 0.715385087 | 0.719984009 | 0.725280193 | 0.729431138 | 0.732088454 | 0.734154689 | 0.736970945 | 0.73948253 | 0.741225306 | 0.742984487 | 0.745036683 | 0.747597253 | 0.751182632 | 0.755212601 | 0.760917169 | 0.768096327 | 0.773427315 | 0.777703241 | 0.782427688 | 0.787829235 | 0.793019186 | 0.798621883 | 0.802159568 | 0.804228811 |
| Sheffield | 0.740870442 | 0.744939332 | 0.749592409 | 0.754683289 | 0.759741945 | 0.763734483 | 0.767149838 | 0.770746987 | 0.774813454 | 0.779963093 | 0.785631604 | 0.790716232 | 0.794503239 | 0.797512257 | 0.801113396 | 0.804709912 | 0.807941721 | 0.811603019 | 0.815804246 | 0.819595632 | 0.822836482 | 0.826136269 | 0.830254159 | 0.83426294 | 0.837077866 | 0.839457807 | 0.842176699 | 0.845270228 | 0.848192577 | 0.851587604 | 0.853609975 | 0.854890971 |
| Wakefield | 0.692436467 | 0.69653247 | 0.701823136 | 0.707800514 | 0.713152829 | 0.716363594 | 0.719061388 | 0.722368367 | 0.725655502 | 0.730279377 | 0.735798563 | 0.739886213 | 0.741830107 | 0.742916791 | 0.744988366 | 0.747007185 | 0.748426089 | 0.749436526 | 0.750027971 | 0.751652934 | 0.75497844 | 0.759731131 | 0.767052498 | 0.775248822 | 0.780873551 | 0.784680304 | 0.78832109 | 0.792547543 | 0.796583195 | 0.801441213 | 0.804744713 | 0.806811491 |
| York | 0.787154023 | 0.792373666 | 0.797581364 | 0.802250845 | 0.806402138 | 0.809928483 | 0.813354821 | 0.817286745 | 0.821463734 | 0.826269566 | 0.831259391 | 0.835850847 | 0.839703692 | 0.842932373 | 0.846272963 | 0.849473273 | 0.852529937 | 0.855591023 | 0.858598213 | 0.861261994 | 0.863791142 | 0.866373361 | 0.869462602 | 0.872594995 | 0.874956754 | 0.877158553 | 0.879473931 | 0.881852205 | 0.884101627 | 0.8865909 | 0.887811277 | 0.888738932 |
| Northern Ireland | 0.722723259 | 0.728469763 | 0.735161603 | 0.742021784 | 0.74762993 | 0.751320924 | 0.754622495 | 0.758643468 | 0.762872168 | 0.768131943 | 0.773847991 | 0.778839521 | 0.783504451 | 0.787673884 | 0.791116714 | 0.793592544 | 0.795962343 | 0.798347427 | 0.801119369 | 0.803823924 | 0.806267932 | 0.809266741 | 0.813113769 | 0.816790165 | 0.820062482 | 0.823786217 | 0.827775763 | 0.830969322 | 0.832806988 | 0.835250701 | 0.837049319 | 0.838531942 |
| Scotland | 0.743972492 | 0.748220021 | 0.753417051 | 0.759543792 | 0.764809282 | 0.76863789 | 0.771395327 | 0.774650905 | 0.779539941 | 0.785256752 | 0.790404802 | 0.794830017 | 0.7984167 | 0.801177674 | 0.803716855 | 0.805954633 | 0.80752952 | 0.80918775 | 0.812059828 | 0.815659149 | 0.819369347 | 0.822449969 | 0.824857595 | 0.827589677 | 0.830944529 | 0.834109986 | 0.837431761 | 0.841140643 | 0.84476207 | 0.848638591 | 0.850787145 | 0.852061416 |
| Wales | 0.710140734 | 0.715478036 | 0.721407686 | 0.727533596 | 0.733105415 | 0.73695851 | 0.74048567 | 0.744775328 | 0.7493989 | 0.755031994 | 0.760996809 | 0.765843408 | 0.76943011 | 0.772096119 | 0.775060178 | 0.777793105 | 0.780192749 | 0.782691394 | 0.785148785 | 0.787624313 | 0.790656664 | 0.794194949 | 0.799118219 | 0.804921906 | 0.809416942 | 0.813151383 | 0.816818185 | 0.820508402 | 0.824617063 | 0.8297892 | 0.832714295 | 0.834278536 |
| Latin America and Caribbean | 0.497738092 | 0.501730505 | 0.505674834 | 0.509875876 | 0.51433915 | 0.518546498 | 0.522877323 | 0.527604 | 0.532559673 | 0.537509233 | 0.542722372 | 0.547888539 | 0.552972476 | 0.557815915 | 0.562849229 | 0.567902619 | 0.573056019 | 0.57835546 | 0.583760176 | 0.588474322 | 0.593556643 | 0.599037703 | 0.604550909 | 0.610163381 | 0.615542877 | 0.620470129 | 0.625105547 | 0.629806568 | 0.634537971 | 0.639173108 | 0.64298253 | 0.646541285 |
| Andean Latin America | 0.50001149 | 0.501652202 | 0.50365586 | 0.506388431 | 0.510305574 | 0.514896344 | 0.519376447 | 0.52403649 | 0.528443909 | 0.532743717 | 0.537560512 | 0.54257235 | 0.547911071 | 0.553108984 | 0.558235444 | 0.563091517 | 0.567987399 | 0.572998972 | 0.578564192 | 0.583998942 | 0.59020118 | 0.59694462 | 0.603839225 | 0.610705169 | 0.617059985 | 0.622760754 | 0.628161263 | 0.633537897 | 0.638860164 | 0.643921325 | 0.647806819 | 0.651602456 |
| Bolivia | 0.423917961 | 0.429397795 | 0.434835542 | 0.440601646 | 0.4468225 | 0.453479243 | 0.460220669 | 0.466982897 | 0.473796201 | 0.48019431 | 0.486327903 | 0.492030764 | 0.497348837 | 0.502365803 | 0.507273036 | 0.512166491 | 0.517146194 | 0.52194613 | 0.52694375 | 0.53179244 | 0.537009068 | 0.542680745 | 0.548615482 | 0.554788216 | 0.560934581 | 0.567000898 | 0.573080316 | 0.579241285 | 0.585184106 | 0.590692043 | 0.594854443 | 0.599010799 |
| Ecuador | 0.518430614 | 0.517679421 | 0.51837907 | 0.520707291 | 0.524908291 | 0.529380752 | 0.532691399 | 0.535349652 | 0.53719633 | 0.539032308 | 0.543044779 | 0.54855215 | 0.554897325 | 0.560955485 | 0.566290293 | 0.569927345 | 0.572516273 | 0.574704702 | 0.57812504 | 0.582484258 | 0.588763056 | 0.596729076 | 0.605454319 | 0.614080275 | 0.622027433 | 0.62854671 | 0.634281143 | 0.640011219 | 0.64588852 | 0.651787987 | 0.656714457 | 0.661017053 |
| Peru | 0.510419852 | 0.512161525 | 0.513821552 | 0.515981506 | 0.51930608 | 0.523640527 | 0.528312042 | 0.533576491 | 0.538721717 | 0.543853377 | 0.548952201 | 0.553775453 | 0.558853263 | 0.563871072 | 0.569063024 | 0.574517001 | 0.580503365 | 0.586958489 | 0.593750811 | 0.599954993 | 0.606520932 | 0.613051221 | 0.619355264 | 0.625519024 | 0.631114354 | 0.636278271 | 0.641285279 | 0.646180855 | 0.650977317 | 0.655433735 | 0.658672244 | 0.662054037 |
| Caribbean | 0.518111381 | 0.522788586 | 0.526984192 | 0.530506786 | 0.533584311 | 0.536650593 | 0.539775932 | 0.543302886 | 0.547365066 | 0.552060172 | 0.557368943 | 0.563110488 | 0.569068592 | 0.574888622 | 0.580533315 | 0.58575799 | 0.590602336 | 0.594709606 | 0.59829691 | 0.601654828 | 0.605480076 | 0.609525046 | 0.613254721 | 0.61687685 | 0.620442645 | 0.624074667 | 0.627582074 | 0.630788066 | 0.633972166 | 0.63732123 | 0.639708532 | 0.642003055 |
| Antigua and Barbuda | 0.612104591 | 0.618817129 | 0.624695818 | 0.630075854 | 0.634768955 | 0.638008629 | 0.641647192 | 0.645895591 | 0.650614287 | 0.655534994 | 0.660819064 | 0.665620632 | 0.670360801 | 0.67531149 | 0.680412429 | 0.685486437 | 0.690826021 | 0.696360039 | 0.702175362 | 0.707128564 | 0.711432246 | 0.715489989 | 0.719509323 | 0.722864978 | 0.725585978 | 0.728372762 | 0.731598944 | 0.734963713 | 0.738879106 | 0.74305296 | 0.74634533 | 0.749886887 |
| The Bahamas | 0.693509268 | 0.689126784 | 0.687343838 | 0.693816501 | 0.706819892 | 0.719381607 | 0.728478844 | 0.734781215 | 0.739281293 | 0.741622848 | 0.742517946 | 0.743592882 | 0.746250322 | 0.749916714 | 0.753759643 | 0.756782661 | 0.759185939 | 0.761990997 | 0.765590503 | 0.769561674 | 0.77411981 | 0.778281891 | 0.781838639 | 0.784623911 | 0.787253492 | 0.789956145 | 0.792738696 | 0.795347181 | 0.798179034 | 0.80103307 | 0.802948018 | 0.805020668 |
| Barbados | 0.653582517 | 0.656654073 | 0.662446635 | 0.668815012 | 0.673409365 | 0.676012247 | 0.677193054 | 0.678644826 | 0.679827167 | 0.680398459 | 0.681304199 | 0.683329389 | 0.687807576 | 0.693235962 | 0.698029703 | 0.701510049 | 0.703856186 | 0.705871649 | 0.708514495 | 0.712143362 | 0.716208546 | 0.720060687 | 0.724162458 | 0.72782617 | 0.730383287 | 0.732602993 | 0.734912535 | 0.737157255 | 0.739531437 | 0.742239309 | 0.744366646 | 0.746748764 |
| Belize | 0.423726992 | 0.433834392 | 0.444198507 | 0.455258577 | 0.465729308 | 0.475278511 | 0.482702165 | 0.488020129 | 0.491819335 | 0.495070196 | 0.49864373 | 0.502399434 | 0.506871655 | 0.512679459 | 0.519697333 | 0.527231062 | 0.534889621 | 0.541900694 | 0.548093223 | 0.553465869 | 0.558546893 | 0.563440363 | 0.568456236 | 0.573236457 | 0.578091326 | 0.583279094 | 0.588275912 | 0.59333729 | 0.598512015 | 0.603351601 | 0.607060046 | 0.610229002 |
| Bermuda | 0.696451196 | 0.700276851 | 0.703984095 | 0.707678222 | 0.711068998 | 0.714468828 | 0.717831671 | 0.721642813 | 0.725853528 | 0.730539823 | 0.735996502 | 0.741918908 | 0.747544503 | 0.753305163 | 0.759008885 | 0.764690903 | 0.77059677 | 0.776114264 | 0.780358325 | 0.784759107 | 0.789181467 | 0.793380524 | 0.79748487 | 0.801388201 | 0.804923407 | 0.807998795 | 0.810550842 | 0.812807229 | 0.814892802 | 0.817000338 | 0.81920342 | 0.821365422 |
| Cuba | 0.558019071 | 0.563413638 | 0.566169006 | 0.565441047 | 0.563186313 | 0.560765902 | 0.559346329 | 0.558924568 | 0.55936322 | 0.561820953 | 0.566676902 | 0.572753861 | 0.57996829 | 0.587647762 | 0.594348715 | 0.599863799 | 0.604970567 | 0.607618099 | 0.609400923 | 0.611775641 | 0.615261262 | 0.62038669 | 0.626156025 | 0.631839801 | 0.637304129 | 0.642818963 | 0.647907866 | 0.652309286 | 0.656858654 | 0.661674717 | 0.665210747 | 0.668729864 |
| Dominica | 0.56360259 | 0.565237843 | 0.569570684 | 0.578811811 | 0.590119147 | 0.601986932 | 0.61438205 | 0.625057353 | 0.633017994 | 0.639453915 | 0.645830937 | 0.652368567 | 0.658423554 | 0.663678868 | 0.668952132 | 0.674549077 | 0.680342482 | 0.685415917 | 0.690197519 | 0.694461869 | 0.698489193 | 0.70246326 | 0.706870608 | 0.712210475 | 0.718455556 | 0.724785536 | 0.731760801 | 0.736062473 | 0.738935954 | 0.742132066 | 0.744419944 | 0.746967185 |
| Dominican Republic | 0.442654076 | 0.446144578 | 0.449953081 | 0.454229297 | 0.458620614 | 0.463313869 | 0.468516938 | 0.474504389 | 0.481170939 | 0.488178541 | 0.495468586 | 0.502775415 | 0.510298019 | 0.517395574 | 0.524543275 | 0.532228649 | 0.54022269 | 0.548187021 | 0.555371313 | 0.561783754 | 0.568130582 | 0.573834813 | 0.578966381 | 0.583625825 | 0.588236743 | 0.592924164 | 0.597619609 | 0.602140291 | 0.606960839 | 0.611913021 | 0.615635625 | 0.619388201 |
| Grenada | 0.436734419 | 0.445887953 | 0.455910058 | 0.46599489 | 0.476410341 | 0.486558999 | 0.496589808 | 0.506217602 | 0.516590019 | 0.527333411 | 0.538388116 | 0.54905897 | 0.559473128 | 0.569718037 | 0.578487456 | 0.587158877 | 0.59405452 | 0.600766336 | 0.606879282 | 0.611805257 | 0.616486997 | 0.620978521 | 0.624977512 | 0.629048336 | 0.633734696 | 0.638936084 | 0.644335234 | 0.649831522 | 0.655372772 | 0.660808512 | 0.665086347 | 0.668993028 |
| Guyana | 0.460430129 | 0.462677069 | 0.466753989 | 0.472174229 | 0.478992924 | 0.486573457 | 0.495216036 | 0.504362779 | 0.513068898 | 0.521595978 | 0.528693992 | 0.534869806 | 0.540514273 | 0.545516473 | 0.55032637 | 0.554844797 | 0.559465611 | 0.564541135 | 0.569595206 | 0.574769931 | 0.580083911 | 0.585688269 | 0.591336582 | 0.596826295 | 0.602136511 | 0.607605487 | 0.61391436 | 0.620602607 | 0.627167041 | 0.633640347 | 0.642284645 | 0.650812335 |
| Haiti | 0.31033463 | 0.315455441 | 0.320389979 | 0.324895851 | 0.328399456 | 0.332999152 | 0.338229396 | 0.343880049 | 0.349698073 | 0.355980743 | 0.362572209 | 0.368643508 | 0.374473325 | 0.380067255 | 0.385061512 | 0.38984542 | 0.394322001 | 0.398973441 | 0.403285871 | 0.407944424 | 0.411840105 | 0.41617719 | 0.420021528 | 0.423985549 | 0.427940902 | 0.43175676 | 0.435288722 | 0.438541003 | 0.441678925 | 0.444328854 | 0.446390614 | 0.448278285 |
| Jamaica | 0.534781234 | 0.53959448 | 0.545088753 | 0.551218492 | 0.557643773 | 0.564315038 | 0.570940131 | 0.577212091 | 0.583048035 | 0.588846467 | 0.594592917 | 0.600192022 | 0.605591748 | 0.610877424 | 0.615836278 | 0.620400057 | 0.62501886 | 0.62954947 | 0.633967851 | 0.638110835 | 0.642129388 | 0.64639583 | 0.65058376 | 0.654573624 | 0.658505341 | 0.662465809 | 0.666469279 | 0.670291045 | 0.674075313 | 0.677778539 | 0.680567177 | 0.683263064 |
| Puerto Rico | 0.658758146 | 0.66326172 | 0.667272192 | 0.671024105 | 0.67545081 | 0.68067395 | 0.685865893 | 0.691274382 | 0.697040445 | 0.702850671 | 0.710035652 | 0.718955861 | 0.725956856 | 0.730475138 | 0.734219546 | 0.737742266 | 0.741629431 | 0.745883807 | 0.750922167 | 0.756361695 | 0.761763253 | 0.766784882 | 0.772311804 | 0.778461162 | 0.785120345 | 0.791800167 | 0.799084083 | 0.806648961 | 0.812906066 | 0.818843191 | 0.822918436 | 0.825525847 |
| Saint Kitts and Nevis | 0.580685877 | 0.59025495 | 0.59918719 | 0.607235892 | 0.614514658 | 0.620716498 | 0.626207411 | 0.63127474 | 0.635539988 | 0.639713739 | 0.644353377 | 0.649226653 | 0.654593409 | 0.659925312 | 0.66756813 | 0.679145457 | 0.690078667 | 0.697727628 | 0.704141494 | 0.709499498 | 0.714460415 | 0.719324381 | 0.723436152 | 0.726706593 | 0.729530219 | 0.732220672 | 0.735566138 | 0.739470681 | 0.743736461 | 0.748152066 | 0.751568409 | 0.754987055 |
| Saint Lucia | 0.49629657 | 0.505975555 | 0.515368261 | 0.524234949 | 0.532731083 | 0.541479794 | 0.550242412 | 0.558293461 | 0.566138703 | 0.573428555 | 0.579788896 | 0.585087558 | 0.590167578 | 0.595675146 | 0.601625482 | 0.607239866 | 0.6127553 | 0.617994714 | 0.623273001 | 0.628169849 | 0.632782905 | 0.637439442 | 0.641567127 | 0.645153079 | 0.648743507 | 0.65215748 | 0.65565334 | 0.659341346 | 0.663086682 | 0.666840359 | 0.66972049 | 0.672509735 |
| Saint Vincent and the Grenadines | 0.475930186 | 0.481075658 | 0.485977871 | 0.490998791 | 0.496379132 | 0.50298609 | 0.509527158 | 0.515482619 | 0.521209679 | 0.527099552 | 0.53317329 | 0.539455712 | 0.546063616 | 0.552693724 | 0.558663543 | 0.563588116 | 0.568221748 | 0.572819052 | 0.577611145 | 0.582549107 | 0.587190384 | 0.590259096 | 0.592153908 | 0.594614075 | 0.598378672 | 0.603754135 | 0.61005921 | 0.616438473 | 0.622634944 | 0.628325986 | 0.632860904 | 0.637195963 |
| Suriname | 0.502054305 | 0.506655795 | 0.510959772 | 0.514419437 | 0.517650245 | 0.521002622 | 0.525059318 | 0.529750812 | 0.534599696 | 0.538932791 | 0.543660023 | 0.548683524 | 0.553879432 | 0.559418373 | 0.565109708 | 0.570462186 | 0.575647048 | 0.580654574 | 0.585644825 | 0.590512805 | 0.595469745 | 0.600356336 | 0.60474187 | 0.608910612 | 0.612704423 | 0.616097315 | 0.618868088 | 0.62168418 | 0.624708011 | 0.627896781 | 0.630683705 | 0.633665739 |
| Trinidad and Tobago | 0.62397015 | 0.628758918 | 0.634039552 | 0.639191914 | 0.64424291 | 0.649220055 | 0.654169455 | 0.659293633 | 0.664507714 | 0.670244574 | 0.676683835 | 0.682763494 | 0.688872271 | 0.695828341 | 0.702610809 | 0.708520985 | 0.71460021 | 0.720196417 | 0.725720702 | 0.729538892 | 0.733237077 | 0.736802405 | 0.739999073 | 0.743593955 | 0.747338963 | 0.751510682 | 0.754953348 | 0.758226988 | 0.761241991 | 0.764169599 | 0.766422081 | 0.768763254 |
| Virgin Islands | 0.655160856 | 0.664018659 | 0.671081871 | 0.676872745 | 0.681982223 | 0.686730211 | 0.691247699 | 0.695598204 | 0.699768686 | 0.703949342 | 0.708436565 | 0.713751115 | 0.724398272 | 0.73460985 | 0.744613598 | 0.754267558 | 0.763447496 | 0.772180593 | 0.780342623 | 0.787055146 | 0.793219696 | 0.798071698 | 0.801092851 | 0.803405789 | 0.805641378 | 0.807889245 | 0.810199044 | 0.812405172 | 0.81482544 | 0.817429008 | 0.819601712 | 0.821830853 |
| Central Latin America | 0.48578741 | 0.489558534 | 0.494110188 | 0.499417966 | 0.505060641 | 0.509874246 | 0.514693467 | 0.520170671 | 0.526131813 | 0.531807023 | 0.537300373 | 0.542439835 | 0.547134354 | 0.551446098 | 0.556011499 | 0.560749531 | 0.565648691 | 0.570707268 | 0.575839326 | 0.579940059 | 0.58425317 | 0.589307552 | 0.594827165 | 0.600731022 | 0.60650929 | 0.611815329 | 0.617086095 | 0.622442429 | 0.627764642 | 0.632847071 | 0.636995887 | 0.6406851 |
| Colombia | 0.480720054 | 0.482339894 | 0.484572359 | 0.488229373 | 0.493278182 | 0.499102757 | 0.504986969 | 0.511079502 | 0.517022505 | 0.52243594 | 0.527839094 | 0.533076501 | 0.538189342 | 0.543265131 | 0.548601832 | 0.554089191 | 0.560182735 | 0.566822644 | 0.573494652 | 0.579876469 | 0.586408108 | 0.593340477 | 0.600254238 | 0.607250637 | 0.614151923 | 0.620889228 | 0.627554497 | 0.634130359 | 0.640255072 | 0.646038852 | 0.650855354 | 0.655442913 |
| Costa Rica | 0.534125181 | 0.539342164 | 0.544624238 | 0.549946626 | 0.55517125 | 0.560547086 | 0.565180862 | 0.570128417 | 0.576067051 | 0.582229492 | 0.588243191 | 0.594889115 | 0.600894433 | 0.605765687 | 0.610315699 | 0.614412619 | 0.617850162 | 0.621200571 | 0.62570391 | 0.631291379 | 0.636595426 | 0.641201295 | 0.646574093 | 0.651945813 | 0.656890456 | 0.662030136 | 0.667430092 | 0.673621392 | 0.681296147 | 0.690049088 | 0.696194605 | 0.700340477 |
| El Salvador | 0.373057975 | 0.374855325 | 0.377076643 | 0.380307326 | 0.385149094 | 0.391780266 | 0.400178468 | 0.410455826 | 0.42142119 | 0.432733148 | 0.443962666 | 0.454177659 | 0.46296886 | 0.470397413 | 0.476536664 | 0.482041719 | 0.486826 | 0.490809752 | 0.495077739 | 0.499489714 | 0.504171335 | 0.508969239 | 0.513396874 | 0.518121908 | 0.523468901 | 0.52938695 | 0.535650269 | 0.541998957 | 0.548143174 | 0.554031648 | 0.558919894 | 0.563775188 |
| Guatemala | 0.311792455 | 0.314329484 | 0.319165481 | 0.326840819 | 0.3334691 | 0.339642101 | 0.346961212 | 0.355995963 | 0.366461193 | 0.374455316 | 0.379877297 | 0.386927686 | 0.396899639 | 0.408234164 | 0.419777065 | 0.430327529 | 0.438856376 | 0.447245965 | 0.456541865 | 0.464003077 | 0.470477364 | 0.476909684 | 0.481862853 | 0.487286692 | 0.494027188 | 0.500655607 | 0.507149792 | 0.513735682 | 0.520995469 | 0.52863047 | 0.534571414 | 0.539972424 |
| Honduras | 0.332042889 | 0.337034649 | 0.342305657 | 0.347838446 | 0.353164852 | 0.358787268 | 0.364328424 | 0.369983894 | 0.375950111 | 0.381752316 | 0.387988053 | 0.394174012 | 0.400371798 | 0.406668381 | 0.413061816 | 0.419612332 | 0.426352997 | 0.433240315 | 0.439998164 | 0.446151054 | 0.452382226 | 0.458700329 | 0.464925155 | 0.470766833 | 0.476587799 | 0.482294176 | 0.487705932 | 0.493130694 | 0.498657227 | 0.504156505 | 0.508669246 | 0.513037248 |
| Mexico | 0.504996083 | 0.50951067 | 0.514638057 | 0.520493167 | 0.526749241 | 0.531554141 | 0.536530827 | 0.541988171 | 0.547842121 | 0.55381337 | 0.559532547 | 0.564774176 | 0.570113531 | 0.575698215 | 0.580984982 | 0.58522408 | 0.588543226 | 0.591447408 | 0.594454558 | 0.596684358 | 0.599527028 | 0.603646095 | 0.608914289 | 0.615089993 | 0.621857405 | 0.628896724 | 0.635925349 | 0.642667198 | 0.649053317 | 0.655095338 | 0.660119062 | 0.664575304 |
| Aguascalientes | 0.524645983 | 0.529086308 | 0.533396884 | 0.538117209 | 0.543268306 | 0.547452751 | 0.552198621 | 0.55740598 | 0.563132208 | 0.56946169 | 0.57602722 | 0.582381993 | 0.588920804 | 0.595282514 | 0.600983949 | 0.605285095 | 0.608663033 | 0.611516294 | 0.614244322 | 0.616218854 | 0.619019148 | 0.622938826 | 0.627726394 | 0.633398087 | 0.639769652 | 0.646386925 | 0.652924602 | 0.659321054 | 0.665505023 | 0.671483231 | 0.676568177 | 0.681159284 |
| Baja California | 0.557776897 | 0.559365698 | 0.561957605 | 0.565692125 | 0.570004399 | 0.573086528 | 0.576674209 | 0.581389375 | 0.587181672 | 0.593318034 | 0.599338278 | 0.60536455 | 0.611858515 | 0.618190654 | 0.623387595 | 0.627120689 | 0.629873396 | 0.632240398 | 0.63481885 | 0.636585989 | 0.638887626 | 0.642666891 | 0.647720868 | 0.653512649 | 0.659712761 | 0.666404319 | 0.673564487 | 0.680559442 | 0.687327174 | 0.693785764 | 0.699235917 | 0.704081972 |
| Baja California Sur | 0.547365468 | 0.552228229 | 0.55700866 | 0.562550597 | 0.568593089 | 0.573090747 | 0.577591103 | 0.58235863 | 0.587326778 | 0.59177257 | 0.59584449 | 0.600069561 | 0.604864616 | 0.610486148 | 0.616221245 | 0.621039641 | 0.624878618 | 0.628269748 | 0.631833803 | 0.63507846 | 0.63924246 | 0.64472575 | 0.651117283 | 0.658030008 | 0.66517874 | 0.672754451 | 0.680229753 | 0.687302146 | 0.693906495 | 0.700063493 | 0.705169781 | 0.709694281 |
| Campeche | 0.467809577 | 0.473008376 | 0.479048708 | 0.486466285 | 0.494683355 | 0.501700286 | 0.509180613 | 0.516994964 | 0.525105941 | 0.533195202 | 0.540819834 | 0.547664248 | 0.553998316 | 0.560108386 | 0.565684719 | 0.570211518 | 0.57369097 | 0.576955406 | 0.580466987 | 0.583587218 | 0.587951869 | 0.593968908 | 0.6012821 | 0.609450135 | 0.618209468 | 0.626665171 | 0.634619859 | 0.642127139 | 0.649084898 | 0.655523252 | 0.660794872 | 0.665363431 |
| Chiapas | 0.380122713 | 0.385087423 | 0.391658899 | 0.399667728 | 0.408412838 | 0.415031544 | 0.420853035 | 0.42670943 | 0.432571889 | 0.438500296 | 0.44413082 | 0.449415253 | 0.45517242 | 0.462033656 | 0.469121556 | 0.475293991 | 0.48064481 | 0.485135611 | 0.489432041 | 0.493138867 | 0.497678027 | 0.503601464 | 0.510552966 | 0.518277029 | 0.526335056 | 0.534333305 | 0.54174283 | 0.548605065 | 0.554845922 | 0.560639715 | 0.56553923 | 0.569940909 |
| Chihuahua | 0.526085567 | 0.528369742 | 0.530621214 | 0.533088262 | 0.535463962 | 0.536770448 | 0.538957206 | 0.542141191 | 0.546449106 | 0.551381625 | 0.556678924 | 0.56253594 | 0.569326249 | 0.576376225 | 0.582840303 | 0.588061588 | 0.592050911 | 0.595463846 | 0.599112449 | 0.60119468 | 0.603157385 | 0.606322463 | 0.61056631 | 0.616353905 | 0.623151157 | 0.631064058 | 0.639130455 | 0.646937818 | 0.654409546 | 0.661581336 | 0.667813093 | 0.673386571 |
| Coahuila | 0.524303567 | 0.529617755 | 0.535356954 | 0.541523107 | 0.547873874 | 0.552441837 | 0.557008538 | 0.562049036 | 0.567506083 | 0.573174326 | 0.578834331 | 0.584025896 | 0.589064103 | 0.594483897 | 0.59954588 | 0.603330643 | 0.605902019 | 0.607651946 | 0.609316907 | 0.60973611 | 0.610414717 | 0.612445468 | 0.615759576 | 0.621034242 | 0.627946172 | 0.635486256 | 0.643410657 | 0.651313103 | 0.658890944 | 0.666104717 | 0.672191562 | 0.677562875 |
| Colima | 0.541152933 | 0.547263727 | 0.553181144 | 0.559053374 | 0.564887752 | 0.569709808 | 0.575167134 | 0.580692342 | 0.586060065 | 0.591773151 | 0.597727773 | 0.602852888 | 0.60751331 | 0.612040981 | 0.616151308 | 0.619768994 | 0.622814155 | 0.625615456 | 0.628623182 | 0.631027977 | 0.634323093 | 0.638716119 | 0.644012382 | 0.650301568 | 0.657202269 | 0.664437333 | 0.671545896 | 0.678179716 | 0.684324875 | 0.69002339 | 0.694792765 | 0.699116771 |
| Durango | 0.471994856 | 0.475753101 | 0.480095953 | 0.484867553 | 0.489882342 | 0.494135503 | 0.499273794 | 0.505544681 | 0.512813591 | 0.519955161 | 0.526474741 | 0.532785066 | 0.539389849 | 0.546365524 | 0.552702061 | 0.55723541 | 0.5603506 | 0.562746726 | 0.565147603 | 0.566877407 | 0.569331973 | 0.573137362 | 0.578345449 | 0.584783995 | 0.591980566 | 0.599643365 | 0.607364309 | 0.614755714 | 0.621769432 | 0.628394043 | 0.63414276 | 0.639324841 |
| Guanajuato | 0.463111395 | 0.470188385 | 0.477361521 | 0.484588445 | 0.491856735 | 0.498138011 | 0.505096921 | 0.512397226 | 0.520005532 | 0.52749219 | 0.534586178 | 0.541008193 | 0.547206378 | 0.553237819 | 0.558739531 | 0.563156155 | 0.566699511 | 0.569683144 | 0.572745176 | 0.575180144 | 0.578376075 | 0.582978389 | 0.588835861 | 0.595149587 | 0.601773573 | 0.608789999 | 0.616296163 | 0.62342171 | 0.630175932 | 0.636525148 | 0.641782941 | 0.646374308 |
| Guerrero | 0.404457237 | 0.408255466 | 0.412470402 | 0.417130337 | 0.422254534 | 0.42746483 | 0.433915892 | 0.440722245 | 0.447597172 | 0.454441561 | 0.460932543 | 0.467201186 | 0.473727389 | 0.480435488 | 0.486645373 | 0.491401424 | 0.495283284 | 0.499075917 | 0.503480186 | 0.507508519 | 0.512481565 | 0.518739203 | 0.526118007 | 0.534328326 | 0.542843318 | 0.550902215 | 0.55826047 | 0.56494497 | 0.570892933 | 0.576313459 | 0.58083578 | 0.584850472 |
| Hidalgo | 0.433772282 | 0.439869628 | 0.446651297 | 0.454131297 | 0.462109622 | 0.469017512 | 0.476188075 | 0.483745892 | 0.491469785 | 0.498988995 | 0.505866568 | 0.512114152 | 0.518289962 | 0.524812974 | 0.531181295 | 0.536413155 | 0.540686983 | 0.544637507 | 0.548861677 | 0.552658268 | 0.557419981 | 0.563486078 | 0.570720526 | 0.578761941 | 0.587238198 | 0.595853076 | 0.603878714 | 0.611313713 | 0.618106205 | 0.624278463 | 0.629196656 | 0.633478453 |
| Jalisco | 0.519722519 | 0.525127651 | 0.530888564 | 0.537022622 | 0.543348927 | 0.548287987 | 0.553519239 | 0.559297302 | 0.565395705 | 0.571402201 | 0.57701229 | 0.582020377 | 0.586946709 | 0.59202047 | 0.596913283 | 0.600728155 | 0.603888054 | 0.606746472 | 0.609848506 | 0.611995901 | 0.614668192 | 0.618402798 | 0.623161302 | 0.628784051 | 0.635018428 | 0.641495644 | 0.648007068 | 0.654350075 | 0.660476696 | 0.66641179 | 0.671425968 | 0.675998373 |
| México | 0.530648662 | 0.535123213 | 0.540586547 | 0.547051784 | 0.5538444 | 0.558051963 | 0.561445872 | 0.564846598 | 0.568399018 | 0.572593908 | 0.576919422 | 0.581063813 | 0.585827362 | 0.591204983 | 0.59686804 | 0.60177977 | 0.605676992 | 0.609108827 | 0.612422629 | 0.614951323 | 0.618019806 | 0.62217966 | 0.627355172 | 0.633324903 | 0.639822518 | 0.646415315 | 0.653153957 | 0.659777749 | 0.666145876 | 0.67220359 | 0.677174495 | 0.681587605 |
| Mexico City | 0.618325598 | 0.622830107 | 0.628087498 | 0.634543876 | 0.64174362 | 0.646990861 | 0.652117183 | 0.657804747 | 0.663818173 | 0.669722375 | 0.67514284 | 0.679619168 | 0.683929756 | 0.688181451 | 0.692029523 | 0.694922318 | 0.696654237 | 0.697880872 | 0.699109289 | 0.699776148 | 0.701055543 | 0.703594959 | 0.707571637 | 0.712722255 | 0.718751796 | 0.725094753 | 0.73171036 | 0.738340934 | 0.744807938 | 0.75091056 | 0.755739333 | 0.759848414 |
| Michoacán de Ocampo | 0.454293326 | 0.461317384 | 0.468512261 | 0.475423283 | 0.481909927 | 0.487128237 | 0.492489666 | 0.498260802 | 0.504338873 | 0.510458735 | 0.516531607 | 0.522100214 | 0.527481598 | 0.532961239 | 0.537894937 | 0.541886157 | 0.545401418 | 0.548370313 | 0.551183857 | 0.55309694 | 0.555604243 | 0.55941862 | 0.564251363 | 0.569764503 | 0.575748634 | 0.582001574 | 0.587998941 | 0.593747183 | 0.599218923 | 0.604468966 | 0.60894406 | 0.613082024 |
| Morelos | 0.520991486 | 0.527159101 | 0.533231346 | 0.539467741 | 0.545786953 | 0.550933604 | 0.556407268 | 0.56197648 | 0.567471342 | 0.573149825 | 0.579158465 | 0.584280718 | 0.588789016 | 0.593023377 | 0.5964867 | 0.598764947 | 0.600441668 | 0.60198986 | 0.603921546 | 0.60521237 | 0.607396193 | 0.610828628 | 0.615390091 | 0.621139352 | 0.627724504 | 0.634769513 | 0.641834922 | 0.64865498 | 0.655017622 | 0.660904708 | 0.66575519 | 0.670074046 |
| Nayarit | 0.47346391 | 0.477936384 | 0.482866787 | 0.48830129 | 0.494012839 | 0.499079268 | 0.505010842 | 0.511456927 | 0.518532192 | 0.526147165 | 0.533618024 | 0.540843166 | 0.548299257 | 0.556183269 | 0.563724435 | 0.569974486 | 0.575046738 | 0.578529229 | 0.581304633 | 0.583584882 | 0.586998344 | 0.591943499 | 0.598321689 | 0.605631774 | 0.613481135 | 0.621476456 | 0.629016128 | 0.636207869 | 0.642864298 | 0.648908953 | 0.653838282 | 0.658178913 |
| Nuevo León | 0.581722309 | 0.585732591 | 0.589944071 | 0.594231065 | 0.598487076 | 0.601406582 | 0.60454057 | 0.608331656 | 0.612985238 | 0.61816602 | 0.623406773 | 0.62844154 | 0.633627286 | 0.638517602 | 0.64273628 | 0.645742758 | 0.647922097 | 0.649693422 | 0.651387127 | 0.652257152 | 0.653703738 | 0.656510893 | 0.660565959 | 0.665456211 | 0.670973116 | 0.676853529 | 0.682831573 | 0.688694968 | 0.694502714 | 0.700331787 | 0.705437899 | 0.710141817 |
| Oaxaca | 0.414302017 | 0.418255379 | 0.422765462 | 0.42802641 | 0.433694002 | 0.43843657 | 0.443780236 | 0.449239643 | 0.454492855 | 0.459714435 | 0.464732105 | 0.470016971 | 0.475966737 | 0.482897715 | 0.48994688 | 0.495717829 | 0.500497363 | 0.505479919 | 0.51104287 | 0.515903124 | 0.521531623 | 0.527721418 | 0.534276773 | 0.541217766 | 0.54829387 | 0.555487626 | 0.562414998 | 0.568826145 | 0.574754064 | 0.58032331 | 0.584912702 | 0.589041897 |
| Puebla | 0.449529952 | 0.456266656 | 0.463574103 | 0.471067188 | 0.478342588 | 0.483552566 | 0.488206112 | 0.493198426 | 0.498537683 | 0.504066375 | 0.509469106 | 0.514762484 | 0.520456303 | 0.526620783 | 0.532464782 | 0.537345856 | 0.541504469 | 0.545157057 | 0.548772249 | 0.551736088 | 0.55548434 | 0.560389676 | 0.566378282 | 0.573133989 | 0.580223492 | 0.587290091 | 0.594166026 | 0.600684558 | 0.60680583 | 0.61261265 | 0.617542682 | 0.622031827 |
| Querétaro | 0.507320191 | 0.512180108 | 0.517465864 | 0.523870787 | 0.530888522 | 0.536820255 | 0.5430477 | 0.550031272 | 0.557462457 | 0.565822742 | 0.574711924 | 0.582626143 | 0.59020629 | 0.596983404 | 0.602863259 | 0.607635277 | 0.611502653 | 0.614934641 | 0.618292914 | 0.620654926 | 0.623370743 | 0.627407356 | 0.632676857 | 0.638470148 | 0.644559631 | 0.650679751 | 0.656909427 | 0.662852939 | 0.668477755 | 0.673858338 | 0.678404251 | 0.682558115 |
| Quintana Roo | 0.491221072 | 0.499658554 | 0.508849401 | 0.518966035 | 0.529275714 | 0.538097916 | 0.547338794 | 0.55607698 | 0.564034086 | 0.571284805 | 0.577776959 | 0.583471002 | 0.588866073 | 0.594279019 | 0.599222152 | 0.603065647 | 0.606195187 | 0.609285337 | 0.612780197 | 0.615827869 | 0.619702596 | 0.624558609 | 0.630125311 | 0.636228607 | 0.642526216 | 0.648966318 | 0.655464105 | 0.661526715 | 0.667161201 | 0.672472917 | 0.6769749 | 0.681068671 |
| San Luis Potosí | 0.450145657 | 0.455178109 | 0.460865887 | 0.467886019 | 0.475908899 | 0.482813693 | 0.490262913 | 0.498007231 | 0.505981191 | 0.513788587 | 0.521069375 | 0.527813256 | 0.534464774 | 0.541379965 | 0.548081942 | 0.554161279 | 0.559641755 | 0.564862148 | 0.570231516 | 0.57481416 | 0.580103043 | 0.585996039 | 0.592324334 | 0.599207895 | 0.606449304 | 0.613519075 | 0.620142948 | 0.626334424 | 0.632144478 | 0.637675324 | 0.642343523 | 0.646651514 |
| Sinaloa | 0.51239837 | 0.515724538 | 0.519110064 | 0.523493293 | 0.529171679 | 0.533824543 | 0.539021242 | 0.545017962 | 0.551864493 | 0.558729337 | 0.565065735 | 0.570694201 | 0.576212372 | 0.58228089 | 0.588235413 | 0.59291479 | 0.59635723 | 0.599432775 | 0.602868433 | 0.60536385 | 0.608455891 | 0.612997256 | 0.618687811 | 0.6254509 | 0.632896913 | 0.640518414 | 0.647646773 | 0.654486312 | 0.661038187 | 0.667309471 | 0.672564729 | 0.677164972 |
| Sonora | 0.546866387 | 0.549324775 | 0.552173274 | 0.55580514 | 0.560149323 | 0.563537758 | 0.567396802 | 0.572244411 | 0.578181096 | 0.584797789 | 0.59178041 | 0.597856953 | 0.603445271 | 0.609295405 | 0.614582729 | 0.618973689 | 0.622556468 | 0.625393801 | 0.628146267 | 0.629637533 | 0.631558168 | 0.63550766 | 0.64130954 | 0.648655569 | 0.657051011 | 0.666061785 | 0.675053347 | 0.683672603 | 0.691868679 | 0.69948653 | 0.705606926 | 0.710679605 |
| Tabasco | 0.463410559 | 0.469836195 | 0.476814745 | 0.483912394 | 0.490924045 | 0.497132636 | 0.504137673 | 0.511622806 | 0.519274266 | 0.526820229 | 0.533891401 | 0.539806776 | 0.545232731 | 0.550867139 | 0.556198718 | 0.560900704 | 0.564831209 | 0.568456563 | 0.572242472 | 0.575436171 | 0.579541169 | 0.585036911 | 0.591667373 | 0.598803513 | 0.606364088 | 0.613994159 | 0.621142358 | 0.627859811 | 0.634108399 | 0.639853147 | 0.644601541 | 0.648728024 |
| Tamaulipas | 0.538947104 | 0.544638461 | 0.549815236 | 0.555307892 | 0.560934086 | 0.566102025 | 0.572559594 | 0.578748304 | 0.584483131 | 0.588908393 | 0.592208153 | 0.595028793 | 0.597789247 | 0.600775706 | 0.603452249 | 0.605472662 | 0.607218691 | 0.609296972 | 0.612003718 | 0.613732134 | 0.615760832 | 0.619319504 | 0.624105707 | 0.630000658 | 0.636708169 | 0.643909786 | 0.651241453 | 0.658314794 | 0.665132558 | 0.671697841 | 0.677236215 | 0.682086111 |
| Tlaxcala | 0.467929212 | 0.475615886 | 0.483461998 | 0.491556131 | 0.499593393 | 0.505981625 | 0.512412878 | 0.519057294 | 0.525890662 | 0.532981824 | 0.540011329 | 0.546454115 | 0.55282779 | 0.559260866 | 0.565431352 | 0.570556345 | 0.574856425 | 0.578762877 | 0.582753815 | 0.585897616 | 0.589621306 | 0.594073324 | 0.599160925 | 0.604872999 | 0.610916335 | 0.617105246 | 0.623161938 | 0.628896509 | 0.634275651 | 0.639374008 | 0.64374855 | 0.64782243 |
| Veracruz de Ignacio de la Llave | 0.457310627 | 0.460899618 | 0.46549269 | 0.47103541 | 0.477123296 | 0.481811683 | 0.486716706 | 0.492450681 | 0.498808516 | 0.504983565 | 0.510222177 | 0.514760744 | 0.519493771 | 0.525066464 | 0.530785483 | 0.5355783 | 0.539599456 | 0.54351481 | 0.547835221 | 0.551545019 | 0.555915035 | 0.561465291 | 0.568118918 | 0.575400903 | 0.58309252 | 0.591075796 | 0.598822571 | 0.606077278 | 0.612787972 | 0.618952266 | 0.623884762 | 0.628136965 |
| Yucatán | 0.469101155 | 0.474451956 | 0.480864076 | 0.488652617 | 0.497235252 | 0.504223762 | 0.511318755 | 0.518418074 | 0.525292033 | 0.531390302 | 0.536690848 | 0.54134838 | 0.545732079 | 0.551110792 | 0.55712125 | 0.562705424 | 0.567892184 | 0.573015048 | 0.578311588 | 0.582671335 | 0.587482585 | 0.593105684 | 0.599330714 | 0.606036724 | 0.61308507 | 0.620080592 | 0.626493436 | 0.632566043 | 0.638357183 | 0.643936744 | 0.648671616 | 0.653050809 |
| Zacatecas | 0.482045818 | 0.48758905 | 0.49321387 | 0.498885367 | 0.504545124 | 0.508949999 | 0.51353475 | 0.518745791 | 0.524468712 | 0.530542762 | 0.536712192 | 0.542570642 | 0.548497394 | 0.554239571 | 0.559473843 | 0.563412523 | 0.566561093 | 0.56944972 | 0.572571967 | 0.574471258 | 0.576589213 | 0.579950064 | 0.584363277 | 0.589726705 | 0.595650477 | 0.601934202 | 0.608260972 | 0.614309889 | 0.620036873 | 0.625543402 | 0.630338787 | 0.634773655 |
| Nicaragua | 0.346035235 | 0.351740622 | 0.357712704 | 0.363562069 | 0.369727968 | 0.376625345 | 0.384087266 | 0.391775893 | 0.399807535 | 0.408085965 | 0.416108363 | 0.423790295 | 0.430813755 | 0.437248152 | 0.443274511 | 0.448844739 | 0.453950499 | 0.459042798 | 0.463837699 | 0.467961839 | 0.472172 | 0.476593466 | 0.4812969 | 0.48610547 | 0.491207346 | 0.496391663 | 0.501853345 | 0.507482022 | 0.512338228 | 0.516597046 | 0.52029267 | 0.523958472 |
| Panama | 0.546048123 | 0.550484218 | 0.554972755 | 0.559168666 | 0.561714534 | 0.563353023 | 0.56531007 | 0.569395939 | 0.574697318 | 0.580432842 | 0.587378393 | 0.594535701 | 0.600138677 | 0.604043365 | 0.60687703 | 0.609494125 | 0.612249713 | 0.615285417 | 0.618958907 | 0.622402806 | 0.625116323 | 0.628633942 | 0.634263435 | 0.641324353 | 0.649108174 | 0.657962047 | 0.667276758 | 0.676783503 | 0.686719925 | 0.69667068 | 0.70378849 | 0.708864828 |
| Venezuela | 0.516891218 | 0.522272296 | 0.529517991 | 0.536279978 | 0.541866058 | 0.546348234 | 0.548649226 | 0.552364437 | 0.557243117 | 0.560277999 | 0.56350626 | 0.565458381 | 0.562604461 | 0.555212224 | 0.550972256 | 0.553941679 | 0.56333419 | 0.575367174 | 0.586184887 | 0.593288422 | 0.599127923 | 0.604823484 | 0.610187465 | 0.614441024 | 0.614686806 | 0.610675357 | 0.606993213 | 0.605160131 | 0.604068247 | 0.602313887 | 0.600034781 | 0.596513059 |
| Tropical Latin America | 0.499587794 | 0.504290478 | 0.508056827 | 0.511622219 | 0.515330113 | 0.519290875 | 0.523498539 | 0.527841481 | 0.532164921 | 0.536658546 | 0.541715646 | 0.546931515 | 0.552364618 | 0.557691442 | 0.563268657 | 0.568897321 | 0.574720306 | 0.580915353 | 0.587353638 | 0.593303117 | 0.599646005 | 0.605956975 | 0.61178731 | 0.617401724 | 0.622646118 | 0.627399632 | 0.631533364 | 0.635765178 | 0.640179901 | 0.644693634 | 0.648602851 | 0.652442394 |
| Brazil | 0.500070509 | 0.504784798 | 0.508539943 | 0.512081793 | 0.515760842 | 0.519685963 | 0.523863329 | 0.528177889 | 0.532481169 | 0.536972035 | 0.542051497 | 0.54730443 | 0.552776035 | 0.55814571 | 0.563772907 | 0.569458118 | 0.575338775 | 0.581588755 | 0.588077818 | 0.594073297 | 0.600445846 | 0.606777291 | 0.612619337 | 0.618218237 | 0.623433814 | 0.628149803 | 0.632235114 | 0.636422188 | 0.640802974 | 0.645298005 | 0.649201568 | 0.653043887 |
| Acre | 0.372460174 | 0.378980241 | 0.384504394 | 0.38967272 | 0.395394211 | 0.401619627 | 0.407548372 | 0.412889884 | 0.418094805 | 0.423294984 | 0.429100865 | 0.4352422 | 0.441715534 | 0.44810708 | 0.454996776 | 0.462208898 | 0.469541035 | 0.477662154 | 0.486323572 | 0.494498393 | 0.502673163 | 0.510151349 | 0.517172382 | 0.523836132 | 0.530065153 | 0.535643301 | 0.540550767 | 0.545506627 | 0.550665348 | 0.55603086 | 0.560873428 | 0.565851526 |
| Alagoas | 0.375345821 | 0.38023745 | 0.384027164 | 0.386951623 | 0.389896012 | 0.392646954 | 0.395593369 | 0.399214316 | 0.403276779 | 0.407308246 | 0.411951402 | 0.416798486 | 0.422190605 | 0.427693085 | 0.433637623 | 0.439780035 | 0.446252822 | 0.453131528 | 0.460104129 | 0.466850058 | 0.474132525 | 0.481873331 | 0.488871408 | 0.495583877 | 0.501814413 | 0.507530874 | 0.512474145 | 0.517429631 | 0.522419929 | 0.527385261 | 0.531592601 | 0.535659976 |
| Amapá | 0.466489972 | 0.473046435 | 0.478145562 | 0.482182483 | 0.486829328 | 0.492740827 | 0.49860984 | 0.504140235 | 0.508931047 | 0.513368306 | 0.518241109 | 0.523566718 | 0.529168238 | 0.533943881 | 0.538676375 | 0.543766861 | 0.549921407 | 0.556730501 | 0.563796865 | 0.5703049 | 0.576616954 | 0.582639762 | 0.588829837 | 0.594780105 | 0.600371867 | 0.605321171 | 0.609514122 | 0.613827423 | 0.61842977 | 0.623291669 | 0.627651849 | 0.632179635 |
| Amazonas | 0.484414577 | 0.490171921 | 0.4941568 | 0.498171213 | 0.500865898 | 0.502034694 | 0.502823745 | 0.503017744 | 0.503369852 | 0.504117595 | 0.506062217 | 0.508355299 | 0.511612632 | 0.515295516 | 0.52014233 | 0.525587094 | 0.531836991 | 0.538247502 | 0.544623832 | 0.550489884 | 0.556768547 | 0.56268609 | 0.567585452 | 0.572357437 | 0.577020763 | 0.581398647 | 0.585372807 | 0.589701003 | 0.594525205 | 0.599677023 | 0.604527351 | 0.609484469 |
| Bahia | 0.41801382 | 0.423900821 | 0.428644816 | 0.432921354 | 0.436994935 | 0.440272212 | 0.443807533 | 0.447529476 | 0.451064095 | 0.454853367 | 0.459268839 | 0.463956524 | 0.469350032 | 0.474745912 | 0.48082891 | 0.487494934 | 0.494242014 | 0.501472687 | 0.508896753 | 0.516226812 | 0.523632281 | 0.53042636 | 0.536573214 | 0.542468778 | 0.547940905 | 0.552789085 | 0.55691537 | 0.561036385 | 0.565299911 | 0.569674237 | 0.573501951 | 0.577394253 |
| Ceará | 0.398353718 | 0.40427565 | 0.409294965 | 0.414032839 | 0.419088629 | 0.424445663 | 0.430080828 | 0.435248358 | 0.43991082 | 0.444360723 | 0.449055465 | 0.45376096 | 0.459070047 | 0.464303218 | 0.46989832 | 0.475759045 | 0.482189717 | 0.488992281 | 0.496508008 | 0.503778291 | 0.51166156 | 0.519605029 | 0.526499626 | 0.532956901 | 0.538832628 | 0.544039969 | 0.548431121 | 0.552799397 | 0.557132872 | 0.561466567 | 0.565090356 | 0.568665698 |
| Distrito Federal | 0.583730192 | 0.591082949 | 0.595897693 | 0.599876679 | 0.604819954 | 0.618006745 | 0.630290826 | 0.640613076 | 0.649632422 | 0.657487179 | 0.664416142 | 0.67097978 | 0.677195301 | 0.68278788 | 0.688186678 | 0.693478299 | 0.699106313 | 0.705230968 | 0.711861883 | 0.718281012 | 0.724844815 | 0.731209692 | 0.736925714 | 0.742308071 | 0.74721747 | 0.751591504 | 0.755405506 | 0.759379142 | 0.763558051 | 0.767908872 | 0.771908796 | 0.776016643 |
| Espírito Santo | 0.489800826 | 0.495332665 | 0.500161475 | 0.504654971 | 0.509780209 | 0.515480061 | 0.520844987 | 0.526081348 | 0.53127897 | 0.536847495 | 0.543561478 | 0.550288609 | 0.556853315 | 0.56311935 | 0.570310901 | 0.577927741 | 0.5856401 | 0.593469081 | 0.60121699 | 0.607382538 | 0.614229728 | 0.621547265 | 0.628328474 | 0.634513152 | 0.639995953 | 0.64483223 | 0.648989612 | 0.653189953 | 0.657612951 | 0.662179321 | 0.666176267 | 0.670192595 |
| Goiás | 0.453438148 | 0.460179009 | 0.465097122 | 0.46976741 | 0.474631888 | 0.479858862 | 0.48553627 | 0.491472893 | 0.497707742 | 0.504092618 | 0.511432619 | 0.519124532 | 0.52749671 | 0.535412334 | 0.543161381 | 0.550362509 | 0.557580459 | 0.565050972 | 0.572590584 | 0.579759638 | 0.586871065 | 0.593962457 | 0.600644445 | 0.606701794 | 0.612093194 | 0.61682493 | 0.620779028 | 0.624788391 | 0.628970099 | 0.63327306 | 0.637081372 | 0.640933873 |
| Maranhão | 0.313023543 | 0.319224358 | 0.324157618 | 0.328661244 | 0.333630525 | 0.339376001 | 0.345669569 | 0.351066591 | 0.355270891 | 0.359166843 | 0.363587739 | 0.368182205 | 0.373286391 | 0.37876805 | 0.384904516 | 0.391809976 | 0.399271104 | 0.407321117 | 0.416230503 | 0.424208262 | 0.432234401 | 0.440448713 | 0.448372282 | 0.455599834 | 0.462055312 | 0.467810703 | 0.472865026 | 0.477983806 | 0.483306317 | 0.488831213 | 0.493858199 | 0.49892987 |
| Mato Grosso | 0.457312691 | 0.463967763 | 0.469716305 | 0.475569609 | 0.481304068 | 0.486368479 | 0.491495218 | 0.497158911 | 0.502799965 | 0.509001634 | 0.51622992 | 0.52354486 | 0.53205088 | 0.541559903 | 0.552339658 | 0.561683675 | 0.569460229 | 0.577843826 | 0.58689801 | 0.594829914 | 0.601699538 | 0.608448329 | 0.614563955 | 0.619861601 | 0.624501429 | 0.628596012 | 0.632052445 | 0.635629733 | 0.639471425 | 0.643613875 | 0.647392236 | 0.651185759 |
| Mato Grosso do Sul | 0.480886355 | 0.485393804 | 0.488630055 | 0.491891739 | 0.495863954 | 0.49877929 | 0.502012889 | 0.505864168 | 0.510425772 | 0.515585159 | 0.521423795 | 0.527766707 | 0.534337703 | 0.541210097 | 0.547835772 | 0.55405041 | 0.560479037 | 0.567234673 | 0.574173166 | 0.580673428 | 0.587696339 | 0.594739358 | 0.601395315 | 0.607658364 | 0.613567567 | 0.618988091 | 0.623692617 | 0.628528762 | 0.633583739 | 0.63874298 | 0.643217378 | 0.64753332 |
| Minas Gerais | 0.494231041 | 0.499747029 | 0.504362287 | 0.508531841 | 0.51311003 | 0.516926011 | 0.520857254 | 0.524825805 | 0.528604659 | 0.532472401 | 0.537102304 | 0.542203722 | 0.547962454 | 0.553922981 | 0.560488499 | 0.566977213 | 0.573669485 | 0.580570115 | 0.587666502 | 0.593648509 | 0.600267821 | 0.60687262 | 0.612879494 | 0.618628612 | 0.623966091 | 0.628765462 | 0.632795279 | 0.636822817 | 0.640982571 | 0.645233209 | 0.648789042 | 0.652293253 |
| Pará | 0.433188565 | 0.439782409 | 0.443914048 | 0.449390902 | 0.454416572 | 0.456197802 | 0.457380857 | 0.458097603 | 0.458675175 | 0.45933038 | 0.460883995 | 0.463262731 | 0.466520248 | 0.470274965 | 0.475370389 | 0.481310726 | 0.488069197 | 0.495409363 | 0.503320242 | 0.510295763 | 0.518748171 | 0.527233401 | 0.534559483 | 0.541351786 | 0.547538133 | 0.553064889 | 0.557855529 | 0.562728666 | 0.567810929 | 0.573028507 | 0.577577712 | 0.582134405 |
| Paraíba | 0.396846309 | 0.403849551 | 0.408899007 | 0.413799628 | 0.419115289 | 0.423480248 | 0.427852546 | 0.43197639 | 0.435780989 | 0.439453252 | 0.443563854 | 0.448289871 | 0.453574631 | 0.458789287 | 0.463969044 | 0.4696234 | 0.476271485 | 0.483372978 | 0.490969775 | 0.498300883 | 0.505711826 | 0.513168598 | 0.520234238 | 0.526856586 | 0.532963347 | 0.538346272 | 0.54285845 | 0.54731419 | 0.551820473 | 0.55632748 | 0.56013944 | 0.563868152 |
| Paraná | 0.509650881 | 0.513544215 | 0.516665469 | 0.519940801 | 0.5240295 | 0.528442393 | 0.533466096 | 0.538918647 | 0.544828513 | 0.551061592 | 0.557641245 | 0.564300732 | 0.571253536 | 0.578486668 | 0.585560116 | 0.591918186 | 0.598160939 | 0.604943674 | 0.611516133 | 0.61731326 | 0.623180324 | 0.628980592 | 0.634399923 | 0.639693043 | 0.644801771 | 0.649565878 | 0.653757304 | 0.658086349 | 0.662669565 | 0.667321201 | 0.671182233 | 0.674856534 |
| Pernambuco | 0.425212065 | 0.431196773 | 0.435294585 | 0.438625264 | 0.441795382 | 0.444270997 | 0.447065377 | 0.450063197 | 0.453262352 | 0.456550562 | 0.460365376 | 0.464630928 | 0.469701609 | 0.474840079 | 0.480336011 | 0.486386525 | 0.49287223 | 0.499854443 | 0.507143012 | 0.514318256 | 0.522271428 | 0.530134975 | 0.537809835 | 0.545074151 | 0.55178047 | 0.557758133 | 0.562921566 | 0.568022782 | 0.573142082 | 0.578163803 | 0.582510643 | 0.586764224 |
| Piauí | 0.344034125 | 0.350490702 | 0.355144925 | 0.360301859 | 0.366061245 | 0.372743273 | 0.379283202 | 0.384930973 | 0.38973714 | 0.394110991 | 0.398821123 | 0.4033661 | 0.408392953 | 0.413639873 | 0.419282919 | 0.425408483 | 0.432252618 | 0.43962404 | 0.447807892 | 0.455965939 | 0.464469463 | 0.472853061 | 0.480327137 | 0.487294612 | 0.493626186 | 0.499226477 | 0.503922857 | 0.508511134 | 0.513092028 | 0.517640911 | 0.521475433 | 0.525243118 |
| Rio de Janeiro | 0.577165929 | 0.580732921 | 0.583569006 | 0.586183932 | 0.588730546 | 0.59157667 | 0.594989694 | 0.598729205 | 0.602983575 | 0.607692053 | 0.613005007 | 0.618353244 | 0.623698832 | 0.628445582 | 0.633567976 | 0.638593627 | 0.643632183 | 0.648696938 | 0.654129663 | 0.658903025 | 0.664069505 | 0.669592672 | 0.674979799 | 0.680218346 | 0.685146937 | 0.689753294 | 0.693938308 | 0.698419029 | 0.703211551 | 0.708117808 | 0.712233046 | 0.716093842 |
| Rio Grande do Norte | 0.41829153 | 0.423517752 | 0.426995884 | 0.43064853 | 0.434084359 | 0.437251325 | 0.441177128 | 0.445355343 | 0.449355275 | 0.453643052 | 0.458730822 | 0.464282383 | 0.470501235 | 0.476402756 | 0.482754431 | 0.48958579 | 0.497018592 | 0.50477026 | 0.512609855 | 0.520072179 | 0.527905837 | 0.535759742 | 0.543180562 | 0.550137977 | 0.55654885 | 0.562263308 | 0.567161654 | 0.572012785 | 0.576910592 | 0.581766856 | 0.585876676 | 0.589854818 |
| Rio Grande do Sul | 0.556901852 | 0.560585518 | 0.564129687 | 0.567855382 | 0.571433991 | 0.57367372 | 0.576171806 | 0.579124158 | 0.582577123 | 0.586737395 | 0.591774422 | 0.597210928 | 0.602861371 | 0.60844358 | 0.613974335 | 0.619186507 | 0.624515432 | 0.630269952 | 0.636166834 | 0.641538556 | 0.647214806 | 0.652411741 | 0.657129774 | 0.661703943 | 0.666047678 | 0.670154551 | 0.673811446 | 0.677666952 | 0.68176946 | 0.686047381 | 0.689720928 | 0.693337528 |
| Rondônia | 0.449843647 | 0.454054902 | 0.456571687 | 0.45884913 | 0.460785842 | 0.462871321 | 0.465668136 | 0.469333872 | 0.4735258 | 0.478398792 | 0.48414852 | 0.490355239 | 0.497407496 | 0.505099253 | 0.513313329 | 0.521684976 | 0.529357473 | 0.537219992 | 0.545544678 | 0.553638024 | 0.561991339 | 0.570608238 | 0.578327511 | 0.585364751 | 0.591733971 | 0.597389859 | 0.602244861 | 0.607063574 | 0.611950302 | 0.616760297 | 0.620875282 | 0.624854665 |
| Roraima | 0.47622098 | 0.481078873 | 0.483531055 | 0.483848404 | 0.483918678 | 0.489355873 | 0.495096223 | 0.500238417 | 0.505062325 | 0.510358709 | 0.516318063 | 0.522611804 | 0.528928396 | 0.534904122 | 0.540092521 | 0.545150533 | 0.550764044 | 0.556862544 | 0.563316339 | 0.569465215 | 0.575505913 | 0.581173841 | 0.58602361 | 0.590652107 | 0.59495541 | 0.598750758 | 0.601926258 | 0.605322663 | 0.609009609 | 0.613161435 | 0.617009419 | 0.621005567 |
| Santa Catarina | 0.547671627 | 0.552450808 | 0.556656648 | 0.560199304 | 0.56401257 | 0.567741304 | 0.57195993 | 0.576439366 | 0.580941199 | 0.585807915 | 0.591746435 | 0.597922213 | 0.604289267 | 0.610665478 | 0.617214841 | 0.6234999 | 0.62967057 | 0.636128908 | 0.642931181 | 0.648991456 | 0.655416994 | 0.661701053 | 0.667216351 | 0.672260907 | 0.676711863 | 0.680563382 | 0.683742632 | 0.687120541 | 0.690787866 | 0.694664641 | 0.698011774 | 0.701340701 |
| São Paulo | 0.564758448 | 0.568849951 | 0.5721516 | 0.575334285 | 0.578935322 | 0.583745835 | 0.588962422 | 0.594491028 | 0.599875602 | 0.605392837 | 0.611467261 | 0.617422241 | 0.623058688 | 0.62826422 | 0.633424686 | 0.638565992 | 0.64372872 | 0.649140918 | 0.654571401 | 0.65967818 | 0.665170021 | 0.670621058 | 0.675678991 | 0.680719106 | 0.685506191 | 0.689835142 | 0.693598484 | 0.697537018 | 0.701747137 | 0.706144563 | 0.709954062 | 0.713727597 |
| Sergipe | 0.444644766 | 0.450327693 | 0.454549905 | 0.45810966 | 0.460630267 | 0.462694986 | 0.465478785 | 0.468966292 | 0.472533866 | 0.476130944 | 0.480224884 | 0.485254713 | 0.491006148 | 0.496766683 | 0.502822552 | 0.509108638 | 0.515785914 | 0.522832655 | 0.530178275 | 0.536583092 | 0.543581929 | 0.550450651 | 0.556748828 | 0.562649609 | 0.56800955 | 0.572738355 | 0.576710478 | 0.580707395 | 0.584819425 | 0.588990825 | 0.592438632 | 0.595840177 |
| Tocantins | 0.385988031 | 0.388290427 | 0.389253254 | 0.390525148 | 0.392989216 | 0.398731675 | 0.404750399 | 0.411020436 | 0.417674795 | 0.424378506 | 0.432208134 | 0.441485903 | 0.451219715 | 0.461555838 | 0.471888203 | 0.481870045 | 0.491620765 | 0.501862965 | 0.512509138 | 0.522696004 | 0.533028979 | 0.542528971 | 0.551320706 | 0.559617279 | 0.567321275 | 0.574402592 | 0.580658462 | 0.586816475 | 0.592878645 | 0.598840601 | 0.603864483 | 0.608587676 |
| Paraguay | 0.469527785 | 0.474352286 | 0.479314169 | 0.4846142 | 0.490226935 | 0.496336876 | 0.502322804 | 0.508286948 | 0.513819896 | 0.518720583 | 0.523007793 | 0.526916763 | 0.531177358 | 0.535360126 | 0.539556655 | 0.543622697 | 0.547943305 | 0.552758676 | 0.558016903 | 0.562900059 | 0.568846562 | 0.574954463 | 0.580650186 | 0.587247042 | 0.593953153 | 0.600484125 | 0.607018145 | 0.613581591 | 0.620032362 | 0.626070139 | 0.631057689 | 0.635718099 |
| North Africa and Middle East | 0.437420668 | 0.445920934 | 0.453971432 | 0.462102156 | 0.470393819 | 0.478553399 | 0.486462858 | 0.493980049 | 0.501445054 | 0.508955784 | 0.516824215 | 0.524091271 | 0.531149449 | 0.538292126 | 0.545678163 | 0.553047386 | 0.560088959 | 0.566516099 | 0.572339255 | 0.577062164 | 0.581849041 | 0.587593075 | 0.594111701 | 0.601127066 | 0.608418687 | 0.61577742 | 0.623142325 | 0.630560773 | 0.637917915 | 0.645094792 | 0.651673493 | 0.658224715 |
| North Africa and Middle East | 0.437420668 | 0.445920934 | 0.453971432 | 0.462102156 | 0.470393819 | 0.478553399 | 0.486462858 | 0.493980049 | 0.501445054 | 0.508955784 | 0.516824215 | 0.524091271 | 0.531149449 | 0.538292126 | 0.545678163 | 0.553047386 | 0.560088959 | 0.566516099 | 0.572339255 | 0.577062164 | 0.581849041 | 0.587593075 | 0.594111701 | 0.601127066 | 0.608418687 | 0.61577742 | 0.623142325 | 0.630560773 | 0.637917915 | 0.645094792 | 0.651673493 | 0.658224715 |
| Afghanistan | 0.173832165 | 0.17647255 | 0.179633724 | 0.180183713 | 0.17851866 | 0.178279128 | 0.178085404 | 0.177884124 | 0.177513403 | 0.177075239 | 0.177025772 | 0.177773144 | 0.18380843 | 0.190518991 | 0.196903541 | 0.203928133 | 0.211074194 | 0.219627611 | 0.228089171 | 0.237900824 | 0.247759949 | 0.257041676 | 0.266484407 | 0.275636558 | 0.284030351 | 0.291849505 | 0.299630696 | 0.307424618 | 0.314866093 | 0.322454047 | 0.329830068 | 0.337199998 |
| Algeria | 0.460486906 | 0.468319197 | 0.475950937 | 0.483255591 | 0.49045066 | 0.497957543 | 0.505990967 | 0.514056566 | 0.522382419 | 0.530785583 | 0.539481508 | 0.547529277 | 0.555172222 | 0.562536956 | 0.569352852 | 0.576001965 | 0.582126362 | 0.587790538 | 0.593190973 | 0.597870384 | 0.602823535 | 0.607825529 | 0.612703533 | 0.617422204 | 0.622087292 | 0.626745568 | 0.631710736 | 0.636973176 | 0.642500039 | 0.648210785 | 0.653651466 | 0.659500924 |
| Bahrain | 0.584578852 | 0.590596897 | 0.596040229 | 0.602165622 | 0.608133725 | 0.613850722 | 0.619485376 | 0.624596062 | 0.630738301 | 0.638083187 | 0.646750697 | 0.657196064 | 0.664901201 | 0.669564111 | 0.674147775 | 0.679790598 | 0.686144289 | 0.693045147 | 0.699806387 | 0.70488584 | 0.70790262 | 0.708958739 | 0.710362418 | 0.713343156 | 0.71676806 | 0.719926605 | 0.723611297 | 0.729323103 | 0.736192583 | 0.742847804 | 0.748103083 | 0.753043204 |
| Egypt | 0.417182742 | 0.426909452 | 0.437744555 | 0.448490053 | 0.458727004 | 0.468035368 | 0.476116627 | 0.482970886 | 0.489184071 | 0.495502619 | 0.502169886 | 0.508716824 | 0.515038129 | 0.520623395 | 0.525181678 | 0.528665483 | 0.530395807 | 0.529457594 | 0.524743833 | 0.516255831 | 0.507715477 | 0.504594044 | 0.509157068 | 0.519334464 | 0.531749809 | 0.544370149 | 0.556012469 | 0.566914632 | 0.577442786 | 0.587736877 | 0.597363341 | 0.606787094 |
| Iran | 0.453799944 | 0.468982858 | 0.480626697 | 0.492277025 | 0.505072857 | 0.517531494 | 0.52860072 | 0.537727779 | 0.546366017 | 0.555624332 | 0.565474561 | 0.574618208 | 0.584097268 | 0.593963012 | 0.60371745 | 0.613291056 | 0.621268095 | 0.627749442 | 0.63286223 | 0.63739838 | 0.64255917 | 0.647899089 | 0.651500587 | 0.654475289 | 0.657961511 | 0.662057987 | 0.667652445 | 0.674343462 | 0.680909621 | 0.686741662 | 0.691918763 | 0.697207398 |
| Alborz | 0.540408689 | 0.552446156 | 0.561720763 | 0.571360408 | 0.581991853 | 0.59235619 | 0.601919501 | 0.609791771 | 0.617160272 | 0.624754093 | 0.63279463 | 0.640064758 | 0.647546833 | 0.655274695 | 0.662959535 | 0.670555438 | 0.677039604 | 0.682555902 | 0.687020349 | 0.691040895 | 0.695817661 | 0.700902919 | 0.704542428 | 0.707988134 | 0.71207689 | 0.716481385 | 0.722142121 | 0.728497177 | 0.734379343 | 0.73937839 | 0.743762409 | 0.748302022 |
| Ardebil | 0.360726369 | 0.378799381 | 0.393000243 | 0.407753646 | 0.424543993 | 0.44117041 | 0.456014466 | 0.468500206 | 0.480594273 | 0.493810004 | 0.50770823 | 0.520560483 | 0.533452481 | 0.546545391 | 0.559148283 | 0.571222808 | 0.58096928 | 0.588520211 | 0.594464419 | 0.599814955 | 0.605856848 | 0.612092341 | 0.616375479 | 0.619667216 | 0.623139813 | 0.6268815 | 0.631789147 | 0.637801588 | 0.643829926 | 0.649168531 | 0.653816149 | 0.658639771 |
| Bushehr | 0.453839596 | 0.469448794 | 0.481662416 | 0.494246564 | 0.508013522 | 0.521385121 | 0.533890659 | 0.544378403 | 0.554069826 | 0.563930667 | 0.573964145 | 0.583082045 | 0.592445924 | 0.60232832 | 0.612040963 | 0.621738228 | 0.630023043 | 0.636807532 | 0.642114309 | 0.646975549 | 0.65255811 | 0.658444789 | 0.66239921 | 0.665559681 | 0.669147721 | 0.673299087 | 0.678900105 | 0.685684069 | 0.692350583 | 0.698211212 | 0.703304964 | 0.708418708 |
| Chahar Mahaal and Bakhtiari | 0.396177428 | 0.413752843 | 0.428041937 | 0.442933803 | 0.459408764 | 0.475501709 | 0.490244611 | 0.502691384 | 0.514474966 | 0.526414669 | 0.538544026 | 0.54949036 | 0.56030347 | 0.571376993 | 0.582217906 | 0.592701572 | 0.601371534 | 0.608318443 | 0.613551282 | 0.618091485 | 0.623195908 | 0.628496791 | 0.631727243 | 0.634141644 | 0.637146084 | 0.64097387 | 0.646628847 | 0.65353781 | 0.660480406 | 0.66674984 | 0.672349773 | 0.677988602 |
| East Azarbayejan | 0.41107167 | 0.428565789 | 0.441380504 | 0.453432793 | 0.466432141 | 0.478877163 | 0.489608139 | 0.498394773 | 0.506906517 | 0.516334524 | 0.526435732 | 0.535823226 | 0.545560479 | 0.555559386 | 0.565326935 | 0.574707869 | 0.582294986 | 0.588221742 | 0.592814792 | 0.597027427 | 0.602057203 | 0.607513653 | 0.611661035 | 0.615442168 | 0.620064998 | 0.625672138 | 0.632887567 | 0.640999871 | 0.648819451 | 0.655768514 | 0.661975804 | 0.668193235 |
| Fars | 0.458096531 | 0.473693688 | 0.486439073 | 0.499443128 | 0.51335231 | 0.526754556 | 0.538837966 | 0.549504903 | 0.559797448 | 0.57048609 | 0.581159139 | 0.591183716 | 0.601538834 | 0.61180187 | 0.621556379 | 0.63081941 | 0.638337049 | 0.64498069 | 0.650531858 | 0.655628184 | 0.661525839 | 0.667797321 | 0.67239882 | 0.676130732 | 0.680080352 | 0.684258144 | 0.689571597 | 0.69564725 | 0.701342142 | 0.706207553 | 0.710401859 | 0.714830769 |
| Gilan | 0.487126643 | 0.499460221 | 0.508700148 | 0.518056868 | 0.528092897 | 0.537867504 | 0.546866678 | 0.554629344 | 0.562175507 | 0.570205493 | 0.57887202 | 0.587013808 | 0.595698026 | 0.604814863 | 0.613961669 | 0.623148438 | 0.631527248 | 0.639269506 | 0.645857556 | 0.651804829 | 0.658139631 | 0.664390404 | 0.668830511 | 0.672679447 | 0.676853501 | 0.681242533 | 0.686866253 | 0.693085832 | 0.698784576 | 0.70363634 | 0.707883433 | 0.712319447 |
| Golestan | 0.407352738 | 0.426926755 | 0.441059963 | 0.454286526 | 0.468554203 | 0.482081553 | 0.49346702 | 0.502532992 | 0.511246358 | 0.520758265 | 0.531086784 | 0.5406348 | 0.550581631 | 0.560925957 | 0.571041927 | 0.581107409 | 0.589334073 | 0.595193786 | 0.599366762 | 0.602933696 | 0.607292195 | 0.611911224 | 0.614494068 | 0.616054685 | 0.618108638 | 0.621009681 | 0.625746836 | 0.632122313 | 0.638853184 | 0.645013911 | 0.650487092 | 0.65607753 |
| Hamadan | 0.393037826 | 0.410220293 | 0.423912725 | 0.437800504 | 0.452938893 | 0.467590975 | 0.480586561 | 0.491692757 | 0.502193297 | 0.513311656 | 0.524906451 | 0.535760896 | 0.546976369 | 0.558722447 | 0.570205424 | 0.58137325 | 0.590865982 | 0.598860268 | 0.605328071 | 0.611107018 | 0.617135019 | 0.623008217 | 0.626750369 | 0.629480691 | 0.632537916 | 0.635932162 | 0.640695849 | 0.646572512 | 0.652372298 | 0.657467578 | 0.661972521 | 0.66673482 |
| Hormozgan | 0.380909867 | 0.398548745 | 0.412525116 | 0.426860879 | 0.442821254 | 0.458529988 | 0.472603368 | 0.484619542 | 0.496242767 | 0.508746293 | 0.521718852 | 0.533482965 | 0.545168268 | 0.557252401 | 0.569519927 | 0.581455712 | 0.591114587 | 0.598234696 | 0.603255498 | 0.607449377 | 0.612072002 | 0.617148232 | 0.620470717 | 0.623050764 | 0.626495557 | 0.630925408 | 0.636918229 | 0.64425644 | 0.651674532 | 0.658411304 | 0.664390432 | 0.670331366 |
| Ilam | 0.409636699 | 0.428514542 | 0.443906192 | 0.459715963 | 0.47669329 | 0.492956965 | 0.507819049 | 0.520575019 | 0.532693066 | 0.545783519 | 0.559538748 | 0.57259365 | 0.585946309 | 0.599608449 | 0.612447932 | 0.624442405 | 0.634923782 | 0.644462336 | 0.652692593 | 0.660030476 | 0.667144489 | 0.673561109 | 0.677405569 | 0.680045454 | 0.682455493 | 0.68473158 | 0.688370891 | 0.69282091 | 0.696898332 | 0.700228521 | 0.703020491 | 0.706194927 |
| Isfahan | 0.487959498 | 0.502499113 | 0.513759556 | 0.524654244 | 0.535936687 | 0.546644571 | 0.556328697 | 0.564527037 | 0.572314491 | 0.580505822 | 0.589088253 | 0.597045736 | 0.605324133 | 0.613951214 | 0.622565995 | 0.631246421 | 0.639093619 | 0.646236139 | 0.652270174 | 0.65777781 | 0.663591479 | 0.669300343 | 0.673190662 | 0.676409261 | 0.679781336 | 0.683158957 | 0.687673268 | 0.692950589 | 0.697868561 | 0.702063752 | 0.705722709 | 0.709716845 |
| Kerman | 0.430231243 | 0.446669591 | 0.459613095 | 0.472939055 | 0.487557248 | 0.50177046 | 0.514550658 | 0.525228903 | 0.535080841 | 0.545405976 | 0.556175637 | 0.565883133 | 0.575695645 | 0.58614764 | 0.596045883 | 0.605090087 | 0.612002284 | 0.617018609 | 0.620325096 | 0.622933114 | 0.626139353 | 0.629681435 | 0.631486259 | 0.632636758 | 0.634410204 | 0.637013521 | 0.641263427 | 0.646987137 | 0.652934496 | 0.658361876 | 0.663270783 | 0.668439265 |
| Kermanshah | 0.404063329 | 0.418944664 | 0.430677775 | 0.442969461 | 0.456671649 | 0.470165926 | 0.482910245 | 0.493319538 | 0.502877113 | 0.512764657 | 0.523326823 | 0.533367722 | 0.544062928 | 0.555647676 | 0.567435635 | 0.579067899 | 0.589104707 | 0.597787183 | 0.604985015 | 0.611589765 | 0.618651635 | 0.625715595 | 0.630735229 | 0.634754569 | 0.638771657 | 0.642881242 | 0.648033942 | 0.654128778 | 0.660017739 | 0.665160192 | 0.669708021 | 0.674466468 |
| Khorasan-e-Razavi | 0.40318232 | 0.419388051 | 0.431379703 | 0.443165339 | 0.456264538 | 0.468991438 | 0.48000785 | 0.488685804 | 0.496687726 | 0.505420726 | 0.514907288 | 0.523878599 | 0.533653405 | 0.544388784 | 0.55547648 | 0.566813376 | 0.576436317 | 0.584380607 | 0.590867255 | 0.596602396 | 0.603023738 | 0.6095685 | 0.614032303 | 0.617746053 | 0.62207644 | 0.627156616 | 0.633776223 | 0.641679638 | 0.64959811 | 0.656817763 | 0.663343373 | 0.669826641 |
| Khuzestan | 0.411717104 | 0.430329418 | 0.444092399 | 0.457051262 | 0.471014342 | 0.484259839 | 0.495041354 | 0.503511676 | 0.511585072 | 0.520772892 | 0.531041338 | 0.540748004 | 0.55094375 | 0.561382942 | 0.571691782 | 0.581795716 | 0.589688446 | 0.595413844 | 0.599791133 | 0.603787655 | 0.608651158 | 0.61396507 | 0.61764073 | 0.620711198 | 0.624423762 | 0.629059013 | 0.635242471 | 0.642720547 | 0.650295432 | 0.657199966 | 0.663397214 | 0.669587913 |
| Kohgiluyeh and Boyer-Ahmad | 0.385444625 | 0.403033901 | 0.416642534 | 0.430717139 | 0.446547889 | 0.462229473 | 0.47612742 | 0.487763929 | 0.499036207 | 0.511072379 | 0.523556607 | 0.535354398 | 0.547768828 | 0.560861447 | 0.57385919 | 0.586421747 | 0.596609496 | 0.60489609 | 0.611499333 | 0.617650129 | 0.624879003 | 0.632514816 | 0.638427284 | 0.643810545 | 0.649591195 | 0.655601248 | 0.662620079 | 0.6703172 | 0.677470867 | 0.68356697 | 0.688652582 | 0.693706198 |
| Kurdistan | 0.34632778 | 0.362447943 | 0.375440307 | 0.38862839 | 0.402963883 | 0.416962406 | 0.429685212 | 0.440474448 | 0.450905653 | 0.46191712 | 0.473710492 | 0.485225188 | 0.497641784 | 0.511078438 | 0.524531126 | 0.537640087 | 0.548860233 | 0.55845825 | 0.56649055 | 0.573753385 | 0.581259509 | 0.588656686 | 0.594073737 | 0.59857926 | 0.603242424 | 0.608024922 | 0.613911649 | 0.62056984 | 0.626901502 | 0.632413609 | 0.637233456 | 0.642261692 |
| Lorestan | 0.372692648 | 0.391835021 | 0.407133613 | 0.422519576 | 0.439322885 | 0.455649233 | 0.470104029 | 0.482000472 | 0.493415816 | 0.505665767 | 0.518797545 | 0.531429387 | 0.544435174 | 0.557669334 | 0.570510339 | 0.582662233 | 0.592589967 | 0.600400752 | 0.606609687 | 0.612092008 | 0.617759459 | 0.623300885 | 0.62683319 | 0.62950842 | 0.63256128 | 0.63628853 | 0.641458362 | 0.647709275 | 0.653853614 | 0.659311001 | 0.664168997 | 0.669118289 |
| Markazi | 0.431126436 | 0.446318425 | 0.458666642 | 0.471251666 | 0.484631548 | 0.497579881 | 0.509690596 | 0.520238798 | 0.530425095 | 0.540916614 | 0.551656231 | 0.561587752 | 0.57175965 | 0.582312322 | 0.592633987 | 0.602439068 | 0.610795172 | 0.617928649 | 0.623620054 | 0.628725743 | 0.634217553 | 0.639747712 | 0.643436832 | 0.646367117 | 0.649588677 | 0.653156021 | 0.658091198 | 0.663915873 | 0.669411257 | 0.674157696 | 0.678377418 | 0.682843822 |
| Mazandaran | 0.504227331 | 0.520307133 | 0.532162754 | 0.543172259 | 0.554438776 | 0.564985363 | 0.574153477 | 0.581401075 | 0.587722361 | 0.594308132 | 0.601369105 | 0.608181092 | 0.616157031 | 0.625652293 | 0.635722964 | 0.646077235 | 0.655337595 | 0.663549855 | 0.67026376 | 0.676079888 | 0.68194593 | 0.68754549 | 0.691223198 | 0.694160519 | 0.697312762 | 0.700781638 | 0.70577577 | 0.71153246 | 0.716916247 | 0.721534916 | 0.72560047 | 0.729929903 |
| North Khorasan | 0.347842053 | 0.365863242 | 0.37902068 | 0.391915395 | 0.406447733 | 0.420753657 | 0.43325316 | 0.4435523 | 0.453444139 | 0.464441351 | 0.476464732 | 0.488082146 | 0.500632789 | 0.514152099 | 0.527958593 | 0.541772267 | 0.553620127 | 0.563255393 | 0.571154979 | 0.57803034 | 0.585353781 | 0.592676291 | 0.597729444 | 0.601772416 | 0.606067715 | 0.610917515 | 0.617242588 | 0.624807838 | 0.632405836 | 0.639311578 | 0.645493107 | 0.651610197 |
| Qazvin | 0.413374606 | 0.42875875 | 0.439910721 | 0.450246346 | 0.461099117 | 0.471356931 | 0.480631073 | 0.488819062 | 0.497443183 | 0.507680985 | 0.5195944 | 0.532077147 | 0.546032566 | 0.561014732 | 0.57585077 | 0.590221127 | 0.602566383 | 0.612841772 | 0.620939509 | 0.627783424 | 0.63474628 | 0.641343445 | 0.645786375 | 0.649177433 | 0.652590764 | 0.656265287 | 0.661261999 | 0.6672753 | 0.673168654 | 0.67833935 | 0.682903203 | 0.6876536 |
| Qom | 0.452628907 | 0.465722037 | 0.475802913 | 0.486425484 | 0.498440084 | 0.510319504 | 0.521096027 | 0.530026839 | 0.53828674 | 0.546971243 | 0.5560084 | 0.56442097 | 0.573136998 | 0.582229697 | 0.591429627 | 0.600851085 | 0.60906901 | 0.615595252 | 0.620914343 | 0.625966552 | 0.631937562 | 0.638316767 | 0.643106982 | 0.64734331 | 0.652097703 | 0.657005641 | 0.663229424 | 0.670397892 | 0.677291514 | 0.683353803 | 0.68869247 | 0.694075907 |
| Semnan | 0.496089451 | 0.505579479 | 0.512703331 | 0.520769634 | 0.530215318 | 0.539840308 | 0.549412651 | 0.557994613 | 0.566637746 | 0.575727814 | 0.585268167 | 0.594267071 | 0.603598115 | 0.613394843 | 0.6230342 | 0.632501223 | 0.640801728 | 0.648054605 | 0.654184954 | 0.659857295 | 0.666145385 | 0.672690353 | 0.677498021 | 0.68199998 | 0.686875855 | 0.691776775 | 0.697728396 | 0.70432963 | 0.710302636 | 0.715287837 | 0.719537284 | 0.723926385 |
| Sistan and Baluchistan | 0.308840734 | 0.326078692 | 0.338899321 | 0.351732463 | 0.366584876 | 0.381150014 | 0.393055912 | 0.401954761 | 0.410016208 | 0.419505526 | 0.4298579 | 0.438746889 | 0.447405678 | 0.456287808 | 0.465329905 | 0.474545646 | 0.480598769 | 0.482918404 | 0.483206592 | 0.483056918 | 0.484565321 | 0.487224362 | 0.488292343 | 0.488781317 | 0.490694759 | 0.495003133 | 0.502038435 | 0.51167765 | 0.52247451 | 0.532827625 | 0.54220697 | 0.551305977 |
| South Khorasan | 0.350446131 | 0.367440589 | 0.38062956 | 0.394002213 | 0.408852797 | 0.423488664 | 0.436553451 | 0.447312873 | 0.457270791 | 0.46779298 | 0.479008713 | 0.489629529 | 0.501065299 | 0.513428831 | 0.526061232 | 0.538834604 | 0.54973374 | 0.558797735 | 0.56611706 | 0.572613665 | 0.579819087 | 0.58729861 | 0.592793223 | 0.597619891 | 0.603048947 | 0.608988306 | 0.616346471 | 0.624767909 | 0.632970669 | 0.64027843 | 0.64672361 | 0.653125383 |
| Tehran | 0.587612685 | 0.597405602 | 0.605000433 | 0.613073986 | 0.622138665 | 0.631118522 | 0.639506955 | 0.646425935 | 0.652979878 | 0.659879899 | 0.667358544 | 0.674300593 | 0.681493625 | 0.689017548 | 0.696495394 | 0.703899522 | 0.710327304 | 0.715895129 | 0.720418636 | 0.724430139 | 0.728984653 | 0.733628395 | 0.736904652 | 0.73985661 | 0.743224805 | 0.746904882 | 0.751863904 | 0.757620713 | 0.763004575 | 0.767649705 | 0.771743291 | 0.776032454 |
| West Azarbayejan | 0.37997815 | 0.394902017 | 0.406170759 | 0.417408542 | 0.429901147 | 0.442058721 | 0.452581184 | 0.460986464 | 0.469157741 | 0.47852181 | 0.488786111 | 0.498362554 | 0.50842601 | 0.519123347 | 0.530007962 | 0.540873083 | 0.549728232 | 0.556244352 | 0.560902989 | 0.564837957 | 0.569474614 | 0.57442046 | 0.57758425 | 0.580098128 | 0.583251678 | 0.587339769 | 0.59304459 | 0.600206352 | 0.607620436 | 0.614484871 | 0.620732482 | 0.627112463 |
| Yazd | 0.454227587 | 0.468809771 | 0.479870146 | 0.491575953 | 0.505026011 | 0.518510439 | 0.530704079 | 0.540959313 | 0.550735949 | 0.561282626 | 0.572302283 | 0.582162398 | 0.592025049 | 0.601983517 | 0.611807591 | 0.621710339 | 0.630176569 | 0.637190268 | 0.642666088 | 0.647579726 | 0.653190986 | 0.659124931 | 0.663136315 | 0.666468064 | 0.670281658 | 0.674857755 | 0.681070046 | 0.688462043 | 0.695673304 | 0.70205677 | 0.707697596 | 0.713344284 |
| Zanjan | 0.391316094 | 0.4088901 | 0.420937843 | 0.432099645 | 0.444346897 | 0.456107387 | 0.465850585 | 0.473262332 | 0.47968483 | 0.486947981 | 0.495135641 | 0.503431287 | 0.513485073 | 0.525359755 | 0.538357345 | 0.552207764 | 0.564924509 | 0.575775942 | 0.584344188 | 0.591526484 | 0.598891826 | 0.606247989 | 0.61159146 | 0.616034488 | 0.620636349 | 0.625474775 | 0.631612155 | 0.638661586 | 0.645468803 | 0.651408578 | 0.656539511 | 0.661729132 |
| Iraq | 0.412044173 | 0.415814116 | 0.420353594 | 0.424834933 | 0.428751731 | 0.432075545 | 0.436818479 | 0.443104027 | 0.452384092 | 0.462736441 | 0.472376399 | 0.48145832 | 0.488664737 | 0.492174445 | 0.498933881 | 0.504719431 | 0.510918049 | 0.517698972 | 0.525242908 | 0.533200914 | 0.542343278 | 0.553048555 | 0.564745467 | 0.576968998 | 0.588317652 | 0.599494007 | 0.611508646 | 0.622674532 | 0.633232108 | 0.643635318 | 0.65330038 | 0.662626231 |
| Jordan | 0.539147468 | 0.542819411 | 0.547458846 | 0.55218235 | 0.557254335 | 0.562571536 | 0.567529655 | 0.572581508 | 0.57764357 | 0.582759199 | 0.587909394 | 0.593182803 | 0.599000385 | 0.60494595 | 0.611934404 | 0.619909228 | 0.627803813 | 0.635670276 | 0.643892693 | 0.652196319 | 0.659949495 | 0.66739668 | 0.674568925 | 0.681119962 | 0.68713404 | 0.692952032 | 0.698473302 | 0.703923826 | 0.709412879 | 0.714998946 | 0.72006824 | 0.725307227 |
| Kuwait | 0.664517904 | 0.668809621 | 0.67015911 | 0.670036252 | 0.671393213 | 0.677215523 | 0.686093304 | 0.695789389 | 0.704383736 | 0.711841328 | 0.718357562 | 0.722840631 | 0.726816976 | 0.731778685 | 0.738153707 | 0.746384913 | 0.754928493 | 0.762411484 | 0.770002615 | 0.777546551 | 0.784873929 | 0.791812065 | 0.798356988 | 0.804583834 | 0.810743233 | 0.816998413 | 0.823026527 | 0.828886987 | 0.834371461 | 0.839688307 | 0.843459812 | 0.846651055 |
| Lebanon | 0.536718969 | 0.539197492 | 0.541793516 | 0.544581306 | 0.547684033 | 0.55147172 | 0.556436159 | 0.562120818 | 0.56905907 | 0.576078528 | 0.582698277 | 0.589185502 | 0.595978788 | 0.60296724 | 0.61084576 | 0.619391355 | 0.627791217 | 0.636938234 | 0.646667663 | 0.657433186 | 0.668804525 | 0.680920617 | 0.693910983 | 0.704811001 | 0.712423873 | 0.718697292 | 0.724583347 | 0.729963294 | 0.734746243 | 0.738644009 | 0.74200972 | 0.744746351 |
| Libya | 0.527998168 | 0.541432399 | 0.554468227 | 0.566798415 | 0.578651416 | 0.589578634 | 0.600350044 | 0.610620315 | 0.619973984 | 0.628600604 | 0.637047935 | 0.644685131 | 0.651669147 | 0.659345125 | 0.666913926 | 0.675322268 | 0.683702 | 0.691855134 | 0.698873253 | 0.704998471 | 0.711362496 | 0.711466657 | 0.716700693 | 0.71766525 | 0.716042207 | 0.713651367 | 0.71076014 | 0.710666264 | 0.712720438 | 0.716157239 | 0.720270393 | 0.725771399 |
| Morocco | 0.35807287 | 0.364984629 | 0.371187194 | 0.376778977 | 0.382849451 | 0.38816411 | 0.394150096 | 0.399504009 | 0.405038792 | 0.410303191 | 0.415344026 | 0.420595133 | 0.42577575 | 0.43121781 | 0.436822352 | 0.442531339 | 0.44867616 | 0.455036325 | 0.461803552 | 0.468929893 | 0.476269799 | 0.483922152 | 0.491603392 | 0.499528607 | 0.507443265 | 0.515582893 | 0.523518592 | 0.531610862 | 0.539729671 | 0.547837444 | 0.555246069 | 0.562698301 |
| Oman | 0.429270949 | 0.442268122 | 0.457737972 | 0.474999033 | 0.493513107 | 0.513194359 | 0.533246394 | 0.554315537 | 0.574474902 | 0.59239847 | 0.608421541 | 0.622160627 | 0.635162126 | 0.647249093 | 0.657626118 | 0.666401231 | 0.675353885 | 0.685055583 | 0.694952263 | 0.703098571 | 0.710688695 | 0.718264528 | 0.726450074 | 0.73343249 | 0.738743772 | 0.743597182 | 0.748797193 | 0.753786058 | 0.759179514 | 0.764527863 | 0.768854216 | 0.773391602 |
| Palestine | 0.40179221 | 0.40572715 | 0.411146869 | 0.416779649 | 0.423136673 | 0.429307027 | 0.434731371 | 0.440901776 | 0.447782415 | 0.454909692 | 0.460681496 | 0.465299102 | 0.468600257 | 0.472881732 | 0.478527322 | 0.485320586 | 0.491890407 | 0.498978426 | 0.506849479 | 0.515815836 | 0.525711905 | 0.53677763 | 0.548295425 | 0.559163481 | 0.569239882 | 0.579204261 | 0.58949277 | 0.599157187 | 0.608124861 | 0.616466839 | 0.623810015 | 0.631011665 |
| Qatar | 0.651208376 | 0.655338767 | 0.660673763 | 0.666194399 | 0.672025384 | 0.678320339 | 0.684835919 | 0.693109654 | 0.701409064 | 0.708880981 | 0.714831609 | 0.720831064 | 0.727112174 | 0.733536599 | 0.740223208 | 0.747189359 | 0.754123028 | 0.760960134 | 0.767659233 | 0.774276849 | 0.780807074 | 0.78721245 | 0.793494901 | 0.799611555 | 0.80572571 | 0.811872852 | 0.817979841 | 0.824016868 | 0.829962127 | 0.835805284 | 0.841467395 | 0.846860584 |
| Saudi Arabia | 0.538954515 | 0.549907717 | 0.561144266 | 0.572186913 | 0.582878871 | 0.593251423 | 0.603648418 | 0.613749116 | 0.623518214 | 0.632796447 | 0.642311774 | 0.651502092 | 0.660356856 | 0.669839577 | 0.679917856 | 0.690520852 | 0.701152613 | 0.711442141 | 0.721960205 | 0.731158488 | 0.740300669 | 0.749959451 | 0.759054935 | 0.767387864 | 0.775178133 | 0.782300802 | 0.788867882 | 0.794886288 | 0.800656985 | 0.806087841 | 0.810541861 | 0.815143493 |
| Sudan | 0.292178643 | 0.296473446 | 0.300863677 | 0.305409326 | 0.31009643 | 0.315208259 | 0.320680229 | 0.326761462 | 0.333087761 | 0.339754884 | 0.346875809 | 0.354215515 | 0.361728237 | 0.369693653 | 0.377920656 | 0.386685712 | 0.396151374 | 0.406079992 | 0.4160876 | 0.425975917 | 0.436171097 | 0.447239442 | 0.456790265 | 0.466435995 | 0.476362135 | 0.486549437 | 0.496848219 | 0.506883534 | 0.516214086 | 0.525009122 | 0.533455422 | 0.541949735 |
| Syria | 0.430492643 | 0.437907493 | 0.445904236 | 0.45414786 | 0.462437298 | 0.470692043 | 0.478743085 | 0.48623235 | 0.493707992 | 0.500474869 | 0.507143686 | 0.513850111 | 0.521309744 | 0.528935756 | 0.538084469 | 0.549750449 | 0.560484761 | 0.57002812 | 0.578990493 | 0.587789118 | 0.595967764 | 0.601524456 | 0.602872601 | 0.602410134 | 0.60223576 | 0.602282914 | 0.602911181 | 0.604896366 | 0.608507164 | 0.613336836 | 0.617727593 | 0.623004075 |
| Tunisia | 0.471138521 | 0.479543057 | 0.488285281 | 0.496852039 | 0.505564882 | 0.51419694 | 0.523294274 | 0.532064044 | 0.540498161 | 0.548724563 | 0.55667265 | 0.56442101 | 0.571719534 | 0.578882669 | 0.586032594 | 0.592937759 | 0.599758513 | 0.606696784 | 0.613529322 | 0.620106833 | 0.626432774 | 0.63201984 | 0.637578345 | 0.643008512 | 0.648297395 | 0.653413538 | 0.658353517 | 0.663222845 | 0.668218997 | 0.673170552 | 0.677615148 | 0.682432216 |
| Türkiye | 0.461606984 | 0.469244066 | 0.476814569 | 0.48465697 | 0.49161995 | 0.49882484 | 0.506238546 | 0.513933356 | 0.521509898 | 0.528512416 | 0.535866331 | 0.54235913 | 0.549023644 | 0.556102527 | 0.564024 | 0.572495886 | 0.581383179 | 0.590442387 | 0.599212061 | 0.607104618 | 0.615597228 | 0.624787211 | 0.634097092 | 0.643723823 | 0.653388835 | 0.662971739 | 0.671948148 | 0.681096272 | 0.689895867 | 0.698263774 | 0.705799744 | 0.712692673 |
| United Arab Emirates | 0.644412271 | 0.660739899 | 0.675926211 | 0.689463801 | 0.701656792 | 0.713138288 | 0.724181976 | 0.734723175 | 0.744476623 | 0.753253916 | 0.760969094 | 0.767858793 | 0.774346424 | 0.781143992 | 0.788525951 | 0.796428086 | 0.805537023 | 0.814870878 | 0.822793435 | 0.828304165 | 0.831284254 | 0.832912804 | 0.83358611 | 0.833803818 | 0.833858727 | 0.834175498 | 0.83522071 | 0.837213553 | 0.83990014 | 0.842985326 | 0.846151976 | 0.849317734 |
| Yemen | 0.215664586 | 0.222885409 | 0.230415046 | 0.238025246 | 0.245879426 | 0.253962572 | 0.262278613 | 0.270782536 | 0.279500507 | 0.288261301 | 0.297145157 | 0.306112424 | 0.315065023 | 0.324081689 | 0.333371285 | 0.343462562 | 0.353472947 | 0.363301218 | 0.373140288 | 0.382762252 | 0.392936641 | 0.401471801 | 0.409561349 | 0.417642405 | 0.425189667 | 0.430197789 | 0.434232983 | 0.437549155 | 0.440727991 | 0.4440014 | 0.446893069 | 0.450376375 |
| South Asia | 0.319796517 | 0.325854134 | 0.331940961 | 0.338013384 | 0.344322901 | 0.350726453 | 0.357286569 | 0.363719215 | 0.370287576 | 0.376979322 | 0.383388457 | 0.389533341 | 0.395194069 | 0.400983424 | 0.407180554 | 0.413986658 | 0.421367361 | 0.429261848 | 0.437011859 | 0.445197355 | 0.454075792 | 0.463339273 | 0.473080558 | 0.483434269 | 0.494084362 | 0.504891461 | 0.515310997 | 0.525128437 | 0.534477408 | 0.543245696 | 0.550585566 | 0.557864657 |
| South Asia | 0.319796517 | 0.325854134 | 0.331940961 | 0.338013384 | 0.344322901 | 0.350726453 | 0.357286569 | 0.363719215 | 0.370287576 | 0.376979322 | 0.383388457 | 0.389533341 | 0.395194069 | 0.400983424 | 0.407180554 | 0.413986658 | 0.421367361 | 0.429261848 | 0.437011859 | 0.445197355 | 0.454075792 | 0.463339273 | 0.473080558 | 0.483434269 | 0.494084362 | 0.504891461 | 0.515310997 | 0.525128437 | 0.534477408 | 0.543245696 | 0.550585566 | 0.557864657 |
| Bangladesh | 0.228548934 | 0.237207413 | 0.245247956 | 0.25209072 | 0.258997294 | 0.265293918 | 0.271177781 | 0.27777328 | 0.28451594 | 0.290951431 | 0.297251976 | 0.303188546 | 0.308640093 | 0.31436004 | 0.320922851 | 0.32809051 | 0.335690598 | 0.343932546 | 0.352520033 | 0.361481513 | 0.370598956 | 0.380124886 | 0.390623552 | 0.401706478 | 0.413263169 | 0.425482121 | 0.437709906 | 0.449752413 | 0.461717749 | 0.473264998 | 0.483079169 | 0.492420885 |
| Bhutan | 0.21503985 | 0.22131244 | 0.228148272 | 0.236004687 | 0.244331246 | 0.253291957 | 0.262362746 | 0.271487422 | 0.280440384 | 0.289510488 | 0.298804875 | 0.308200652 | 0.317943536 | 0.327670422 | 0.337286495 | 0.346998578 | 0.356824209 | 0.367560498 | 0.378233543 | 0.388977162 | 0.399751936 | 0.410149249 | 0.419443216 | 0.427790675 | 0.43496927 | 0.441810748 | 0.448401855 | 0.454280497 | 0.459624711 | 0.464633353 | 0.468714479 | 0.473062378 |
| India | 0.332593603 | 0.338591779 | 0.344591727 | 0.350685397 | 0.35699603 | 0.363418501 | 0.370018644 | 0.376358763 | 0.382817848 | 0.389453889 | 0.395752331 | 0.401837472 | 0.407411414 | 0.413115783 | 0.419184961 | 0.425913062 | 0.433321558 | 0.44135146 | 0.449235851 | 0.457672332 | 0.467018996 | 0.476832909 | 0.487210876 | 0.498317981 | 0.5097221 | 0.521242719 | 0.532220023 | 0.542388507 | 0.551930326 | 0.560809927 | 0.568138149 | 0.575401649 |
| Nepal | 0.199560654 | 0.205687443 | 0.21211309 | 0.218843431 | 0.22607306 | 0.233253154 | 0.240700616 | 0.248382797 | 0.256227179 | 0.26435918 | 0.272719115 | 0.281287467 | 0.289513608 | 0.297772595 | 0.30619653 | 0.314661796 | 0.323056346 | 0.33144561 | 0.340150706 | 0.348701893 | 0.3568681 | 0.364642605 | 0.372437588 | 0.380022326 | 0.387689088 | 0.395021154 | 0.401628776 | 0.408463874 | 0.415236684 | 0.422007252 | 0.427417089 | 0.433174635 |
| Pakistan | 0.310467621 | 0.316265667 | 0.322248051 | 0.328219252 | 0.334433128 | 0.340926296 | 0.347656203 | 0.354218627 | 0.360875336 | 0.367641397 | 0.374319132 | 0.380593927 | 0.386629992 | 0.392719263 | 0.399084167 | 0.405667437 | 0.41223483 | 0.418519756 | 0.4243482 | 0.430019833 | 0.435640657 | 0.441455585 | 0.447096599 | 0.452823095 | 0.458781424 | 0.464792668 | 0.471022168 | 0.477544137 | 0.484326128 | 0.491022981 | 0.497364406 | 0.504028689 |
| Azad Jammu & Kashmir | 0.325857101 | 0.333077208 | 0.340429071 | 0.347704205 | 0.355188695 | 0.36290103 | 0.370811847 | 0.378509273 | 0.386226886 | 0.39394301 | 0.401498379 | 0.408603265 | 0.415455566 | 0.422355367 | 0.429582007 | 0.437046441 | 0.444661193 | 0.452318423 | 0.459789715 | 0.467147638 | 0.474261641 | 0.481325642 | 0.487924526 | 0.494234619 | 0.500536166 | 0.506652081 | 0.512701361 | 0.518794973 | 0.524990042 | 0.530935981 | 0.53639063 | 0.54211877 |
| Balochistan | 0.248830294 | 0.253867226 | 0.259137459 | 0.264445518 | 0.270032258 | 0.275808956 | 0.281657954 | 0.28728496 | 0.292936773 | 0.298615917 | 0.304123501 | 0.309363286 | 0.314486793 | 0.319767541 | 0.325408179 | 0.331358275 | 0.337312959 | 0.342947156 | 0.348160279 | 0.353256825 | 0.358392437 | 0.363767123 | 0.368950276 | 0.374140892 | 0.379493292 | 0.384691831 | 0.389889988 | 0.395343926 | 0.401056798 | 0.406750451 | 0.412161247 | 0.417971505 |
| Gilgit-Baltistan | 0.222103674 | 0.227640973 | 0.233261906 | 0.238770234 | 0.244414316 | 0.250163095 | 0.256324108 | 0.262331705 | 0.268435004 | 0.274619029 | 0.2806372 | 0.286449667 | 0.292099121 | 0.297895335 | 0.304072308 | 0.310637869 | 0.317451071 | 0.324220863 | 0.330744423 | 0.337148884 | 0.343332309 | 0.349566741 | 0.355264683 | 0.360580297 | 0.365620515 | 0.370252676 | 0.374865913 | 0.379497987 | 0.38426318 | 0.38884361 | 0.393019782 | 0.397549987 |
| Islamabad Capital Territory | 0.487150872 | 0.494111387 | 0.501070598 | 0.50780845 | 0.514676352 | 0.521761529 | 0.529113389 | 0.536212659 | 0.543302326 | 0.550405879 | 0.557424714 | 0.564086066 | 0.570521696 | 0.577016131 | 0.583826388 | 0.591056051 | 0.598529198 | 0.606138542 | 0.613597881 | 0.620954942 | 0.628112811 | 0.635273606 | 0.642007775 | 0.648431244 | 0.654795368 | 0.660935334 | 0.667044898 | 0.673161092 | 0.679344849 | 0.68524364 | 0.690594808 | 0.696195374 |
| Khyber Pakhtunkhwa | 0.267901742 | 0.272521311 | 0.277278922 | 0.282095856 | 0.287208184 | 0.292664619 | 0.298363471 | 0.303939494 | 0.309610949 | 0.315386855 | 0.321016482 | 0.326206782 | 0.331154606 | 0.336238999 | 0.341759438 | 0.347726209 | 0.353999838 | 0.360512546 | 0.366993854 | 0.373538072 | 0.379999399 | 0.386566235 | 0.392838756 | 0.398991867 | 0.405288659 | 0.411493023 | 0.417721039 | 0.424078678 | 0.430705536 | 0.437348572 | 0.44373658 | 0.450581146 |
| Punjab | 0.316049943 | 0.322229572 | 0.328631987 | 0.335042017 | 0.341726759 | 0.348740259 | 0.356039491 | 0.363112926 | 0.370279528 | 0.377573896 | 0.384802757 | 0.391614429 | 0.398166428 | 0.404727993 | 0.411529175 | 0.418536232 | 0.425547556 | 0.432290326 | 0.438536327 | 0.444551067 | 0.450444633 | 0.456469687 | 0.46227533 | 0.468185607 | 0.474276293 | 0.480432883 | 0.486844769 | 0.493559385 | 0.500478325 | 0.507240922 | 0.513584121 | 0.520200488 |
| Sindh | 0.327061045 | 0.332559514 | 0.338170748 | 0.343712065 | 0.349423746 | 0.355314165 | 0.361355173 | 0.367341984 | 0.373442645 | 0.3796483 | 0.385785028 | 0.39154363 | 0.397097704 | 0.402757134 | 0.408706022 | 0.41484554 | 0.420845354 | 0.426343572 | 0.431269767 | 0.436063685 | 0.440975115 | 0.44624365 | 0.451541902 | 0.457063379 | 0.46304727 | 0.469235197 | 0.475786409 | 0.482793609 | 0.490243177 | 0.497751522 | 0.50498481 | 0.512574957 |
| Southeast Asia, east Asia, and Oceania | 0.471036942 | 0.479318189 | 0.487345622 | 0.49528092 | 0.502971282 | 0.511167456 | 0.520513218 | 0.529602631 | 0.53725808 | 0.54471189 | 0.551286754 | 0.556468474 | 0.562479429 | 0.569543561 | 0.577065736 | 0.58501175 | 0.593528725 | 0.602226266 | 0.610408078 | 0.618390416 | 0.628143532 | 0.636360524 | 0.641725375 | 0.647351187 | 0.652750402 | 0.656819032 | 0.661605599 | 0.668919875 | 0.676603325 | 0.684704677 | 0.69221505 | 0.698849901 |
| East Asia | 0.471179162 | 0.479935584 | 0.488431396 | 0.496859473 | 0.504885881 | 0.513530275 | 0.5237577 | 0.533815513 | 0.542652548 | 0.551367028 | 0.558815666 | 0.564234748 | 0.570798377 | 0.578904729 | 0.587682668 | 0.597096172 | 0.607273415 | 0.617523836 | 0.626955795 | 0.636271151 | 0.64798501 | 0.65750207 | 0.662929873 | 0.668681911 | 0.67406275 | 0.677306858 | 0.681528332 | 0.689747572 | 0.698625829 | 0.708327813 | 0.717709308 | 0.725704902 |
| China | 0.458668935 | 0.46730197 | 0.475784031 | 0.484353831 | 0.492578645 | 0.501500908 | 0.512033996 | 0.522326866 | 0.531379484 | 0.540333688 | 0.547989193 | 0.553680374 | 0.560542936 | 0.569018827 | 0.578178091 | 0.588052194 | 0.59875817 | 0.609520246 | 0.619454833 | 0.629307727 | 0.641521443 | 0.651447978 | 0.657144292 | 0.663103007 | 0.668640847 | 0.671940111 | 0.676218437 | 0.684619661 | 0.693717996 | 0.703686824 | 0.713364585 | 0.72162976 |
| North Korea | 0.497780128 | 0.499120512 | 0.499629841 | 0.499700646 | 0.49953134 | 0.499080529 | 0.498429014 | 0.497136869 | 0.49615921 | 0.496484754 | 0.497461995 | 0.499674043 | 0.502633569 | 0.506165243 | 0.510229134 | 0.515097763 | 0.51974144 | 0.524066965 | 0.528813498 | 0.533114411 | 0.537192918 | 0.54121227 | 0.545331713 | 0.549483297 | 0.55360356 | 0.557321271 | 0.560743563 | 0.563388289 | 0.565161484 | 0.566765706 | 0.568188551 | 0.569854634 |
| Taiwan (province of China) | 0.667633854 | 0.676537237 | 0.684803636 | 0.69292413 | 0.70107875 | 0.709626821 | 0.719128415 | 0.729145497 | 0.738125787 | 0.745338399 | 0.752926841 | 0.760829809 | 0.769008984 | 0.776955803 | 0.784677978 | 0.792445129 | 0.800174675 | 0.808137694 | 0.815400691 | 0.821432279 | 0.827226752 | 0.832064074 | 0.836677665 | 0.841280463 | 0.84569556 | 0.850055269 | 0.854466038 | 0.858888019 | 0.863158361 | 0.86741185 | 0.871247947 | 0.874747053 |
| Oceania | 0.391195082 | 0.394418822 | 0.397572264 | 0.401113099 | 0.404892361 | 0.408507251 | 0.412437065 | 0.415893127 | 0.419224844 | 0.422520527 | 0.425069659 | 0.427004026 | 0.42853491 | 0.43005967 | 0.431581333 | 0.433151845 | 0.434584099 | 0.436101755 | 0.43739244 | 0.438807984 | 0.440751869 | 0.442617769 | 0.444524399 | 0.446593514 | 0.449363524 | 0.452397261 | 0.455453805 | 0.458377792 | 0.460818103 | 0.463333934 | 0.465534643 | 0.467445126 |
| American Samoa | 0.613633924 | 0.615847612 | 0.618583654 | 0.622078598 | 0.626053241 | 0.629814054 | 0.632706983 | 0.634926685 | 0.636597976 | 0.638322193 | 0.64060955 | 0.643214421 | 0.646002062 | 0.648951964 | 0.651921141 | 0.655061005 | 0.657725723 | 0.660781176 | 0.664025762 | 0.667300532 | 0.671248916 | 0.675979288 | 0.680861539 | 0.685833061 | 0.691343137 | 0.697162912 | 0.702726398 | 0.707647909 | 0.712800591 | 0.717308133 | 0.7208385 | 0.723727533 |
| Cook Islands | 0.564514854 | 0.570622776 | 0.577123468 | 0.584059175 | 0.591540868 | 0.598789968 | 0.606414151 | 0.613991564 | 0.621202228 | 0.62802903 | 0.635365607 | 0.644421442 | 0.652790488 | 0.661465038 | 0.668769675 | 0.674004103 | 0.680644001 | 0.687505191 | 0.695592069 | 0.70377698 | 0.711861612 | 0.719444666 | 0.726963385 | 0.733317015 | 0.739595704 | 0.746550297 | 0.752353087 | 0.758233572 | 0.764041182 | 0.769795913 | 0.774700578 | 0.779109955 |
| Fiji | 0.534648908 | 0.53877826 | 0.543188552 | 0.547776474 | 0.55284902 | 0.558530972 | 0.565041879 | 0.571187753 | 0.577172354 | 0.583573942 | 0.589196631 | 0.594497419 | 0.599322031 | 0.603489622 | 0.60754489 | 0.610812944 | 0.613932767 | 0.616582915 | 0.618958809 | 0.620883465 | 0.623308786 | 0.626438251 | 0.629881335 | 0.634278114 | 0.639390066 | 0.645026353 | 0.65074268 | 0.65646401 | 0.661991454 | 0.667060979 | 0.671431491 | 0.675051631 |
| Guam | 0.676220305 | 0.669974292 | 0.66635634 | 0.667318181 | 0.672111712 | 0.678120215 | 0.684173735 | 0.691684871 | 0.701819289 | 0.713641593 | 0.724802357 | 0.734267376 | 0.741284245 | 0.74591153 | 0.74901652 | 0.750865875 | 0.751176245 | 0.752625471 | 0.755152408 | 0.758252296 | 0.761484317 | 0.763987394 | 0.766845984 | 0.770224957 | 0.773441684 | 0.776723614 | 0.780511644 | 0.78555492 | 0.791294773 | 0.796775452 | 0.800969472 | 0.803982203 |
| Kiribati | 0.410389821 | 0.412884199 | 0.415577609 | 0.418481661 | 0.421716217 | 0.425024354 | 0.428300682 | 0.431641941 | 0.435741904 | 0.439692923 | 0.444436339 | 0.448782301 | 0.453462298 | 0.458208374 | 0.462336213 | 0.466722276 | 0.470557197 | 0.47422745 | 0.477201933 | 0.480035601 | 0.482447689 | 0.485028211 | 0.488175864 | 0.491880767 | 0.495317241 | 0.500059699 | 0.505207837 | 0.510019241 | 0.514810384 | 0.519441321 | 0.523454113 | 0.527186583 |
| Marshall Islands | 0.430839288 | 0.435571412 | 0.440491018 | 0.445479084 | 0.450848221 | 0.456859674 | 0.46120943 | 0.464752728 | 0.468133032 | 0.471292727 | 0.474562536 | 0.478588316 | 0.482776259 | 0.48624862 | 0.489573077 | 0.493271391 | 0.497456175 | 0.502250042 | 0.506359588 | 0.510794536 | 0.516085138 | 0.521177087 | 0.526042019 | 0.5314463 | 0.536640131 | 0.541793167 | 0.546782936 | 0.552065152 | 0.557599278 | 0.56342875 | 0.568837509 | 0.574091128 |
| Federated States of Micronesia | 0.462511617 | 0.468144831 | 0.473792936 | 0.479939595 | 0.485300006 | 0.491015185 | 0.495570308 | 0.499110106 | 0.502861564 | 0.506663431 | 0.511004228 | 0.515316814 | 0.519545672 | 0.523829599 | 0.527610965 | 0.531703321 | 0.535718682 | 0.539431464 | 0.542766354 | 0.546275496 | 0.550122546 | 0.554240411 | 0.557965853 | 0.561082821 | 0.56384003 | 0.567151342 | 0.570458101 | 0.574039833 | 0.577555081 | 0.581195255 | 0.584527229 | 0.587534967 |
| Nauru | 0.539145981 | 0.538433557 | 0.537038893 | 0.535059742 | 0.533001097 | 0.530890925 | 0.528544474 | 0.52624159 | 0.523885302 | 0.521894324 | 0.520254888 | 0.519235425 | 0.518438705 | 0.518459986 | 0.51880954 | 0.519892863 | 0.522673179 | 0.52320135 | 0.526084096 | 0.530221092 | 0.535689995 | 0.542342743 | 0.549890621 | 0.560112906 | 0.571424364 | 0.581283234 | 0.590102988 | 0.597619076 | 0.605311879 | 0.612697253 | 0.619330076 | 0.625177834 |
| Niue | 0.587532984 | 0.593882057 | 0.599881243 | 0.605514779 | 0.610650175 | 0.615374609 | 0.619734555 | 0.623913848 | 0.627578934 | 0.630857172 | 0.63406895 | 0.637366627 | 0.641122021 | 0.64577954 | 0.650614832 | 0.657281265 | 0.664162983 | 0.670199299 | 0.676396859 | 0.682579173 | 0.68628063 | 0.690315578 | 0.694150949 | 0.697221078 | 0.700545924 | 0.704232979 | 0.707476099 | 0.711220996 | 0.71510823 | 0.719078885 | 0.72279025 | 0.72622205 |
| Northern Mariana Islands | 0.708593838 | 0.712223642 | 0.715787607 | 0.719179396 | 0.722510494 | 0.726219053 | 0.730074086 | 0.733795366 | 0.737423127 | 0.740934815 | 0.744492865 | 0.747170606 | 0.748755189 | 0.750171533 | 0.751528841 | 0.751826692 | 0.751797809 | 0.751608416 | 0.750807192 | 0.748597173 | 0.746617262 | 0.744411908 | 0.742937351 | 0.74228436 | 0.742605449 | 0.743797398 | 0.748455466 | 0.756495479 | 0.7612554 | 0.765770647 | 0.76942766 | 0.771535213 |
| Palau | 0.66290951 | 0.668230731 | 0.673302462 | 0.676648715 | 0.680195775 | 0.68483214 | 0.690095673 | 0.695122123 | 0.69965291 | 0.703115322 | 0.705196945 | 0.707402386 | 0.709587644 | 0.71100022 | 0.712543811 | 0.714339831 | 0.716353431 | 0.718809542 | 0.720806623 | 0.722288267 | 0.724065956 | 0.726733446 | 0.729612899 | 0.731953475 | 0.734768297 | 0.738166425 | 0.741361911 | 0.744224767 | 0.74776125 | 0.750715307 | 0.752780929 | 0.754046931 |
| Papua New Guinea | 0.310668629 | 0.314967305 | 0.319530259 | 0.325005984 | 0.330664468 | 0.335421285 | 0.340348698 | 0.344258325 | 0.347801408 | 0.351250621 | 0.354135791 | 0.356588986 | 0.358572086 | 0.360736816 | 0.362827542 | 0.365294965 | 0.367826759 | 0.370794147 | 0.373426209 | 0.376528285 | 0.380286778 | 0.383587539 | 0.386781028 | 0.389926437 | 0.394004571 | 0.398285864 | 0.402407267 | 0.406153435 | 0.409165083 | 0.412311147 | 0.415186879 | 0.417797443 |
| Samoa | 0.487491428 | 0.490987146 | 0.494027448 | 0.497079264 | 0.499484836 | 0.502369735 | 0.505778026 | 0.509096382 | 0.512367566 | 0.51540905 | 0.518902327 | 0.522971626 | 0.52724267 | 0.531603736 | 0.536003276 | 0.540557908 | 0.544812068 | 0.549110752 | 0.553164596 | 0.556474015 | 0.559622905 | 0.562891574 | 0.565320388 | 0.567448536 | 0.569493661 | 0.571998983 | 0.575402051 | 0.578925435 | 0.582320661 | 0.586256695 | 0.590075296 | 0.593392769 |
| Solomon Islands | 0.301217167 | 0.305830668 | 0.311431697 | 0.317151092 | 0.323300247 | 0.329968564 | 0.336208053 | 0.341835627 | 0.34713506 | 0.351867191 | 0.354799143 | 0.356626745 | 0.357910116 | 0.359424359 | 0.361290558 | 0.363580019 | 0.366443698 | 0.369827951 | 0.373764884 | 0.377821028 | 0.38271759 | 0.388055337 | 0.39300464 | 0.39790625 | 0.402244745 | 0.406233897 | 0.410406962 | 0.414676394 | 0.418889221 | 0.422736386 | 0.426112429 | 0.429360316 |
| Tokelau | 0.521942386 | 0.526628473 | 0.53130785 | 0.535935005 | 0.540523665 | 0.545295634 | 0.550282652 | 0.555631326 | 0.561047611 | 0.566415406 | 0.57181437 | 0.576915597 | 0.581858317 | 0.586854346 | 0.592342442 | 0.598176117 | 0.60442939 | 0.611163237 | 0.617940247 | 0.624070078 | 0.63017554 | 0.636016518 | 0.641611991 | 0.647966148 | 0.653231999 | 0.658798995 | 0.663974319 | 0.669054134 | 0.674179514 | 0.679247839 | 0.682937378 | 0.686425621 |
| Tonga | 0.49180684 | 0.498166205 | 0.503659848 | 0.50890445 | 0.514207835 | 0.52021502 | 0.525808135 | 0.531009092 | 0.536172314 | 0.54131702 | 0.546303195 | 0.551054251 | 0.556008345 | 0.560736066 | 0.564806379 | 0.568449169 | 0.571619868 | 0.57435151 | 0.577305415 | 0.57917075 | 0.580657299 | 0.582656723 | 0.58477088 | 0.587351141 | 0.590826853 | 0.594965748 | 0.600343669 | 0.606243592 | 0.611953942 | 0.617324063 | 0.622104456 | 0.626349936 |
| Tuvalu | 0.406247566 | 0.414819937 | 0.42290198 | 0.43051826 | 0.438563051 | 0.444962242 | 0.44980498 | 0.455368428 | 0.462454678 | 0.468845802 | 0.474956823 | 0.480850886 | 0.487252252 | 0.492754711 | 0.497692152 | 0.50185131 | 0.505975411 | 0.510504301 | 0.515611601 | 0.519870566 | 0.52345551 | 0.527744245 | 0.531203235 | 0.535166235 | 0.539122244 | 0.54404788 | 0.549173928 | 0.55446648 | 0.559859395 | 0.566045489 | 0.571666102 | 0.576620529 |
| Vanuatu | 0.353100252 | 0.357005379 | 0.360893296 | 0.364444155 | 0.368501002 | 0.372479126 | 0.376460885 | 0.380667171 | 0.384690196 | 0.388300526 | 0.392158349 | 0.395325055 | 0.397723782 | 0.400396456 | 0.403226097 | 0.406488572 | 0.410501729 | 0.414915578 | 0.419853237 | 0.42485465 | 0.429658049 | 0.434283044 | 0.438661998 | 0.442720176 | 0.446687298 | 0.450329861 | 0.454072763 | 0.458016834 | 0.462080221 | 0.466276162 | 0.470038391 | 0.473100706 |
| Southeast Asia | 0.46410364 | 0.471749871 | 0.479490406 | 0.487349418 | 0.495348107 | 0.50335114 | 0.511232528 | 0.518664155 | 0.52443938 | 0.529667781 | 0.534566608 | 0.539010433 | 0.543478548 | 0.548051006 | 0.552707418 | 0.557391298 | 0.562302171 | 0.567654259 | 0.573208389 | 0.578497394 | 0.58426847 | 0.590414618 | 0.596780286 | 0.60314492 | 0.609438681 | 0.615689065 | 0.621839858 | 0.627978129 | 0.634095876 | 0.640106755 | 0.645069663 | 0.649777295 |
| Cambodia | 0.289075059 | 0.2924792 | 0.296900955 | 0.301627202 | 0.305722592 | 0.310678881 | 0.31563097 | 0.32050375 | 0.324974299 | 0.330224246 | 0.33607503 | 0.342507911 | 0.349465712 | 0.35692966 | 0.364854645 | 0.373331815 | 0.381698166 | 0.38987623 | 0.39767952 | 0.404220933 | 0.410211121 | 0.415964409 | 0.421769754 | 0.42771506 | 0.433597749 | 0.439487358 | 0.445358172 | 0.451221744 | 0.45734149 | 0.46364343 | 0.468876054 | 0.473621491 |
| Indonesia | 0.457134954 | 0.466505583 | 0.475753157 | 0.484775241 | 0.4936071 | 0.502008381 | 0.510108146 | 0.518102189 | 0.524045604 | 0.52917633 | 0.533789587 | 0.537977981 | 0.542245676 | 0.546550264 | 0.550902314 | 0.555424913 | 0.560265924 | 0.565811896 | 0.572225267 | 0.578866902 | 0.585982419 | 0.593652985 | 0.601185701 | 0.608509904 | 0.615499887 | 0.622222469 | 0.628666625 | 0.634895745 | 0.64094875 | 0.646865457 | 0.651926648 | 0.656868336 |
| Aceh | 0.482673083 | 0.493504261 | 0.503725238 | 0.513293532 | 0.522194442 | 0.530244852 | 0.537785887 | 0.545269986 | 0.550976463 | 0.555867589 | 0.560267942 | 0.564383878 | 0.568721888 | 0.573074571 | 0.577722521 | 0.582303878 | 0.586726023 | 0.59150689 | 0.596743272 | 0.601813052 | 0.607059527 | 0.612682724 | 0.618546527 | 0.624548728 | 0.630505255 | 0.636482357 | 0.642394996 | 0.648250805 | 0.654058774 | 0.659865184 | 0.66475876 | 0.669551641 |
| Bali | 0.458655011 | 0.468848044 | 0.478844524 | 0.488700458 | 0.498232752 | 0.507060762 | 0.515231093 | 0.522993626 | 0.528355586 | 0.532622854 | 0.536164258 | 0.539277406 | 0.542573467 | 0.545939228 | 0.549365862 | 0.553032786 | 0.557032914 | 0.561905018 | 0.567800695 | 0.574177239 | 0.581140545 | 0.588570325 | 0.59582696 | 0.60287149 | 0.609659423 | 0.616201839 | 0.622454071 | 0.628472747 | 0.63430388 | 0.640005392 | 0.644852346 | 0.649585885 |
| Bangka-Belitung Islands | 0.452646767 | 0.462591923 | 0.472391479 | 0.481888282 | 0.491048594 | 0.499481814 | 0.507367902 | 0.514946326 | 0.520543825 | 0.525260596 | 0.528921139 | 0.532123819 | 0.53552897 | 0.539175226 | 0.542987035 | 0.546989555 | 0.551276501 | 0.556316987 | 0.562254321 | 0.568355782 | 0.574789695 | 0.581786707 | 0.588683375 | 0.595378152 | 0.601870208 | 0.60823522 | 0.614608509 | 0.621025153 | 0.627381446 | 0.633595593 | 0.638989 | 0.64425964 |
| Banten | 0.450965928 | 0.460784951 | 0.470637488 | 0.48025382 | 0.489594952 | 0.498356376 | 0.506821488 | 0.514934415 | 0.520591505 | 0.525083292 | 0.52859153 | 0.53158023 | 0.534635837 | 0.537753833 | 0.540951156 | 0.544360877 | 0.548187184 | 0.552791142 | 0.558376243 | 0.56437318 | 0.571021141 | 0.578572494 | 0.5859379 | 0.593109118 | 0.599988388 | 0.606625673 | 0.613036651 | 0.619180886 | 0.625147682 | 0.630960517 | 0.635878658 | 0.640712993 |
| Bengkulu | 0.410618592 | 0.421338809 | 0.43188088 | 0.441945224 | 0.451774972 | 0.4609698 | 0.46969509 | 0.478202963 | 0.484290714 | 0.489268721 | 0.493368589 | 0.496847756 | 0.500313994 | 0.503917881 | 0.507677926 | 0.511763825 | 0.51629332 | 0.521736767 | 0.528172614 | 0.534797477 | 0.541818624 | 0.549403637 | 0.556812782 | 0.564090489 | 0.57103184 | 0.577776701 | 0.584380496 | 0.590869074 | 0.597218496 | 0.603419362 | 0.608631084 | 0.613670654 |
| Gorontalo | 0.389888128 | 0.397140957 | 0.404527932 | 0.411806004 | 0.418943452 | 0.425658298 | 0.43220747 | 0.438480857 | 0.44209189 | 0.44455879 | 0.446024711 | 0.447168916 | 0.448762757 | 0.450801824 | 0.453291354 | 0.456420275 | 0.460402502 | 0.465663645 | 0.4724715 | 0.480080093 | 0.488507483 | 0.49763464 | 0.506564417 | 0.515190525 | 0.52338898 | 0.531194961 | 0.538596681 | 0.545691902 | 0.552508683 | 0.559108057 | 0.564639122 | 0.569990208 |
| Jakarta | 0.603337382 | 0.612305583 | 0.621480363 | 0.631010736 | 0.64050786 | 0.649534732 | 0.658244406 | 0.666732384 | 0.672972623 | 0.678467268 | 0.683577422 | 0.688364188 | 0.693230495 | 0.69805222 | 0.702773706 | 0.707515873 | 0.712428578 | 0.717862971 | 0.723957335 | 0.730238909 | 0.736892211 | 0.743932459 | 0.750709752 | 0.757177289 | 0.763344509 | 0.769257219 | 0.774920346 | 0.780349837 | 0.785585062 | 0.790697155 | 0.795089511 | 0.799409443 |
| Jambi | 0.423986255 | 0.434086314 | 0.443835819 | 0.453051079 | 0.461985935 | 0.470551523 | 0.478941705 | 0.487365789 | 0.493585119 | 0.498877558 | 0.503415662 | 0.507584273 | 0.511946148 | 0.516477523 | 0.521143625 | 0.525892201 | 0.531117186 | 0.537460095 | 0.545114894 | 0.553175633 | 0.561921318 | 0.571293817 | 0.580318251 | 0.58885735 | 0.596842594 | 0.604270829 | 0.611179632 | 0.617639247 | 0.623782029 | 0.629717612 | 0.634702306 | 0.639563676 |
| West Java | 0.453362331 | 0.462900417 | 0.472227416 | 0.481260302 | 0.490077931 | 0.498420828 | 0.50635713 | 0.51402432 | 0.519463845 | 0.523829587 | 0.527555116 | 0.530723862 | 0.533932301 | 0.537219636 | 0.54068547 | 0.544558242 | 0.548987834 | 0.55426903 | 0.560516379 | 0.567007559 | 0.573938972 | 0.581443237 | 0.588783562 | 0.595974101 | 0.602831345 | 0.609430098 | 0.615769647 | 0.621953187 | 0.62798418 | 0.633863396 | 0.638841358 | 0.643676633 |
| Central Java | 0.418426869 | 0.427270317 | 0.436058992 | 0.444525024 | 0.452776675 | 0.460755364 | 0.468489217 | 0.476047616 | 0.481393227 | 0.48599492 | 0.490037615 | 0.493750549 | 0.497699221 | 0.501905207 | 0.506366319 | 0.511191984 | 0.516470279 | 0.522424185 | 0.529136583 | 0.535970759 | 0.543189535 | 0.550815899 | 0.558248862 | 0.565494778 | 0.572389663 | 0.579031466 | 0.585406722 | 0.591572699 | 0.59758761 | 0.603490246 | 0.608508812 | 0.613388815 |
| East Java | 0.439345376 | 0.448002535 | 0.456568647 | 0.464981405 | 0.473362709 | 0.481554707 | 0.489625168 | 0.497734151 | 0.503899208 | 0.509461652 | 0.514829367 | 0.519944223 | 0.525155446 | 0.530392571 | 0.535633676 | 0.541009654 | 0.546614886 | 0.552806956 | 0.559715811 | 0.566762495 | 0.574268569 | 0.582269108 | 0.590083125 | 0.597565834 | 0.604651661 | 0.611407922 | 0.617839862 | 0.624019292 | 0.629982621 | 0.635786767 | 0.640729871 | 0.645517381 |
| West Kalimantan | 0.392420939 | 0.403546934 | 0.414559711 | 0.425181012 | 0.435596831 | 0.445592807 | 0.455135464 | 0.464363134 | 0.471289924 | 0.476975088 | 0.481438069 | 0.484997385 | 0.488390089 | 0.491657658 | 0.494746457 | 0.497557396 | 0.500436298 | 0.50407497 | 0.508614669 | 0.513617603 | 0.5193251 | 0.525779882 | 0.532523628 | 0.539333417 | 0.545941462 | 0.552374557 | 0.558607119 | 0.564666542 | 0.570581784 | 0.576362347 | 0.581289351 | 0.586121672 |
| South Kalimantan | 0.4410948 | 0.450659333 | 0.460186271 | 0.469587689 | 0.478715406 | 0.487260019 | 0.495360632 | 0.50319087 | 0.50876176 | 0.513397467 | 0.517247588 | 0.520499496 | 0.523801673 | 0.527093162 | 0.530268675 | 0.533399137 | 0.536695497 | 0.540652203 | 0.545450823 | 0.550620237 | 0.556422071 | 0.562998037 | 0.569668107 | 0.576335177 | 0.582879302 | 0.589249781 | 0.595471527 | 0.601564795 | 0.607513836 | 0.613341893 | 0.618310461 | 0.623206076 |
| Central Kalimantan | 0.458816473 | 0.468694807 | 0.478852254 | 0.489080158 | 0.499159777 | 0.508785407 | 0.517859927 | 0.526160727 | 0.531642782 | 0.535617829 | 0.538384563 | 0.540532219 | 0.542941203 | 0.545460202 | 0.547914568 | 0.550400235 | 0.553095629 | 0.556392354 | 0.560776212 | 0.565695267 | 0.571374211 | 0.577880614 | 0.584530859 | 0.591174468 | 0.597656029 | 0.603957534 | 0.610191418 | 0.616400368 | 0.622556258 | 0.628645715 | 0.633866616 | 0.638993825 |
| East Kalimantan | 0.570464049 | 0.579709067 | 0.588155672 | 0.59575493 | 0.603144549 | 0.610012928 | 0.61667543 | 0.623547959 | 0.629222386 | 0.634267668 | 0.638796471 | 0.64279042 | 0.646854687 | 0.6511169 | 0.655559049 | 0.660203656 | 0.665086446 | 0.67062866 | 0.677116861 | 0.683659061 | 0.690666493 | 0.698431801 | 0.706072311 | 0.713361384 | 0.720201576 | 0.726721012 | 0.732873955 | 0.738721137 | 0.744368522 | 0.749891063 | 0.754639486 | 0.759293869 |
| North Kalimantan | 0.555308193 | 0.563444647 | 0.571335245 | 0.578925818 | 0.586396151 | 0.593391674 | 0.60026322 | 0.607352228 | 0.613417455 | 0.619052162 | 0.624296974 | 0.6292073 | 0.63436734 | 0.639742224 | 0.645318572 | 0.65115801 | 0.657171624 | 0.663551607 | 0.670508983 | 0.677146303 | 0.683919382 | 0.691147925 | 0.698260962 | 0.705172954 | 0.711888182 | 0.71842697 | 0.724745797 | 0.730898414 | 0.736873529 | 0.742719223 | 0.747902022 | 0.752986755 |
| Riau Islands | 0.573195374 | 0.582604149 | 0.591698026 | 0.600398522 | 0.608909874 | 0.617005694 | 0.624868761 | 0.632527177 | 0.638224392 | 0.642955195 | 0.646758994 | 0.649916625 | 0.652990359 | 0.656061059 | 0.659111762 | 0.662319842 | 0.665911595 | 0.670292199 | 0.675560302 | 0.681127419 | 0.68712948 | 0.693589474 | 0.699993682 | 0.706231529 | 0.712199405 | 0.717942409 | 0.723485175 | 0.728857416 | 0.734073809 | 0.739154856 | 0.743469598 | 0.747754306 |
| Lampung | 0.391631433 | 0.401893655 | 0.412229167 | 0.422372896 | 0.432420699 | 0.442266267 | 0.45188518 | 0.461299065 | 0.468325456 | 0.474384663 | 0.479505141 | 0.483898697 | 0.48810632 | 0.492172449 | 0.496203848 | 0.500417272 | 0.505176715 | 0.510993727 | 0.518020191 | 0.525646434 | 0.534074159 | 0.542893715 | 0.551370299 | 0.559396713 | 0.566887109 | 0.573970777 | 0.580641694 | 0.586999082 | 0.593081112 | 0.598978995 | 0.603973383 | 0.608871357 |
| Maluku | 0.476402589 | 0.484988378 | 0.493485215 | 0.50141601 | 0.50877279 | 0.515041977 | 0.520424846 | 0.524928074 | 0.526587821 | 0.525959227 | 0.522988336 | 0.518526528 | 0.513491541 | 0.508006739 | 0.502214099 | 0.496700392 | 0.492511332 | 0.490833455 | 0.492291326 | 0.495953643 | 0.50170832 | 0.509140163 | 0.517045024 | 0.525091957 | 0.532944399 | 0.540679188 | 0.548132911 | 0.55534118 | 0.562338841 | 0.569186954 | 0.574825335 | 0.580321875 |
| North Maluku | 0.372965609 | 0.382162104 | 0.391831112 | 0.40169747 | 0.411834386 | 0.421759355 | 0.431455905 | 0.44083814 | 0.447156939 | 0.45223301 | 0.455989588 | 0.45832082 | 0.459890657 | 0.460912621 | 0.461634893 | 0.462388373 | 0.463584322 | 0.466058753 | 0.470469244 | 0.476164915 | 0.483337478 | 0.491765113 | 0.500263614 | 0.508629476 | 0.516688937 | 0.524421004 | 0.531806695 | 0.538888869 | 0.545704098 | 0.552291419 | 0.557641557 | 0.562910873 |
| West Nusa Tenggara | 0.337279768 | 0.348596437 | 0.360281417 | 0.3721195 | 0.383887897 | 0.395203377 | 0.406173953 | 0.416987393 | 0.425463443 | 0.433193111 | 0.440705527 | 0.447987762 | 0.455251195 | 0.462408148 | 0.469621993 | 0.476757439 | 0.483833614 | 0.491206004 | 0.498897461 | 0.506536972 | 0.514042282 | 0.521937265 | 0.529664329 | 0.537174668 | 0.544414464 | 0.551393396 | 0.558092789 | 0.564539028 | 0.570784472 | 0.576865896 | 0.582015196 | 0.587034621 |
| East Nusa Tenggara | 0.365588037 | 0.373390941 | 0.38136121 | 0.389191357 | 0.396974847 | 0.404505209 | 0.411801645 | 0.418800405 | 0.422702411 | 0.425734701 | 0.428451047 | 0.430954666 | 0.433816501 | 0.436965311 | 0.440294155 | 0.443832431 | 0.447765376 | 0.452535105 | 0.458477217 | 0.464895776 | 0.472099608 | 0.480086447 | 0.488014716 | 0.495788638 | 0.50327052 | 0.510536672 | 0.517612166 | 0.524616147 | 0.531497889 | 0.538245358 | 0.543913254 | 0.54945119 |
| Papua | 0.462138557 | 0.469889521 | 0.477720022 | 0.485722916 | 0.493730909 | 0.501559539 | 0.509246938 | 0.516897106 | 0.523062066 | 0.528222317 | 0.532522125 | 0.536098956 | 0.539284826 | 0.542057298 | 0.544442022 | 0.54693318 | 0.549585134 | 0.553069545 | 0.557665657 | 0.563130143 | 0.56958178 | 0.577055745 | 0.584654967 | 0.592196418 | 0.599546241 | 0.60674908 | 0.613769891 | 0.620686883 | 0.627472265 | 0.634120608 | 0.640012638 | 0.645802883 |
| West Papua | 0.457306637 | 0.466233925 | 0.475295762 | 0.484221157 | 0.492996739 | 0.501069789 | 0.508700871 | 0.516041171 | 0.521316627 | 0.525568147 | 0.528854185 | 0.531531734 | 0.534331325 | 0.537432633 | 0.540799122 | 0.544722823 | 0.549525408 | 0.55578751 | 0.563940756 | 0.57329506 | 0.584217781 | 0.596487163 | 0.607721069 | 0.618038433 | 0.627409894 | 0.635997196 | 0.643877305 | 0.651159328 | 0.657959065 | 0.664400303 | 0.669829389 | 0.675125644 |
| Riau | 0.527812917 | 0.539003159 | 0.549370911 | 0.558666293 | 0.567224066 | 0.574760449 | 0.581775474 | 0.588952942 | 0.594783332 | 0.600042462 | 0.605197719 | 0.610016302 | 0.614727025 | 0.618967317 | 0.622763139 | 0.626268583 | 0.629925349 | 0.634407916 | 0.640002951 | 0.645998296 | 0.652603156 | 0.659967663 | 0.667502166 | 0.674902955 | 0.681986844 | 0.68878372 | 0.695186835 | 0.701317593 | 0.707257738 | 0.7130141 | 0.717963462 | 0.722784516 |
| West Sulawesi | 0.388169169 | 0.396456145 | 0.404603634 | 0.412332862 | 0.419839087 | 0.426883335 | 0.433684845 | 0.44045657 | 0.444978373 | 0.448429021 | 0.450863688 | 0.452648868 | 0.454577399 | 0.456679678 | 0.458984687 | 0.461742845 | 0.465298754 | 0.470215932 | 0.476854961 | 0.484306447 | 0.49271753 | 0.501942748 | 0.511091619 | 0.519918389 | 0.528304505 | 0.536275541 | 0.543781967 | 0.55095746 | 0.557861768 | 0.564530581 | 0.570184707 | 0.57565685 |
| South Sulawesi | 0.41327701 | 0.422717823 | 0.432096037 | 0.441230921 | 0.450205126 | 0.458776699 | 0.467112157 | 0.475422193 | 0.481601972 | 0.486907342 | 0.491639048 | 0.495941029 | 0.500368448 | 0.504825692 | 0.509234352 | 0.513644342 | 0.518364966 | 0.523951522 | 0.530707433 | 0.538061796 | 0.5461011 | 0.554615251 | 0.562834758 | 0.570714055 | 0.578224344 | 0.585400839 | 0.592225244 | 0.598734353 | 0.604977681 | 0.611023274 | 0.61614532 | 0.621135414 |
| Central Sulawesi | 0.411857501 | 0.420823131 | 0.42982927 | 0.438825231 | 0.447877725 | 0.456371897 | 0.4643591 | 0.472248668 | 0.478015632 | 0.483022386 | 0.487363918 | 0.491258709 | 0.495361968 | 0.499501472 | 0.50362144 | 0.507683516 | 0.512010606 | 0.517204666 | 0.523589477 | 0.530574154 | 0.538375105 | 0.547028684 | 0.555506977 | 0.563674043 | 0.571442152 | 0.578865703 | 0.58590289 | 0.592651712 | 0.599163424 | 0.60551197 | 0.610947884 | 0.616253843 |
| Southeast Sulawesi | 0.386931522 | 0.397433053 | 0.407674437 | 0.417535343 | 0.427348765 | 0.43685672 | 0.446111545 | 0.455278285 | 0.462217344 | 0.468035766 | 0.473014156 | 0.477524073 | 0.482318105 | 0.487364286 | 0.492611183 | 0.498320634 | 0.504583439 | 0.511835478 | 0.520301018 | 0.529039547 | 0.538055432 | 0.547405291 | 0.556473911 | 0.565102723 | 0.573186206 | 0.58073303 | 0.587589402 | 0.593943409 | 0.599972037 | 0.605796318 | 0.610617761 | 0.615325868 |
| North Sulawesi | 0.479692906 | 0.487089939 | 0.494600444 | 0.502300884 | 0.510088618 | 0.517712785 | 0.525358743 | 0.532788289 | 0.537612742 | 0.541079774 | 0.543639487 | 0.545565233 | 0.547512917 | 0.549528384 | 0.551692018 | 0.554190053 | 0.557176701 | 0.561159049 | 0.566428547 | 0.572370583 | 0.579071074 | 0.586487286 | 0.59387226 | 0.601106572 | 0.608061763 | 0.614788165 | 0.621308552 | 0.627679021 | 0.633913222 | 0.640027764 | 0.645296607 | 0.650468306 |
| West Sumatra | 0.465210234 | 0.474324214 | 0.483333039 | 0.492277723 | 0.501058181 | 0.509330998 | 0.517298811 | 0.525176518 | 0.530886205 | 0.535735015 | 0.539886646 | 0.54357323 | 0.547454891 | 0.551496864 | 0.555708188 | 0.560237942 | 0.565251968 | 0.571087098 | 0.577808789 | 0.584755505 | 0.592201612 | 0.600186895 | 0.608067951 | 0.6157333 | 0.623009563 | 0.630032184 | 0.636701359 | 0.643146231 | 0.649369733 | 0.655481061 | 0.660658938 | 0.665763664 |
| South Sumatra | 0.456248761 | 0.465829716 | 0.474790255 | 0.48295831 | 0.490696948 | 0.497941212 | 0.505024074 | 0.512306214 | 0.517875345 | 0.522473583 | 0.526408004 | 0.529822475 | 0.53328289 | 0.53690512 | 0.540676675 | 0.544763338 | 0.549304001 | 0.554641094 | 0.560854314 | 0.567212125 | 0.574047631 | 0.581486893 | 0.588950072 | 0.596305 | 0.603324103 | 0.610156753 | 0.616681654 | 0.623004343 | 0.629151996 | 0.635213318 | 0.640410331 | 0.645536761 |
| North Sumatra | 0.469947965 | 0.479159906 | 0.488431968 | 0.497510076 | 0.506390271 | 0.514469837 | 0.522109974 | 0.529810711 | 0.535438327 | 0.540252562 | 0.5441669 | 0.547520177 | 0.551144953 | 0.55502277 | 0.559075862 | 0.563275349 | 0.567830079 | 0.573166133 | 0.579450903 | 0.586074631 | 0.593325838 | 0.601253317 | 0.609014729 | 0.616579339 | 0.623790139 | 0.630685848 | 0.637391528 | 0.643873462 | 0.650203848 | 0.656407291 | 0.661745515 | 0.667025647 |
| Yogyakarta | 0.463588443 | 0.473680778 | 0.484262987 | 0.495513713 | 0.506926164 | 0.517913177 | 0.528175512 | 0.537648885 | 0.544056497 | 0.549395041 | 0.553914174 | 0.557779636 | 0.561647556 | 0.565581872 | 0.569673465 | 0.574038039 | 0.578881552 | 0.584569879 | 0.591113861 | 0.597928843 | 0.605234562 | 0.613075874 | 0.620744175 | 0.628167845 | 0.635211665 | 0.641952194 | 0.648316783 | 0.654442068 | 0.660364272 | 0.666132809 | 0.670861084 | 0.675429917 |
| Laos | 0.264283164 | 0.268787618 | 0.273435312 | 0.278140418 | 0.28327023 | 0.288795341 | 0.294838372 | 0.301477967 | 0.308402545 | 0.315972615 | 0.323947359 | 0.332580409 | 0.341667271 | 0.350658178 | 0.359681759 | 0.368728678 | 0.378122281 | 0.387723649 | 0.397011299 | 0.406139022 | 0.414865566 | 0.423354523 | 0.431149361 | 0.438691039 | 0.445869424 | 0.452749603 | 0.459583996 | 0.466168892 | 0.472618103 | 0.478811373 | 0.484192959 | 0.489136091 |
| Malaysia | 0.545799405 | 0.551683548 | 0.558186752 | 0.565845656 | 0.574380446 | 0.583441103 | 0.593152135 | 0.603418105 | 0.612645412 | 0.621724901 | 0.630523544 | 0.638257477 | 0.645044248 | 0.650885678 | 0.656243326 | 0.661242241 | 0.665956287 | 0.670843726 | 0.67639991 | 0.682070654 | 0.688258622 | 0.694083403 | 0.699664734 | 0.704930717 | 0.709953756 | 0.714659871 | 0.719223425 | 0.724235412 | 0.729388078 | 0.734587751 | 0.738743993 | 0.742523828 |
| Maldives | 0.331601544 | 0.344398607 | 0.35825234 | 0.372806999 | 0.388081604 | 0.403945583 | 0.420519128 | 0.437436026 | 0.453973361 | 0.469338066 | 0.483495671 | 0.495923695 | 0.50740486 | 0.518926864 | 0.529910191 | 0.538253332 | 0.547950412 | 0.557529247 | 0.566839634 | 0.57444206 | 0.581980986 | 0.58968446 | 0.596948519 | 0.604238519 | 0.611578071 | 0.61845439 | 0.62487595 | 0.631083288 | 0.637205659 | 0.643082794 | 0.647608589 | 0.650886627 |
| Mauritius | 0.544586533 | 0.548209121 | 0.55345463 | 0.56068864 | 0.569092171 | 0.57731939 | 0.584797061 | 0.591497735 | 0.597363039 | 0.602034234 | 0.607087901 | 0.612240817 | 0.617190007 | 0.622764533 | 0.629030193 | 0.63476658 | 0.639545183 | 0.644358656 | 0.649630852 | 0.655000295 | 0.6604698 | 0.666497627 | 0.67312765 | 0.680114023 | 0.686616503 | 0.69223973 | 0.697020562 | 0.701618136 | 0.706266651 | 0.710935045 | 0.714629804 | 0.718260446 |
| Myanmar | 0.319219724 | 0.322034719 | 0.325628884 | 0.329707434 | 0.33429012 | 0.339590976 | 0.345191641 | 0.350892883 | 0.356696902 | 0.363037732 | 0.370360017 | 0.37817198 | 0.386343628 | 0.395218766 | 0.404692358 | 0.414662082 | 0.424705071 | 0.434536927 | 0.443595531 | 0.451954884 | 0.460180429 | 0.467850872 | 0.475336423 | 0.482821855 | 0.490255145 | 0.497374156 | 0.504115978 | 0.510823431 | 0.517557469 | 0.52410897 | 0.529434703 | 0.53390084 |
| Philippines | 0.510011796 | 0.513707183 | 0.517109502 | 0.520600378 | 0.524613607 | 0.528827907 | 0.532938849 | 0.536589868 | 0.53949647 | 0.542257968 | 0.544997723 | 0.547409432 | 0.549521153 | 0.551567968 | 0.553704287 | 0.555664103 | 0.557976265 | 0.560822024 | 0.563974721 | 0.56735582 | 0.571765888 | 0.577240283 | 0.583767625 | 0.590837549 | 0.59838452 | 0.606215624 | 0.614224961 | 0.622315029 | 0.630464276 | 0.638547384 | 0.644867233 | 0.651219329 |
| Abra | 0.493114157 | 0.498240114 | 0.502877528 | 0.507468037 | 0.512240724 | 0.516914091 | 0.521143513 | 0.524627936 | 0.527188491 | 0.529350873 | 0.531280711 | 0.532737179 | 0.533768011 | 0.534780507 | 0.535920878 | 0.536974187 | 0.53856713 | 0.54110627 | 0.544557477 | 0.548797095 | 0.554289832 | 0.560962093 | 0.568787728 | 0.577236327 | 0.586248815 | 0.595549477 | 0.60492779 | 0.614256225 | 0.62341822 | 0.632369231 | 0.639530606 | 0.646546912 |
| Agusan Del Norte | 0.488478852 | 0.491758668 | 0.494592713 | 0.497382002 | 0.500520542 | 0.503695088 | 0.506641442 | 0.508979151 | 0.510384405 | 0.511557489 | 0.512596477 | 0.513204646 | 0.513410804 | 0.513513573 | 0.51379208 | 0.513946345 | 0.514626951 | 0.516154741 | 0.518496374 | 0.52144351 | 0.525597218 | 0.530978057 | 0.537616746 | 0.544938187 | 0.55285983 | 0.561185418 | 0.56979452 | 0.578497674 | 0.587159006 | 0.595781073 | 0.602572879 | 0.609360621 |
| Agusan Del Sur | 0.394358429 | 0.398943598 | 0.403099123 | 0.407256794 | 0.411808217 | 0.416351049 | 0.420605615 | 0.424133121 | 0.426757 | 0.429158978 | 0.431407844 | 0.433208878 | 0.434620478 | 0.435974208 | 0.437421416 | 0.438740048 | 0.440524103 | 0.443080836 | 0.446310351 | 0.450082446 | 0.455025254 | 0.461014105 | 0.468096015 | 0.47573261 | 0.483899359 | 0.492382626 | 0.501129634 | 0.509986887 | 0.518932093 | 0.527949309 | 0.535185779 | 0.542413674 |
| Aklan | 0.500143976 | 0.503005165 | 0.505672601 | 0.508519903 | 0.511977488 | 0.515774545 | 0.519592613 | 0.522999766 | 0.525531724 | 0.527903028 | 0.530223477 | 0.53217388 | 0.533853013 | 0.535631882 | 0.537765954 | 0.539931972 | 0.542630859 | 0.546237005 | 0.550597629 | 0.555223872 | 0.560758296 | 0.56692079 | 0.573868673 | 0.581215499 | 0.588860028 | 0.59662837 | 0.604503516 | 0.612352923 | 0.620160642 | 0.627954298 | 0.633931326 | 0.640074805 |
| Albay | 0.488673629 | 0.491577906 | 0.494292548 | 0.497167734 | 0.500767412 | 0.50478604 | 0.50882488 | 0.512453496 | 0.515364353 | 0.518140187 | 0.520930143 | 0.523396543 | 0.525608532 | 0.527881415 | 0.530422667 | 0.532925061 | 0.535996097 | 0.539869616 | 0.544407098 | 0.549303518 | 0.555136049 | 0.561669225 | 0.569040642 | 0.57679863 | 0.584911814 | 0.593244391 | 0.601663761 | 0.610068339 | 0.61845767 | 0.62675705 | 0.633276353 | 0.639863402 |
| Antique | 0.401776103 | 0.408036697 | 0.413740711 | 0.419361308 | 0.425361161 | 0.431335493 | 0.436817936 | 0.441544273 | 0.445299125 | 0.44869387 | 0.451788351 | 0.454292556 | 0.456238933 | 0.458102065 | 0.459994135 | 0.461637242 | 0.463672201 | 0.466517665 | 0.470202684 | 0.474588693 | 0.480238594 | 0.486884424 | 0.494670464 | 0.503093316 | 0.512029664 | 0.521345939 | 0.530809364 | 0.540324219 | 0.549782409 | 0.559183672 | 0.566787755 | 0.57433252 |
| Apayao | 0.435660953 | 0.440534223 | 0.44499167 | 0.449480294 | 0.454497768 | 0.459735846 | 0.464886627 | 0.469586845 | 0.473454544 | 0.477184743 | 0.480860032 | 0.48416371 | 0.487165312 | 0.490185271 | 0.4935853 | 0.497059497 | 0.501058698 | 0.50583892 | 0.511262831 | 0.51696672 | 0.523624105 | 0.530912382 | 0.538997921 | 0.547524064 | 0.556199045 | 0.564862205 | 0.573456291 | 0.581940082 | 0.590278737 | 0.598449726 | 0.604754734 | 0.611059719 |
| Aurora | 0.451738519 | 0.456950748 | 0.46184658 | 0.466781985 | 0.472254264 | 0.477906092 | 0.483338571 | 0.488149519 | 0.492013942 | 0.49558613 | 0.499004794 | 0.501987368 | 0.50456618 | 0.507100226 | 0.509681067 | 0.512062502 | 0.514770387 | 0.518162507 | 0.522061299 | 0.526335729 | 0.531638787 | 0.537724482 | 0.544850651 | 0.552660355 | 0.560787515 | 0.56896931 | 0.577171834 | 0.585376562 | 0.593593198 | 0.601794783 | 0.608145361 | 0.614506074 |
| Basilan | 0.350254265 | 0.357039364 | 0.363322389 | 0.369543406 | 0.37621656 | 0.383008064 | 0.389521453 | 0.395442204 | 0.400600427 | 0.405563315 | 0.410375678 | 0.414741658 | 0.418702161 | 0.422518058 | 0.426225306 | 0.429588176 | 0.433272688 | 0.437528078 | 0.442310817 | 0.447511662 | 0.453745201 | 0.460913568 | 0.468950801 | 0.477437642 | 0.486316616 | 0.495410346 | 0.504645911 | 0.513935146 | 0.523203062 | 0.53235515 | 0.539644709 | 0.546825716 |
| Bataan | 0.517309034 | 0.520735717 | 0.523928236 | 0.527231496 | 0.531170753 | 0.535383608 | 0.539494776 | 0.54317543 | 0.546147216 | 0.549052443 | 0.551947704 | 0.554487645 | 0.556723508 | 0.558893568 | 0.561087586 | 0.563053729 | 0.565335079 | 0.568207477 | 0.571582439 | 0.575175054 | 0.579697491 | 0.584985523 | 0.591126791 | 0.597803808 | 0.604979657 | 0.612505851 | 0.620275462 | 0.628161655 | 0.636197248 | 0.644260586 | 0.650610439 | 0.656901515 |
| Batanes | 0.528049634 | 0.531939891 | 0.535585737 | 0.539339097 | 0.54393731 | 0.54910787 | 0.554562617 | 0.559594854 | 0.5634099 | 0.566946327 | 0.570591094 | 0.574018099 | 0.577282258 | 0.580516134 | 0.583982984 | 0.587495348 | 0.591652521 | 0.596644919 | 0.602111657 | 0.607391062 | 0.613190702 | 0.61895739 | 0.625147596 | 0.631710267 | 0.638454329 | 0.645197179 | 0.651902753 | 0.658631431 | 0.665392083 | 0.672192879 | 0.677072361 | 0.682304862 |
| Batangas | 0.552429857 | 0.555717665 | 0.558729844 | 0.561799595 | 0.565363594 | 0.569134324 | 0.572820149 | 0.576062009 | 0.578537285 | 0.580780854 | 0.58297807 | 0.584840845 | 0.586454679 | 0.588105378 | 0.589894513 | 0.591534161 | 0.593554173 | 0.596220047 | 0.599435147 | 0.603023074 | 0.607605939 | 0.612993825 | 0.619279288 | 0.626088925 | 0.633376622 | 0.640971253 | 0.648749731 | 0.656582353 | 0.664446337 | 0.672209371 | 0.678279522 | 0.684411269 |
| Benguet | 0.584464349 | 0.58722893 | 0.589732125 | 0.59234214 | 0.59546542 | 0.598804337 | 0.602104872 | 0.604939372 | 0.606964205 | 0.608896272 | 0.610887176 | 0.612651089 | 0.614277795 | 0.616001798 | 0.617993824 | 0.619963776 | 0.622429335 | 0.625666771 | 0.629537943 | 0.633715161 | 0.638768728 | 0.644452069 | 0.650850345 | 0.657592004 | 0.664652723 | 0.672013873 | 0.679626654 | 0.687280249 | 0.694831439 | 0.702234858 | 0.707832924 | 0.713503653 |
| Biliran | 0.488957278 | 0.493982806 | 0.498632759 | 0.503306257 | 0.508460646 | 0.51375464 | 0.518918048 | 0.523558344 | 0.52737613 | 0.530937113 | 0.534363029 | 0.53739152 | 0.54004356 | 0.542689362 | 0.545424974 | 0.547984814 | 0.550826801 | 0.554307619 | 0.558449704 | 0.562779869 | 0.567783697 | 0.573256575 | 0.579397707 | 0.585956257 | 0.592990601 | 0.600351251 | 0.60789209 | 0.615553294 | 0.623379092 | 0.631300515 | 0.637642809 | 0.644053354 |
| Bohol | 0.443903869 | 0.448302517 | 0.452348692 | 0.456438737 | 0.461137897 | 0.466146757 | 0.471126903 | 0.475564943 | 0.479107722 | 0.482418945 | 0.485595858 | 0.488287203 | 0.490605979 | 0.492812912 | 0.495182942 | 0.497454551 | 0.500177674 | 0.503699305 | 0.507945852 | 0.512612142 | 0.518259894 | 0.524737473 | 0.532207002 | 0.540209303 | 0.548502955 | 0.556941413 | 0.565468842 | 0.57392992 | 0.582293907 | 0.590590407 | 0.597147302 | 0.603780595 |
| Bukidnon | 0.399380416 | 0.404023114 | 0.408275929 | 0.412588651 | 0.417425495 | 0.42236631 | 0.427035384 | 0.431084392 | 0.434323197 | 0.437297738 | 0.440079094 | 0.442330742 | 0.444111966 | 0.445788939 | 0.447443537 | 0.448824221 | 0.450490222 | 0.452759167 | 0.455643951 | 0.459055161 | 0.463623799 | 0.469308272 | 0.476188358 | 0.483684513 | 0.491755064 | 0.50024768 | 0.509001016 | 0.517913126 | 0.526952197 | 0.535963883 | 0.543268199 | 0.550516848 |
| Bulacan | 0.562840071 | 0.566428461 | 0.569766048 | 0.57318035 | 0.577094385 | 0.581283004 | 0.585510602 | 0.589436448 | 0.592693193 | 0.595756173 | 0.59879603 | 0.601521059 | 0.604015596 | 0.606536583 | 0.609258565 | 0.611889156 | 0.614894205 | 0.618507787 | 0.622582887 | 0.626859174 | 0.631975557 | 0.637749415 | 0.644303526 | 0.651246474 | 0.65846391 | 0.665868069 | 0.673396649 | 0.680934637 | 0.688456064 | 0.695882857 | 0.701654078 | 0.707522024 |
| Cagayan | 0.476523863 | 0.480897844 | 0.484963897 | 0.489117194 | 0.493926938 | 0.498971508 | 0.50394762 | 0.508414834 | 0.512001765 | 0.515321235 | 0.518498351 | 0.521208829 | 0.523500869 | 0.52569649 | 0.527944022 | 0.53001031 | 0.532466139 | 0.535702946 | 0.539629579 | 0.543976721 | 0.549380608 | 0.5556774 | 0.562973051 | 0.570884107 | 0.579244506 | 0.587800749 | 0.596503049 | 0.605148704 | 0.613709783 | 0.622083301 | 0.628604784 | 0.635068086 |
| Camarines Norte | 0.454389757 | 0.457840474 | 0.461063575 | 0.464462618 | 0.468428777 | 0.472583487 | 0.476532628 | 0.47984856 | 0.482216625 | 0.484312886 | 0.486281657 | 0.487770933 | 0.488860739 | 0.489877625 | 0.491244325 | 0.492612777 | 0.494568868 | 0.497402237 | 0.500922028 | 0.504811706 | 0.509699364 | 0.515479392 | 0.522340247 | 0.529816659 | 0.53781991 | 0.546123308 | 0.554623171 | 0.563232708 | 0.571861908 | 0.580495589 | 0.587383515 | 0.594286494 |
| Camarines Sur | 0.485164875 | 0.489361424 | 0.493200974 | 0.49705719 | 0.501506153 | 0.506143495 | 0.510733672 | 0.514853108 | 0.518063872 | 0.52104005 | 0.523883282 | 0.526299288 | 0.528360344 | 0.530350726 | 0.532343301 | 0.534127585 | 0.536272122 | 0.539133264 | 0.542684365 | 0.546575346 | 0.551429637 | 0.557056401 | 0.563639173 | 0.570799212 | 0.578468162 | 0.58641965 | 0.594578974 | 0.602821155 | 0.611079036 | 0.619296162 | 0.625866263 | 0.632554644 |
| Camiguin | 0.495305819 | 0.498206943 | 0.500951424 | 0.503858005 | 0.507181188 | 0.510702709 | 0.514173609 | 0.517129638 | 0.519112757 | 0.520852399 | 0.522573272 | 0.524148202 | 0.525565882 | 0.526986406 | 0.529062747 | 0.531424811 | 0.534507704 | 0.538523449 | 0.543214244 | 0.547879442 | 0.553380609 | 0.55942911 | 0.56627343 | 0.573676715 | 0.581440997 | 0.589335931 | 0.597333927 | 0.605312388 | 0.613184785 | 0.620903809 | 0.626572391 | 0.632437897 |
| Capiz | 0.42655316 | 0.430796418 | 0.434569814 | 0.438338288 | 0.442581245 | 0.446866816 | 0.450868951 | 0.454242389 | 0.456737284 | 0.458974499 | 0.461021309 | 0.462491556 | 0.463480089 | 0.4644371 | 0.465643373 | 0.466692329 | 0.468227572 | 0.470604782 | 0.473824656 | 0.47749504 | 0.482229395 | 0.488024175 | 0.494948413 | 0.502382707 | 0.510403655 | 0.518825313 | 0.527520206 | 0.536345227 | 0.545179631 | 0.553962793 | 0.561020374 | 0.568087958 |
| Catanduanes | 0.462551509 | 0.467032831 | 0.471102942 | 0.475205722 | 0.479818552 | 0.484521052 | 0.489002939 | 0.492805339 | 0.495766235 | 0.498440936 | 0.500913206 | 0.502899052 | 0.504401341 | 0.505785908 | 0.507128669 | 0.508099417 | 0.509278286 | 0.510966172 | 0.513200008 | 0.516083848 | 0.520204633 | 0.525721005 | 0.532653669 | 0.540349577 | 0.548788234 | 0.557617772 | 0.566670326 | 0.575856953 | 0.585134144 | 0.594436086 | 0.602012226 | 0.609478687 |
| Cavite | 0.610211825 | 0.612466496 | 0.614431865 | 0.616456474 | 0.619049671 | 0.621937059 | 0.624878303 | 0.627476237 | 0.629373258 | 0.631148984 | 0.632947768 | 0.634480968 | 0.63585756 | 0.637359874 | 0.639071915 | 0.640777448 | 0.642914798 | 0.645733833 | 0.649087376 | 0.652661106 | 0.657138814 | 0.662267839 | 0.668092632 | 0.674312847 | 0.680870155 | 0.68761723 | 0.694576246 | 0.701607673 | 0.708706241 | 0.715790429 | 0.721230565 | 0.726784372 |
| Cebu | 0.532735498 | 0.535184099 | 0.537409336 | 0.539696072 | 0.542495726 | 0.545542569 | 0.54856922 | 0.551193724 | 0.553063284 | 0.554743529 | 0.556428985 | 0.557830636 | 0.559040875 | 0.560342736 | 0.561830984 | 0.563279432 | 0.565181382 | 0.567863173 | 0.57124477 | 0.574954317 | 0.579631325 | 0.585043289 | 0.591311227 | 0.598060975 | 0.605272376 | 0.612818718 | 0.620623544 | 0.628513326 | 0.636378419 | 0.64408431 | 0.65001485 | 0.656076358 |
| Cotabato (North Cotabato) | 0.424363132 | 0.42818722 | 0.431601429 | 0.435025749 | 0.438957556 | 0.443011906 | 0.446847905 | 0.450027437 | 0.45227877 | 0.454300441 | 0.456170858 | 0.457523295 | 0.458411121 | 0.459181734 | 0.460048427 | 0.460650591 | 0.461659368 | 0.463338975 | 0.465597325 | 0.468342094 | 0.472242717 | 0.477272922 | 0.483517259 | 0.490461994 | 0.497983512 | 0.505972664 | 0.514248317 | 0.522731641 | 0.531342073 | 0.539995654 | 0.54690644 | 0.55377082 |
| Davao de Oro | 0.394188933 | 0.398475716 | 0.402272643 | 0.406034811 | 0.410264612 | 0.414613669 | 0.418697157 | 0.422188964 | 0.42477907 | 0.427079438 | 0.42921786 | 0.430839045 | 0.432015176 | 0.433058501 | 0.434174843 | 0.43514979 | 0.436631309 | 0.438897824 | 0.44175208 | 0.445121594 | 0.449694104 | 0.455361839 | 0.462189619 | 0.469662693 | 0.477613471 | 0.485841932 | 0.494311082 | 0.502982735 | 0.511743368 | 0.520520608 | 0.52746244 | 0.534417764 |
| Davao Del Norte | 0.47725283 | 0.482257954 | 0.486909844 | 0.491538247 | 0.4966396 | 0.501958362 | 0.507110136 | 0.511742636 | 0.515444042 | 0.518837267 | 0.522120843 | 0.525056469 | 0.527697642 | 0.530312204 | 0.533089241 | 0.535758115 | 0.538817069 | 0.542488042 | 0.546598406 | 0.550971917 | 0.556183654 | 0.562072744 | 0.568831342 | 0.576031955 | 0.583640436 | 0.5915541 | 0.599766883 | 0.608107427 | 0.61643135 | 0.624740349 | 0.631337641 | 0.638104952 |
| Davao Del Sur | 0.509118509 | 0.513016088 | 0.516571489 | 0.52016433 | 0.524170872 | 0.528321223 | 0.532319316 | 0.535822319 | 0.538557143 | 0.541112922 | 0.543631751 | 0.545757805 | 0.547570063 | 0.549425211 | 0.551430462 | 0.553347585 | 0.555726991 | 0.558880834 | 0.562663382 | 0.56688136 | 0.572182857 | 0.578391669 | 0.585621961 | 0.593481223 | 0.601876316 | 0.61064279 | 0.619648579 | 0.628677465 | 0.637614829 | 0.646313325 | 0.653169735 | 0.660001466 |
| Davao Occidental | 0.388923824 | 0.394538632 | 0.399788939 | 0.405090332 | 0.410838851 | 0.416692939 | 0.422258121 | 0.42732412 | 0.431901298 | 0.436420405 | 0.440860226 | 0.444907536 | 0.448568078 | 0.452112089 | 0.455533386 | 0.458604198 | 0.461903194 | 0.46566822 | 0.469958105 | 0.474882209 | 0.480874138 | 0.488191655 | 0.496780711 | 0.50589737 | 0.515619953 | 0.525617584 | 0.535665984 | 0.545661611 | 0.555472435 | 0.565128605 | 0.573384744 | 0.5814625 |
| Davao Oriental | 0.385245835 | 0.389594617 | 0.393582355 | 0.397643446 | 0.402508439 | 0.407752423 | 0.413024333 | 0.417851716 | 0.421871716 | 0.425709117 | 0.429476315 | 0.432803996 | 0.435689614 | 0.438497556 | 0.441241804 | 0.443590743 | 0.446160164 | 0.449306319 | 0.452927517 | 0.45694815 | 0.461944146 | 0.46785605 | 0.474789459 | 0.482306052 | 0.490335155 | 0.498679178 | 0.507177286 | 0.515750388 | 0.524375165 | 0.532986088 | 0.539850269 | 0.546673438 |
| Dinagat Islands | 0.45740369 | 0.463025617 | 0.468196986 | 0.473345622 | 0.478953085 | 0.484672292 | 0.490109225 | 0.494991968 | 0.499196657 | 0.503243733 | 0.507154632 | 0.510705347 | 0.513803716 | 0.516720982 | 0.519596119 | 0.522186472 | 0.525008417 | 0.528265757 | 0.531762007 | 0.535481642 | 0.540121381 | 0.545719467 | 0.552349102 | 0.55945463 | 0.567025332 | 0.574906725 | 0.582980458 | 0.591182512 | 0.59934323 | 0.607509149 | 0.61416855 | 0.620781361 |
| Eastern Samar | 0.358787376 | 0.362878035 | 0.36639932 | 0.369924619 | 0.373965159 | 0.378004916 | 0.38158973 | 0.384331237 | 0.38619929 | 0.387758011 | 0.389039162 | 0.389672025 | 0.389645074 | 0.389378809 | 0.389224429 | 0.388795203 | 0.388861766 | 0.389830749 | 0.391696228 | 0.394397363 | 0.398519239 | 0.404410106 | 0.41208072 | 0.420475465 | 0.429674338 | 0.439358519 | 0.449295635 | 0.459424451 | 0.469637069 | 0.479797133 | 0.487963835 | 0.495675232 |
| Guimaras | 0.448414677 | 0.453165677 | 0.457533785 | 0.46197678 | 0.466960044 | 0.472143782 | 0.477133844 | 0.481523946 | 0.484957624 | 0.488114925 | 0.491143482 | 0.493704605 | 0.495852275 | 0.497981683 | 0.500569417 | 0.503233772 | 0.506451757 | 0.510539529 | 0.515348727 | 0.520282617 | 0.526019269 | 0.532405448 | 0.539637729 | 0.547314773 | 0.555285735 | 0.563419609 | 0.571613585 | 0.579776635 | 0.58797511 | 0.596206239 | 0.602688283 | 0.609240673 |
| Ifugao | 0.442548221 | 0.447995267 | 0.45305081 | 0.458133786 | 0.463600232 | 0.46907812 | 0.474208503 | 0.478664686 | 0.482238057 | 0.485578325 | 0.488721388 | 0.491342902 | 0.493458939 | 0.495407877 | 0.497422378 | 0.499215428 | 0.501312155 | 0.504009273 | 0.507193837 | 0.510768402 | 0.515415522 | 0.521163259 | 0.527970465 | 0.535244721 | 0.542996183 | 0.551038077 | 0.559220486 | 0.567524773 | 0.575797714 | 0.584066632 | 0.590626782 | 0.597128222 |
| Ilocos Norte | 0.556207974 | 0.559411424 | 0.562402595 | 0.565460193 | 0.568922794 | 0.572547553 | 0.576090887 | 0.579200076 | 0.58153885 | 0.583742928 | 0.585925877 | 0.587783118 | 0.589399404 | 0.59100477 | 0.592753883 | 0.594419442 | 0.596553409 | 0.599430239 | 0.602955001 | 0.606837788 | 0.611664846 | 0.617276026 | 0.623725207 | 0.630591254 | 0.637745801 | 0.645109112 | 0.652585884 | 0.660099008 | 0.667615159 | 0.675079173 | 0.680936786 | 0.686921587 |
| Ilocos Sur | 0.524452639 | 0.528775256 | 0.532831024 | 0.536976835 | 0.541527269 | 0.546162984 | 0.550553538 | 0.554366487 | 0.557282695 | 0.559956615 | 0.562520154 | 0.564648724 | 0.566455195 | 0.568224882 | 0.570229584 | 0.572229307 | 0.574701329 | 0.577931419 | 0.581835587 | 0.586062387 | 0.59125395 | 0.59725136 | 0.604179096 | 0.611568025 | 0.619340291 | 0.627308584 | 0.635434293 | 0.643524963 | 0.651565539 | 0.659546276 | 0.665894293 | 0.672277756 |
| Iloilo | 0.527433705 | 0.531643384 | 0.535534166 | 0.539448689 | 0.543682832 | 0.548062125 | 0.552240901 | 0.555844502 | 0.558512792 | 0.560955746 | 0.56335959 | 0.565378105 | 0.567142498 | 0.568929779 | 0.57108015 | 0.57327554 | 0.575997995 | 0.579634914 | 0.58399969 | 0.588639059 | 0.594201905 | 0.600350153 | 0.60725643 | 0.614592781 | 0.622252546 | 0.63002535 | 0.637910076 | 0.645763821 | 0.653490129 | 0.661115028 | 0.666937332 | 0.672917821 |
| Isabela | 0.486994063 | 0.49145654 | 0.495622618 | 0.49979648 | 0.504431543 | 0.509272084 | 0.513958911 | 0.518081911 | 0.521359963 | 0.524337691 | 0.527167725 | 0.529544065 | 0.531544118 | 0.53351323 | 0.53560178 | 0.537523627 | 0.539847748 | 0.542869592 | 0.546496805 | 0.55053601 | 0.555600881 | 0.561535988 | 0.5684623 | 0.575945509 | 0.583846582 | 0.591961524 | 0.600224211 | 0.608473283 | 0.616691591 | 0.624772308 | 0.631042607 | 0.637319646 |
| Kalinga | 0.396586905 | 0.402252916 | 0.407611127 | 0.413029176 | 0.418959489 | 0.425070996 | 0.431054064 | 0.436504759 | 0.441050314 | 0.445317027 | 0.449453558 | 0.45307383 | 0.456282074 | 0.45949121 | 0.463000803 | 0.466460039 | 0.470394001 | 0.475135788 | 0.480555167 | 0.486325404 | 0.492996471 | 0.500175723 | 0.508089547 | 0.516391805 | 0.524810592 | 0.533176856 | 0.541489447 | 0.549720992 | 0.557861374 | 0.565903045 | 0.57200029 | 0.578198283 |
| La Union | 0.533148639 | 0.536071317 | 0.538675071 | 0.541342804 | 0.544516874 | 0.547894539 | 0.551145072 | 0.553907373 | 0.555905488 | 0.557709025 | 0.559447153 | 0.560799717 | 0.561819747 | 0.562811207 | 0.563905955 | 0.564865444 | 0.566243754 | 0.568405928 | 0.571243685 | 0.57457037 | 0.579053429 | 0.584544451 | 0.591178639 | 0.598492763 | 0.606413218 | 0.614622791 | 0.623028895 | 0.631447074 | 0.639827859 | 0.648061686 | 0.654517609 | 0.660963661 |
| Laguna | 0.583112351 | 0.585723601 | 0.587962551 | 0.590211801 | 0.592919678 | 0.595829433 | 0.598658227 | 0.601091405 | 0.60282229 | 0.604381112 | 0.605930342 | 0.607168693 | 0.608186913 | 0.60927961 | 0.610612999 | 0.611881468 | 0.613626879 | 0.616113856 | 0.619221235 | 0.622676067 | 0.627029882 | 0.632151505 | 0.638147324 | 0.644604028 | 0.651551499 | 0.658822662 | 0.666339261 | 0.673910371 | 0.681538336 | 0.689098323 | 0.694997121 | 0.700924655 |
| Lanao Del Norte | 0.46393371 | 0.467302053 | 0.470247295 | 0.473227993 | 0.476695249 | 0.480238009 | 0.48352204 | 0.486144581 | 0.48790355 | 0.489433351 | 0.490811053 | 0.491661914 | 0.491990883 | 0.492168039 | 0.492353949 | 0.492237121 | 0.492469258 | 0.493353852 | 0.49493157 | 0.497095161 | 0.500426088 | 0.505078254 | 0.511044597 | 0.517701101 | 0.525141324 | 0.533141501 | 0.541575255 | 0.550278285 | 0.559156547 | 0.568048983 | 0.575309364 | 0.58250552 |
| Lanao Del Sur | 0.393071715 | 0.397833358 | 0.402118499 | 0.406409448 | 0.410865852 | 0.415142323 | 0.418885318 | 0.42184144 | 0.42389968 | 0.425612163 | 0.4270541 | 0.427969337 | 0.428344056 | 0.428633862 | 0.429147049 | 0.429537229 | 0.430476562 | 0.432244231 | 0.434797192 | 0.438023302 | 0.442490207 | 0.448172481 | 0.455077454 | 0.462670056 | 0.470931095 | 0.47958677 | 0.488497819 | 0.497553568 | 0.506725278 | 0.515909614 | 0.523297827 | 0.530702771 |
| Leyte | 0.452754461 | 0.457255664 | 0.461338765 | 0.46545871 | 0.470031788 | 0.474745284 | 0.47934116 | 0.483398128 | 0.486532868 | 0.489419634 | 0.492161869 | 0.494514966 | 0.496515336 | 0.498477722 | 0.500661224 | 0.502775232 | 0.505314697 | 0.508589038 | 0.512521351 | 0.516959355 | 0.522545932 | 0.529053072 | 0.536669566 | 0.544947776 | 0.553651924 | 0.562486181 | 0.571404707 | 0.580239727 | 0.588898636 | 0.597438099 | 0.604161108 | 0.610942558 |
| Maguindanao | 0.338054564 | 0.343896149 | 0.349301553 | 0.354721946 | 0.360538202 | 0.366364899 | 0.371911775 | 0.376854923 | 0.380888447 | 0.384578933 | 0.388073898 | 0.391020554 | 0.393479611 | 0.395795689 | 0.398243161 | 0.400620915 | 0.40344862 | 0.407028579 | 0.411187863 | 0.415689436 | 0.421281179 | 0.427867389 | 0.435490193 | 0.443629051 | 0.452173348 | 0.46098824 | 0.469934359 | 0.478951288 | 0.487995973 | 0.496997247 | 0.504170202 | 0.51129551 |
| Marinduque | 0.415902044 | 0.419914225 | 0.423549257 | 0.427238105 | 0.431340635 | 0.435469671 | 0.43925705 | 0.442293083 | 0.44439647 | 0.446224249 | 0.447807662 | 0.448835177 | 0.449389119 | 0.449842629 | 0.450498745 | 0.450924782 | 0.451783076 | 0.45340269 | 0.455830857 | 0.458979577 | 0.463387611 | 0.469165048 | 0.476386212 | 0.484227603 | 0.492724401 | 0.501588826 | 0.510512741 | 0.519453993 | 0.528413503 | 0.537409494 | 0.544653323 | 0.55186124 |
| Masbate | 0.357228929 | 0.360130301 | 0.362581514 | 0.365084303 | 0.367976114 | 0.370850194 | 0.37327649 | 0.374858543 | 0.375489568 | 0.375839335 | 0.375921216 | 0.375413678 | 0.374287362 | 0.372964418 | 0.371506436 | 0.369597194 | 0.367884607 | 0.366838882 | 0.36655854 | 0.367260942 | 0.369423421 | 0.373479633 | 0.379349859 | 0.385994628 | 0.393665587 | 0.402062834 | 0.410916177 | 0.420090446 | 0.42946856 | 0.438960973 | 0.446644118 | 0.454108095 |
| Misamis Occidental | 0.479137772 | 0.482130921 | 0.484755605 | 0.487375966 | 0.490228026 | 0.493005319 | 0.49542158 | 0.497170318 | 0.497955155 | 0.498451251 | 0.498778934 | 0.49865014 | 0.498149213 | 0.497552641 | 0.497274835 | 0.49699739 | 0.497280907 | 0.498382296 | 0.500215342 | 0.502601019 | 0.506171454 | 0.511005831 | 0.517145559 | 0.523995205 | 0.531455696 | 0.539339113 | 0.547512232 | 0.555821438 | 0.564125635 | 0.572474244 | 0.579088616 | 0.585721104 |
| Misamis Oriental | 0.546148333 | 0.548962943 | 0.551466481 | 0.553998532 | 0.55705062 | 0.5603096 | 0.56349272 | 0.566164354 | 0.567962378 | 0.569569385 | 0.571114255 | 0.572237958 | 0.573036696 | 0.573822765 | 0.574712391 | 0.575430198 | 0.576565569 | 0.578318733 | 0.580562417 | 0.583155295 | 0.586744283 | 0.591273182 | 0.596834241 | 0.603020595 | 0.60979533 | 0.617042719 | 0.624601154 | 0.63232768 | 0.640121068 | 0.647917307 | 0.654033818 | 0.660208517 |
| Mountain Province | 0.384142978 | 0.389890999 | 0.394983191 | 0.399825221 | 0.404880792 | 0.409762101 | 0.414070159 | 0.417486337 | 0.419958369 | 0.421967521 | 0.423576144 | 0.424415756 | 0.424545244 | 0.424380253 | 0.424064876 | 0.423283213 | 0.422717696 | 0.42292593 | 0.424135619 | 0.426306276 | 0.429745883 | 0.434549402 | 0.440726397 | 0.447680786 | 0.455742303 | 0.464620266 | 0.473961155 | 0.483803559 | 0.493937125 | 0.504174238 | 0.512545067 | 0.52052229 |
| National Capital Region | 0.631604081 | 0.633853859 | 0.635862419 | 0.637940062 | 0.640545157 | 0.643394295 | 0.646241426 | 0.648794579 | 0.650709013 | 0.652618625 | 0.654604325 | 0.656332255 | 0.657905204 | 0.659512627 | 0.661330994 | 0.663141961 | 0.665462125 | 0.668555473 | 0.672216085 | 0.676126499 | 0.680945208 | 0.686416526 | 0.692572738 | 0.699092689 | 0.70589905 | 0.712882252 | 0.720027051 | 0.727182234 | 0.734260315 | 0.741208188 | 0.746426063 | 0.751742216 |
| Negros Occidental | 0.492135423 | 0.495108113 | 0.497767506 | 0.500450479 | 0.50352918 | 0.506708844 | 0.509752136 | 0.512205671 | 0.513725278 | 0.514994993 | 0.516161729 | 0.516911833 | 0.51731886 | 0.517660217 | 0.51814239 | 0.518488514 | 0.519298935 | 0.52083332 | 0.523004143 | 0.525621092 | 0.529293467 | 0.533919433 | 0.53958533 | 0.545858223 | 0.552694985 | 0.559971965 | 0.567542441 | 0.575250076 | 0.58299961 | 0.590779078 | 0.596917778 | 0.60317817 |
| Negros Oriental | 0.438173264 | 0.44179132 | 0.445143902 | 0.448556065 | 0.452496588 | 0.456665244 | 0.460740328 | 0.464351625 | 0.467150815 | 0.469714873 | 0.472169983 | 0.474220253 | 0.475935123 | 0.47761205 | 0.479298261 | 0.480762461 | 0.482549151 | 0.48498773 | 0.488058526 | 0.491601083 | 0.496182205 | 0.501567024 | 0.507899076 | 0.514881653 | 0.522414195 | 0.530260189 | 0.53836243 | 0.546541576 | 0.554796992 | 0.563012188 | 0.569530702 | 0.576084024 |
| Northern Samar | 0.377649131 | 0.382447777 | 0.386814676 | 0.391203467 | 0.395976421 | 0.400712917 | 0.405101783 | 0.408750108 | 0.411410092 | 0.413746228 | 0.415835151 | 0.417309689 | 0.41822878 | 0.418972522 | 0.419924882 | 0.420824393 | 0.422263881 | 0.424582606 | 0.427714994 | 0.431290263 | 0.435888653 | 0.441514928 | 0.448270834 | 0.455627291 | 0.463566223 | 0.471915596 | 0.480579279 | 0.489483596 | 0.498557881 | 0.507690373 | 0.515012608 | 0.522209106 |
| Nueva Ecija | 0.531381357 | 0.533786251 | 0.535898211 | 0.538114883 | 0.540863167 | 0.543851476 | 0.546716691 | 0.549103589 | 0.550740588 | 0.55222002 | 0.553666367 | 0.554806852 | 0.55570562 | 0.556664733 | 0.557800362 | 0.558878014 | 0.560402712 | 0.562660895 | 0.565593242 | 0.568938632 | 0.573219502 | 0.578295707 | 0.584291802 | 0.590860928 | 0.597998088 | 0.605509802 | 0.613329772 | 0.621278253 | 0.629315263 | 0.637345883 | 0.643745698 | 0.650166602 |
| Nueva Vizcaya | 0.482176184 | 0.486871208 | 0.490908008 | 0.494807479 | 0.499088297 | 0.503269888 | 0.506990304 | 0.50997028 | 0.512084895 | 0.513901027 | 0.515490166 | 0.516515095 | 0.517136574 | 0.517638035 | 0.518242301 | 0.518662444 | 0.519454152 | 0.520904878 | 0.523090815 | 0.526035992 | 0.530171955 | 0.535633172 | 0.542367452 | 0.549624164 | 0.557555345 | 0.565958793 | 0.574675433 | 0.583593324 | 0.592629367 | 0.601705876 | 0.609047062 | 0.616192946 |
| Occidental Mindoro | 0.339037642 | 0.344967049 | 0.350049168 | 0.354918039 | 0.359983643 | 0.364635755 | 0.368377774 | 0.370958902 | 0.37274986 | 0.374176824 | 0.375166214 | 0.375354313 | 0.374649143 | 0.373513435 | 0.37183662 | 0.36944013 | 0.367149583 | 0.365479093 | 0.36472746 | 0.36531598 | 0.36746442 | 0.371751915 | 0.377965967 | 0.384953826 | 0.393498256 | 0.403174661 | 0.413604149 | 0.424641385 | 0.436083937 | 0.447730879 | 0.457555159 | 0.466835042 |
| Oriental Mindoro | 0.438054195 | 0.443436909 | 0.448351613 | 0.453247272 | 0.458485922 | 0.463696843 | 0.468584791 | 0.472821106 | 0.476154401 | 0.479238124 | 0.482143917 | 0.484633626 | 0.486756393 | 0.488812424 | 0.490998682 | 0.493043222 | 0.495562685 | 0.498890065 | 0.503008853 | 0.507635203 | 0.513390772 | 0.520089304 | 0.527763201 | 0.535919255 | 0.544539453 | 0.553391057 | 0.562378472 | 0.571336174 | 0.58022105 | 0.589074264 | 0.596163334 | 0.603235742 |
| Palawan | 0.391125531 | 0.395348366 | 0.399044743 | 0.402657344 | 0.406857344 | 0.411162737 | 0.415200162 | 0.418529354 | 0.42092942 | 0.423082913 | 0.425051425 | 0.426467154 | 0.427370377 | 0.428129266 | 0.428864144 | 0.429259551 | 0.430027792 | 0.431498188 | 0.433704908 | 0.436541244 | 0.440654056 | 0.446152806 | 0.453037612 | 0.460607176 | 0.468851397 | 0.477569873 | 0.486676785 | 0.496026396 | 0.505468197 | 0.514924801 | 0.522534685 | 0.530005129 |
| Pampanga | 0.557746428 | 0.56147988 | 0.564849035 | 0.56814459 | 0.571902747 | 0.5759152 | 0.579865413 | 0.583400558 | 0.586245314 | 0.588925177 | 0.591613872 | 0.594000074 | 0.59614123 | 0.598301234 | 0.600502693 | 0.602470682 | 0.604754928 | 0.607607688 | 0.610940894 | 0.61458145 | 0.619170993 | 0.624552378 | 0.630845023 | 0.637658628 | 0.644930229 | 0.652514817 | 0.660296555 | 0.668116788 | 0.675994804 | 0.683775116 | 0.689864563 | 0.695999283 |
| Pangasinan | 0.535583582 | 0.538726455 | 0.541605051 | 0.544599252 | 0.548122585 | 0.551787063 | 0.555310788 | 0.558284381 | 0.560421018 | 0.562357038 | 0.564231599 | 0.565742404 | 0.56694724 | 0.568155255 | 0.569523748 | 0.57082225 | 0.572580645 | 0.575084219 | 0.578263835 | 0.58187691 | 0.586549216 | 0.592100896 | 0.598627526 | 0.605766038 | 0.613365562 | 0.621196267 | 0.629213404 | 0.637202407 | 0.645198509 | 0.653106506 | 0.659294182 | 0.66557535 |
| Quezon | 0.494755975 | 0.497904923 | 0.500708955 | 0.503549027 | 0.506778012 | 0.51003994 | 0.513068701 | 0.515543543 | 0.517203748 | 0.518652522 | 0.520046586 | 0.521184022 | 0.52212822 | 0.523158315 | 0.524456969 | 0.52578499 | 0.527665846 | 0.53041695 | 0.533952681 | 0.538089124 | 0.543358401 | 0.549474797 | 0.556576611 | 0.564321996 | 0.572581054 | 0.581074092 | 0.589729568 | 0.598371244 | 0.606955266 | 0.615372393 | 0.621990633 | 0.628562384 |
| Quirino | 0.417716701 | 0.423038166 | 0.428007276 | 0.433081114 | 0.438683669 | 0.444393929 | 0.449863614 | 0.45469782 | 0.458600441 | 0.462218691 | 0.465569722 | 0.468368185 | 0.470613972 | 0.472647021 | 0.474638393 | 0.476296709 | 0.478260043 | 0.480830024 | 0.483971541 | 0.487427557 | 0.491804551 | 0.497173576 | 0.503627409 | 0.510572383 | 0.518029512 | 0.525795397 | 0.533833012 | 0.541942241 | 0.550150055 | 0.558375077 | 0.564940783 | 0.571498837 |
| Rizal | 0.556537796 | 0.559979648 | 0.563691384 | 0.567884345 | 0.573027924 | 0.578714588 | 0.584603287 | 0.590210396 | 0.59504784 | 0.599613195 | 0.603973613 | 0.607792657 | 0.611141819 | 0.614335441 | 0.61742208 | 0.620126512 | 0.622976822 | 0.626241416 | 0.629797381 | 0.633461704 | 0.637943134 | 0.643006195 | 0.64879787 | 0.655012573 | 0.661610803 | 0.668485458 | 0.675633697 | 0.682941302 | 0.690327967 | 0.697721852 | 0.703495306 | 0.709407899 |
| Romblon | 0.373491683 | 0.377969909 | 0.381990022 | 0.386059667 | 0.390773318 | 0.395689695 | 0.400402555 | 0.404387302 | 0.407427651 | 0.410250436 | 0.412865386 | 0.414882958 | 0.416393505 | 0.417815382 | 0.419339213 | 0.420547172 | 0.422108378 | 0.424444103 | 0.427651628 | 0.431618966 | 0.43687056 | 0.443553621 | 0.451723356 | 0.460528923 | 0.469861011 | 0.479370412 | 0.488929312 | 0.498423192 | 0.507875398 | 0.517313797 | 0.524898287 | 0.532390648 |
| Samar (Western Samar) | 0.402122967 | 0.406060274 | 0.40956094 | 0.41303879 | 0.416847929 | 0.420653889 | 0.424128116 | 0.426910148 | 0.428750932 | 0.430251405 | 0.431537681 | 0.432274489 | 0.432526219 | 0.43266752 | 0.433148984 | 0.4336472 | 0.434712466 | 0.436623025 | 0.439277355 | 0.44229819 | 0.446336756 | 0.451453409 | 0.457739529 | 0.464642265 | 0.472058856 | 0.479784565 | 0.487752881 | 0.495883523 | 0.504161079 | 0.512451229 | 0.519013635 | 0.525508195 |
| Sarangani | 0.413238597 | 0.41756627 | 0.421676191 | 0.425924586 | 0.430727485 | 0.435847042 | 0.441004866 | 0.44584224 | 0.449861615 | 0.453705551 | 0.457588511 | 0.461120082 | 0.464434567 | 0.467802201 | 0.471396059 | 0.474916783 | 0.478845906 | 0.483446884 | 0.48840828 | 0.493615078 | 0.499838084 | 0.506630184 | 0.514203783 | 0.522301771 | 0.53047681 | 0.538645119 | 0.54684426 | 0.555023822 | 0.56316235 | 0.57121446 | 0.577447405 | 0.583869433 |
| Siquijor | 0.436579156 | 0.441220798 | 0.445638191 | 0.450172774 | 0.455378212 | 0.460941425 | 0.466650478 | 0.471978037 | 0.476360385 | 0.480491999 | 0.484565442 | 0.488363869 | 0.491907314 | 0.495494278 | 0.498891999 | 0.501825944 | 0.504940677 | 0.508599601 | 0.512653577 | 0.516987145 | 0.522359739 | 0.528345507 | 0.535149307 | 0.542510279 | 0.550130867 | 0.557829559 | 0.565636781 | 0.573468374 | 0.581259283 | 0.588961196 | 0.59472782 | 0.600739699 |
| Sorsogon | 0.451186555 | 0.454349618 | 0.457268478 | 0.460329264 | 0.464040471 | 0.468062619 | 0.47202674 | 0.475468521 | 0.478059933 | 0.480413304 | 0.482692066 | 0.48461039 | 0.486252098 | 0.487953997 | 0.489952729 | 0.491938373 | 0.49446113 | 0.497897116 | 0.502176566 | 0.506830335 | 0.512506412 | 0.519023511 | 0.526488661 | 0.534410309 | 0.54272952 | 0.551285152 | 0.560021906 | 0.568820991 | 0.577648144 | 0.58640728 | 0.59330996 | 0.600254568 |
| South Cotabato | 0.493304671 | 0.49706363 | 0.500426958 | 0.503817861 | 0.507708152 | 0.511725939 | 0.515581972 | 0.518903425 | 0.521396419 | 0.523662082 | 0.525864899 | 0.527642942 | 0.529029988 | 0.530401201 | 0.531929921 | 0.533289421 | 0.535074299 | 0.53759213 | 0.540689889 | 0.544242532 | 0.548904101 | 0.554568304 | 0.561357516 | 0.568775841 | 0.576735431 | 0.58503259 | 0.593530603 | 0.602082252 | 0.610561411 | 0.618930743 | 0.625497041 | 0.632081366 |
| Southern Leyte | 0.444659979 | 0.448982679 | 0.452956869 | 0.456982715 | 0.461473383 | 0.466026471 | 0.470339646 | 0.474005831 | 0.47678152 | 0.479322292 | 0.481713492 | 0.483725613 | 0.485410745 | 0.487131784 | 0.489142697 | 0.491048276 | 0.493502354 | 0.496766171 | 0.500649542 | 0.505048996 | 0.510567604 | 0.517192742 | 0.524949537 | 0.533332551 | 0.542114911 | 0.551038161 | 0.560031258 | 0.56894778 | 0.577750579 | 0.586465963 | 0.593334869 | 0.600211252 |
| Sultan Kudarat | 0.389772512 | 0.394785842 | 0.399217783 | 0.40355312 | 0.408228945 | 0.412786795 | 0.416883858 | 0.42011876 | 0.422402672 | 0.424357512 | 0.426030489 | 0.427101893 | 0.42755292 | 0.427745617 | 0.427871174 | 0.427601431 | 0.427678164 | 0.428416174 | 0.429845939 | 0.431927422 | 0.43524464 | 0.439987864 | 0.44614033 | 0.452972816 | 0.460621764 | 0.468844825 | 0.477547677 | 0.486621148 | 0.495960736 | 0.505394235 | 0.513116997 | 0.520676083 |
| Sulu | 0.291025666 | 0.29649175 | 0.301892531 | 0.30759409 | 0.313756704 | 0.32001469 | 0.326065953 | 0.33159151 | 0.336166694 | 0.340449304 | 0.344636091 | 0.34847542 | 0.352046263 | 0.355681598 | 0.359445381 | 0.363011713 | 0.366932998 | 0.371517606 | 0.376545034 | 0.382027343 | 0.388779226 | 0.396413301 | 0.405014882 | 0.41425509 | 0.42366848 | 0.433075472 | 0.442469819 | 0.451744373 | 0.460891655 | 0.469852519 | 0.476618688 | 0.483535851 |
| Surigao Del Norte | 0.468963284 | 0.472884636 | 0.47654864 | 0.480356046 | 0.48489205 | 0.489801918 | 0.494734744 | 0.49930364 | 0.503123817 | 0.506793805 | 0.51047132 | 0.513786962 | 0.516838046 | 0.519915776 | 0.523038173 | 0.52596493 | 0.529271046 | 0.533151973 | 0.537403266 | 0.541825559 | 0.54712347 | 0.55304533 | 0.559810516 | 0.567004605 | 0.574608732 | 0.582428569 | 0.590426276 | 0.598505407 | 0.606595913 | 0.614657931 | 0.620977478 | 0.627445196 |
| Surigao Del Sur | 0.44007541 | 0.445207018 | 0.449961915 | 0.454740389 | 0.459897717 | 0.46504644 | 0.469852059 | 0.473986797 | 0.477235737 | 0.480264831 | 0.483121665 | 0.48546311 | 0.487357365 | 0.489154691 | 0.490996694 | 0.492659094 | 0.49469545 | 0.497418935 | 0.500714378 | 0.504354046 | 0.509001099 | 0.514461996 | 0.520833996 | 0.527695627 | 0.535063635 | 0.542752704 | 0.550720285 | 0.558849004 | 0.567096735 | 0.575476773 | 0.582103707 | 0.588740715 |
| Tarlac | 0.516577193 | 0.519922204 | 0.523110165 | 0.526450561 | 0.53036813 | 0.534556241 | 0.538590973 | 0.542085881 | 0.544809162 | 0.547277083 | 0.549635012 | 0.551540675 | 0.553077324 | 0.554560119 | 0.556117417 | 0.557442016 | 0.559069245 | 0.561300221 | 0.564082302 | 0.567297011 | 0.571588496 | 0.576887693 | 0.583225867 | 0.590095603 | 0.597492171 | 0.605277883 | 0.613320147 | 0.62144073 | 0.629612045 | 0.637740796 | 0.644190398 | 0.650645532 |
| Tawi-Tawi | 0.362263287 | 0.36809861 | 0.373558566 | 0.379019491 | 0.384748407 | 0.390361297 | 0.395535287 | 0.39997237 | 0.403411219 | 0.406466383 | 0.409299843 | 0.411574241 | 0.413413581 | 0.415166511 | 0.417413255 | 0.419877838 | 0.423115726 | 0.427403683 | 0.432423023 | 0.437730037 | 0.444093309 | 0.451221053 | 0.459207879 | 0.467640618 | 0.47641084 | 0.485412961 | 0.494528873 | 0.503662002 | 0.512772032 | 0.521807301 | 0.528799692 | 0.535812038 |
| Zambales | 0.53965418 | 0.541446109 | 0.543004867 | 0.544689636 | 0.546872467 | 0.549227103 | 0.551467688 | 0.553240691 | 0.554230586 | 0.555102245 | 0.555972249 | 0.556543104 | 0.556866962 | 0.557295367 | 0.557935625 | 0.558490053 | 0.559527045 | 0.561367112 | 0.563885896 | 0.566935015 | 0.571146256 | 0.57629563 | 0.582445698 | 0.589242075 | 0.596688244 | 0.60453675 | 0.612687937 | 0.620988287 | 0.629342064 | 0.637670848 | 0.644268072 | 0.650840597 |
| Zamboanga Del Norte | 0.398464758 | 0.402127833 | 0.405412583 | 0.408717262 | 0.4125698 | 0.416593958 | 0.420464873 | 0.423802083 | 0.426194965 | 0.428362735 | 0.430458881 | 0.432083352 | 0.433280123 | 0.434391779 | 0.435718206 | 0.436950098 | 0.438636763 | 0.441074759 | 0.444194008 | 0.447765524 | 0.45248399 | 0.458245584 | 0.465123477 | 0.472612326 | 0.480568522 | 0.488834265 | 0.497319432 | 0.505932896 | 0.514658009 | 0.523349767 | 0.530290971 | 0.537184539 |
| Zamboanga Del Sur | 0.475176508 | 0.479541486 | 0.483501746 | 0.487530367 | 0.492059601 | 0.496769323 | 0.50135883 | 0.505476055 | 0.50877701 | 0.511864566 | 0.514889737 | 0.517504648 | 0.519832783 | 0.522166864 | 0.524748861 | 0.52730215 | 0.530315949 | 0.53407927 | 0.538418311 | 0.543003284 | 0.548534167 | 0.554779753 | 0.561819564 | 0.569363131 | 0.577256801 | 0.585396585 | 0.593669761 | 0.60194062 | 0.61015045 | 0.618268575 | 0.624552055 | 0.630908782 |
| Zamboanga Sibugay | 0.387055592 | 0.391744715 | 0.396017619 | 0.400336691 | 0.405210618 | 0.410316488 | 0.41527972 | 0.419610867 | 0.423007552 | 0.426101387 | 0.429072998 | 0.431565003 | 0.43365052 | 0.435627876 | 0.437898889 | 0.440089669 | 0.442808364 | 0.446406234 | 0.450672284 | 0.455286055 | 0.460947666 | 0.467526738 | 0.475180193 | 0.483393694 | 0.491957984 | 0.500707708 | 0.509520302 | 0.51831286 | 0.527070553 | 0.53581192 | 0.542740356 | 0.549696015 |
| Seychelles | 0.575526496 | 0.582698331 | 0.590052919 | 0.598044194 | 0.606041268 | 0.613853281 | 0.62213517 | 0.630697732 | 0.638438882 | 0.645339666 | 0.651551988 | 0.656490668 | 0.66090779 | 0.664162027 | 0.666461353 | 0.669264742 | 0.672149914 | 0.675077438 | 0.676590946 | 0.677847028 | 0.68016364 | 0.683681491 | 0.687629394 | 0.692525734 | 0.697114589 | 0.70172282 | 0.706183623 | 0.711027281 | 0.716407819 | 0.722073685 | 0.726663769 | 0.730150775 |
| Sri Lanka | 0.522622553 | 0.528239203 | 0.533345535 | 0.538891102 | 0.545124551 | 0.551744058 | 0.558938888 | 0.566128422 | 0.572326187 | 0.577630048 | 0.582942638 | 0.587556599 | 0.592359127 | 0.597410284 | 0.602250062 | 0.606906656 | 0.611807083 | 0.616979265 | 0.622414317 | 0.62814963 | 0.634263123 | 0.640799571 | 0.648049816 | 0.655435838 | 0.662782434 | 0.669760292 | 0.676277441 | 0.682405274 | 0.688125858 | 0.693516063 | 0.697648535 | 0.701534935 |
| Thailand | 0.506644861 | 0.515911466 | 0.525161869 | 0.534303709 | 0.543325547 | 0.552452209 | 0.561302487 | 0.568449492 | 0.573817902 | 0.578854168 | 0.583500917 | 0.587661561 | 0.591759098 | 0.59604892 | 0.600520665 | 0.605040775 | 0.610356678 | 0.616368591 | 0.621067543 | 0.62432907 | 0.62858944 | 0.633226728 | 0.638410972 | 0.643672916 | 0.648772057 | 0.654015623 | 0.659369303 | 0.664804134 | 0.670225587 | 0.675273901 | 0.679119989 | 0.682547933 |
| Timor-Leste | 0.262468083 | 0.270943349 | 0.27991676 | 0.289266629 | 0.298763189 | 0.308018684 | 0.31758875 | 0.326705374 | 0.334894497 | 0.33925214 | 0.343793649 | 0.34949032 | 0.35416202 | 0.358440025 | 0.362687998 | 0.366913584 | 0.370654092 | 0.375390768 | 0.381227528 | 0.387944823 | 0.394887019 | 0.401872356 | 0.4089103 | 0.415217878 | 0.421155538 | 0.42638362 | 0.431207272 | 0.434519734 | 0.437018663 | 0.440195845 | 0.442349336 | 0.444667619 |
| Viet Nam | 0.407630048 | 0.41321725 | 0.419781657 | 0.427072185 | 0.435106272 | 0.443858785 | 0.45310241 | 0.462853518 | 0.472478892 | 0.481445774 | 0.489802045 | 0.497630349 | 0.505453447 | 0.513367511 | 0.52122939 | 0.528956332 | 0.536300653 | 0.543342652 | 0.550127119 | 0.556455944 | 0.562768571 | 0.568945458 | 0.575078672 | 0.581029215 | 0.586913815 | 0.592849573 | 0.598849485 | 0.605019421 | 0.611351406 | 0.617733815 | 0.62309032 | 0.627933721 |
| Sub-Saharan Africa | 0.297147968 | 0.30063245 | 0.303873882 | 0.306848923 | 0.309705038 | 0.312728678 | 0.316139229 | 0.319699114 | 0.323309973 | 0.327028574 | 0.330901959 | 0.334963282 | 0.339353466 | 0.344034375 | 0.349248417 | 0.355021813 | 0.361074742 | 0.367561611 | 0.374100635 | 0.380555193 | 0.387310016 | 0.394121758 | 0.40091964 | 0.407758129 | 0.414670486 | 0.421433007 | 0.427886948 | 0.43425414 | 0.440672802 | 0.44717187 | 0.45311872 | 0.458587301 |
| Central sub-Saharan Africa | 0.302374953 | 0.305220591 | 0.307803569 | 0.309082037 | 0.309897978 | 0.311059245 | 0.312674396 | 0.314501685 | 0.316506276 | 0.318568997 | 0.320841477 | 0.323749194 | 0.327566904 | 0.331934652 | 0.337420017 | 0.344099267 | 0.351459736 | 0.359565506 | 0.368488175 | 0.376669289 | 0.385380946 | 0.394691759 | 0.404233879 | 0.413522207 | 0.422578651 | 0.430987531 | 0.438745917 | 0.446178585 | 0.45324977 | 0.460159559 | 0.466456368 | 0.472255651 |
| Angola | 0.270736935 | 0.275160625 | 0.2792315 | 0.281867703 | 0.284487594 | 0.287447813 | 0.291421279 | 0.295433993 | 0.299553353 | 0.303720042 | 0.307603312 | 0.311697534 | 0.316706571 | 0.321777362 | 0.327395266 | 0.333785188 | 0.341012622 | 0.348846841 | 0.357082005 | 0.364407744 | 0.372033537 | 0.380006613 | 0.387891857 | 0.395796329 | 0.403720086 | 0.411372026 | 0.418822062 | 0.426177082 | 0.433355005 | 0.440454598 | 0.447283992 | 0.453721949 |
| Central African Republic | 0.216825191 | 0.220191656 | 0.223007541 | 0.225451125 | 0.228487724 | 0.231696792 | 0.233989591 | 0.236671999 | 0.239743529 | 0.24298853 | 0.246021132 | 0.24924674 | 0.252524369 | 0.255101519 | 0.257778778 | 0.260451918 | 0.263712446 | 0.267332195 | 0.271158118 | 0.275338515 | 0.279981382 | 0.285097245 | 0.290460023 | 0.29010493 | 0.289999498 | 0.290827871 | 0.2924304 | 0.294752243 | 0.298011223 | 0.301953064 | 0.305426298 | 0.30916769 |
| Congo (Brazzaville) | 0.420654655 | 0.426734374 | 0.431987662 | 0.436606594 | 0.440168347 | 0.443744125 | 0.447566642 | 0.451026961 | 0.454445045 | 0.45717654 | 0.460764814 | 0.463751682 | 0.467037268 | 0.470134374 | 0.473612436 | 0.478137627 | 0.483355822 | 0.48764074 | 0.492822855 | 0.498299969 | 0.505542849 | 0.513815777 | 0.522928283 | 0.531551679 | 0.540594486 | 0.54841403 | 0.555108608 | 0.5618341 | 0.56779397 | 0.573373665 | 0.578451546 | 0.583075236 |
| DR Congo | 0.28984321 | 0.290431567 | 0.290959242 | 0.28962879 | 0.286859146 | 0.283967532 | 0.28041316 | 0.276336503 | 0.271813823 | 0.267367662 | 0.262456747 | 0.257857505 | 0.25413636 | 0.252333885 | 0.252470919 | 0.254161023 | 0.257355123 | 0.262272267 | 0.268103872 | 0.274361144 | 0.282223754 | 0.290872953 | 0.300454783 | 0.310831256 | 0.321622927 | 0.332421307 | 0.341988787 | 0.351102035 | 0.360287677 | 0.369340684 | 0.376712271 | 0.383179849 |
| Equatorial Guinea | 0.268783629 | 0.275296992 | 0.283487764 | 0.292236032 | 0.30068634 | 0.310443551 | 0.324601221 | 0.350550963 | 0.371237163 | 0.391128023 | 0.41138345 | 0.433590057 | 0.451939 | 0.467846833 | 0.483879612 | 0.498683715 | 0.511579828 | 0.524076461 | 0.53701899 | 0.548514683 | 0.559109377 | 0.570277424 | 0.581833899 | 0.592800613 | 0.603473167 | 0.613134624 | 0.621858157 | 0.630234538 | 0.638022823 | 0.645303093 | 0.652124903 | 0.657857456 |
| Gabon | 0.455421187 | 0.461563098 | 0.467024048 | 0.472394463 | 0.478061947 | 0.483826218 | 0.48946146 | 0.495074162 | 0.50037255 | 0.50480635 | 0.509133087 | 0.513045709 | 0.516917621 | 0.521051412 | 0.525479071 | 0.530488575 | 0.534797716 | 0.539441424 | 0.544110892 | 0.548591319 | 0.554022706 | 0.560232082 | 0.566842964 | 0.57380813 | 0.580915656 | 0.588365 | 0.596062305 | 0.603816252 | 0.61172438 | 0.619904727 | 0.627609647 | 0.634691393 |
| Eastern sub-Saharan Africa | 0.233622123 | 0.236340461 | 0.238615291 | 0.240816973 | 0.242901814 | 0.245423542 | 0.24858486 | 0.252144544 | 0.255890509 | 0.259957122 | 0.264165312 | 0.26877226 | 0.273484445 | 0.278433675 | 0.283936614 | 0.28997039 | 0.296455257 | 0.30357591 | 0.310918495 | 0.318456773 | 0.326295972 | 0.334253401 | 0.341628076 | 0.3492073 | 0.35700952 | 0.364907041 | 0.372758832 | 0.380499991 | 0.388355112 | 0.396344656 | 0.403480212 | 0.409720983 |
| Burundi | 0.20586736 | 0.20849572 | 0.210864619 | 0.213249386 | 0.215222973 | 0.215631676 | 0.215526325 | 0.21576182 | 0.216402603 | 0.216855767 | 0.216869858 | 0.216988726 | 0.217782477 | 0.218961537 | 0.220918709 | 0.223084372 | 0.22582189 | 0.228996819 | 0.232853551 | 0.23694304 | 0.241447786 | 0.246277277 | 0.251572303 | 0.257191355 | 0.262794722 | 0.267327938 | 0.271587852 | 0.275840505 | 0.27973329 | 0.283442515 | 0.286496 | 0.289374365 |
| Comoros | 0.270048116 | 0.279145649 | 0.288506271 | 0.297607417 | 0.305706587 | 0.314220341 | 0.322249069 | 0.330148125 | 0.337517914 | 0.344774694 | 0.351875506 | 0.359211999 | 0.366486127 | 0.373410566 | 0.380035976 | 0.386891845 | 0.393521434 | 0.399051094 | 0.404649755 | 0.410253575 | 0.415543376 | 0.421092972 | 0.426729957 | 0.432789932 | 0.438844505 | 0.444712394 | 0.450506979 | 0.456280841 | 0.461951344 | 0.467388014 | 0.472003132 | 0.475978688 |
| Djibouti | 0.337781789 | 0.338770011 | 0.340986061 | 0.344904394 | 0.347981404 | 0.351245633 | 0.354385634 | 0.358096401 | 0.361596764 | 0.365305211 | 0.369015095 | 0.372853155 | 0.376650004 | 0.380686615 | 0.384987706 | 0.389568706 | 0.395037471 | 0.400618546 | 0.406658303 | 0.411969335 | 0.41699935 | 0.42231614 | 0.42771144 | 0.433360437 | 0.439455722 | 0.445855285 | 0.453442854 | 0.460440152 | 0.467705672 | 0.475263243 | 0.481879945 | 0.487958371 |
| Eritrea | 0.216028239 | 0.222574737 | 0.230296066 | 0.239385041 | 0.250752541 | 0.261498485 | 0.272527055 | 0.283551721 | 0.293764828 | 0.30314234 | 0.310248162 | 0.317247988 | 0.323566585 | 0.328593581 | 0.33287938 | 0.336682511 | 0.339835824 | 0.342843174 | 0.343427801 | 0.345147743 | 0.348146177 | 0.354163147 | 0.360112751 | 0.364343653 | 0.372527588 | 0.376935162 | 0.382171666 | 0.385936029 | 0.391154884 | 0.396330558 | 0.400459411 | 0.403863943 |
| Ethiopia | 0.148033885 | 0.14959971 | 0.150174386 | 0.151582364 | 0.152982637 | 0.155085605 | 0.158127229 | 0.161874743 | 0.16507771 | 0.168903545 | 0.17341853 | 0.178972969 | 0.184138013 | 0.188694814 | 0.195030701 | 0.202868243 | 0.211524229 | 0.221819931 | 0.232872186 | 0.244265818 | 0.25608146 | 0.267880862 | 0.279006958 | 0.289885267 | 0.300484548 | 0.310736419 | 0.320431867 | 0.329469244 | 0.338050075 | 0.346422664 | 0.35321635 | 0.358823295 |
| Addis Ababa | 0.487060041 | 0.495700289 | 0.502057376 | 0.508601978 | 0.514369793 | 0.520299676 | 0.526683615 | 0.533187064 | 0.538390746 | 0.543738777 | 0.549392679 | 0.555421611 | 0.560430451 | 0.56431784 | 0.569431506 | 0.575507797 | 0.581793407 | 0.58915693 | 0.596921141 | 0.604889655 | 0.61329088 | 0.621763342 | 0.629521789 | 0.637415929 | 0.645552442 | 0.653807554 | 0.66176964 | 0.669404029 | 0.67681666 | 0.684175853 | 0.689966686 | 0.694729596 |
| Afar | 0.102500069 | 0.102923831 | 0.102304887 | 0.102424632 | 0.102491546 | 0.103319651 | 0.105128034 | 0.107613013 | 0.109385397 | 0.111748498 | 0.11480189 | 0.119052377 | 0.12278243 | 0.125762457 | 0.130607715 | 0.136970023 | 0.14420382 | 0.153105163 | 0.162783668 | 0.172904437 | 0.183564452 | 0.194300556 | 0.204640198 | 0.214940642 | 0.225215268 | 0.235472535 | 0.245504833 | 0.255178611 | 0.26462211 | 0.273987062 | 0.281946443 | 0.28877341 |
| Amhara | 0.096349458 | 0.097325773 | 0.097289302 | 0.098146296 | 0.098993032 | 0.100681932 | 0.103542465 | 0.107321049 | 0.110521867 | 0.114484273 | 0.119321728 | 0.125539429 | 0.131277082 | 0.136190578 | 0.143249107 | 0.152135409 | 0.162029226 | 0.173820633 | 0.186399607 | 0.199275248 | 0.212603496 | 0.225784785 | 0.237985059 | 0.249617758 | 0.260904174 | 0.271720652 | 0.281870132 | 0.291171169 | 0.299934758 | 0.308401443 | 0.315058985 | 0.320453189 |
| Benishangul-Gumuz | 0.0929276 | 0.093242299 | 0.092373199 | 0.092442514 | 0.092501201 | 0.093497947 | 0.095699051 | 0.098780885 | 0.101087265 | 0.104172732 | 0.108181579 | 0.113636274 | 0.118492022 | 0.122465671 | 0.128820566 | 0.137216003 | 0.146907567 | 0.158848621 | 0.171841805 | 0.185382652 | 0.199532341 | 0.213470306 | 0.226435978 | 0.239210675 | 0.251668247 | 0.263781961 | 0.275302193 | 0.286020941 | 0.296111523 | 0.305808755 | 0.313705067 | 0.320228483 |
| Dire Dawa | 0.312205934 | 0.317408907 | 0.32136416 | 0.325969914 | 0.330416077 | 0.335292053 | 0.340748931 | 0.346489448 | 0.351424683 | 0.356676908 | 0.36228323 | 0.368284602 | 0.373457478 | 0.377791602 | 0.383401316 | 0.390137031 | 0.397525684 | 0.406219719 | 0.41557533 | 0.425320016 | 0.435564869 | 0.445847804 | 0.455620004 | 0.465686715 | 0.475958521 | 0.486344013 | 0.496518423 | 0.506387494 | 0.516014334 | 0.525567684 | 0.533667629 | 0.540668646 |
| Gambella | 0.168610253 | 0.17373969 | 0.177538991 | 0.18211209 | 0.186359835 | 0.190956687 | 0.196128721 | 0.201735096 | 0.206360155 | 0.211526595 | 0.217454831 | 0.224613524 | 0.231196338 | 0.23711931 | 0.245387358 | 0.255567796 | 0.266989876 | 0.280696131 | 0.295580104 | 0.311034768 | 0.327077215 | 0.342853488 | 0.357485609 | 0.371760261 | 0.385764224 | 0.39934786 | 0.41203614 | 0.423404495 | 0.433901339 | 0.443954165 | 0.451866699 | 0.458220084 |
| Harari | 0.322842077 | 0.327439623 | 0.330356931 | 0.333740959 | 0.336843341 | 0.340420403 | 0.344672091 | 0.349115288 | 0.352605147 | 0.356454396 | 0.360605372 | 0.365325293 | 0.369460465 | 0.372944404 | 0.377980109 | 0.384340196 | 0.391401543 | 0.399879548 | 0.409092228 | 0.418664883 | 0.428695065 | 0.438782762 | 0.448322656 | 0.458222131 | 0.468560236 | 0.479259712 | 0.49003538 | 0.500598889 | 0.51105614 | 0.521489099 | 0.530448765 | 0.538168759 |
| Oromia | 0.125544882 | 0.126509567 | 0.126441794 | 0.127249568 | 0.128025155 | 0.129534961 | 0.132016399 | 0.135189515 | 0.137689748 | 0.140860153 | 0.144768732 | 0.149866088 | 0.154533243 | 0.158564728 | 0.16460966 | 0.172315138 | 0.18092932 | 0.191360765 | 0.202633257 | 0.214279127 | 0.22638323 | 0.238588332 | 0.250290453 | 0.261907467 | 0.273269514 | 0.284185876 | 0.294551966 | 0.304229461 | 0.313416001 | 0.322347689 | 0.329693972 | 0.335815016 |
| Somali | 0.093590078 | 0.094118884 | 0.093758812 | 0.094108373 | 0.094380873 | 0.095260047 | 0.096930178 | 0.099120064 | 0.100596636 | 0.102567257 | 0.105173405 | 0.108761181 | 0.111945498 | 0.114508189 | 0.118730198 | 0.124399185 | 0.130917246 | 0.139047596 | 0.148040347 | 0.15757025 | 0.1676664 | 0.177880912 | 0.187800649 | 0.197750656 | 0.207670071 | 0.217529442 | 0.2270536 | 0.236208994 | 0.245143455 | 0.254025766 | 0.261652247 | 0.268253815 |
| Southern Nations, Nationalities, and Peoples | 0.132426296 | 0.13344635 | 0.133144737 | 0.133851279 | 0.134522203 | 0.136105734 | 0.138925428 | 0.14251578 | 0.145163238 | 0.148526634 | 0.152734813 | 0.158312815 | 0.163249936 | 0.167281943 | 0.173641111 | 0.181969304 | 0.191369588 | 0.202856322 | 0.21523697 | 0.228044157 | 0.24140239 | 0.254725107 | 0.267268804 | 0.279402183 | 0.291027273 | 0.302356072 | 0.313064228 | 0.32310623 | 0.332605836 | 0.341874098 | 0.349338891 | 0.355521531 |
| Tigray | 0.132771026 | 0.134682067 | 0.135957655 | 0.138249694 | 0.140704188 | 0.143957874 | 0.148141461 | 0.153040465 | 0.157343997 | 0.162224438 | 0.167870485 | 0.174690342 | 0.181381976 | 0.187632189 | 0.195772677 | 0.205581037 | 0.216403196 | 0.229047416 | 0.242563629 | 0.256376216 | 0.270216709 | 0.283424294 | 0.295751997 | 0.307762299 | 0.319430021 | 0.330726337 | 0.341332687 | 0.351135099 | 0.360330549 | 0.369232352 | 0.376346687 | 0.382183506 |
| Kenya | 0.333850293 | 0.34047622 | 0.346298636 | 0.351611769 | 0.356779194 | 0.361535807 | 0.366203076 | 0.370436465 | 0.374581401 | 0.378630009 | 0.382395616 | 0.386188415 | 0.389886256 | 0.39368048 | 0.397743651 | 0.402444011 | 0.407745764 | 0.413736227 | 0.419738147 | 0.426123888 | 0.433305839 | 0.44089383 | 0.448666396 | 0.456666942 | 0.464724302 | 0.473084051 | 0.481709072 | 0.490296619 | 0.499101274 | 0.508004211 | 0.516136572 | 0.523768077 |
| Baringo | 0.277332089 | 0.286878009 | 0.295822039 | 0.304372219 | 0.312974314 | 0.321200334 | 0.32941504 | 0.337292157 | 0.345077278 | 0.352742706 | 0.360261775 | 0.367707196 | 0.374960988 | 0.382107317 | 0.388951441 | 0.395877128 | 0.402848854 | 0.409944037 | 0.416535429 | 0.423225848 | 0.43042939 | 0.437778253 | 0.445237466 | 0.452865588 | 0.460460642 | 0.468325633 | 0.476370043 | 0.484323262 | 0.492424587 | 0.50052297 | 0.507836674 | 0.514883215 |
| Bomet | 0.281945483 | 0.288978204 | 0.295293783 | 0.301345026 | 0.30755692 | 0.313625026 | 0.319692092 | 0.325478983 | 0.331270626 | 0.337193122 | 0.343233239 | 0.34934892 | 0.355463063 | 0.361771481 | 0.368343616 | 0.375589087 | 0.383283932 | 0.391590403 | 0.400008587 | 0.408854098 | 0.418375624 | 0.428141421 | 0.438158248 | 0.44836723 | 0.458639244 | 0.469145108 | 0.479687117 | 0.490006826 | 0.500382824 | 0.510720637 | 0.520366458 | 0.529367852 |
| Bungoma | 0.289281609 | 0.296687682 | 0.303245026 | 0.30916962 | 0.314806558 | 0.319902117 | 0.324822574 | 0.329336651 | 0.333755213 | 0.338064073 | 0.342016059 | 0.345968295 | 0.34983724 | 0.353888788 | 0.35830797 | 0.363524204 | 0.369410196 | 0.375931366 | 0.382377831 | 0.389285339 | 0.397130174 | 0.405447362 | 0.413940241 | 0.422750804 | 0.431674064 | 0.440819654 | 0.450271865 | 0.459783883 | 0.469541512 | 0.479335024 | 0.488234119 | 0.49672518 |
| Busia | 0.229493355 | 0.237501538 | 0.245283572 | 0.253022094 | 0.260764321 | 0.268296617 | 0.275677969 | 0.282650691 | 0.289517931 | 0.296358333 | 0.302781508 | 0.309049166 | 0.315209718 | 0.321285116 | 0.327401014 | 0.334060545 | 0.341133856 | 0.348761092 | 0.356235821 | 0.364040116 | 0.372583965 | 0.381401685 | 0.390345183 | 0.39962196 | 0.408933968 | 0.418526513 | 0.428425466 | 0.438368158 | 0.448565801 | 0.458849495 | 0.468461616 | 0.477665024 |
| Elgeyo Marakwet | 0.272798674 | 0.282228145 | 0.290881506 | 0.2990872 | 0.307222665 | 0.315020211 | 0.322700147 | 0.330033053 | 0.337356882 | 0.344705996 | 0.35191274 | 0.359033124 | 0.36593207 | 0.372766811 | 0.379711403 | 0.387195874 | 0.395105718 | 0.403584417 | 0.411849691 | 0.42018957 | 0.429153069 | 0.438291986 | 0.447456591 | 0.456657405 | 0.465752982 | 0.47495817 | 0.484138762 | 0.493042173 | 0.501926817 | 0.510784334 | 0.518801071 | 0.526297038 |
| Embu | 0.372525502 | 0.378069856 | 0.382859621 | 0.387355614 | 0.39192078 | 0.396238366 | 0.400489568 | 0.404374282 | 0.408165465 | 0.411846168 | 0.415289031 | 0.418849672 | 0.422355511 | 0.425953065 | 0.429822652 | 0.434227692 | 0.439125403 | 0.444629822 | 0.450094125 | 0.456055145 | 0.462764907 | 0.469904216 | 0.477134665 | 0.484607328 | 0.49219877 | 0.500061338 | 0.508151156 | 0.516152044 | 0.524376201 | 0.532682663 | 0.540298622 | 0.547555396 |
| Garissa | 0.158613685 | 0.162075617 | 0.165442493 | 0.16880049 | 0.172383212 | 0.176036287 | 0.179871369 | 0.183679778 | 0.187553513 | 0.191494598 | 0.195299262 | 0.199359527 | 0.203508874 | 0.2078281 | 0.212454999 | 0.21753118 | 0.223143725 | 0.229296059 | 0.235495105 | 0.241870215 | 0.248733662 | 0.255820271 | 0.262892366 | 0.26980041 | 0.276506866 | 0.283180026 | 0.289790818 | 0.296197167 | 0.30269397 | 0.309230819 | 0.315298423 | 0.321068269 |
| Homa Bay | 0.231035423 | 0.238208688 | 0.24496969 | 0.25161778 | 0.257939844 | 0.263911807 | 0.269616851 | 0.274818633 | 0.280234259 | 0.285549547 | 0.290594767 | 0.295540618 | 0.300173084 | 0.304977796 | 0.310242515 | 0.316452444 | 0.323426222 | 0.331350321 | 0.33957262 | 0.348645248 | 0.358791068 | 0.369579889 | 0.380947133 | 0.392937298 | 0.405141057 | 0.417770862 | 0.430745861 | 0.443755873 | 0.45697261 | 0.47018344 | 0.482598866 | 0.493871805 |
| Isiolo | 0.233789194 | 0.240136919 | 0.246029388 | 0.251700393 | 0.257510266 | 0.263085837 | 0.268767424 | 0.27424321 | 0.279685288 | 0.28506706 | 0.290220404 | 0.295382533 | 0.300447525 | 0.305535926 | 0.310498022 | 0.315713545 | 0.32120826 | 0.326862117 | 0.332165118 | 0.337915166 | 0.344500056 | 0.351700619 | 0.359069959 | 0.366859879 | 0.374739343 | 0.382963361 | 0.391580259 | 0.400181297 | 0.408988824 | 0.417901897 | 0.426126664 | 0.433966834 |
| Kajiado | 0.299327765 | 0.306299633 | 0.312299085 | 0.317767754 | 0.323185612 | 0.328127219 | 0.333007381 | 0.337434417 | 0.341698631 | 0.34578703 | 0.349825143 | 0.353880047 | 0.357912869 | 0.362013372 | 0.366345635 | 0.371497585 | 0.377256509 | 0.383766446 | 0.390516285 | 0.397714438 | 0.405629344 | 0.413841765 | 0.422233279 | 0.43102159 | 0.439981356 | 0.449339571 | 0.458985237 | 0.468617119 | 0.478439686 | 0.488406492 | 0.498000224 | 0.506969707 |
| Kakamega | 0.309166492 | 0.316082197 | 0.322142025 | 0.327535645 | 0.332549378 | 0.3369821 | 0.341248814 | 0.345135014 | 0.349037071 | 0.352837825 | 0.356379085 | 0.359825129 | 0.363104932 | 0.366433281 | 0.37019984 | 0.374744991 | 0.380093897 | 0.386406713 | 0.392940998 | 0.400074524 | 0.408217186 | 0.416881595 | 0.42572593 | 0.434921395 | 0.444211203 | 0.4536624 | 0.463300914 | 0.472835017 | 0.482470283 | 0.49208871 | 0.501019618 | 0.509528998 |
| Kericho | 0.30207479 | 0.308864481 | 0.314698953 | 0.319936672 | 0.325052651 | 0.329781843 | 0.334690188 | 0.339360909 | 0.344219039 | 0.349340188 | 0.354597672 | 0.360152146 | 0.365873458 | 0.371789278 | 0.377842111 | 0.384458326 | 0.391394429 | 0.398860435 | 0.4063073 | 0.414126682 | 0.422687087 | 0.431516341 | 0.440470989 | 0.449535518 | 0.458721708 | 0.468135707 | 0.477679959 | 0.487050258 | 0.496501863 | 0.506033096 | 0.515079954 | 0.523573355 |
| Kiambu | 0.410469356 | 0.417320566 | 0.422914458 | 0.427542947 | 0.43178245 | 0.435489619 | 0.439143408 | 0.442421965 | 0.445771814 | 0.449176001 | 0.452421301 | 0.455733973 | 0.459027239 | 0.462606469 | 0.466675515 | 0.471554086 | 0.477151256 | 0.483528287 | 0.489983171 | 0.496749665 | 0.504244383 | 0.512064532 | 0.519867024 | 0.527817485 | 0.535710548 | 0.543912998 | 0.552377157 | 0.560748533 | 0.569340941 | 0.577987173 | 0.585786674 | 0.593110152 |
| Kilifi | 0.258065918 | 0.264638143 | 0.270643202 | 0.276129217 | 0.281366995 | 0.286267669 | 0.29116174 | 0.295678144 | 0.299911897 | 0.303960339 | 0.307761036 | 0.311443861 | 0.315129611 | 0.318793262 | 0.322605859 | 0.327218625 | 0.332726436 | 0.339290434 | 0.34646581 | 0.354275828 | 0.363203005 | 0.372821703 | 0.382981435 | 0.393550546 | 0.404422836 | 0.415841851 | 0.427606972 | 0.439435626 | 0.45138702 | 0.463434679 | 0.47510409 | 0.486009157 |
| Kirinyaga | 0.406480966 | 0.412135271 | 0.416486334 | 0.419920108 | 0.42311659 | 0.425837437 | 0.428359335 | 0.430413807 | 0.432451874 | 0.434515702 | 0.436364372 | 0.43832667 | 0.440202209 | 0.442203735 | 0.444358491 | 0.447026088 | 0.450300341 | 0.454472528 | 0.458668357 | 0.463317566 | 0.46882596 | 0.474695993 | 0.480662689 | 0.486893647 | 0.493236407 | 0.500055614 | 0.50722261 | 0.514382136 | 0.521785964 | 0.529359034 | 0.536317159 | 0.54301445 |
| Kisii | 0.325030301 | 0.333612106 | 0.341175248 | 0.347928262 | 0.354020159 | 0.359278054 | 0.36421181 | 0.368447746 | 0.372632803 | 0.376657104 | 0.380373225 | 0.384251535 | 0.388031719 | 0.391918467 | 0.396308016 | 0.4015104 | 0.40755891 | 0.414508057 | 0.421670822 | 0.429476715 | 0.438345733 | 0.447801417 | 0.457541497 | 0.467455973 | 0.477478633 | 0.487528354 | 0.497703186 | 0.507600741 | 0.517532116 | 0.527325355 | 0.536295845 | 0.544604844 |
| Kisumu | 0.306497721 | 0.316416331 | 0.325436172 | 0.333735869 | 0.341396111 | 0.348410666 | 0.354855748 | 0.360556843 | 0.366052267 | 0.371220317 | 0.375907965 | 0.380341979 | 0.384541787 | 0.388736614 | 0.39336326 | 0.398836634 | 0.405172705 | 0.412555961 | 0.420268994 | 0.428450439 | 0.437464315 | 0.446871546 | 0.456455132 | 0.466145655 | 0.475832211 | 0.48576018 | 0.495937228 | 0.505989773 | 0.516171715 | 0.526381884 | 0.535757398 | 0.544409324 |
| Kitui | 0.281505982 | 0.287870185 | 0.293429062 | 0.298578876 | 0.303634717 | 0.30832271 | 0.312815604 | 0.316995208 | 0.321138962 | 0.325151708 | 0.328951755 | 0.332601765 | 0.336296515 | 0.340142044 | 0.344332065 | 0.349270587 | 0.354726026 | 0.360776397 | 0.366758133 | 0.373247733 | 0.380674122 | 0.388582679 | 0.396668311 | 0.405106812 | 0.413650846 | 0.422448299 | 0.431423868 | 0.440274725 | 0.449241901 | 0.458276183 | 0.466731847 | 0.474749457 |
| Kwale | 0.27463023 | 0.281139161 | 0.287040655 | 0.29245678 | 0.297643945 | 0.302320604 | 0.306849191 | 0.310865037 | 0.314636206 | 0.318185739 | 0.321342951 | 0.324351351 | 0.327189853 | 0.330069975 | 0.333107885 | 0.336671097 | 0.341021175 | 0.346369927 | 0.352155796 | 0.358545056 | 0.36598907 | 0.374187841 | 0.383000519 | 0.392343863 | 0.40200224 | 0.412208077 | 0.422763988 | 0.433370974 | 0.444029269 | 0.454781139 | 0.465209628 | 0.475138205 |
| Laikipia | 0.375311824 | 0.380289937 | 0.384354573 | 0.387921611 | 0.391472315 | 0.394764889 | 0.39812225 | 0.401319836 | 0.404635168 | 0.408182913 | 0.411893892 | 0.416034175 | 0.420507853 | 0.42545425 | 0.430872427 | 0.437148409 | 0.444119746 | 0.451859318 | 0.459837445 | 0.467968761 | 0.476552844 | 0.4851985 | 0.493941841 | 0.502791734 | 0.511654309 | 0.520793122 | 0.530152159 | 0.539393118 | 0.548786528 | 0.558275906 | 0.567201834 | 0.575711773 |
| Lamu | 0.308688861 | 0.315858991 | 0.32230194 | 0.328268032 | 0.334149911 | 0.339731428 | 0.345165422 | 0.350113364 | 0.354774515 | 0.359235424 | 0.363358443 | 0.367410061 | 0.371418263 | 0.375381636 | 0.379415547 | 0.38391899 | 0.389050975 | 0.394790259 | 0.400513063 | 0.406531449 | 0.413339276 | 0.420574444 | 0.428081826 | 0.435825732 | 0.443617821 | 0.451918618 | 0.460543247 | 0.469210157 | 0.478160964 | 0.48724627 | 0.495694014 | 0.503786492 |
| Machakos | 0.36059665 | 0.366629546 | 0.371698098 | 0.376123795 | 0.380260963 | 0.383958354 | 0.387674486 | 0.391111035 | 0.394660229 | 0.398261804 | 0.40165728 | 0.405310031 | 0.409086028 | 0.41314001 | 0.417533813 | 0.422542729 | 0.428287973 | 0.434700396 | 0.441017691 | 0.4477737 | 0.455299744 | 0.463288309 | 0.471389246 | 0.479677659 | 0.48792392 | 0.496444296 | 0.505176926 | 0.513872648 | 0.522789377 | 0.531798647 | 0.540095767 | 0.548035791 |
| Makueni | 0.295311685 | 0.302407578 | 0.308792118 | 0.31480265 | 0.320786566 | 0.326346147 | 0.331794835 | 0.336949064 | 0.342080128 | 0.347085149 | 0.351859861 | 0.356568011 | 0.361233362 | 0.366074084 | 0.37123528 | 0.377172366 | 0.383717508 | 0.390929628 | 0.398042476 | 0.405572841 | 0.41400964 | 0.422829745 | 0.431652015 | 0.44070173 | 0.449679287 | 0.458863902 | 0.468196269 | 0.477358962 | 0.486661665 | 0.49599583 | 0.504472463 | 0.512568943 |
| Mandera | 0.112796963 | 0.114710494 | 0.116499203 | 0.118206025 | 0.120081361 | 0.12206423 | 0.124250297 | 0.126571695 | 0.129153551 | 0.131879035 | 0.134605482 | 0.137509405 | 0.140702984 | 0.144161797 | 0.147937495 | 0.152004908 | 0.1565108 | 0.161530153 | 0.166690305 | 0.172206179 | 0.178114605 | 0.184125515 | 0.190029172 | 0.195826739 | 0.201622992 | 0.207349812 | 0.212886209 | 0.218179501 | 0.223415981 | 0.228539303 | 0.233436435 | 0.238263833 |
| Marsabit | 0.204167587 | 0.209181919 | 0.213861832 | 0.218392609 | 0.223077631 | 0.22765943 | 0.232422125 | 0.237073522 | 0.241817875 | 0.246536497 | 0.251064163 | 0.255583027 | 0.260140919 | 0.26479248 | 0.26959364 | 0.274875335 | 0.280605714 | 0.286732294 | 0.292798614 | 0.299365799 | 0.306712857 | 0.31457732 | 0.322558321 | 0.330864084 | 0.339225321 | 0.347859627 | 0.356777307 | 0.36561218 | 0.374571198 | 0.383605176 | 0.392079792 | 0.40018158 |
| Meru | 0.313881707 | 0.32028248 | 0.325855052 | 0.330852571 | 0.335717435 | 0.340262064 | 0.344674977 | 0.348596344 | 0.35240216 | 0.356104582 | 0.359590253 | 0.363165461 | 0.366724329 | 0.37051689 | 0.374748109 | 0.379640802 | 0.385187952 | 0.391474743 | 0.397812871 | 0.404557718 | 0.412075525 | 0.420021367 | 0.428126919 | 0.436454207 | 0.44493826 | 0.453822302 | 0.462978584 | 0.472065845 | 0.481364094 | 0.490800988 | 0.499716545 | 0.508216146 |
| Migori | 0.199865536 | 0.205883867 | 0.211993161 | 0.218439856 | 0.224884551 | 0.231356707 | 0.237868705 | 0.244064712 | 0.25054333 | 0.257141704 | 0.2635732 | 0.269829455 | 0.275905778 | 0.28214766 | 0.288653586 | 0.295858315 | 0.303462984 | 0.311744219 | 0.320310536 | 0.329467455 | 0.339420965 | 0.349834499 | 0.360781185 | 0.372138347 | 0.383672534 | 0.395711053 | 0.408391955 | 0.421276454 | 0.434433861 | 0.447766446 | 0.460788655 | 0.472803008 |
| Mombasa | 0.407873141 | 0.413952754 | 0.419216297 | 0.423935689 | 0.428567486 | 0.43261654 | 0.436912702 | 0.440749575 | 0.444374099 | 0.447876932 | 0.451005238 | 0.454429169 | 0.457706311 | 0.460991664 | 0.46472932 | 0.469264633 | 0.474868238 | 0.481588932 | 0.488455042 | 0.495652365 | 0.503643943 | 0.512137613 | 0.52080746 | 0.529644747 | 0.538310938 | 0.547058588 | 0.555882456 | 0.564405916 | 0.573022149 | 0.581578726 | 0.589168589 | 0.596387778 |
| Murang'a | 0.367814565 | 0.374654381 | 0.380424549 | 0.385632142 | 0.390711094 | 0.395411575 | 0.399939801 | 0.404054967 | 0.408210411 | 0.412402098 | 0.416382651 | 0.420324202 | 0.424209256 | 0.428188367 | 0.432384454 | 0.437063013 | 0.442209755 | 0.447999783 | 0.453714952 | 0.45978679 | 0.46661773 | 0.473781755 | 0.480960349 | 0.488301742 | 0.495637387 | 0.503337788 | 0.511324568 | 0.519302519 | 0.527564491 | 0.535945494 | 0.543577884 | 0.550869493 |
| Nairobi | 0.522778723 | 0.527295829 | 0.530936286 | 0.534057641 | 0.537299975 | 0.540339994 | 0.543621476 | 0.546664333 | 0.549828568 | 0.553306654 | 0.556745068 | 0.560661747 | 0.564589615 | 0.568691475 | 0.573026948 | 0.57790573 | 0.583405742 | 0.589569067 | 0.595541389 | 0.601634382 | 0.608372694 | 0.615332314 | 0.622189608 | 0.628967972 | 0.635532396 | 0.642338463 | 0.649423351 | 0.656440352 | 0.663703077 | 0.670998932 | 0.677501788 | 0.683722442 |
| Nakuru | 0.355830725 | 0.362978684 | 0.36906454 | 0.374535834 | 0.379914658 | 0.384788103 | 0.38972562 | 0.394251801 | 0.398727016 | 0.403222967 | 0.407623742 | 0.412321693 | 0.416933249 | 0.421632692 | 0.426993532 | 0.433254188 | 0.440546639 | 0.448794476 | 0.456991475 | 0.465386757 | 0.474400378 | 0.483665936 | 0.492934772 | 0.502193428 | 0.511251276 | 0.520285373 | 0.529299163 | 0.537925344 | 0.546589463 | 0.555160729 | 0.562796611 | 0.570045398 |
| Nandi | 0.289951784 | 0.298532129 | 0.30628018 | 0.313513689 | 0.320512487 | 0.327070198 | 0.333285169 | 0.338996734 | 0.344421211 | 0.349670131 | 0.354739743 | 0.359652111 | 0.364505965 | 0.369245457 | 0.374084167 | 0.379437954 | 0.385182092 | 0.391734704 | 0.398540012 | 0.405815716 | 0.413796564 | 0.422098935 | 0.430727868 | 0.439713523 | 0.448957286 | 0.458578113 | 0.468336876 | 0.477969672 | 0.487743539 | 0.497687131 | 0.507258223 | 0.516279712 |
| Narok | 0.213001059 | 0.219832858 | 0.226263942 | 0.232615568 | 0.23909751 | 0.245397357 | 0.251663101 | 0.257634254 | 0.263614734 | 0.269653794 | 0.27584801 | 0.282057247 | 0.288409006 | 0.294785307 | 0.30108875 | 0.30787153 | 0.314857395 | 0.322286006 | 0.32995789 | 0.338148229 | 0.347074293 | 0.356334806 | 0.365864874 | 0.375655608 | 0.385741372 | 0.396188479 | 0.406797334 | 0.417373403 | 0.428054525 | 0.438828383 | 0.449296153 | 0.459103072 |
| Nyamira | 0.353838595 | 0.361933874 | 0.369320218 | 0.376132473 | 0.382747587 | 0.388888308 | 0.394719451 | 0.399922482 | 0.40508713 | 0.410234682 | 0.415304091 | 0.420384016 | 0.425318272 | 0.430489229 | 0.436138743 | 0.442491015 | 0.449334107 | 0.456935904 | 0.46445611 | 0.47263268 | 0.481833522 | 0.491467201 | 0.501387427 | 0.511585925 | 0.521899691 | 0.532275552 | 0.54259666 | 0.552578759 | 0.562517271 | 0.57232096 | 0.581184672 | 0.589267078 |
| Nyandarua | 0.374020323 | 0.380029956 | 0.384965144 | 0.389294058 | 0.393686702 | 0.397903926 | 0.402347268 | 0.406838521 | 0.411643022 | 0.416676136 | 0.421717214 | 0.427074801 | 0.432500562 | 0.438119612 | 0.443889201 | 0.450134067 | 0.457033482 | 0.464498591 | 0.47175062 | 0.479239491 | 0.487250516 | 0.49545696 | 0.503553184 | 0.511735627 | 0.519831657 | 0.528155728 | 0.536570319 | 0.544789658 | 0.553108108 | 0.561413375 | 0.568909286 | 0.576109863 |
| Nyeri | 0.409157218 | 0.41528834 | 0.420285295 | 0.424671172 | 0.428909275 | 0.432821312 | 0.436575649 | 0.439918068 | 0.44321536 | 0.446406337 | 0.449352175 | 0.452467085 | 0.455543378 | 0.458713212 | 0.462393125 | 0.466940894 | 0.472298801 | 0.478607571 | 0.484907529 | 0.491468442 | 0.498737534 | 0.506238988 | 0.513639009 | 0.521075477 | 0.528420877 | 0.535895217 | 0.543448659 | 0.550775456 | 0.558279902 | 0.565802897 | 0.572430949 | 0.578787484 |
| Samburu | 0.203841145 | 0.208472034 | 0.212419411 | 0.216068502 | 0.219785184 | 0.223259402 | 0.226828881 | 0.230249271 | 0.233745767 | 0.237228318 | 0.240700627 | 0.24433472 | 0.247983846 | 0.251693338 | 0.255598798 | 0.260053987 | 0.264927889 | 0.27031853 | 0.275773699 | 0.281672668 | 0.288237065 | 0.295159731 | 0.302378771 | 0.309885983 | 0.317590346 | 0.325839838 | 0.334464904 | 0.343087203 | 0.35205085 | 0.361237702 | 0.370004484 | 0.378312395 |
| Siaya | 0.229809712 | 0.236939692 | 0.243607305 | 0.250120001 | 0.25651642 | 0.262582972 | 0.26846617 | 0.273755658 | 0.279084929 | 0.284349415 | 0.28932821 | 0.29424968 | 0.298974728 | 0.303832567 | 0.309184225 | 0.315460858 | 0.322478345 | 0.330290953 | 0.338246302 | 0.346839505 | 0.35642434 | 0.366632691 | 0.377254141 | 0.388136749 | 0.39915091 | 0.410297166 | 0.421719141 | 0.433062263 | 0.444516822 | 0.455912354 | 0.466632292 | 0.476524395 |
| Taita Taveta | 0.343602499 | 0.349733192 | 0.355083819 | 0.359999178 | 0.364840536 | 0.369378824 | 0.374013622 | 0.378267827 | 0.382338517 | 0.386231377 | 0.389798972 | 0.3935252 | 0.397215975 | 0.400920752 | 0.404927037 | 0.409592214 | 0.415158212 | 0.421705986 | 0.428400659 | 0.435441981 | 0.443333362 | 0.451730134 | 0.460397496 | 0.469267546 | 0.47814447 | 0.487264416 | 0.496562093 | 0.505716641 | 0.514986961 | 0.524269243 | 0.532768711 | 0.540980982 |
| Tana River | 0.231789736 | 0.236304822 | 0.240055184 | 0.24317251 | 0.246068031 | 0.248472631 | 0.250663384 | 0.252335202 | 0.253672818 | 0.254820981 | 0.255689154 | 0.256498305 | 0.257284349 | 0.258056216 | 0.259220653 | 0.261261307 | 0.264326045 | 0.268385922 | 0.27277766 | 0.277835521 | 0.284083465 | 0.291098798 | 0.29868733 | 0.306908499 | 0.315400595 | 0.324503219 | 0.334024478 | 0.34367519 | 0.353528431 | 0.363559442 | 0.373453015 | 0.382845346 |
| Tharaka Nithi | 0.311294385 | 0.318161516 | 0.324406452 | 0.330220761 | 0.336010206 | 0.341489728 | 0.346834873 | 0.351980976 | 0.357078214 | 0.362093357 | 0.366891834 | 0.371626476 | 0.37651194 | 0.381639324 | 0.387082792 | 0.393240832 | 0.400031862 | 0.407434404 | 0.414820355 | 0.42269232 | 0.431431702 | 0.440456761 | 0.449555707 | 0.458782033 | 0.467930687 | 0.477151694 | 0.486385134 | 0.495395098 | 0.504444614 | 0.513492735 | 0.521789972 | 0.529680112 |
| Trans Nzoia | 0.326157148 | 0.334040233 | 0.341036141 | 0.347356539 | 0.353497308 | 0.359018313 | 0.364477075 | 0.369464613 | 0.374460727 | 0.379496401 | 0.384420503 | 0.38957093 | 0.39475898 | 0.400220196 | 0.405896966 | 0.411986738 | 0.418460722 | 0.425549179 | 0.432679349 | 0.440110338 | 0.448184709 | 0.456627428 | 0.46527019 | 0.474087026 | 0.483016789 | 0.492316277 | 0.501899767 | 0.51136886 | 0.521046626 | 0.53079554 | 0.539869686 | 0.54850455 |
| Turkana | 0.17963154 | 0.184595301 | 0.189320069 | 0.194012228 | 0.198911833 | 0.203689386 | 0.208625628 | 0.213444575 | 0.218359032 | 0.223219564 | 0.22786553 | 0.232581778 | 0.237288461 | 0.24207306 | 0.246848011 | 0.251951295 | 0.257322388 | 0.263121618 | 0.268957893 | 0.275347963 | 0.282535371 | 0.290188798 | 0.298098165 | 0.306283306 | 0.314715435 | 0.323414647 | 0.332280261 | 0.340970367 | 0.349581677 | 0.357978624 | 0.365914713 | 0.373434245 |
| Uasin Gishu | 0.351121368 | 0.358988709 | 0.365838113 | 0.371976508 | 0.378017822 | 0.38359007 | 0.389104903 | 0.394281318 | 0.399377787 | 0.404554879 | 0.409643792 | 0.414851272 | 0.420143704 | 0.425628597 | 0.431295437 | 0.43744848 | 0.444005763 | 0.451033801 | 0.457933808 | 0.465146264 | 0.472964516 | 0.48106746 | 0.48936919 | 0.497840532 | 0.506386415 | 0.515174206 | 0.524117776 | 0.532968344 | 0.541952861 | 0.550969586 | 0.559345775 | 0.567345589 |
| Vihiga | 0.292847673 | 0.300805202 | 0.308182414 | 0.315385132 | 0.322616471 | 0.329540915 | 0.336348736 | 0.342660257 | 0.348901362 | 0.355161749 | 0.361181597 | 0.367230143 | 0.373082587 | 0.378844105 | 0.384682484 | 0.391126762 | 0.398066683 | 0.405598107 | 0.412821795 | 0.420276752 | 0.428488618 | 0.436932985 | 0.445452065 | 0.454147368 | 0.462687227 | 0.471450792 | 0.480428633 | 0.489259117 | 0.498209093 | 0.50720557 | 0.515325056 | 0.523089788 |
| Wajir | 0.114632781 | 0.117329999 | 0.120087561 | 0.122832208 | 0.125758988 | 0.128681816 | 0.131747473 | 0.134866156 | 0.138041871 | 0.141228778 | 0.144359057 | 0.147636667 | 0.151065872 | 0.154658264 | 0.158474886 | 0.162597059 | 0.16714514 | 0.172108369 | 0.177130107 | 0.182440532 | 0.188210003 | 0.194272432 | 0.20030946 | 0.206341445 | 0.212296473 | 0.218211943 | 0.224072098 | 0.229761026 | 0.23551673 | 0.241298242 | 0.246768022 | 0.252050106 |
| West Pokot | 0.215643183 | 0.22239318 | 0.228657488 | 0.234625908 | 0.240556788 | 0.24610384 | 0.251641623 | 0.256799193 | 0.261983743 | 0.267220236 | 0.272461513 | 0.277802921 | 0.283210973 | 0.288805767 | 0.294635927 | 0.301135742 | 0.307981464 | 0.315363154 | 0.322748175 | 0.330513501 | 0.338966876 | 0.347829012 | 0.357062523 | 0.366574394 | 0.376245514 | 0.386194704 | 0.396256392 | 0.406139523 | 0.416058663 | 0.426002684 | 0.435325732 | 0.444120899 |
| Madagascar | 0.279889465 | 0.280429589 | 0.280501283 | 0.280204299 | 0.279615149 | 0.279164003 | 0.279066493 | 0.27942619 | 0.280317671 | 0.281571072 | 0.283190721 | 0.28566818 | 0.287153149 | 0.289675457 | 0.292629885 | 0.295919327 | 0.299723392 | 0.304026245 | 0.308990265 | 0.313450891 | 0.318353184 | 0.323801043 | 0.329947541 | 0.336519232 | 0.343352611 | 0.350443595 | 0.358026341 | 0.366235587 | 0.374969644 | 0.384209196 | 0.392778709 | 0.400246943 |
| Malawi | 0.204010245 | 0.206043786 | 0.206732359 | 0.208091205 | 0.209144566 | 0.211756232 | 0.215264475 | 0.219416252 | 0.22312233 | 0.227068368 | 0.23090081 | 0.234020507 | 0.237602155 | 0.241559228 | 0.24643454 | 0.251672318 | 0.257595649 | 0.265226928 | 0.273689856 | 0.282296509 | 0.29117962 | 0.300448509 | 0.309197624 | 0.317936213 | 0.327042426 | 0.336140138 | 0.344731542 | 0.353121067 | 0.36147041 | 0.370114605 | 0.37787902 | 0.384553634 |
| Mozambique | 0.173064715 | 0.175550435 | 0.177285188 | 0.179851807 | 0.182236861 | 0.183427982 | 0.185705869 | 0.188650436 | 0.191998629 | 0.195435288 | 0.198534397 | 0.202241924 | 0.20639006 | 0.21053449 | 0.214972457 | 0.219692372 | 0.224758087 | 0.230158227 | 0.23579017 | 0.241360656 | 0.2467515 | 0.252232471 | 0.258315562 | 0.265280462 | 0.272892814 | 0.281032685 | 0.289152425 | 0.297164772 | 0.305226772 | 0.313174508 | 0.320291182 | 0.326462614 |
| Rwanda | 0.27509719 | 0.276194698 | 0.277178171 | 0.276980133 | 0.272331258 | 0.271102227 | 0.271097922 | 0.272455861 | 0.274582551 | 0.277014387 | 0.280983454 | 0.286082055 | 0.29295543 | 0.299676918 | 0.306943916 | 0.314219578 | 0.321998519 | 0.329616591 | 0.337039226 | 0.344494633 | 0.351659416 | 0.35894034 | 0.366411821 | 0.373769682 | 0.381533179 | 0.389895415 | 0.398287959 | 0.406411475 | 0.414657912 | 0.423086727 | 0.429896596 | 0.435588706 |
| Somalia | 0.048848564 | 0.049422382 | 0.050115531 | 0.050797999 | 0.051471696 | 0.052148606 | 0.052840548 | 0.053570967 | 0.054321527 | 0.055071838 | 0.055842063 | 0.056643165 | 0.057457612 | 0.05827215 | 0.059076945 | 0.059898539 | 0.060741484 | 0.061598151 | 0.062475387 | 0.063382225 | 0.064322128 | 0.065306811 | 0.066354879 | 0.067459486 | 0.068609299 | 0.069811521 | 0.071064047 | 0.072334776 | 0.073642248 | 0.074979811 | 0.07636337 | 0.077688109 |
| South Sudan | 0.2066565 | 0.208932716 | 0.211199401 | 0.213416471 | 0.215612089 | 0.217920463 | 0.220399437 | 0.223037171 | 0.225788869 | 0.228648585 | 0.231730344 | 0.235014128 | 0.238341186 | 0.241797191 | 0.245462499 | 0.249280904 | 0.253300508 | 0.257491764 | 0.261740458 | 0.266179235 | 0.270572622 | 0.274719143 | 0.275066143 | 0.275813942 | 0.276311461 | 0.275833634 | 0.274455491 | 0.274387859 | 0.274987848 | 0.275706201 | 0.276787924 | 0.278371125 |
| Uganda | 0.187001096 | 0.188924156 | 0.191003433 | 0.193557456 | 0.197293193 | 0.201835423 | 0.207080731 | 0.213091696 | 0.219712442 | 0.226916945 | 0.234323225 | 0.242186388 | 0.250552388 | 0.259164883 | 0.268004611 | 0.277369193 | 0.287030583 | 0.29674042 | 0.306748843 | 0.316731153 | 0.326543661 | 0.336217716 | 0.345364515 | 0.354381615 | 0.363430903 | 0.372537986 | 0.381312226 | 0.390082259 | 0.399117777 | 0.408431557 | 0.416846079 | 0.423261181 |
| Tanzania | 0.259306074 | 0.262416238 | 0.264989448 | 0.267254915 | 0.269417204 | 0.272078202 | 0.275507909 | 0.279259184 | 0.28315261 | 0.287381679 | 0.291827265 | 0.296715645 | 0.301983995 | 0.307615488 | 0.313772983 | 0.320437339 | 0.327234002 | 0.334313237 | 0.34135263 | 0.348378035 | 0.355567563 | 0.36303632 | 0.370412488 | 0.377963171 | 0.385974579 | 0.394277757 | 0.402879308 | 0.411824974 | 0.420985364 | 0.430225379 | 0.438747238 | 0.446568273 |
| Zambia | 0.304008549 | 0.30584236 | 0.307316277 | 0.308780208 | 0.309223449 | 0.309995813 | 0.311326691 | 0.31308733 | 0.314926435 | 0.317396437 | 0.320625322 | 0.324401368 | 0.328680122 | 0.333721398 | 0.339488917 | 0.346048864 | 0.353540709 | 0.362450967 | 0.371688496 | 0.381667118 | 0.392470989 | 0.403475629 | 0.414574501 | 0.425492302 | 0.436244006 | 0.446744719 | 0.457122596 | 0.467532631 | 0.477887712 | 0.487911523 | 0.497485561 | 0.505948954 |
| Southern sub-Saharan Africa | 0.506946633 | 0.512377786 | 0.517569473 | 0.522772503 | 0.528063317 | 0.533379781 | 0.538780402 | 0.544134279 | 0.549141665 | 0.553884659 | 0.558490622 | 0.562511271 | 0.56604984 | 0.569215186 | 0.572389699 | 0.576115686 | 0.580684833 | 0.585672998 | 0.590405319 | 0.594585068 | 0.598835553 | 0.603346853 | 0.60803777 | 0.612843814 | 0.617452594 | 0.62183758 | 0.625919059 | 0.629761503 | 0.633410656 | 0.63694019 | 0.639881827 | 0.642200282 |
| Botswana | 0.418077748 | 0.429752286 | 0.440490843 | 0.450523614 | 0.459954967 | 0.469266513 | 0.478588352 | 0.487767857 | 0.496193622 | 0.504978826 | 0.513498223 | 0.521370182 | 0.529252964 | 0.537000179 | 0.544349223 | 0.55170697 | 0.55922162 | 0.566702496 | 0.573883805 | 0.579696412 | 0.58582415 | 0.591985457 | 0.597739553 | 0.604064731 | 0.610086049 | 0.615411124 | 0.620797575 | 0.625704953 | 0.630606841 | 0.635324093 | 0.639275298 | 0.642721629 |
| Eswatini | 0.399420955 | 0.408269969 | 0.416683379 | 0.424802995 | 0.432503472 | 0.440127047 | 0.447399238 | 0.454089854 | 0.460136984 | 0.465990845 | 0.471852843 | 0.477233374 | 0.482474423 | 0.487608829 | 0.492669159 | 0.498043849 | 0.503658294 | 0.509260219 | 0.514730896 | 0.520427398 | 0.526245802 | 0.53191391 | 0.537798023 | 0.543836416 | 0.549613848 | 0.555373739 | 0.561061186 | 0.566698493 | 0.572154807 | 0.577206305 | 0.581653071 | 0.585459713 |
| Lesotho | 0.339155125 | 0.345496571 | 0.35216963 | 0.358780457 | 0.365310801 | 0.371265315 | 0.377221202 | 0.383096648 | 0.388406478 | 0.393495001 | 0.398635802 | 0.403990126 | 0.409021657 | 0.413982571 | 0.418670919 | 0.423211228 | 0.427806732 | 0.432745038 | 0.438340567 | 0.443580127 | 0.448966476 | 0.454530098 | 0.460419689 | 0.466692256 | 0.473188157 | 0.479754456 | 0.486144827 | 0.491978155 | 0.497381791 | 0.502457317 | 0.506746655 | 0.510393066 |
| Namibia | 0.450040233 | 0.45453444 | 0.460021314 | 0.465734135 | 0.471873217 | 0.477794329 | 0.483116378 | 0.488012938 | 0.492812548 | 0.497613862 | 0.502406958 | 0.506719033 | 0.510818275 | 0.514574645 | 0.519103605 | 0.523881736 | 0.529350118 | 0.535062126 | 0.540896995 | 0.546516477 | 0.552741565 | 0.559618899 | 0.566978697 | 0.574590344 | 0.582321425 | 0.58982104 | 0.596283059 | 0.601754645 | 0.606618403 | 0.610921091 | 0.614435889 | 0.617564872 |
| South Africa | 0.541571435 | 0.546921136 | 0.552066899 | 0.557198439 | 0.562448636 | 0.567905304 | 0.573453988 | 0.579099278 | 0.584437313 | 0.589407349 | 0.594093683 | 0.597884168 | 0.600921676 | 0.603515602 | 0.606262514 | 0.609989979 | 0.61501469 | 0.62060815 | 0.625948031 | 0.630645626 | 0.635268538 | 0.639991722 | 0.644715661 | 0.649430554 | 0.653964495 | 0.658417761 | 0.662642487 | 0.666622608 | 0.670335365 | 0.674041037 | 0.677166096 | 0.679626598 |
| Eastern Cape | 0.487156629 | 0.491945768 | 0.496558751 | 0.501227233 | 0.505989096 | 0.51093299 | 0.516048444 | 0.521361921 | 0.526467367 | 0.531267891 | 0.535874875 | 0.539464836 | 0.542272959 | 0.544803397 | 0.547609206 | 0.551394614 | 0.556413416 | 0.561946556 | 0.566944819 | 0.571103982 | 0.575152262 | 0.579233196 | 0.583398915 | 0.587671831 | 0.591833658 | 0.595990218 | 0.599972411 | 0.603862936 | 0.607564965 | 0.611351819 | 0.614632221 | 0.617204696 |
| Free State | 0.547462374 | 0.552883824 | 0.557829772 | 0.562617174 | 0.567353036 | 0.572049629 | 0.576614774 | 0.581126929 | 0.585306812 | 0.58910135 | 0.592661321 | 0.595591494 | 0.598099888 | 0.600237784 | 0.602475159 | 0.605691032 | 0.610412652 | 0.615942192 | 0.621403531 | 0.626405345 | 0.631530835 | 0.63683378 | 0.642212751 | 0.647591996 | 0.652732411 | 0.657569206 | 0.661892208 | 0.665809994 | 0.669398034 | 0.672884934 | 0.675747696 | 0.677908602 |
| Gauteng | 0.634850535 | 0.638354675 | 0.641818295 | 0.64537204 | 0.649149055 | 0.653144945 | 0.657292104 | 0.661432136 | 0.6651077 | 0.668250714 | 0.671018919 | 0.672939057 | 0.674165069 | 0.6749922 | 0.676046831 | 0.678074789 | 0.681392316 | 0.685288982 | 0.689072497 | 0.692468621 | 0.695959353 | 0.699695335 | 0.703542016 | 0.707525204 | 0.711480204 | 0.715515309 | 0.71941139 | 0.72314472 | 0.726676792 | 0.730206757 | 0.733147086 | 0.735426628 |
| KwaZulu-Natal | 0.513597109 | 0.519883128 | 0.525872525 | 0.531685065 | 0.537429182 | 0.54332559 | 0.549247727 | 0.555189823 | 0.560854845 | 0.566177928 | 0.571215227 | 0.575375503 | 0.578786924 | 0.581735469 | 0.584800607 | 0.588960772 | 0.59451299 | 0.600662074 | 0.606566771 | 0.611633657 | 0.616425042 | 0.621236852 | 0.626006585 | 0.630695763 | 0.635173232 | 0.639587648 | 0.643771345 | 0.647739751 | 0.651417746 | 0.655070297 | 0.658128148 | 0.660483699 |
| Limpopo | 0.445061873 | 0.452611513 | 0.459856043 | 0.467197201 | 0.474783033 | 0.482535902 | 0.490389071 | 0.498458945 | 0.506234473 | 0.513697542 | 0.520945609 | 0.527181962 | 0.53241909 | 0.536899734 | 0.541273603 | 0.546318365 | 0.552386079 | 0.558964302 | 0.565226289 | 0.570713185 | 0.576001948 | 0.581101994 | 0.585910854 | 0.590504059 | 0.59461867 | 0.598293895 | 0.601487945 | 0.604134354 | 0.606461065 | 0.608898692 | 0.610844939 | 0.612287836 |
| Mpumalanga | 0.48855668 | 0.497037138 | 0.505426137 | 0.513684302 | 0.521975611 | 0.53017023 | 0.537754564 | 0.544821961 | 0.551184002 | 0.556967812 | 0.562283239 | 0.566625078 | 0.569931591 | 0.572361095 | 0.574670053 | 0.578043002 | 0.58284765 | 0.588373248 | 0.593841135 | 0.598896049 | 0.603910273 | 0.60897037 | 0.613827092 | 0.618471483 | 0.622857772 | 0.627092219 | 0.631117644 | 0.63480553 | 0.638104195 | 0.641287929 | 0.643954271 | 0.646057473 |
| North West | 0.511582018 | 0.516519186 | 0.521134664 | 0.525462575 | 0.529596926 | 0.533738699 | 0.537988641 | 0.542500563 | 0.546909151 | 0.551071976 | 0.555073703 | 0.558262263 | 0.560723383 | 0.562805636 | 0.56517049 | 0.568830702 | 0.574187972 | 0.580472434 | 0.586700054 | 0.592313447 | 0.597940542 | 0.603880364 | 0.609834816 | 0.615760936 | 0.621487559 | 0.627202774 | 0.632673772 | 0.637584143 | 0.64194456 | 0.646160335 | 0.649710934 | 0.652476432 |
| Northern Cape | 0.528126699 | 0.532204689 | 0.535917002 | 0.539584307 | 0.543479865 | 0.547881717 | 0.552713272 | 0.558067867 | 0.563489877 | 0.568810429 | 0.57398248 | 0.578372682 | 0.581974336 | 0.584985888 | 0.587809724 | 0.591197451 | 0.595574521 | 0.600243163 | 0.604477536 | 0.607949243 | 0.611489623 | 0.615576209 | 0.620319351 | 0.625579001 | 0.631039967 | 0.63666516 | 0.642210839 | 0.647482922 | 0.652409206 | 0.657142132 | 0.661072458 | 0.664140622 |
| Western Cape | 0.630621788 | 0.633124877 | 0.635137853 | 0.636892409 | 0.638716432 | 0.640890698 | 0.643456189 | 0.646366031 | 0.649109334 | 0.651688918 | 0.6541842 | 0.656129731 | 0.657741841 | 0.659297807 | 0.661265739 | 0.664078203 | 0.667881468 | 0.672027656 | 0.675915486 | 0.67938032 | 0.682948924 | 0.686707047 | 0.690460724 | 0.694167939 | 0.697676218 | 0.701073811 | 0.704319599 | 0.707544148 | 0.7106979 | 0.713880998 | 0.716514641 | 0.718532896 |
| Zimbabwe | 0.398559341 | 0.406052877 | 0.412083826 | 0.4179742 | 0.424051564 | 0.42892904 | 0.434187192 | 0.438141988 | 0.441093135 | 0.443246604 | 0.444841402 | 0.446354681 | 0.446970895 | 0.446002182 | 0.443696404 | 0.439651657 | 0.434405758 | 0.42916514 | 0.422448974 | 0.418330613 | 0.417305357 | 0.419729695 | 0.42574852 | 0.432881888 | 0.439997226 | 0.446552872 | 0.452473037 | 0.458424984 | 0.464379262 | 0.468384438 | 0.471573171 | 0.473819486 |
| Western sub-Saharan Africa | 0.273700004 | 0.277410732 | 0.281024681 | 0.284431723 | 0.287699583 | 0.2909832 | 0.294618466 | 0.298302443 | 0.302025797 | 0.305809779 | 0.309780958 | 0.314028092 | 0.318880999 | 0.324232893 | 0.330228268 | 0.336779707 | 0.343248175 | 0.350040166 | 0.356716457 | 0.363491543 | 0.370608753 | 0.377609144 | 0.384753386 | 0.391890443 | 0.39916209 | 0.406238201 | 0.412927287 | 0.419615019 | 0.426437347 | 0.433383889 | 0.439822327 | 0.446022979 |
| Benin | 0.218907154 | 0.222539019 | 0.226323465 | 0.230312594 | 0.234032036 | 0.238009696 | 0.241895323 | 0.245731534 | 0.249376451 | 0.253215341 | 0.257380681 | 0.261388011 | 0.265417458 | 0.269544281 | 0.273941145 | 0.27840675 | 0.282933936 | 0.287673055 | 0.292627446 | 0.297702227 | 0.302809008 | 0.307925538 | 0.313373588 | 0.319318688 | 0.325727272 | 0.33221635 | 0.338763921 | 0.345606759 | 0.352652696 | 0.360031163 | 0.366964487 | 0.373486574 |
| Burkina Faso | 0.129695615 | 0.133299128 | 0.136594798 | 0.139825465 | 0.142848973 | 0.146115673 | 0.150041686 | 0.154228401 | 0.158707048 | 0.163468067 | 0.16808434 | 0.172996395 | 0.17798199 | 0.183253539 | 0.188564995 | 0.194086128 | 0.199571952 | 0.204891092 | 0.210223295 | 0.215423374 | 0.22100103 | 0.226696632 | 0.232589135 | 0.238497491 | 0.244378901 | 0.250208261 | 0.256081774 | 0.262033009 | 0.268102073 | 0.274287092 | 0.279871317 | 0.285118402 |
| Cabo Verde | 0.276723367 | 0.282931722 | 0.288460371 | 0.294517286 | 0.302216794 | 0.310675634 | 0.319736229 | 0.329049585 | 0.338786069 | 0.349296918 | 0.359899017 | 0.370070276 | 0.379846674 | 0.389419786 | 0.398838732 | 0.408009221 | 0.417433786 | 0.427379787 | 0.43737213 | 0.446691506 | 0.455647568 | 0.464473969 | 0.472857771 | 0.480796481 | 0.488259896 | 0.495229489 | 0.501855424 | 0.508435792 | 0.515285886 | 0.522488008 | 0.528461595 | 0.533534539 |
| Cameroon | 0.303055334 | 0.309623458 | 0.315601269 | 0.320831654 | 0.325410123 | 0.329539318 | 0.333425982 | 0.337346001 | 0.34119709 | 0.344955364 | 0.348814789 | 0.352631873 | 0.356511817 | 0.360668581 | 0.36510003 | 0.369545756 | 0.374355878 | 0.379490349 | 0.384838304 | 0.390288643 | 0.396067145 | 0.402092799 | 0.408580123 | 0.415607965 | 0.42303356 | 0.430830276 | 0.438878945 | 0.4471851 | 0.455687847 | 0.464246473 | 0.472285846 | 0.479691223 |
| Chad | 0.114638829 | 0.117954634 | 0.121132832 | 0.123600072 | 0.126080572 | 0.128289492 | 0.130169484 | 0.132136021 | 0.134090304 | 0.135825261 | 0.137412374 | 0.139663692 | 0.1420417 | 0.145348052 | 0.150910868 | 0.157033036 | 0.162576402 | 0.168161062 | 0.173396784 | 0.178335361 | 0.184110621 | 0.189591609 | 0.195262749 | 0.200952884 | 0.206658836 | 0.212428697 | 0.21753218 | 0.22220695 | 0.226913881 | 0.231704755 | 0.236103083 | 0.240436019 |
| Côte d'Ivoire | 0.279320353 | 0.286492681 | 0.293451293 | 0.299743215 | 0.305210653 | 0.310105508 | 0.314965385 | 0.319843979 | 0.324712873 | 0.329201009 | 0.333015393 | 0.336545511 | 0.339977638 | 0.343348932 | 0.346375411 | 0.348727913 | 0.350715944 | 0.352727478 | 0.355087643 | 0.357938755 | 0.361205187 | 0.36408365 | 0.367831685 | 0.37235379 | 0.377575444 | 0.38349138 | 0.389825192 | 0.396693974 | 0.404091933 | 0.41172558 | 0.419040103 | 0.425941883 |
| The Gambia | 0.238714846 | 0.245010164 | 0.251087863 | 0.256996141 | 0.262424792 | 0.267696507 | 0.27295019 | 0.278274122 | 0.283946056 | 0.290070621 | 0.296354282 | 0.302543257 | 0.307712927 | 0.312930916 | 0.318615029 | 0.32356835 | 0.328151431 | 0.332640147 | 0.337315245 | 0.342389614 | 0.34799783 | 0.352869236 | 0.35837365 | 0.364255878 | 0.3699013 | 0.375840485 | 0.381569217 | 0.387273644 | 0.393155679 | 0.39918978 | 0.404524194 | 0.40971416 |
| Ghana | 0.373112005 | 0.379239256 | 0.384856767 | 0.389968494 | 0.394953663 | 0.399930204 | 0.405003047 | 0.410038073 | 0.414888532 | 0.419755496 | 0.424725212 | 0.429411846 | 0.434104128 | 0.439118213 | 0.444236977 | 0.44936002 | 0.454692737 | 0.460606245 | 0.467259067 | 0.473848047 | 0.480777892 | 0.489020974 | 0.497513932 | 0.506004647 | 0.514119061 | 0.521539202 | 0.528629586 | 0.53616994 | 0.543840539 | 0.551632027 | 0.558462173 | 0.56493039 |
| Guinea | 0.178295421 | 0.181459391 | 0.184485016 | 0.187686242 | 0.191128837 | 0.194573245 | 0.198633755 | 0.203280449 | 0.208003383 | 0.212412705 | 0.216649792 | 0.221090941 | 0.225898779 | 0.230522594 | 0.23513497 | 0.239691845 | 0.244010127 | 0.24847169 | 0.253167689 | 0.257552785 | 0.262405949 | 0.2676246 | 0.273428319 | 0.279600444 | 0.285961264 | 0.292363614 | 0.299112412 | 0.306881462 | 0.314839588 | 0.322839848 | 0.329877997 | 0.336401293 |
| Guinea-Bissau | 0.207614839 | 0.212505984 | 0.217026088 | 0.221678978 | 0.226434933 | 0.231223526 | 0.236106374 | 0.241360584 | 0.244371797 | 0.248217317 | 0.252026519 | 0.255786768 | 0.259321816 | 0.262747724 | 0.266271736 | 0.270056132 | 0.273803987 | 0.277805017 | 0.282151502 | 0.286423345 | 0.291141639 | 0.296682967 | 0.301562342 | 0.306602009 | 0.311602905 | 0.317090015 | 0.32297967 | 0.329326492 | 0.335667833 | 0.342156115 | 0.347798998 | 0.353109621 |
| Liberia | 0.235296847 | 0.238212218 | 0.238283313 | 0.235984406 | 0.232656774 | 0.228424478 | 0.222682591 | 0.219672765 | 0.219053855 | 0.222212867 | 0.231173362 | 0.239594386 | 0.248311971 | 0.251595458 | 0.25517961 | 0.25896799 | 0.262897258 | 0.267239568 | 0.271853596 | 0.276804111 | 0.282235752 | 0.288015145 | 0.294854315 | 0.302985656 | 0.31095544 | 0.318485166 | 0.325085174 | 0.331527786 | 0.337784578 | 0.343403262 | 0.34813389 | 0.352442452 |
| Mali | 0.126526428 | 0.12912815 | 0.131537449 | 0.134177054 | 0.136937829 | 0.139866225 | 0.143078916 | 0.146414326 | 0.14998673 | 0.153737336 | 0.157190028 | 0.161344448 | 0.165420696 | 0.169733502 | 0.173779552 | 0.178029844 | 0.182452468 | 0.186912995 | 0.191536746 | 0.196537651 | 0.201803536 | 0.207097221 | 0.21246506 | 0.217993867 | 0.22372998 | 0.229826263 | 0.236186231 | 0.242726135 | 0.249419731 | 0.256250746 | 0.262629219 | 0.268579941 |
| Mauritania | 0.335780942 | 0.341135175 | 0.346531288 | 0.352176572 | 0.357585912 | 0.363037695 | 0.368508328 | 0.373021339 | 0.37708025 | 0.381112297 | 0.38420538 | 0.386487416 | 0.388564478 | 0.391104879 | 0.394012934 | 0.397499105 | 0.403159467 | 0.408377122 | 0.413233621 | 0.417910609 | 0.423202436 | 0.42886729 | 0.434724376 | 0.44106891 | 0.447653881 | 0.454523488 | 0.461269545 | 0.468452365 | 0.475839507 | 0.483767738 | 0.491365603 | 0.4989451 |
| Niger | 0.08086848 | 0.082848208 | 0.084771578 | 0.08669649 | 0.088608733 | 0.090473708 | 0.092346359 | 0.094219115 | 0.096545153 | 0.098813686 | 0.100886948 | 0.103271777 | 0.105741109 | 0.108322891 | 0.110764495 | 0.113452023 | 0.116247074 | 0.118971626 | 0.121957786 | 0.124829381 | 0.127952996 | 0.130958067 | 0.134469718 | 0.13797817 | 0.141731738 | 0.145440503 | 0.149200109 | 0.15295917 | 0.156875196 | 0.160889484 | 0.164539265 | 0.168072774 |
| Nigeria | 0.305868047 | 0.308489225 | 0.311234052 | 0.31398306 | 0.316747447 | 0.319637439 | 0.323137685 | 0.326690553 | 0.330263851 | 0.3339365 | 0.338201743 | 0.343029751 | 0.349092528 | 0.355911392 | 0.363780814 | 0.372583553 | 0.38102604 | 0.389779649 | 0.398037706 | 0.406353307 | 0.414997748 | 0.423351915 | 0.431780376 | 0.440124664 | 0.448766823 | 0.457240991 | 0.465183781 | 0.472972869 | 0.480844413 | 0.488787361 | 0.496204741 | 0.503390833 |
| São Tomé and Príncipe | 0.309542852 | 0.310601028 | 0.311515447 | 0.312743558 | 0.314261087 | 0.31584392 | 0.317729288 | 0.31979567 | 0.322519219 | 0.325651916 | 0.329460109 | 0.334484786 | 0.340302837 | 0.346851981 | 0.353722384 | 0.361105881 | 0.369413007 | 0.377813517 | 0.386688944 | 0.395821238 | 0.405530666 | 0.415783728 | 0.425721315 | 0.43559246 | 0.445146237 | 0.454051473 | 0.463230082 | 0.47217093 | 0.481098204 | 0.48983823 | 0.497861409 | 0.505413747 |
| Senegal | 0.238047613 | 0.244482178 | 0.250416532 | 0.255767964 | 0.260379997 | 0.264438773 | 0.268318965 | 0.272072875 | 0.275995656 | 0.280111918 | 0.283784903 | 0.287451899 | 0.291046288 | 0.294887172 | 0.298984521 | 0.303257209 | 0.30763391 | 0.312570298 | 0.317894089 | 0.323657726 | 0.329804072 | 0.336059897 | 0.342977114 | 0.350134004 | 0.357181573 | 0.364144496 | 0.371114525 | 0.378351477 | 0.386079666 | 0.394012316 | 0.401197516 | 0.408054193 |
| Sierra Leone | 0.211569335 | 0.213013567 | 0.21422304 | 0.216089201 | 0.218277411 | 0.219822604 | 0.21996927 | 0.219089791 | 0.218584313 | 0.217943042 | 0.218492837 | 0.220391233 | 0.224585825 | 0.2292359 | 0.234131674 | 0.23954994 | 0.245647886 | 0.252523077 | 0.259781562 | 0.26705838 | 0.274270275 | 0.281673575 | 0.290338512 | 0.300736693 | 0.310348867 | 0.31699865 | 0.324091565 | 0.331213431 | 0.338341564 | 0.345770019 | 0.352412022 | 0.358665881 |
| Togo | 0.269692273 | 0.274530568 | 0.27878931 | 0.281224631 | 0.284653387 | 0.288536692 | 0.292606677 | 0.296947835 | 0.300022333 | 0.303020232 | 0.305472963 | 0.307828258 | 0.31046991 | 0.313792064 | 0.316687944 | 0.31902558 | 0.32165939 | 0.324382497 | 0.327743136 | 0.331558542 | 0.335899583 | 0.34077726 | 0.346320495 | 0.352278605 | 0.358661442 | 0.365600169 | 0.372910028 | 0.380336231 | 0.387921229 | 0.395667269 | 0.402356922 | 0.408533695 |
