# Supplementary material for: Assessing the global dengue burden: Incidence, mortality, and disability trends over three decades
Source: PLoS Negl Trop Dis. 2025 Mar 12;19(3):e0012932. doi: 10.1371/journal.pntd.0012932 (PMC11925280; doi:10.1371/journal.pntd.0012932)
Supplement: S4 Table — (DOCX) [file pntd.0012932.s004.docx]

**S4 Table Incident cases and ASIR of dengue in 1990 and 2021, and the EAPC of ASIR from 1990 to 2021.**

| **Characteristics** | **1990** | | **2021** | | **1990-2021** |
| --- | --- | --- | --- | --- | --- |
|  | **Incident Cases**  **No. (95% UI)** | **ASIR per 100,000**  **No. (95% UI)** | **Incident Cases**  **No. (95% UI)** | **ASIR per 100,000**  **No. (95% UI)** | **EAPC in ASIR**  **No. (95% CI)** |
| Overall | 26447128.76 | 481.85 | 58964184.93 | 752.04 | 1.83 |
|  | （3933185.59,51891941.54) | (70.76,946.29) | （15473438.96,106885036.40) | (196.33,1363.35) | （1.58,2.08） |
| Sex |  |  |  |  |  |
| Males | 12513019.50 | 454.24 | 27346971.82 | 694.78 | 1.76 |
|  | (1553630.18,24768277.58) | (55.41,900.38) | (6000367.20,50327387.66) | (152.25,1278.44) | (1.53,2.00) |
| Females | 13934109.26 | 511.68 | 31617213.11 | 810.65 | 1.88 |
|  | (2051682.30,27126802.53) | (74.57,998.72) | (8473243.15,56574525.87) | (215.59,1450.93) | (1.62,2.13) |
| **SDI region** |  |  |  |  |  |
| High SDI | 324498.20 | 38.03 | 601233.17 | 54.64 | 1.89 |
|  | （46667.68,790584.79) | (5.47,92.83) | （132600.81,1314069.47) | (12.22,119.21) | （0.93,2.87） |
| High-middle SDI | 1046511.90 | 98.43 | 2494215.93 | 215.66 | 3.38 |
|  | （130035.03,2806271.66) | (12.21,263.08) | （1061801.68,4307562.33) | (91.61,372.30) | （2.98,3.79） |
| Middle SDI | 13612466.10 | 782.97 | 30664156.54 | 1269.27 | 2.13 |
|  | （890229.87,29939998.16) | (51.81,1725.40) | （10546524.25,54885088.05) | (437.36,2268.00) | （1.832.43） |
| Low-middle SDI | 9294746.93 | 802.75 | 21251661.13 | 1117.70 | 1.37 |
|  | （949482.20,19993511.78) | (79.39,1725.04) | （2294596.78,45470002.43) | (123.23,2373.55) | （1.18,1.56） |
| Low SDI | 2157286.76 | 431.07 | 3932556.30 | 368.03 | -1.07 |
|  | （1251604.87,3202640.14) | (242.88,648.51) | （125885.92,9580300.76) | (11.69,884.71) | （-1.71,-0.42） |
| **GBD region** |  |  |  |  |  |
| High-income Asia Pacific | 219058.30 | 130.09 | 485712.47 | 294.01 | 3.50 |
|  | （32182.12,572882.53) | (19.12,340.20) | （107597.24,1123586.02) | (65.07,679.53) | (2.34,4.68) |
| High-income North America | 290.22 | 0.10 | 1376.40 | 0.36 | 6.75 |
|  | （47.98,985.44) | (0.02,0.35) | （40.25,7206.87) | (0.01,1.86) | (5.30,8.22) |
| Western Europe | 0 | 0 | 0 | 0 | 0 |
|  | (0,0) | (0,0) | (0,0) | (0,0) | (0,0) |
| Australasia | 5791.88 | 28.69 | 18447.84 | 58.99 | 3.76 |
|  | （403.15,17085.53) | (2,84 .63) | （5642.05,43236.80) | (18.09,139.42) | (2.93,4.59) |
| Andean Latin America | 117506.49 | 305.85 | 391707.88 | 593.22 | 2.31 |
|  | （13807.05,275359.37) | (36.08,714.51) | （163083.19,669824.19) | (247.03,1014.24) | (1.92,2.70) |
| Tropical Latin America | 6863968.09 | 4460.04 | 13043195.24 | 5774.82 | 1.59 |
|  | （405329.91,18146519.50) | (265.15,11852.45) | （3996125.94,26305621.50) | (1774.73,11624.76) | (1.08,2.11) |
| Central Latin America | 1146582.62 | 696.38 | 2886641.39 | 1140.37 | 2.86 |
|  | （190138.20,2317016.86) | (115.55,1409.02) | （1757443.51,4048875.24) | (694.53,1599.48) | (1.74,3.98) |
| Southern Latin America | 38199.74 | 77.21 | 80129.01 | 118.83 | 1.90 |
|  | （801.64,132559.25) | (1.62,267.93) | （18026.93,188076.27) | (26.67,278.96) | (1.43,2.37) |
| Caribbean | 145496.07 | 416.07 | 227073.50 | 475.93 | 0.65 |
|  | （4620.95,426127.04) | (13.34,1220.54) | （29104.17,644225.75) | (60.68,1325.33) | (-0.02,1.33) |
| Eastern Europe | 0 | 0 | 0 | 0 | 0 |
|  | (0,0) | (0,0) | (0,0) | (0,0) | (0,0) |
| Central Europe | 0 | 0 | 0 | 0 | 0 |
|  | (0,0) | (0,0) | (0,0) | (0,0) | (0,0) |
| Central Asia | 0 | 0 | 0 | 0 | 0 |
|  | (0,0) | (0,0) | (0,0) | (0,0) | (0,0) |
| North Africa and Middle East | 17977.84 | 5.23 | 53390.97 | 8.50 | 1.82 |
|  | （5946.19,56487.55) | (1.74,16.53) | （17761.11,188662.52) | (2.86,30.02) | (0.88,2.76) |
| South Asia | 12693595.10 | 1163.51 | 31812189.13 | 1726.94 | 1.48 |
|  | （136546.14,27050060.50) | (12.61,2478.46) | （1872476.97,67070659.35) | (102.48,3635.94) | (1.42,1.54) |
| Southeast Asia | 2757881.59 | 584.49 | 6728443.85 | 971.89 | 2.17 |
|  | （539807.46,7562135.02) | (114.66,1585.42) | （4787955.06,10431377.55) | (691.33,1500.41) | (1.87,2.47) |
| East Asia | 35781.60 | 3.03 | 61440.30 | 4.27 | 1.15 |
|  | （3515.52,119644.88) | (0.30,10.15) | （15719.84,155194.76) | (1.11,10.66) | (0.99,1.30) |
| Oceania | 24034.46 | 370.69 | 63970.37 | 486.03 | 1.54 |
|  | （5327.09,63232.24) | (81.90,969.58) | （28313.99,126070.14) | (211.17,953.78) | (1.19,1.88) |
| Western Sub-Saharan Africa | 720737.84 | 378.12 | 2476656.12 | 512.53 | 1.08 |
|  | （435.16,2626893.34) | (0.22,1357.39) | （120934.32,8632874.73) | (25.35,1792.58) | (0.98,1.18) |
| Eastern Sub-Saharan Africa | 1586665.26 | 829.74 | 387627.24 | 94.37 | -9.06 |
|  | （121293.93,4159409.80) | (63.78,2162.22) | （6547.47,1462385.76) | (1.55,364.39) | (-11.01,-7.06) |
| Central Sub-Saharan Africa | 72872.67 | 133.39 | 245096.55 | 178.08 | 1.09 |
|  | （887.76,467311.63) | (1.64,850.51) | （11852.44,1403021.73) | (8.62,1019.40) | (0.99,1.18) |
| Southern Sub-Saharan Africa | 689.00 | 1.23 | 1086.66 | 1.32 | -2.22 |
|  | （33.45,4553.10) | (0.06,8.10) | （57.33,7292.00) | (0.07,8.83) | (-3.06,-1.38) |

No: number; ASIR: the age-standardized incidence rate; EAPC: estimated annual percentage change; UI: uncertainty interval; CI: confidential interval; SDI: the socio-demographic index.
