# Supplementary material for: Assessing the global dengue burden: Incidence, mortality, and disability trends over three decades
Source: PLoS Negl Trop Dis. 2025 Mar 12;19(3):e0012932. doi: 10.1371/journal.pntd.0012932 (PMC11925280; doi:10.1371/journal.pntd.0012932)
Supplement: S5 Table — (DOCX) [file pntd.0012932.s005.docx]

**S5 Table Deaths Cases and ASDR of dengue in 1990 and 2021, and the EAPC of ASDR from 1990 to 2021.**

| **Characteristics** | **1990** | | **2021** | | **1990-2021** |
| --- | --- | --- | --- | --- | --- |
|  | **Death Cases**  **No. (95% UI)** | **ASDR per 100000000**  **No. (95% UI)** | **Death Cases**  **No. (95% UI)** | **ASDR per 100000000**  **No. (95% UI)** | **EAPC in ASDR**  **No. (95% CI)** |
| Overall | 14315.03 | 268.01 | 29075.97 | 376.47 | 1.70 |
|  | (11102.71,18652.24) | (212.91,333.43) | (17627.53,38980.49) | (227.85,506.42) | (1.45,1.94) |
| Sex |  |  |  |  |  |
| Males | 7721.61 | 295.36 | 15877.57 | 424.19 | 1.76 |
|  | (5874.61,10494.06) | (233.97,377.39) | (9078.85,21524.91) | (245.43,572.58) | (1.53,2.00) |
| Females | 6593.41 | 245.97 | 13198.40 | 331.63 | 1.57 |
|  | (4863.77,8932.77) | (187.29,321.96) | (8185.40,18493.37) | (205.75,466.08) | (1.31,1.83) |
| **SDI region** |  |  |  |  |  |
| High SDI | 14.64 | 1.60 | 30.50 | 1.53 | 4.37 |
|  | (9.09,22.42) | (1.00,2.42) | (14.04,49.97) | (0.71,2.48) | (2.66,6.10) |
| High-middle SDI | 1057.84 | 108.19 | 1549.02 | 139.35 | 0.94 |
|  | (773.33,1504.57) | (77.58,156.29) | (930.20,2110.74) | (83.18,190.80) | (0.62,1.26) |
| Middle SDI | 6765.52 | 402.21 | 13064.58 | 598.27 | 1.98 |
|  | (4826.82,9847.63) | (308.80,551.30) | (8153.81,17809.94) | (373.72,814.14) | (1.69,2.27) |
| Low-middle SDI | 5781.52 | 586.78 | 12680.97 | 840.87 | 1.75 |
|  | (4470.51,7165.41) | (465.26,716.17) | (7342.14,17711.50) | (500.79,1175.97) | (1.51,1.99) |
| Low SDI | 681.08 | 200.40 | 1732.61 | 302.05 | 2.04 |
|  | (484.26,947.90) | (139.60,277.68) | (848.18,2766.38) | (168.77,457.10) | (1.70,2.38) |
| **GBD region** |  |  |  |  |  |
| High-income Asia Pacific | 0.78 | 0.56 | 1.45 | 0.38 | 1.11 |
|  | (0.49,1.23) | (0.37,0.83) | (0.73,2.41) | (0.19,0.61) | (0.09,2.15) |
| High-income North America | 0.06 | 0.02 | 0.54 | 0.17 | 8.60 |
|  | (0.04,0.08) | (0.02,0.03) | (0.28,0.83) | (0.09,0.26) | (7.59,9.63) |
| Western Europe | 9.60 | 1.85 | 0.11 | 0.01 | -8.72 |
|  | (4.53,16.85) | (0.88,3.29) | (0.04,0.20) | (0,0.02) | (-14.17,-2.92) |
| Australasia | 0.004 | 0.02 | 0.04 | 0.11 | 7.76 |
|  | (0.002,0.009) | (0.01,0.04) | (0.01,0.11) | (0.03,0.29) | (6.30,9.24) |
| Andean Latin America | 3.90 | 10.60 | 10.69 | 17.21 | 3.44 |
|  | (2.45,5.50) | (6.83,14.76) | (5.33,16.80) | (8.69,27.02) | (1.08,5.85) |
| Tropical Latin America | 28.09 | 22.30 | 558.10 | 230.67 | 10.79 |
|  | (20.35,38.76) | (15.94,30.62) | (352.93,746.49) | (145.97,309.12) | (9.23,12.37) |
| Central Latin America | 43.65 | 30.60 | 292.41 | 122.13 | 5.51 |
|  | (34.30,52.45) | (23.39,37.19) | (161.13,457.44) | (66.64,195.72) | (4.07,6.96) |
| Southern Latin America | 0.28 | 0.68 | 0.03 | 0.03 | -9.14 |
|  | (0.18,0.40) | (0.44,0.94) | (0.01,0.05) | (0.01,0.06) | (-10.18,-8.09) |
| Caribbean | 6.06 | 18.45 | 12.05 | 24.61 | 2.38 |
|  | (4.30,7.98) | (13.10,23.82) | (5.53,19.99) | (11.25,41.19) | (0.30,4.52) |
| Eastern Europe | 0 | 0 | 0 | 0 | 0 |
|  | (0,0) | (0,0) | (0,0) | (0,0) | (0,0) |
| Central Europe | 0 | 0 | 0 | 0 | 0 |
|  | (0,0) | (0,0) | (0,0) | (0,0) | (0,0) |
| Central Asia | 0 | 0 | 0 | 0 | 0 |
|  | (0,0) | (0,0) | (0,0) | (0,0) | (0,0) |
| North Africa and Middle East | 12.69 | 5.81 | 15.75 | 3.85 | 0.12 |
|  | (7.25,19.53) | (3.46,8.51) | (8.38,26.66) | (2.20,6.17) | (-1.39,1.66) |
| South Asia | 4801.95 | 643.11 | 15446.82 | 1080.95 | 2.45 |
|  | (3450.04,6452.90) | (460.60,862.40) | (8464.09,22362.18) | (618.85,1536.08) | (2.12,2.78) |
| Southeast Asia | 9340.02 | 1866.72 | 12674.99 | 2041.74 | 0.69 |
|  | (6060.69,14351.12) | (1301.03,2699.43) | (7893.45,17448.99) | (1280.87,2820.35) | (0.49,0.89) |
| East Asia | 46.37 | 4.33 | 29.79 | 1.73 | -0.66 |
|  | (28.25,64.84) | (2.63,6.03) | (13.66,48.64) | (0.82,2.79) | (-1.87,0.57) |
| Oceania | 10.94 | 230.04 | 3.66 | 53.65 | -1.96 |
|  | (8.42,15.22) | (159.62,342.94) | (1.53,6.42) | (22.95,95.25) | (-2.82,-1.09) |
| Western Sub-Saharan Africa | 1.30 | 0.98 | 2.22 | 0.80 | 0.69 |
|  | (0.69,1.92) | (0.33,1.58) | (0.56,3.73) | (0.16,1.36) | (-0.76,2.16) |
| Eastern Sub-Saharan Africa | 9.16 | 3.92 | 26.59 | 5.05 | 1.40 |
|  | (3.33,19.08) | (2.19,6.77) | (14.86,49.31) | (2.74,9.22) | (1.06,1.74) |
| Central Sub-Saharan Africa | 0.14 | 0.78 | 0.69 | 1.14 | 1.71 |
|  | (0.05,0.27) | (0.21,1.69) | (0.22,1.43) | (0.40,2.18) | (1.49,1.93) |
| Southern Sub-Saharan Africa | 0.02 | 0.05 | 0.02 | 0.02 | -2.08 |
|  | (0,0.06) | (0.01,0.12) | (0,0.05) | (0.01,0.06) | (-2.59,-1.56) |

No: number; ASDR: the age-standardized death rate; EAPC: estimated annual percentage change; UI: uncertainty interval; CI: confidential interval; SDI: the socio-demographic index.
