# Supplementary material for: Assessing the global dengue burden: Incidence, mortality, and disability trends over three decades
Source: PLoS Negl Trop Dis. 2025 Mar 12;19(3):e0012932. doi: 10.1371/journal.pntd.0012932 (PMC11925280; doi:10.1371/journal.pntd.0012932)
Supplement: S6 Table — (DOCX) [file pntd.0012932.s006.docx]

**S6 Table DALYs and age-standardized DALYs rate of dengue in 1990 and 2021, and the EAPC of age-standardized DALYs rate from 1990 to 2021.**

| **Characteristics** | **1990** | | **2021** | | **1990-2021** |
| --- | --- | --- | --- | --- | --- |
|  | **DALYs**  **No. (95% UI)** | **Age-standardized**  **DALYs rate**  **per 100 000**  **No. (95% UI)** | **DALYs**  **No. (95% UI)** | **Age-standardized**  **DALYs rate per 100 000**  **No. (95% UI)** | **EAPC in Age-standardized DALYs rate**  **No. (95% CI)** |
| Overall | 1248669.44 | 21.63 | 2076524.69 | 27.76 | 1.33 |
|  | (876050.15,1552996.46) | (15.09,26.92) | (1056228.35,3130717.69) | (14.21,41.65) | (1.10,1.57) |
| Sex |  |  |  |  |  |
| Males | 663835.63 | 22.64 | 1123515.85 | 29.85 | 1.42 |
|  | (418010.78,853938.31) | (14.52，28.74） | (550671.89,1655322.25) | (14.72，43.70） | (1.20,1.65) |
| Females | 584833.81 | 20.65 | 953008.84 | 25.62 | 1.22 |
|  | (394269.20,715961.45) | (13.99，25.67） | (494292.17,1492008.00) | (13.38，39.97） | (0.96,1.47) |
| **SDI region** |  |  |  |  |  |
| High SDI | 3539.95 | 0.42 | 6494.66 | 0.58 | 1.96 |
|  | (698.25,9046.54) | (0.09,1.07) | (1606.63,15294.08) | (0.14,1.39) | (1.04,2.90) |
| High-middle SDI | 87623.66 | 8.92 | 110148.34 | 11.39 | 1.00 |
|  | (61326.63,121292.73) | (6.20,12.61) | (64062.56,156180.11) | (6.67,16.03) | (0.67,1.33) |
| Middle SDI | 630452.75 | 33.64 | 1045828.36 | 48.78 | 1.87 |
|  | (403745.10,805798) | (21.46,42.85) | (575251.63,1544203.75) | (27.32,71.02) | (1.58,2.16) |
| Low-middle SDI | 463610.47 | 35.92 | 793856.40 | 43.35 | 1.06 |
|  | (326499.31,593002.68) | (24.95,47.94) | (351111.93,1290420.25) | (19.69,69.74) | (0.86,1.25) |
| Low SDI | 62235.85 | 11.84 | 118794.90 | 12.34 | 0.34 |
|  | (43901.59,85211.83) | (8.11,16.08) | (38238.80,222378.23) | (4.44,22.08) | (0.05,0.64) |
| **GBD region** |  |  |  |  |  |
| High-income Asia Pacific | 2175.23 | 1.30 | 4713.22 | 2.90 | 3.50 |
|  | (268.36,5921.93) | (0.17,3.53) | (875.96,12044.73) | (0.52,7.39) | (2.33,4.68) |
| High-income North America | 7.18 | 0 | 42.09 | 0.02 | 8.00 |
|  | (3.72,14.11) | (0,0.01) | (15.85,121.12) | (0.01,0.04) | (7.16,8.85) |
| Western Europe | 90.77 | 0.02 | 1.49 | 0 | -7.95 |
|  | (43.05,157.67) | (0.01,0.03) | (0.51,2.74) | (0,0) | (-12.28,-3.41) |
| Australasia | 59.70 | 0.30 | 191.74 | 0.61 | 3.81 |
|  | (3.75,200.24) | (0.02,0.99) | (49.82,498.28) | (0.16,1.60) | (2.97,4.65) |
| Andean Latin America | 1423.42 | 3.58 | 4327.20 | 6.56 | 2.58 |
|  | (304.36,3500.82) | (0.74,8.91) | (1845.49,8129.16) | (2.80,12.31) | (1.69,3.47) |
| Tropical Latin America | 67757.32 | 43.56 | 143869.56 | 63.76 | 2.23 |
|  | (5126.05,176964.63) | (3.32,113.62) | (50995.10,306624.28) | (22.43,136.04) | (1.69,2.76) |
| Central Latin America | 14065.85 | 8.26 | 43074.33 | 17.53 | 3.63 |
|  | (4220.81,29841.91) | (2.37,17.68) | (24432.76,67687.04) | (9.92,27.60) | (2.67,4.59) |
| Southern Latin America | 399.89 | 0.81 | 819.81 | 1.22 | 1.80 |
|  | (14.68,1508.51) | (0.03,3.05) | (149.57,2211.01) | (0.22,3.28) | (1.33,2.28) |
| Caribbean | 1785.22 | 5.02 | 2751.25 | 5.83 | 1.12 |
|  | (304.22,5235.65) | (0.84,14.80) | (546.46,7216.81) | (1.17,15.48) | (-0.04,2.29) |
| Eastern Europe | 0 | 0 | 0 | 0 | 0 |
|  | (0,0) | (0,0) | (0,0) | (0,0) | (0,0) |
| Central Europe | 0 | 0 | 0 | 0 | 0 |
|  | (0,0) | (0,0) | (0,0) | (0,0) | (0,0) |
| Central Asia | 0 | 0 | 0 | 0 | 0 |
|  | (0,0) | (0,0) | (0,0) | (0,0) | (0,0) |
| North Africa and Middle East | 894.99 | 0.25 | 1035.07 | 0.18 | 0.82 |
|  | (497.55,1515.96) | (0.15,0.41) | (445.39,2669.40) | (0.08,0.45) | (-1.03,2.71) |
| South Asia | 388993.59 | 35.79 | 931668.08 | 53.46 | 1.88 |
|  | (197761.07,625033.32) | (17.97,57.41) | (324113.44,1630476.70) | (19.54,91.91) | (1.64,2.12) |
| Southeast Asia | 742194.35 | 134.99 | 909114.20 | 147.04 | 0.74 |
|  | (469882.43,1158430.46) | (88.76,204.74) | (589034.85,1241630.50) | (95.32,200.97) | (0.54,0.94) |
| East Asia | 3889.78 | 0.34 | 1360.02 | 0.09 | -3.38 |
|  | (2247.11,5661.56) | (0.20,0.49) | (568.26,2540.32) | (0.04,0.18) | (-3.94,-2.8) |
| Oceania | 945.79 | 13.43 | 766.78 | 6.04 | -0.02 |
|  | (645.03,1424.32) | (8.60,20.83) | (347.48,1540.46) | (2.73,11.88) | (-0.74,0.71) |
| Western Sub-Saharan Africa | 7124.21 | 3.68 | 24191.21 | 4.93 | 1.08 |
|  | (60.22,27505.24) | (0.02,14.27) | (1156.70,83576.17) | (0.24,17.05) | (0.99,1.17) |
| Eastern Sub-Saharan Africa | 16106.09 | 8.17 | 5940.78 | 1.28 | -7.81 |
|  | (1432.56,42353.59) | (0.69,21.51) | (1374.58,17522.87) | (0.23,4.12) | (-9.69,-5.89) |
| Central Sub-Saharan Africa | 747.48 | 1.36 | 2645.58 | 1.91 | 1.14 |
|  | (12.48,4636.78) | (0.02,8.41) | (125.85,14646.47) | (0.09,10.31) | (1.06,1.22) |
| Southern Sub-Saharan Africa | 8.58 | 0.02 | 12.28 | 0.01 | -2.21 |
|  | (1.05,50.14) | (0,0.09) | (1.13,79.37) | (0,0.10) | (-3.01,-1.42) |

No: number; DALYs: the disability-adjusted life-years; EAPC: estimated annual percentage change; UI: uncertainty interval; CI confidential interval; SDI: the socio-demographic index.
