# Supplementary material for: Assessing the global dengue burden: Incidence, mortality, and disability trends over three decades
Source: PLoS Negl Trop Dis. 2025 Mar 12;19(3):e0012932. doi: 10.1371/journal.pntd.0012932 (PMC11925280; doi:10.1371/journal.pntd.0012932)
Supplement: S7 Table — (DOCX) [file pntd.0012932.s007.docx]

**S7 Table. Top 10 Countries/Regions with the Highest EAPC in ASIR, ASDR, and Age-Standardized DALYs Rate from 1990 to 2021.**

ASIR: the age-standardized incidence rate; EAPC: estimated annual percentage change; CI: confidential interval;
